# Aligning protein-generative models to experimental fitness with ProteinDPO

**DOI:** 10.1038/s41592-026-03137-3

**Published:** 2026-08-14

**Authors:** Talal Widatalla, Ashir A. Borah, Samuel H. King, Claudia L. Driscoll, Rafael Rafailov, Brian L. Hie

**Affiliations:** 1 Stanford University, Stanford, CA USA; 2 Arc Institute, Palo Alto, CA USA; 3 University of California, San Francisco, San Francisco, CA USA

**Keywords:** Membrane proteins, Computational models, Computational biophysics, Protein design, Drug discovery

## Abstract

Biological generative models can predict biological functions without task-specific training data but often under-perform specialized models. This is due to a fundamental ‘alignment gap’, where the rules learned during unsupervised training are not related to the function of interest. Here we demonstrate how to provide task-specific information without losing the general knowledge learned during pretraining by using direct preference optimization to align a structure-conditioned protein language model to preferentially generate stable protein sequences. Our aligned model, ProteinDPO, achieves stability prediction competitive to task-specific models and consistently outperforms unsupervised and fine-tuned versions of the model. Notably, ProteinDPO generalizes beyond its training data to enable stabilization and improved binding affinity prediction of large multichain protein complexes. When applied to stabilization of the hemagglutinin trimer, a primary component of influenza vaccines, ~80% of designs achieve increased or similar stability compared with the native hemagglutinin and up to 32 °C improvements from recently emerged mammalian strains. Our results demonstrate how to augment generative models with biophysical information and, more broadly, provide a general framework for the alignment of biological foundation models.

## Abstract (web summary)

This Article demonstrates that direct preference optimization (DPO) can be used to effectively align an unsupervised structure-conditioned language model with biophysical information. The aligned model, ProteinDPO, achieves stability prediction competitive with that of task-specific models and consistently outperforms unsupervised and fine-tuned versions of the model.

## Main

Machine learning models for biological structure and function prediction have seen tremendous improvements due to large-scale unsupervised training on databases such as UniProt and the Protein Data Bank (PDB)^1–7^. One such class of models are structure-informed language models or ‘inverse-folding’ models, which generate protein sequences that are compatible with a given structure^5,8,9^. These models are surprisingly capable of guiding improvements in binding affinity^10^ and improving protein expression, solubility and stability^11^ despite not observing these properties, nor any explicit biophysical information, during training.

These capabilities most likely emerge because a diverse training dataset naturally contains examples that possess these properties. For example, many of the structures in the PDB are of stable or stabilized proteins^12^, so models trained on the PDB demonstrate modest zero-shot stability prediction. However, evolutionary selection does not only optimize natural proteins for stability. Consistent with this, unsupervised models are still inferior at stability scoring compared with supervised models such as ThermoMPNN^13^. Using a model trained on unlabeled data for stability-related tasks results in an ‘alignment gap’: a disagreement between the information learned by the unsupervised model and the specific definition of protein fitness.

A solution would be to explicitly provide the generative model with task-specific information while retaining the model’s generative capabilities. A simple way of doing this is with supervised fine-tuning (SFT) in which we further train the model on a curated set of positive examples according to a property of interest^14,15^. However, SFT only maximizes the likelihood over a set of positively labeled samples, making it easy for the model to overfit to the fine-tuning dataset and lose the general information learned during unsupervised pretraining. More broadly, finding better ways to incorporate experimental fitness information into protein-generative models is an important open question.

**Fig. 1:**
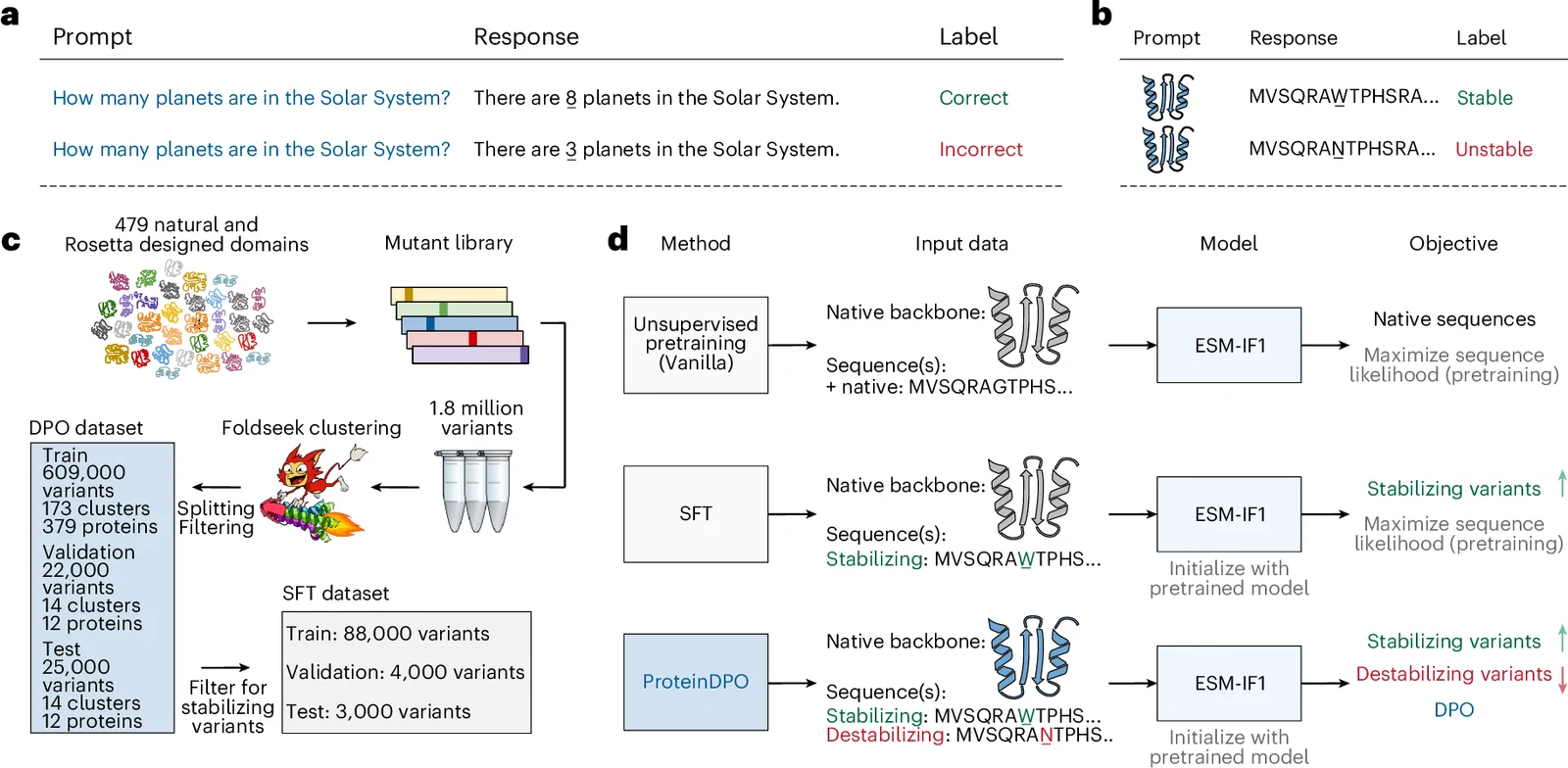
Dataset curation and model training. **a**, DPO was developed to align the responses of language models to labeled human preferences given a text prompt. **b**, Analogously, we align a structure-conditioned language model to perform thermostable sequence design using protein backbone conditioning as a ‘prompt’, sequence generation as a ‘response’ and experimental fitness as ‘preference’ data. **c**, Overview of the curation of DPO and SFT datasets from the Megascale dataset, which contains stability measurements of~1.84 million sequence variants from 479 protein domains from the PDB and Rosetta design^18,46,47^. While filtering out unreliable ΔΔ*G* measurements, we also structurally cluster the protein domains with FoldSeek at a threshold of 0.5 similarity to alleviate leakage between training splits, resulting in a final dataset with ~660,000 measurements across 403 domains. From these same splits used for DPO, variants with stability superior to the native protein (ΔΔ*G* < 0) were selected for SFT. **d**, Overview of the training procedure for the models compared in this study: vanilla ESM-IF1, SFT ESM-IF1 and ProteinDPO. Vanilla ESM-IF1 was developed and trained by Hsu et al.^5^ with maximum likelihood training on structure-conditioned, autoregressive token prediction with protein domains from PDB (CATH)^48^ and AlphaFold2-predicted^2^ structures (AF2)^5^. The SFT model was trained in the same manner but initialized with the vanilla weights and trained on stabilizing variants from the curated Megascale dataset. ProteinDPO was trained on the curated Megascale on variants of all fitness with a DPO objective (Methods). Foldseek logo reproduced under GNU General Public License v3.0.

Direct preference optimization (DPO) is an algorithm for lightweight alignment of generative models^16^. DPO enables us to explicitly inform the model of examples that may appear similar at a surface level but are actually very different when considered according to a property of interest (Fig. 1a,b). This is especially useful in biological settings where single amino-acid changes can have global effects on stability and structure^17^. By providing the model with positive and negative labels of similar proteins, DPO discourages overfitting to a set of positive examples and uses the full fitness landscape to inform the model’s predictions.

In this study, we demonstrate alignment of a generative model with experimental fitness data by using DPO to imbue stability information into a pretrained structure-conditioned protein language model, ESM-IF1^5^, using a curated dataset from experimental measurements from the ‘Megascale’ stability dataset^18^ containing approximately 660,000 sequence variants across 403 diverse protein domains (Fig. 1c,d; Methods). Our aligned model, which we refer to as ProteinDPO (Protein Direct Preference Optimization), substantially improves the stability scoring of vanilla (unsupervised) ESM-IF1 and a model trained via SFT on a subset of stable variants from Megascale (Fig. 1d, Extended Data Table 4 and Extended Data Fig. 6) and even performs competitively to supervised and physics-based models on single-site substitution stability prediction. Despite being trained solely on the relative changes in stability (ΔΔ*G*) of one or two substitutions in small protein monomers, ProteinDPO generalizes to improved binding affinity scoring of large complexes and thermal stability of highly diverse and variable length antibodies, which are regimes inaccessible to several supervised models. Beyond scoring, generating new sequences with ProteinDPO results in designs that have higher stability compared with the native protein and to generations produced by the vanilla and SFT models when evaluated with an in silico force field.

To illustrate the practical utility of this approach in addressing urgent biological challenges, we applied ProteinDPO to the stabilization of H5N1 influenza hemagglutinin (HA)—a critical need given the recent emergence of H5N1 variants across mammalian populations (including human cases) and their pandemic potential^19^. HA undergoes a conformational change to enable fusion of the virus to the host membrane, and stabilization of the prefusion conformation is important for efficacious immune response^20^. However, the prefusion state presents a formidable obstacle for vaccine development due to its propensity to be unstable, have poor expression, and fail to trimerize^20,21^. We used ProteinDPO to generate variants that improve the stability of the prefusion state and maintain antigenicity, which is a challenging problem due to the vast search space of possible HA variants. Despite this, with only 45 total designs, ProteinDPO identified 27 stabilizing variants, achieving up to a 17 °C improvement in melting temperature while preserving binding to broadly neutralizing antibodies. We also show these results can generalize to recent H5N1 strains of concern, achieving up to a 32 °C improvement in melting temperature in variants from 2024. These results demonstrate ProteinDPO’s capacity for rapid response to emerging pathogens and establish DPO as an effective method for aligning a generative model with an experimental fitness landscape.

## Results

In large language models of text, directly sampling from a pretrained model (such as GPT-4) is not practically useful, as it produces sequences that resemble raw unstructured data from the Internet (GPT-4’s training data). In practice, these models need to be aligned with human ‘preference’ labels to produce useful responses to a user’s prompt; for example, aligning GPT-5 to become a helpful question-answering system like ChatGPT^22^. Alignment is accomplished with feedback-based reinforcement learning (RL) or with lighter-weight alternatives such as DPO^16^ (see the Methods for additional details on this procedure).

We repurposed DPO, originally designed to align the responses of natural language models to text prompts using human preference labels (Fig. 1a), to instead align the generation of protein sequences (‘responses’) given their native structure (‘prompts’) using experimental stability measurements (‘preferences’) (Fig. 1b). We implemented the previously described DPO reward models that utilize preference-ordered pairs and rankings as input (referred to as ‘paired’ and ‘ranked’, respectively)^16^. In this study, we also derive a third, new DPO training objective (‘weighted’) that directly incorporates scalar labeled data (Methods).

### Megascale training and relative stability prediction performance

**Fig. 2:**
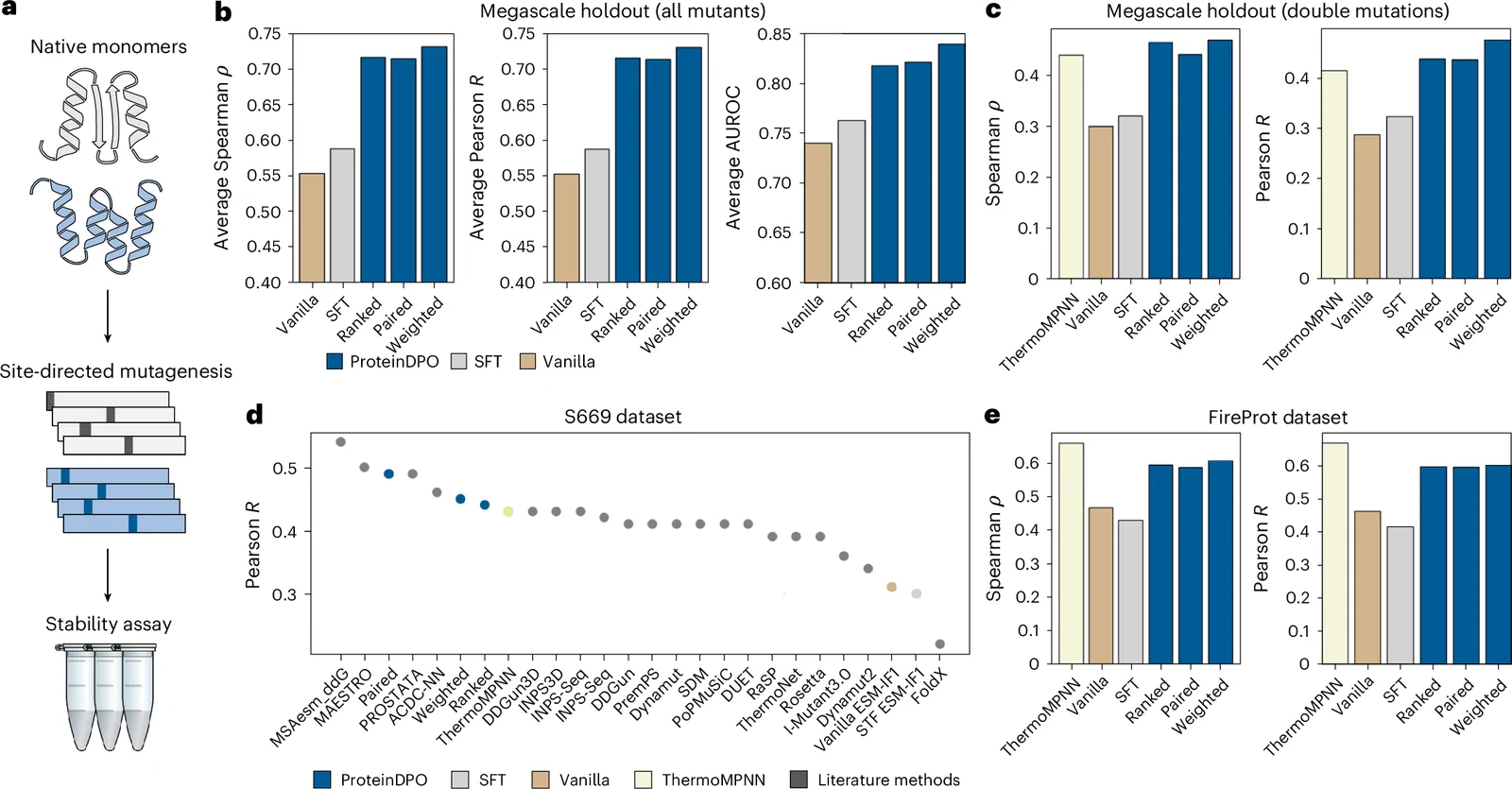
ProteinDPO achieves competitive mutational stability prediction. **a**, We benchmarked models using experimental protein stability datasets FireProt^27^ and S669^26^. These dataset are compiled from small-scale mutagenesis experiments across diverse monomeric proteins, in which certain residues are mutated, variants are expressed and their stability is measured experimentally. **b**, Performance of models on predicting stability changes upon mutation (ΔΔ*G*) in curated Megascale holdout data, including variants with one or two substitutions. We report the correlation of sequence likelihoods with their experimentally measured ΔΔ*G* averaged across each native protein in the holdout data. AUROC measures the models’ ability to classify between variants which increase (ΔΔ*G* < 0) or decrease (ΔΔ*G* > 0) stability compared with the native sequence. Distributional plots of the individual values are included in Extended Data Fig. 1. **c**, Trained on the same training data as ThermoMPNN, we show the correlation between all model predictions (ThermoMPNN) or likelihoods (Vanilla, SFT and ProteinDPO) with ΔΔ*G* of all double-mutation variants in ThermoMPNN holdout data^13^. As ThermoMPNN cannot score multiple mutations, we add the predicted ΔΔ*G* of the two respective single-mutations as a proxy. **d**, Performance of ProteinDPO, SFT, vanilla ESM-IF1 and literature methods on the S669 dataset. Performance of literature methods are provided from Cuturello et al.^25,26^. **e**, Performance of models on predicting stability changes after single-site substitutions (ΔΔ*G*) with a version of the FireProt dataset^27^ filtered of proteins homologous to those in Megascale^13^.

All ProteinDPO models were trained on the Megascale dataset with the DPO objective using native backbones and stability ordered pairs, rankings or scalar-labeled sets of sequence variants as input, depending on the reward model (Methods). To prevent data leakage between training and evaluation splits, we structurally clustered the variants by native structure with FoldSeek^23^ and randomly distributed clusters across splits. To determine whether DPO and SFT training improved upon the zero-shot capability of vanilla ESM-IF1 to score changes in stability upon mutation (ΔΔ*G*) (Fig. 2a), we evaluated the average correlation of sequence likelihoods with ΔΔ*G* across each native protein in the holdout set (Fig. 2b). Although the SFT model modestly improves (Pearson *R* = 0.59, Spearman *ρ* = 0.57) from vanilla ESM-IF1 (Pearson *R* = 0.55, Spearman *ρ* = 0.53), ProteinDPO clearly surpasses both models (Pearson *R* = 0.72–0.73, Spearman *ρ* = 0.69–0.72). We also report the area under the receiver operating characteristic curve (AUROC) similarly averaged across each protein in the holdout set, representing the models’ ability to distinguish between stabilizing (ΔΔ*G* < 0) or destabilizing (ΔΔ*G* > 0) variants and observe the same trend in the vanilla (AUROC of 0.74), SFT (AUROC of 0.76) and ProteinDPO models (AUROC of 0.82–0.84).

In addition, we sought to evaluate performance specifically on Megascale variants that have multiple mutations relative to the native sequence. Along with SFT and vanilla ESM-IF1, we compared with ThermoMPNN, a state-of-the-art supervised model based on ProteinMPNN^9^ also trained on Megascale. For this comparison, we retrained our models on the training split from Diekhaus et al.^13^ and evaluated on the double-mutation variants belonging to their holdout proteins. Notably, ProteinDPO can be trained with double-mutation variants (although we did not for this evaluation for a fair comparison) and can also naturally evaluate the likelihood of multiple mutations. By contrast, the architecture and training procedure of ThermoMPNN restricts the model to single mutations.

We found that all models perform worse when scoring variants with multiple mutations (Pearson *R* and Spearman *ρ* = 0.3–0.5) (Fig. 2c) compared with their ability to score single-site substitutions (Pearson *R* and Spearman *ρ* = 0.5–0.8)^13^, suggesting the nonlinear effects of simultaneous mutations, which can energetically influence one another^24^, pose a challenge to stability prediction. When we score these variants as a linear combination of single mutations by adding likelihoods (ProteinDPO) or scores (ThermoMPNN) of only the individual mutations, ProteinDPO underperforms ThermoMPNN (ProteinDPO Pearson *R* = 0.36–0.40, ThermoMPNN Pearson *R* = 0.41) (Extended Data Table 1). However, when we use ProteinDPO to score variants by the likelihood summed across the whole sequence, all ProteinDPO models surpass ThermoMPNN (Pearson *R* = 0.44–0.48) (Fig. 2c), which is fundamentally constrained to the ‘additive’ regime. This suggests ProteinDPO, even when trained only on single-mutation variants, benefits from synthesizing information across the full sequence to capture nonlinear effects of multiple mutations.

### Comparison with other methods for predicting relative stability

Next, we evaluated if ProteinDPO, trained on Megascale proteins (40–72 residues), can generalize to other datasets with larger (up to ~300 residues) and unseen folds: S669 and FireProt. These datasets are compilations of experimental stability measurements of derived from several small-scale mutagenesis experiments of native proteins (Fig. 1a) and are popular benchmarks of protein stability predictors. To alleviate data leakage with S669, we retrained ProteinDPO models on a S669-Homolog-free Megascale training set from Cutrello et al.^25^, and to alleviate leakage with FireProt, we evaluated on a Megascale-Homolog-free FireProt curation from Diekhaus et al.^13^.

We observed that ProteinDPO ranks highly amongst supervised and physics-based ΔΔ*G* predictors (Fig. 2d), some of which were also trained on Megascale. In addition, we find ProteinDPO is competitive with ThermoMPNN^13,25^ on the respective S669^26^ (ProteinDPO Pearson *R* = 0.44–0.47, ThermoMPNN Pearson *R* = 0.43) (Fig. 2d) and FireProt^27^ (ProteinDPO Pearson *R* = 0.6, ThermoMPNN Pearson *R* = 0.65) (Fig. 2e) test sets. Interestingly, we find the SFT model, despite improving upon vanilla ESM-IF1 on Megascale holdout prediction (Pearson *R* increase of 0.03, Spearman *ρ* increase of 0.04), does slightly worse on the FireProt (Pearson *R* decrease of 0.05, Spearman *ρ* decrease of 0.04) and S669 datasets (Pearson *R* decrease of 0.01), while the improvement of ProteinDPO over the Vanilla model generalizes to FireProt (Pearson *R* and Spearman *ρ* increases of 0.13–0.14) and S669 (Pearson *R* and Spearman *ρ* increases of 0.13–0.16) as well, evidence that the SFT model has overfit to the training data while ProteinDPO has incorporated generalizable stability information.

### Generalization to diverse design tasks

**Fig. 3:**
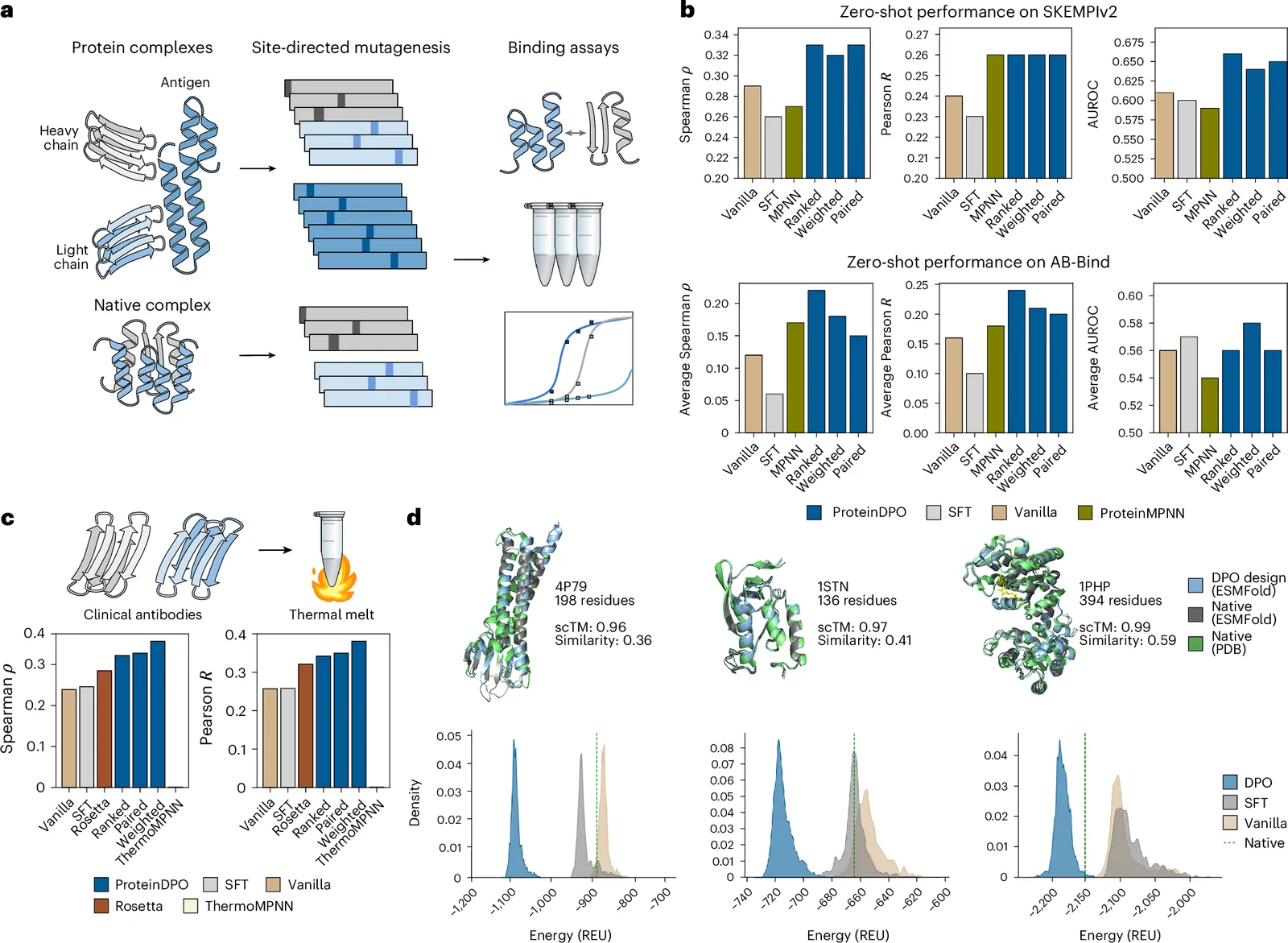
ProteinDPO generalizes beyond stability prediction to diverse tasks. **a**, We benchmarked models using binding affinity mutagenesis studies, which mutate one or more protein chains that form a bound complex and subsequently measure the resulting binding affinity of the complex. **b**, Zero-shot correlation and AUROC of model likelihoods with protein–protein binding affinity from the SKEMPIv2 dataset^28^. Zero-shot correlation and AUROC of complex-conditioned model likelihoods with ΔΔ*G* of antibody–antigen binding from the AB-Bind dataset^29^, averaged across each complex. AUROC measures a model’s ability to classify mutations that increase or decrease affinity relative to the original complex. Distributional plots of the individual values for the AB-Bind dataset are included in Extended Data Fig. 1. **c**, We benchmarked models using datasets of thermal unfolding measurements (determination of the temperature which unfolds half the concentration of a protein) of antibodies. Correlation of model likelihoods (Vanilla, SFT, ProteinMPNN and ProteinDPO) or predicted values (ThermoMPNN) with experimentally measured thermal melting points of clinical and naturally derived mAbs curated from Shehata et al.^33^ and Jain et al.^34^. **d**, We evaluate the self-consistency and confidence of Paired model ProteinDPO designs for PDB structures 1STN, 1PHP and 4P79, comparing with experimental^6^ and ESMFold predicted^1^ structures. We show the experimental structure of the native sequence in green, the predicted structure of the native sequence in gray and the predicted structure of the ProteinDPO design in blue. Self-consistency TM (scTM) is the structural similarity score (TM) of the native sequence predicted structure ProteinDPO sequence predicted structure; pLDDT is the average ESMFold^1^ confidence (predicted error) for the structure of the generated sequence. Similarity is the percent of shared residue identity between the generation and native sequence. Average in silico free energy of protein folding (Δ*G*) for sequences from ProteinDPO, vanilla ESM-IF1 and SFT ESM-IF1 in REU compared with the native sequence derived from Rosetta *cart_ddg* protocol^38^ with five replicate runs for each sequence. 500 samples were taken from each model across five sampling temperatures within [0.01, 1] with no additional filtering. Lower energy corresponds to higher stability.

Motivated by previous work in which ESM-IF1 guided antibody affinity maturation by ranking sequence likelihoods conditioned on the respective antibody–antigen complex backbone^10^, we were interested if our method, optimized for stability, could generalize to binding affinity given that they are both determined by similar entropic and enthalpic interactions. To this end, we evaluate on two datasets, SKEMPIv2^28^ and AB-Bind^29^, which consist of experimentally measured binding affinities for thousands of sequence variants across hundreds of protein–protein complexes, with AB-Bind containing exclusively antibody–antigen complexes (Fig. 3a). Due to SKEMPIv2 containing very few variants per complex (as low as one), we report the correlation of all model likelihoods with all affinity measurements, while for AB-Bind we report correlations averaged across each complex. Surprisingly, despite only training on the ΔΔ*G* of small protein monomers, ProteinDPO has modestly increased scoring of the binding affinity of large protein–protein complexes (Pearson *R* increase of 0.02–0.04, Spearman *ρ* increase of 0.02–0.04 and AUROC increase of 0.04–0.05) and larger performance gains when scoring antibody–antigen complexes (Pearson *R* increase of 0.04–0.08, Spearman *ρ* increase of 0.03–0.10, AUROC increase of 0–0.02) (Fig. 3b). Similar to previous results (Fig. 2), the SFT model fails to generalize to binding-related tasks.

Encouraged by our finding that using whole-sequence likelihoods with our method improves upon addition of single-mutation likelihoods for scoring the relative stability (ΔΔ*G*) of double-mutation variants in Megascale, we sought to evaluate if ProteinDPO can use whole-sequence reasoning to rank absolute stability of sequences within a given fold, particularly antibodies. Antibodies are relevant therapeutic proteins for a wide array of diseases, for which high thermal stability is important for developability and storage^30–32^. To this end, we evaluate on a dataset of thermal melting points (*T*_m_) of 483 clinical and human-derived antibodies from Jain et al. and Shehata et al.^33,34^ (Fig. 3c). Despite only training on relative stability data of small monomers, ProteinDPO improves (Pearson *R* and Spearman *ρ* increases of 0.08–0.12) on the ability of vanilla ESM-IF1 in ranking the differences in thermal melting points (*T*_mid_) across diverse clinical and human-derived multichain antibodies. Notably, other supervised models trained on Megascale to predict ΔΔ*G* (such as ThermoMPNN) cannot evaluate proteins with multiple chains (such as most antibodies) or produce absolute stability metrics.

Beyond merely a supervised scoring function, ProteinDPO is also a generative model. We were therefore interested in testing if ProteinDPO would be useful for stabilizing sequences for given protein backbones, and whether it could do so more favorably than the original ESM-IF1 model. This is a complex task, as combining favorable single-mutations to design a sequence that is more stable than the native protein is non-trivial due to the nonlinear and higher order interactions among protein residues^24^. Here, we generate protein sequences for three diverse, native backbones (Fig. 3d): staphylococcal nuclease^35^ (PDB: 1STN), PGK^36^ (PDB: 1PHP), and the membrane protein claudin-15^37^ (PDB: 4P79). We computed the distribution of the average Rosetta force-field energy (Rosetta energy units, REU) across replicate runs for 500 generations for each model and native backbone, as well as the energy of each native sequence, all derived by modeling the mutated sequence onto the native backbone via the Rosetta cart_ddg protocol^38^. Although Rosetta energy can be inaccurate and is not a substitute for experimental testing, it is a widely used independent evaluator of machine learning generations due to its reliance on physics-based parameters rather than shared training data with machine learning models^39,40^.

Despite only training on the stabilities of small proteins (40–75 residues), sequences generated by ProteinDPO for much larger backbones (136–394 residues) are predicted to be substantially more stable (lower energy) than vanilla ESM-IF1, the SFT model and the native sequence. Surprisingly, despite having lower similarity to the native sequence than SFT and vanilla ESM-IF1, sequences generated by ProteinDPO have confident ESMFold-predicted structures (pLDDT >80) (Fig. 3d) that closely match the native structure (Extended Data Table 2). Although these sequences were not experimentally verified to express, this functions as a computational ‘sanity-check’ that the stability optimized ProteinDPO has not lost the rules of structure-conditioned sequence design learned from the unsupervised pretraining of ESM-IF1.

### Prefusion stabilization of H5 influenza HA

Protein stabilization has particularly critical applications in vaccine development, where rapid-response capabilities and thermal stability are essential for their global deployment^41^. For influenza vaccines, maintaining the prefusion conformation of HA helps elicit protective antibodies, as the native protein undergoes dramatic conformational changes during viral entry that can expose undesirable epitopes^20^. In addition, temperature stability is vital for vaccine storage and distribution, especially in resource-limited settings where cold-chain maintenance is challenging, and vaccines may be exposed to elevated temperatures^41^.

Traditional stabilization approaches rely on rational design or resource-intensive screens and, therefore, remain imperfect solutions. These manual changes to improve stability often leverage proline insertions, new disulfide bond formation and core stabilization through cavity filling^42^. However, these changes can often come at the cost of reduced protein expression, altered surface properties and disrupted native folding patterns when applied naively without considering the context of the full protein structure. Structure-conditioned language models, such as ProteinDPO, allow us to sample mutations that are likely to preserve the global protein structure—a key component for vaccine design, where preservation of antigenicity is critical.

**Fig. 4:**
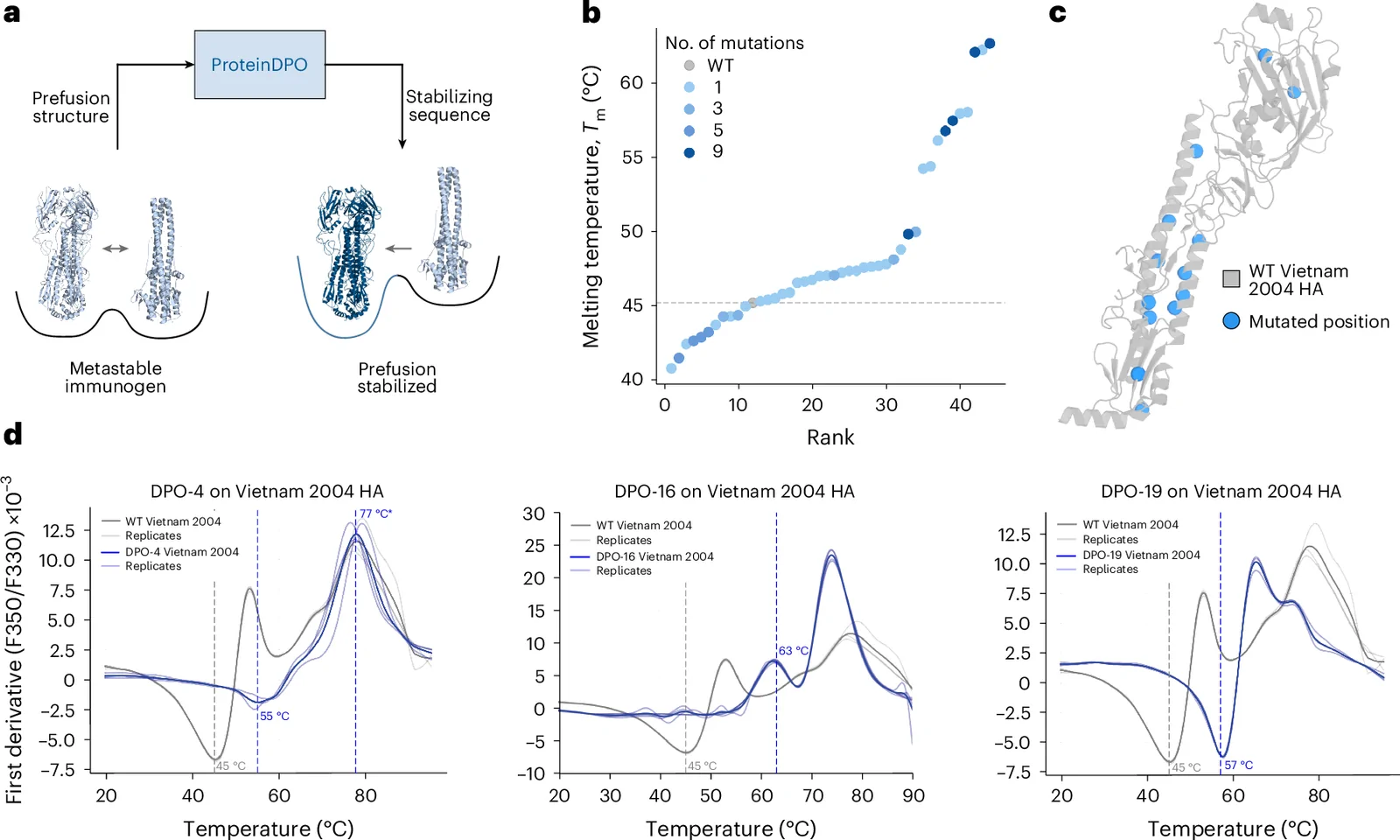
ProteinDPO enables rapid zero-shot stabilization of H5 influenza HA. **a**, Illustration of our prefusion stabilization campaign. Mutations are introduced by conditioning ProteinDPO with the prefusion structure of a fold-switching protein in order to stabilize this conformation and prevent a transition to the post-fusion state. **b**, Rank-ordered thermal stability of ProteinDPO variants applied to 2004 Vietnam H5N1 in melting temperature (*T*_m_) as determined by differential scanning fluorimetry (DSF) and colored by the number of mutations. **c**, Mutation positions of ProteinDPO variants plotted onto an experimental structure (PDB: 6CFG) of A/Vietnam/1203/04 (H5N1) (Vietnam 2004). **d**, The first derivative of the fluorescence ratio is plotted versus temperature as determined by DSF of three stabilizing nine-substitution variants: DPO-4, DPO-16 and DPO-19. Thermal melting events correspond to positive or negative peaks of the first-derivative curve and are labeled with gray (native) and blue (variant) dashed lines. *Although we report the first thermal melting event of DPO-4 as 56 °C, as determined by NanoTemper software, the dynamic range at this temperature is much lower than the first major melting event at 77 °C. WT, wild type. Source data

We therefore applied ProteinDPO to the task of stabilizing the prefusion state of influenza HA (Fig. 4a), a primary component of modern influenza vaccines and consequently an important target of protein stabilization efforts^20^. We specifically target the H5 subtype of HA, given the emergence of highly pathogenic H5N1 influenza A virus that has recently been found in mammalian populations, including sporadic human cases^19^. We use the structure from the HA of A/Vietnam/1203/04 (H5N1) (referred to as Vietnam 2004) as the starting point for our stabilization campaign (PDB: 6CFG), using ProteinDPO to rank all possible single-site substitutions based on their effects on ProteinDPO likelihood.

**Extended Data Fig. 2:**
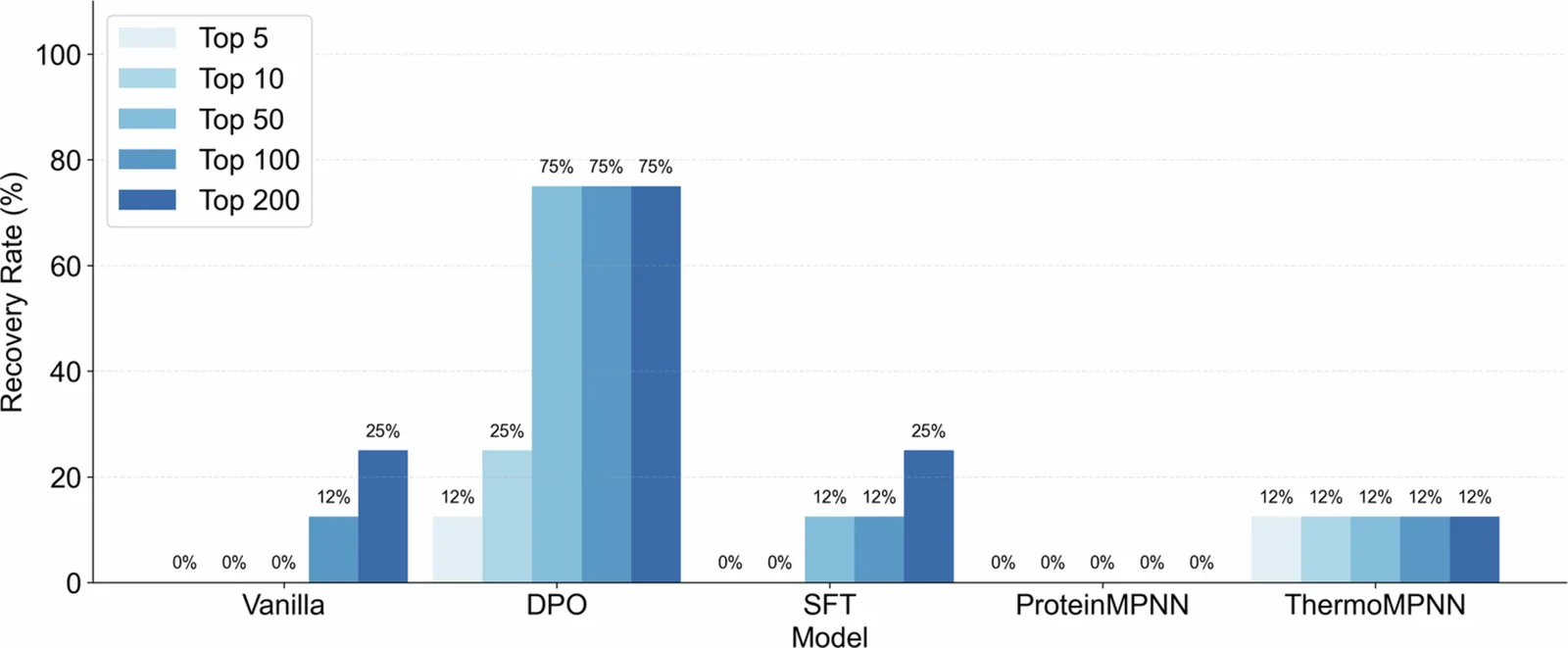
Recovery of literature-validated stabilizing mutations by design models. Recovery of literature-validated stabilizing mutations across protein design models. Bar plot showing the percentage of n=8 known stabilizing mutations (Milder et al. and Yassine et al.) recovered within the top-ranked predictions of each model. Models were evaluated using ensemble scoring across three PDB structures (6cf5, 6cfg, 2fk0).

Remarkably, despite only using the H5 HA structure from Vietnam 2004, ProteinDPO recovers known stabilizing mutations to H1 and H3 subtypes from the literature. Specifically, among the top-30 substitutions tested, six (H26W, K51M, K51I, E103I, E103L and N95L) exactly match the eight stabilizing mutations (BY94D and BH106R were not identified) reported in previous studies by Milder et al. and Yassine et al., which each report four mutations respectively^20,21^; an additional 5 of these top-30 single mutations occur at identical positions as the above 6 mutations. We then compared the ability of the Vanilla and SFT models, as well as ThermoMPNN^13^ and its predecessor ProteinMPNN^9^, to recover these eight stabilizing mutations from literature (Extended Data Fig. 2). In brief, this is evaluated by taking the percentage of stabilizing mutations that lie within the top-*n* single-mutations, upon scoring and ranking every possible single-mutation with the given model. While other models were able to recover one mutation, ProteinDPO has a higher recovery rate across all top-*n* levels. Most strikingly, ProteinDPO successfully identified critical residues (H26, K51 and E103) which make up the ‘pH-switch’ hypothesized by Milder et al. to modulate the pH-sensitive transition to the post-fusion state^20^. Importantly, the model was not trained on any data from HA and identified these residues in a zero-shot manner despite the >500 residue search space of HA. The remaining mutations primarily consist of cavity-filling substitutions in the helical core involving the introduction of leucine, tryptophan, tyrosine and phenylalanine residues that enhance hydrophobic core packing^43^ (Fig. 4c and Extended Data Table 3). In total, these results demonstrate that the model has learned to recognize fundamental structural principles that govern protein conformational stability.

**Extended Data Fig. 3:**
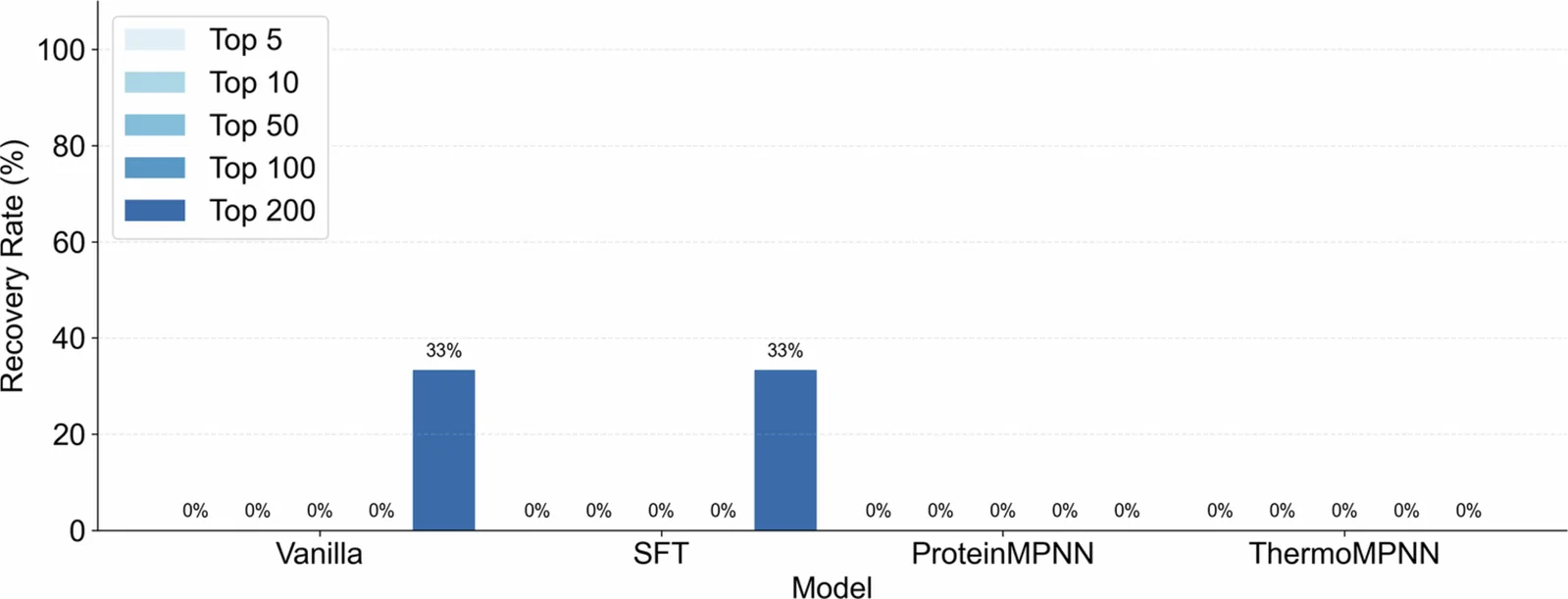
Recovery of highly stabilizing ProteinDPO mutations by design models. Recovery of highly-stabilizing ProteinDPO-identified single-substitutions across protein design models. Bar plot showing the percentage of n=6 significantly stabilizing (>= 9 C) DPO-identified mutations recovered within the top-ranked predictions of each model. Models were evaluated using ensemble scoring across three PDB structures (6cf5, 6cfg, 2fk0).

**Extended Data Fig. 4:**
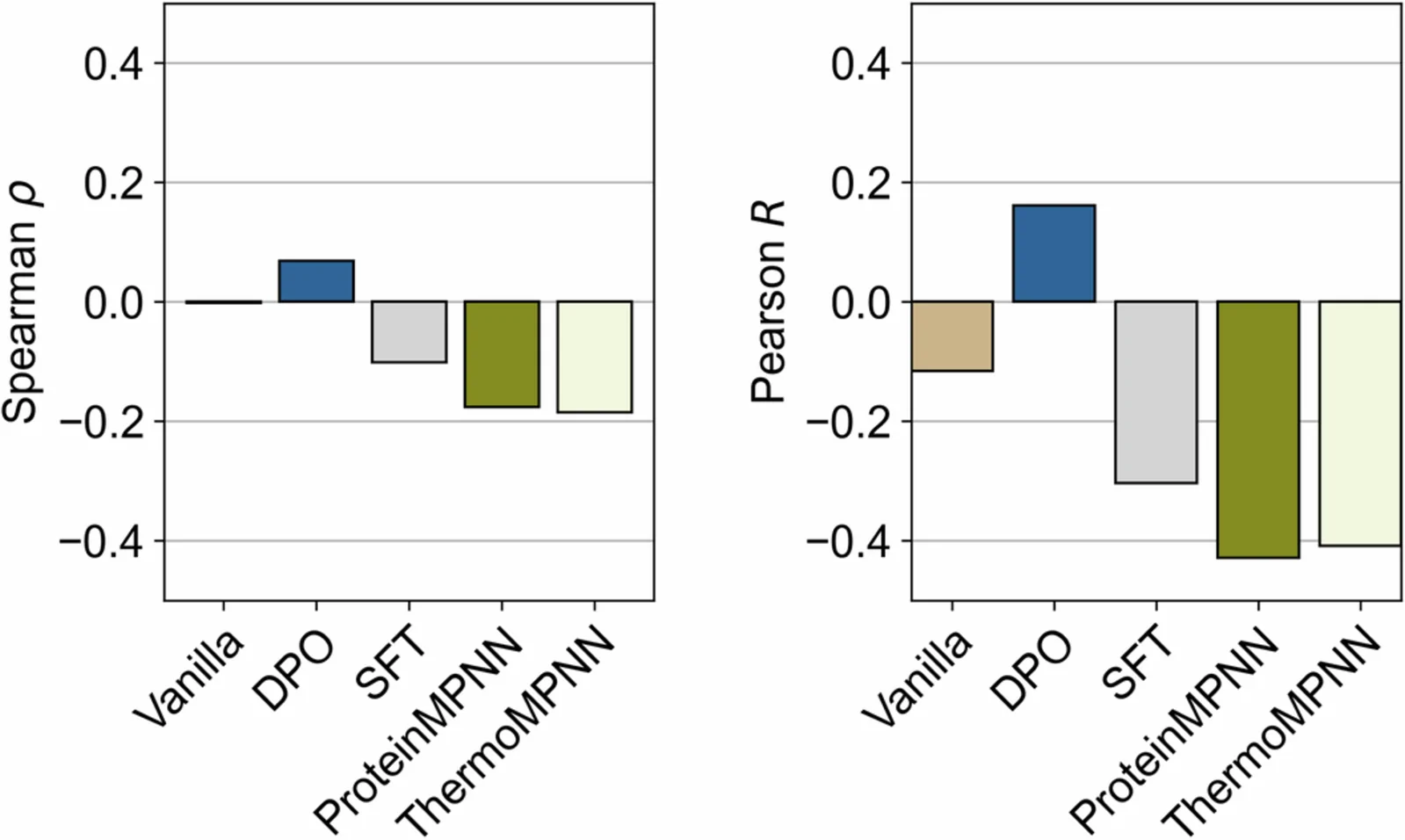
Ranking correlation between model scores and experimental melting temperatures. Comparison of methods in ability to rank the n=30 experimentally tested single-substitution variants identified by ProteinDPO, measured by Pearson R and Spearman rho correlation coefficients between model scores and temperature of thermal unfolding Tm.

**Extended Data Fig. 5:**
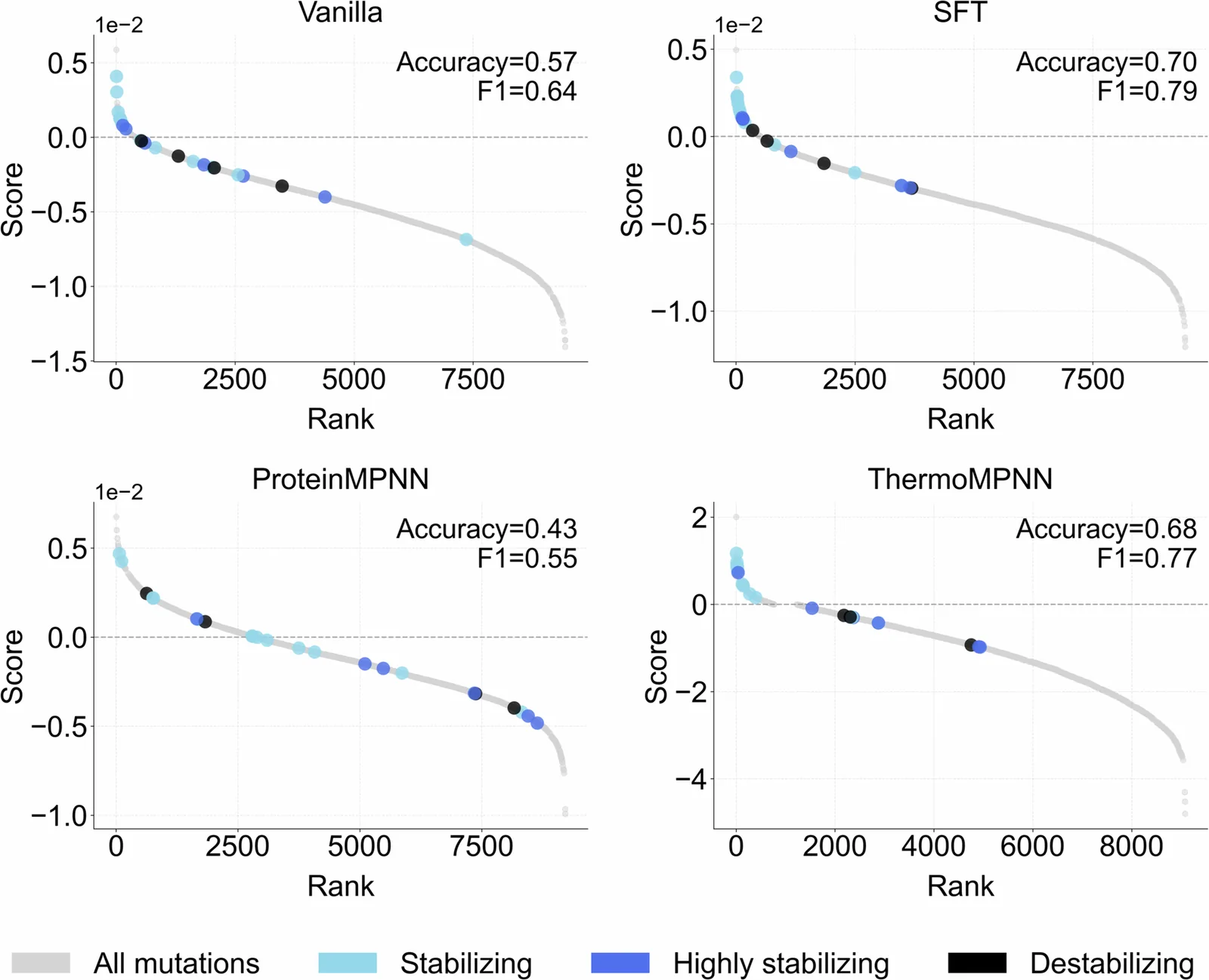
Score distribution placement of stabilizing versus destabilizing mutations. Method comparison in placement of stabilizing (light blue) and destabilizing (black) ProteinDPO-identified and experimentally-tested single-substitution variants within the score distributions of all possible mutations (gray). Mutations are subset to those which had temperature of thermal unfolding (Tm) that was at least 1 degree higher or lower than the native sequence n=23. Accuracy and F1 scores derived from the models’ prediction of a given mutation being favorable or unfavorable is also shown. Additionally, the position of the six significantly stabilizing mutations (dark blue) analyzed Figure S3 are shown.

**Extended Data Fig. 6:**
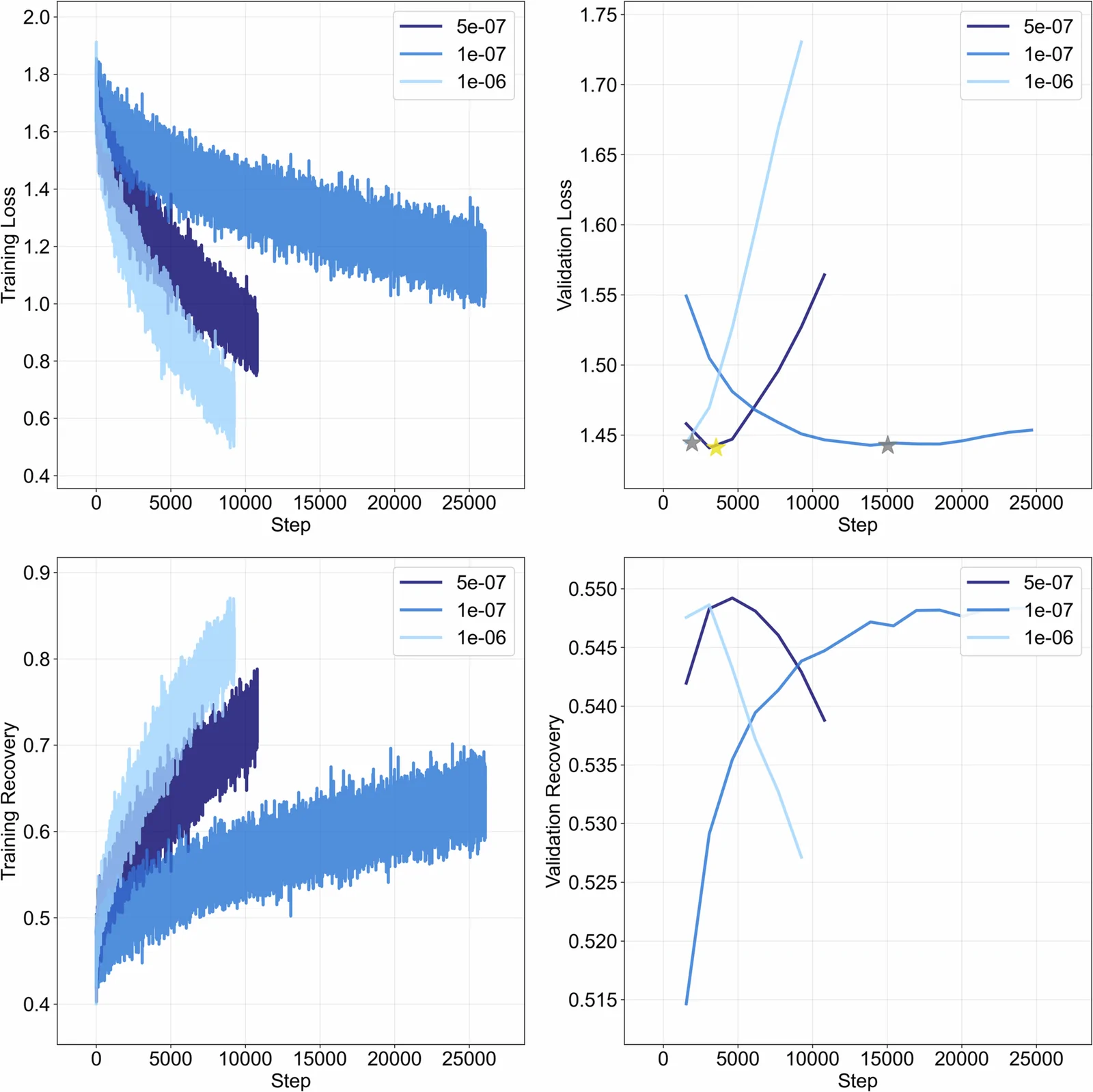
Training loss curves and sequence recovery during supervised fine-tuning. Training and validation loss and sequence recovery curves for training the supervised finetuned model. The selected checkpoint was the checkpoint which best minimized validation loss across all runs, and is denoted with a star. Checkpoints which minimized validation loss for other runs, are denoted with a gray star, and are compared against the selected checkpoint in Table S4.

We experimentally measured the thermal melting profiles with differential scanning fluorimetry on an initial round of the top-30 ProteinDPO-identified substitutions. Of these, 19 variants obtained greater thermal stability (*T*_m_) than the wild-type HA (>1 °C improvement), and 7 variants obtained similar melting temperature (<1 °C difference) (Fig. 4b). We then conducted a similar analysis as done for literature mutation recovery, but instead comparing models’ ability to identify 6 of the 30 ProteinDPO-identified single-substitution variants which were found to be significantly stabilizing (≥9 °C) (Extended Data Fig. 3). Only the SFT and Vanilla models were able to recover mutations in their top 200; although, it is possible other top-ranked mutants from these baseline models are also stabilizing. In addition, while all models are unable to rank the thermal stability (*T*_m_) of these 30 variants (Extended Data Fig. 4), the SFT model and ThermoMPNN show strong ability (F1 score >0.75) to classify them as stabilizing or destabilizing (Extended Data Fig. 5), despite crucially failing to capture most highly stabilizing mutations (Extended Data Fig. 3).

Beyond single-substitutions and aiming to discover synergistic effects among beneficial mutations, we used ProteinDPO to score all possible combinations of size three, five and nine (the total number of unique single-substitution positions was nine) mutations among non-deleterious mutations from the first round. For combinations of three and five, we additionally included a variant consisting of a simple combination of the top-n stabilizing variants. In line with in silico results suggesting the ability of ProteinDPO to generalize well to multiple mutations (Figs. 2c and 3c), all nine-substitution variants achieved greater thermal stability than the wild type, with the most successful variants achieving improvements of up to 17 °C (Fig. 4b). While the best nine-substitution variant did not significantly surpass the best single-substitution variant, nine-substitution variants were 9.8 °C more stable on average than single-substitution variants. All variants and their associated melting temperatures are reported in Extended Data Table 3. We show the thermal shift profiles of three variants containing nine substitutions (DPO-4, DPO-16 and DPO-19) demonstrating clear improvement in thermal stability from the wild type, with 10 °C (with a 32 °C change in the major thermal shift transition), 17 °C and 12 °C increases in *T*_m_, respectively (Fig. 4d).

**Fig. 5:**
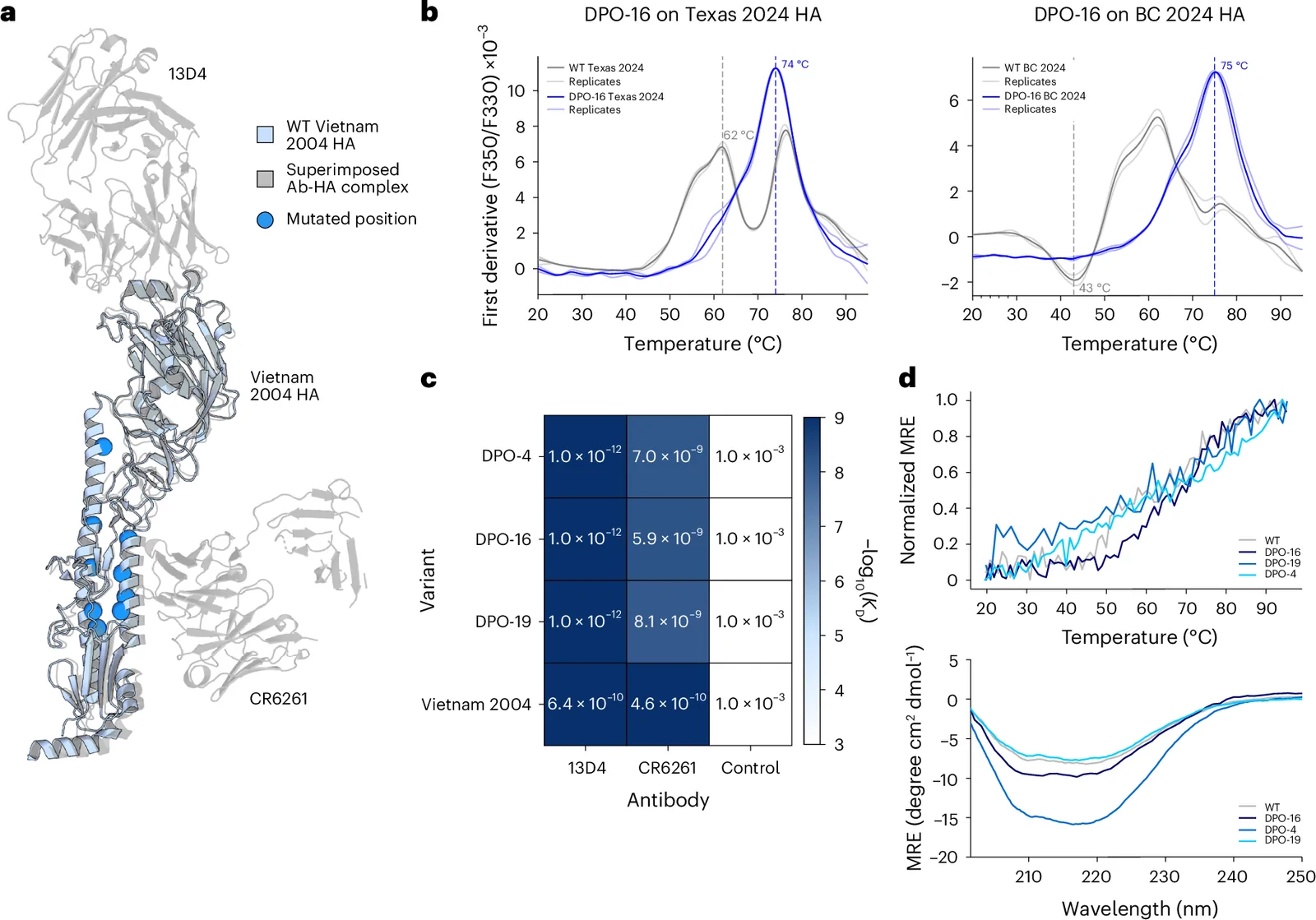
Variants generalize to recent variants of concern and retain native antigenicity. **a**, Structure of the Vietnam 2004 HA monomer (PDB: 6CFG, pale blue) along with mutation positions (bright blue) aligned and superimposed onto HA-bound structures of fragment antigen binding (Fab) domains of broadly neutralizing antibodies (bnAbs) CR6261 (PDB: 3GBN) and 13D4 (PDB: 6A0Z), which target the stem and head regions respectively. **b**, First derivative of the fluorescence ratio with respect to increasing temperature determined by differential scanning fluorimetry (DSF) of native Texas 2024 and British Columbia (BC) 2024 H5 HA as well as DPO-16-mutated variants. **c**, Heat map displaying antibody binding affinity in *K*_D_ of the native Vietnam 2004 H5N1 and ProteinDPO variants to bnAbs 13D4 and CR6261, with an anti-SARS-CoV-2 RBD antibody AZD3152 as a non-binding control. **d**, CD spectra showing mean residue ellipticity (MRE) at varying wavelengths of native Vietnam 2004 HA and ProteinDPO-identified variants at 20 °C (bottom) and a melting profile from 20 °C to 95 °C showing normalized MRE at 217 nm (top). Source data

To determine whether ProteinDPO-identified mutations maintain key epitopes recognized by existing antibodies, we measured the binding affinity of HA variants with nine substitutions to anti-HA broadly neutralizing antibodies (bnAbs) targeting either the head or the stem domains of HA via biolayer interferometry. Strikingly, despite several mutations being located within or adjacent to key antibody-binding epitopes (Fig. 5a), we observed strong nanomolar or subnanomolar affinities for all antibody–antigen pairs (Fig. 5c). In addition, circular dichroism (CD) spectra at 20 °C and a melting profile up to 95 °C confirmed that variants preserved the secondary structure character of the native HA (Fig. 5d). Differences in absorbance signal magnitude are due to concentration differences between samples (Methods).

Given the recent emergence of H5N1 variants with pandemic potential, we then introduced the same ProteinDPO-identified mutations to the A/Texas/dairycattle/Texas/24009367011/2024 variant (GISAID EPI_ISL_19094620; referred to as Texas 2024) derived from dairy cattle and the A/British_Columbia/PHL-2032/2024 (GISAID EPI_ISL_19548836; referred to as BC 2024) human variant, which are two distinct genotypes (B.3.13 and D1.1, respectively) of the H5N1 clade 2.3.4.4b virus that began circulating in 2024^44^. We again saw substantial improvements in *T*_m_ despite 20 years of viral evolution: DPO-16 achieves 13 °C and 32 °C improvements in *T*_m_ compared with native HA from the Texas and British Columbia strains, respectively (Fig. 5b). The ability to stabilize a desirable conformation of a protein, in this case the prefusion state of HA, while retaining native antigenicity and generalizing to evolved variants, are broadly useful components in the development of robust vaccine candidates that can withstand the rapid evolution characteristic of influenza viruses.

## Discussion

We have shown that DPO can effectively imbue an unsupervised structure-conditioned language model with biophysical information. The resulting model, ProteinDPO, outperforms the original pretrained model and a model further specialized via SFT in its ability to score mutations for stability and to generate stable protein sequences.

Strikingly, we see that the information learned from training ProteinDPO only on the relative changes in stability of small protein monomers generalizes to larger, unseen folds. This includes scoring the binding affinity of large protein–protein complexes, thermal melting points of multichain antibodies and single-step stabilization of proteins an order of magnitude larger than the size of training data examples. By contrast, SFT, which does improve scoring within the training data, achieves poor generalization to these tasks, suggesting that SFT merely overfits to the data in the training distribution. Moreover, in silico training of ProteinDPO on small, monomeric proteins has generalized toward an in vitro stabilization campaign of a large, trimeric, viral immunogen. In total, our results suggest that ProteinDPO has not ‘forgotten’ the general information learned during unsupervised pretraining on diverse proteins and instead learned generalizable rules of protein stability from its experimental biophysical alignment dataset.

The HA H5 stabilization campaign with ProteinDPO demonstrates the practical utility of this approach. We observed that 80% of ProteinDPO-identified variants result in either increased or maintained stability relative to the native protein. Testing only 45 total designs, we achieved up to 17 °C improvements in melting temperature (*T*_m_). Notably, heavily mutated variants retained binding affinity to neutralizing antibodies despite several mutations being located within or adjacent to the antibody binding epitope (Fig. 5a). When applied to genetically diverse H5N1 strains which emerged during the development of this work, a nine-substitution ProteinDPO variant (DPO-16) achieved improvements in *T*_m_ of 13 °C and 32 °C. This suggests that ProteinDPO stabilization occurs via fundamental structural principles rather than strain-specific modifications. This capability enables rapid development of broadly stable vaccine candidates that can withstand the antigenic evolution characteristic of influenza viruses, as demonstrated by mutations identified from a 2004 strain maintaining effectiveness across 20 years of viral evolution on strains from 2024.

Although the model is competitive with supervised, task-specific regression models such as ThermoMPNN^13^, it has not clearly surpassed all supervised models in scoring^25^ (Fig. 2b,d,e). However, using RL, unlike supervised regression, preserves essential generative capabilities. This enables ProteinDPO to both score sequences (such as regression models) and generate entirely new sequences aligned with desirable functions, which are capabilities that supervised models cannot combine. This generative capability further extends to the prediction of binding affinity and thermal melting across diverse wild-type proteins, multichain antibodies and protein complexes more accurately than the unaligned ESM-IF1 model (Fig. 3b–d) and the sampling of experimentally validated nine-substitution variants (Figs. 4 and 5). Furthermore, we find that alignment based on one specific property (such as the stability of small monomers) improves performance on these diverse tasks (such as binding affinity of large protein–protein complexes), most likely because both properties share common underlying physical principles.

Future directions of this work could explore whether alignment based on other properties could likewise enable generalizable information transfer. In addition, while our experimental efforts aimed to stabilize the prefusion state of HA, explicitly discouraging the postfusion state was not considered in variant generation but could be considered in future versions of our scoring procedure^45^. Moreover, while prefusion stabilization and retention of binding to native antibodies are key components of vaccine design, further evidence would be needed to demonstrate antigenic properties required for vaccine efficacy. Although ProteinDPO incorporates stability information into a structure-conditioned language model, this approach generalizes to any property, any generative model and any biological data modality. To this end, either for rapid protein stabilization or as a framework for alignment of biological generative models, we make ProteinDPO open-source and publicly available at https://github.com/evo-design/protein-dpo.

## Methods

### Generative model alignment

Generative AI applications are now routinely fine-tuned with feedback-based RL during post-training, which has been crucial for the success of applications in the natural language domain^49,50^. Here, we provide a brief overview of the classical model training pipeline, which consists of three stages: SFT, feedback collection and RL fine-tuning^51,52^.

#### SFT

Here, we define SFT as continuing the original pretraining objective on high-quality samples from a downstream labeled dataset. In this work for example, for SFT on the ‘Megascale’ dataset, high-quality samples are sequences which achieve superior stability to the native sequence. During the SFT stage we use a dataset of input-output pairs, ${\mathcal{D}}={\{{\bf{x}},{\bf{y}}\}}_{i = 1}^{N}$ and train a model *p*_*θ*_ with next-token maximum likelihood loss on high-quality pairs, where *y*_*i*_ is the *i*th token of the output **y**

1 $${{\mathscr{ \mathcal L }}}_{{p}_{\theta }}=-{{\mathbb{E}}}_{{\bf{x}},{\bf{y}}\sim{\mathscr{ \mathcal D }}}\left[\sum _{i=1}^{| {\bf{y}}| }\mathrm{log}{p}_{\theta }(\,{y}_{i}| {\bf{x}},{{\bf{y}}}_{ < i})\right].$$

#### Feedback stage

During the feedback stage we use the base model or the SFT model *p*_SFT_ and a set of inputs to sample a number *K* > 0 of candidate completions **y**^(*i*)^, …, **y**^(*K*)^ per prompt **x** from a dataset of preferences ${{\mathcal{D}}}_{{\rm{pref}}}$. Feedback can be provided in several ways:

1. Prior RLHF work has used ranking type of comparisons in which the candidate outputs are ranked ${{\bf{y}}}^{({i}_{1})}\succ {{\bf{y}}}^{({i}_{2})}\succ \ldots \succ {{\bf{y}}}^{({i}_{K})}$, with pairwise rankings being a popular choice. To use this data from the RL stage, we need to extract an evaluation score from it. We usually assume a parameterization of the preference distribution, such as the Plackett-Luce^53,54^ choice model (or the Bradley–Terry^55^ for pairwise data). In the most general case, given the permutation $\tau :{{\bf{y}}}^{(1)},\ldots ,{{\bf{y}}}^{(K)}\to {{\bf{y}}}^{({i}_{1})},\ldots ,{{\bf{y}}}^{({i}_{k})}$, we have
  2 $$P(\tau | {{\bf{y}}}^{(1)},\ldots ,{{\bf{y}}}^{(K)},{\bf{x}})=\prod _{k=1}^{K}\frac{\exp (r({\bf{x}},{{\bf{y}}}^{({i}_{k})}))}{{\sum }_{j=k}^{K}\exp (r({\bf{x}},{{\bf{y}}}^{({i}_{\!j})}))}.$$
  We can then form ulate this as a maximum likelihood loss over the reward model
  3 $${{{ \mathcal L }}}_{r}=-{{\mathbb{E}}}_{{\bf{x}},{{\bf{y}}}^{({i}_{1})},\ldots ,{{\bf{y}}}^{({i}_{K})}\sim {{{ \mathcal D }}}_{\mathrm{pref}}}\sum _{k=1}^{K}\left[r\left({\bf{x}},{{\bf{y}}}^{({i}_{k})}\right)-{\mathrm{log}}\sum _{j=k}^{K}\exp \left(r\left({\bf{x}},{{\bf{y}}}^{({i}_{\!j})}\right)\right)\right].$$
2. In the case of pairwise rankings **y**^*w*^ ≻ **y**^*l*^, this just reduces to the standard binary logistic regression loss,
  4 $${{{ \mathcal L }}}_{r}=-{{\mathbb{E}}}_{{\bf{x}},{{\bf{y}}}^{w},{{\bf{y}}}^{l}\sim {{{ \mathcal D }}}_{\mathrm{pref}}}\left[{\mathrm{log}}\sigma (r({\bf{x}},{{\bf{y}}}^{w})-r({\bf{x}},{{\bf{y}}}^{l}))\right],$$
  where *σ*(⋅) is the sigmoid function. Notice that if we are provided with a complete ranking over *K* candidates then we can construct $$\left(\begin{array}{c}K\\ 2\end{array}\right)$$ pairwise rankings, which was used in prior works^22^. These can still be easily optimized together since we still require a single forward pass through the reward model.
3. Alternatively a numerical score, *r*_*i*_ (such as used in a Likert scale) could be provided per candidate input-output pair (**x**, **y**^(*i*)^).

#### RL stage

During the RL stage we use a reference model *p*_ref_, sometimes initialized as *p*_SFT_ from the SFT stage, and optimize the following RL problem:

5 $${\mathop{\max }\limits_{{p}_{\theta }}}\,\underbrace{{{\mathbb{E}}}_{{\bf{x}} \sim {\mathcal{D}},{\bf{y}} \sim {p}_{\theta }({\bf{y}}| {\bf{x}})}[r({\bf{x}},{\bf{y}})]}_{{\rm{maximize}}\,{\rm{score}}\,{\rm{of}}\,{\rm{generations}}}-\underbrace{\beta {{\mathbb{D}}}_{{\rm{KL}}}[{p}_{\theta }({\bf{y}}| {\bf{x}})| | {p}_{{\rm{ref}}}({\bf{y}}| {\bf{x}})]}_{{\rm{distributional}}\,{\rm{penalty}}}.$$

We want to optimize a model *p*_*θ*_ to maximize the score, while also maintaining a distribution close to the reference model. The second terms aims to keep the model close to the distribution, which was used to collect the feedback data and prevent out-of-distribution issues^56^.

### Optimization algorithms

Two main algorithms have emerged for the optimization of the objective in equation (5): (1) classical RL algorithms, such as PPO^57^, which use on-policy rollouts from the model; and (2) direct alignment algorithms, such as DPO^16^, which train fully-offline using only feedback data. Although successful in large scale-systems, such as GPT-4, the PPO pipeline requires a very accurate score model that can be queried in a continuous manner. Prior work has directly trained a separate score network *r*_*θ*_(**x**, **y**) using the feedback data with the objective in equation (4) and directly applied PPO to the problem in equation (5) using the trained score model. However, such a pipeline can be quite complex, unstable and computationally expensive^58,59^. DPO^16^ is an alternative to this algorithmic pipeline, which we present below.

### DPO

The DPO algorithm is based on the observation that the objective in equation (5) has a closed form solution of the type

6 $${p}^{* }({\bf{y}}| {\bf{x}})=\frac{1}{Z({\bf{x}})}{p}_{\mathrm{ref}}({\bf{y}}| {\bf{x}}){{\rm{e}}}^{\frac{1}{\beta }r({\bf{x}},{\bf{y}})},$$

where

7 $$Z({\bf{x}})=\sum _{{\bf{y}}}{p}_{\mathrm{ref}}({\bf{y}}| {\bf{x}}){{\rm{e}}}^{\frac{1}{\beta }r({\bf{x}},{\bf{y}})}$$

is an intractable partition function. The key insight of the DPO algorithm is the model-reward equivalency, that is,

8 $$r({\bf{x}},{\bf{y}})=\beta \log \frac{{p}^{* }({\bf{y}}| {\bf{x}})}{{p}_{{\rm{ref}}}({\bf{y}}| {\bf{x}})}+\beta \log Z({\bf{x}}).$$

Now, if instead of training a score model *r*_*θ*_, we directly parameterize the generative model *p*_*θ*_, we can use the preference data to directly optimize *p*_*θ*_, essentially marginalizing the RL loop.

For the ranked objective, if we substitute the above expression into the Plackett–Luce reward model in equation (3), we obtain the following objective:

9 $$\begin{array}{l}{{\mathcal{L}}}_{{p}_{\theta }}=-{{\mathbb{E}}}_{{\bf{x}},{{\bf{y}}}^{({i}_{1})},\ldots ,{{\bf{y}}}^{({i}_{K})} \sim {{\mathcal{D}}}_{{\rm{pref}}}}\mathop{\sum }\limits_{k=1}^{K}\left[\beta \log \frac{{p}_{\theta }({{\bf{y}}}^{{i}_{k}}| {\bf{x}})}{{p}_{{\rm{ref}}}({{\bf{y}}}^{{i}_{k}}| {\bf{x}})}\right.\\\quad\,\,\,\,\left.-\log \mathop{\sum }\limits_{j=k}^{K}\exp \left(\beta \log \frac{{p}_{\theta }({{\bf{y}}}^{({i}_{j})}| {\bf{x}})}{{p}_{{\rm{ref}}}({{\bf{y}}}^{({i}_{j})}| {\bf{x}})}\right)\right].\end{array}$$

For the paired objective, If we instead use the pairwise objective in equation (4), we obtain the canonical form of the DPO objective

10 $${{\mathcal{L}}}_{r}=-{{\mathbb{E}}}_{{\bf{x}},{{\bf{y}}}^{w},{{\bf{y}}}^{l} \sim {{\mathcal{D}}}_{{\rm{pref}}}}\log \sigma \left(\beta \log \frac{{p}_{\theta }({{\bf{y}}}^{w}| {\bf{x}})}{{p}_{{\rm{ref}}}({{\bf{y}}}^{w}| {\bf{x}})}-\beta \log \frac{{p}_{\theta }({{\bf{y}}}^{l}| {\bf{x}})}{{p}_{{\rm{ref}}}({{\bf{y}}}^{l}| {\bf{x}})}\right).$$

Here, due to the homogeneity of both the Plackett–Luce and Bradley–Terry model (that is, comparing several options for the same context), the untractable partition term *Z*(**x**) cancels out and we are left with tractable, fully offline objectives.

### Weighted DPO algorithm

Prior methods, including DPO^16^, have all focused on extracting a per-sample score *r*(**x**, **y**) from rankings. However, if we have some external evaluation score, that is, data ${\{{\bf{x}},{{\bf{y}}}_{1},r({\bf{x}},{{\bf{y}}}_{1})\}}_{i=1}^{K}$, then this step is redundant. Given numerical scores, we could train a score model, using a regression-based framework and then use the standard PPO pipeline, but this approach inherits all the shortcomings of the standard RLHF pipeline. We could use the numerical scores to induce a ranking over candidates **y**_*i*_ and the apply the Plackett–Luce or Bradley–Terry DPO objectives in equations (9) and (10), respectively; however that faces two drawbacks:

1. Plackett–Luce and Bradley–Terry both assume a particular parametric form of the ranking likelihood, that is, the rankings are inherently probabilistic, which would induce additional noise in the optimization procedure if we generate rankings based on those choice models.
2. We can also generate rankings based on raw scores similarly to prior works^60^. However, note that any monotone transformation would still induce the same relative rankings. That is, there might be actual relative information in the score which could be lost in such a scenario.

Weighted regression-based methods^61–63^ are a good fit for this problem; however, they have been suboptimal in the large generative model setting and do not yield significant improvement^64^. Instead, we will aim to develop a DPO type of algorithm that can efficiently utilize the numerical scores of the candidate sequences. Given an **x** and candidate sequences ${\{{{\bf{y}}}^{(i)},r({\bf{x}},{{\bf{y}}}^{(i)})\}}_{i = 1}^{K}$, consider the parameterized reward

11 $${r}_{\theta }({\bf{x}},{{\bf{y}}}^{(i)})=\beta\,\mathrm{log}\frac{{p}_{\theta }({{\bf{y}}}^{(i)}| {\bf{x}})}{{p}_{\mathrm{ref}}({{\bf{y}}}^{(i)}| {\bf{x}})}+\beta \mathrm{log}{Z}_{\theta }({\bf{x}}).$$

That is a parameterized distribution *p*_*θ*(**y**∣**x**)_ induces a reward function *r*_*θ*_(**x**, **y**), such that the model *p*_*θ*_ is the optimal solution for the corresponding RL problem in equation (5). Now, instead of aligning the generative model *p*_*θ*_ with some sampled rankings or preferences, we will instead minimize a distance function of the type *D*(*r*(**x**, **y**), *r*_*θ*_(**x**, **y**)); that is, we will match the true score samples with the implicit ones. A remaining issue is that we still have the constraining partition function *Z*_*θ*_(**x**) (because it depends on *p*_*θ*_), which we would like to eliminate. When we consider

12 $${w}_{\theta }^{(i)}=\frac{\exp\left({r}_{\theta }\left({\bf{x}},{{\bf{y}}}^{(i)}\right)\right)}{{\sum }_{i}\exp\left({r}_{\theta }\left({\bf{x}},{{\bf{y}}}^{(i)}\right)\right)}=\frac{\exp\left(\beta \log \frac{{p}_{\theta }({{\bf{y}}}^{(i)}| {\bf{x}})}{{p}_{ref}({{\bf{y}}}^{(i)}| {\bf{x}})}\right)}{{\sum }_{i}\exp \left(\beta \log \frac{{p}_{\theta }({{\bf{y}}}^{(i)}| {\bf{x}})}{{p}_{ref}({{\bf{y}}}^{(i)}| {\bf{x}})}\right)},$$

notice that the partition function *Z*_*θ*_(**x**) was eliminated. In the same manner we have that

13 $${w}^{(i)}=\frac{\exp\left(r\left({\bf{x}},{{\bf{y}}}^{(i)}\right)\right)}{{\sum }_{i}\exp\left(r\left({\bf{x}},{{\bf{y}}}^{(i)}\right)\right)}.$$

Notice now that **w** = (*w*^(1)^, …, *w*^(*K*)^) and ${{\bf{w}}}_{\theta }=({w}_{\theta }^{(1)},\ldots ,{w}_{\theta }^{(K)})$ are both valid probability distributions. In fact, the probability distributions resemble the Boltzmann distribution from statistical mechanics^65^

14 $$p(i)=\frac{\exp\left(-\frac{{E}_{i}}{{k}_{B}T}\right)}{{\sum }_{i}\exp\left(-\frac{{E}_{i}}{{k}_{B}T}\right)},$$

where *p*(*i*) is the probability of the system being in a state *i* with energy *E*_*i*_, and *k*_B_*T* is the scaled absolute temperature of the system. Substituting *r*(**x**, **y**^(*i*)^) for the negative energy of the state −*E*_*i*_, and considering *k*_B_*T*, a parameter which allows us to scale the numerical scores *r*(**x**, **y**^(*i*)^), the weighted DPO objective is simply minimization of the Kullback–Leibler (KL) divergence ${{\mathbb{D}}}_{KL}[{\bf{w}}| {{\bf{w}}}_{\theta }]$ between the Boltzmann-like distributions parameterized by the generative model *p*_*θ*_ and numerical scores from the dataset ${\mathcal{D}}$. With some simple algebra, this yields:

15 $${{\mathscr{ \mathcal L }}}_{{p}_{\theta }}=-{{\mathbb{E}}}_{{\mathscr{ \mathcal D }}}\left[\sum _{i}{w}^{(i)}\beta \mathrm{log}\frac{{p}_{\theta }({{\bf{y}}}^{(i)}| {\bf{x}})}{{p}_{\mathrm{ref}}({{\bf{y}}}^{(i)}| {\bf{x}})}-\mathrm{log}\sum _{i}\exp \left(\beta \mathrm{log}\frac{{p}_{\theta }({{\bf{y}}}^{(i)}| {\bf{x}})}{{p}_{\mathrm{ref}}({{\bf{y}}}^{(i)}| {\bf{x}})}\right)\right].$$

In the case where we have only two candidate sequences **y**^(1)^ and **y**^(2)^, this can be simplified to

16 $${{\mathscr{ \mathcal L }}}_{{p}_{\theta }}=-{{\mathbb{E}}}_{{\mathscr{ \mathcal D }}}\left[{w}^{(i)}\mathrm{log}\sigma \left(\beta \mathrm{log}\frac{{p}_{\theta }({{\bf{y}}}^{(1)}| {\bf{x}})}{{p}_{\mathrm{ref}}({{\bf{y}}}^{(1)}| {\bf{x}})}-\beta \mathrm{log}\frac{{p}_{\theta }({{\bf{y}}}^{(2)}| {\bf{x}})}{{p}_{\mathrm{ref}}({{\bf{y}}}^{(2)}| {\bf{x}})}\right)+\right.$$

17 $$\left.{w}^{(2)}\mathrm{log}\sigma \left(\beta \mathrm{log}\frac{{p}_{\theta }({{\bf{y}}}^{(2)}| {\bf{x}})}{{p}_{\mathrm{ref}}({{\bf{y}}}^{(2)}| {\bf{x}})}-\beta \mathrm{log}\frac{{p}_{\theta }({{\bf{y}}}^{(1)}| {\bf{x}})}{{p}_{\mathrm{ref}}({{\bf{y}}}^{(1)}| {\bf{x}})}\right)\right].$$

That is, this is essentially a weighted version of the DPO algorithm. One interpretation is that this objective is the expected DPO objective under *p*(**y**^(1)^ ≻ **y**^(2)^∣**x**) = *w*^(1)^ and *p*(**y**^(2)^ ≻ **y**^(1)^∣**x**) = *w*^(2)^. These objectives are theoretically optimal in the offline regime as they are both mode-seeking and use a ‘negative gradient’ formulation^64^.

#### Implementation

**Algorithm 1** Compute sequence log-likelihood

 1: **Input:** sequence **y**, encoded structure **h**

 2: **Output:** log-likelihood $\log {p}_{\theta }({\bf{y}}| {\bf{h}})$

 3: // Get logits via teacher forcing

 4: **O** ← TransformerDecoder(**y**_[0: |**y**|−1]_, **h**) // Logits for next-token prediction

 5: // Convert logits to probabilities

 6: **P** ← Softmax(**O**) // Token probabilities over vocabulary

 7: // Compute log-likelihood as negative cross-entropy

 8: **l** ← − CrossEntropy(**P**, **y**_[1: |**y**|]_) // Per-position negative loss

 9: $\log {p}_{\theta }({\bf{y}}| {\bf{h}})\leftarrow {\sum }_{t}{\ell }_{t}$ // Sum over sequence positions

10: **return** $$\log {p}_{\theta }({\bf{y}}| {\bf{h}})$$

**Algorithm 2** DPO for structure-conditioned protein language model (pairwise)

 1: **Input:** structure ${\bf{X}}\in {{\mathbb{R}}}^{L\times 3}$, preferred sequence **y**_*w*_, dispreferred sequence **y**_*l*_, reference log-likelihoods $\log {p}_{{\rm{ref}}}({{\bf{y}}}_{w}| {\bf{X}})$ and $\log {p}_{{\rm{ref}}}({{\bf{y}}}_{l}| {\bf{X}})$, KL penalty coefficient *β*

 2: **Output:** DPO loss ${{\mathscr{ \mathcal L }}}_{\mathrm{DPO}}$

 3: // Encode structure once for both sequences

 4: **h** ← GVPEncoder(**X**)

 5: // Compute policy log-likelihoods for both sequences

 6: **O**_*w*_, **O**_*l*_ ← TransformerDecoder(**y**_*w*_, **h**), TransformerDecoder(**y**_*l*_, **h**) // Logits

 7: $$\mathrm{log}{p}_{\theta }({{\bf{y}}}_{w}| {\bf{X}}),\mathrm{log}{p}_{\theta }({{\bf{y}}}_{l}| {\bf{X}})\leftarrow \,\mathrm{ComputeLogLikelihood}\,({{\bf{y}}}_{w},{{\bf{O}}}_{w}),\,$$$$\mathrm{ComputeLogLikelihood}\,({{\bf{y}}}_{l},{{\bf{O}}}_{l})$$

 8: // Compute pairwise DPO loss

 9: ${r}_{\theta }\leftarrow \log {p}_{\theta }({{\bf{y}}}_{w}| {\bf{X}})-\log {p}_{\theta }({{\bf{y}}}_{l}| {\bf{X}})$ // Policy log-ratio

10: ${r}_{\rm{ref}} \leftarrow {\rm{log}} \,\,{p}_{\rm{ref}} ({\bf{y}}_{w} |{\bf{X}}) - {\rm{log}}\,{p}_{\rm{ref}}({\bf{y}}_{l}|{\bf{X}})$ // Reference log-ratio

11: $${{{ \mathcal L }}}_{\mathrm{DPO}}\leftarrow -\mathrm{log}\sigma (\beta ({r}_{\theta }-{r}_{\mathrm{ref}}))$$

12: **return** $${{\mathcal{L}}}_{{\rm{DPO}}}$$

**Algorithm 3** DPO for structure-conditioned protein language model (ranked)

 1: **Input:** structure ${\bf{X}}\in {{\mathbb{R}}}^{L\times 3}$, ranked sequences {**y**_1_, …, **y**_*K*_} where **y**_1_ ≻ **y**_2_ ≻ … ≻ **y**_*K*_, reference log-likelihoods ${\{\log {p}_{{\rm{ref}}}({{\bf{y}}}_{k}| {\bf{X}})\}}_{k = 1}^{K}$, KL penalty coefficient *β*

 2: **Output:** DPO loss ${{\mathcal{L}}}_{{\rm{DPO}}}$

 3: // Encode structure once for all sequences

 4: **h** ← GVPEncoder(**X**)

 5: // Compute policy log-likelihoods for all sequences

 6: **for** *k* = 1 to *K* do

 7:  ** O**_*k*_ ← TransformerDecoder(**y**_*k*_, **h**) // Logits

 8:  $$\log {p}_{\theta }({{\bf{y}}}_{k}| {\bf{X}})\leftarrow \,\mathrm{ComputeLogLikelihood}\,({{\bf{y}}}_{k},{{\bf{O}}}_{k})$$

 9: **end for**

10: // Plackett–Luce ranking loss

11: $${{\mathcal{L}}}_{{\rm{DPO}}}\leftarrow 0$$

12: **for** *k* = 1 to *K* − 1 **do**

13:  ${r}_{k}\leftarrow {\beta}\,({\mathrm{log}}\,{p}_{\theta } ({{\bf{y}}}_{k}| {\bf{X}})- \mathrm{log}\,{p}_{\mathrm{ref}} ({{\bf{y}}}_{k}| {\bf{X}}))$ // Reward for rank *k*

14:  $${s}_{k}\leftarrow \log \mathop{\sum }\nolimits_{j = k+1}^{K}\exp \left(\beta (\log {p}_{\theta }({{\bf{y}}}_{j}| {\bf{X}})-\log {p}_{{\rm{ref}}}({{\bf{y}}}_{j}| {\bf{X}}))\right)$$

15:  $${{\mathscr{ \mathcal L }}}_{\mathrm{DPO}}\leftarrow {{\mathscr{ \mathcal L }}}_{\mathrm{DPO}}-\mathrm{log}\sigma ({r}_{k}-{s}_{k})$$

16: **end for**

17: **return** $${{\mathcal{L}}}_{{\rm{DPO}}}$$

**Algorithm 4** DPO for structure-conditioned protein language model (weighted)

 1: **Input:** structure ${\bf{X}}\in {{\mathbb{R}}}^{L\times 3}$, sequences ${\{{{\bf{y}}}_{k}\}}_{k = 1}^{K}$, reference log-likelihoods ${\{\log {p}_{{\rm{ref}}}({{\bf{y}}}_{k}| {\bf{X}})\}}_{k = 1}^{K}$, preference targets ${\bf{w}}\in {{\mathbb{R}}}^{K}$, KL penalty coefficient *β*, temperature *T*

 2: **Output:** DPO loss ${{\mathcal{L}}}_{{\rm{DPO}}}$

 3: // Encode structure once for all sequences

 4: **h** ← GVPEncoder(**X**)

 5: // Compute policy log-likelihoods for all sequences

 6: **for** *k* = 1 to *K* **do**

 7:  ** O**_*k*_ ← TransformerDecoder(**y**_*k*_, **h**) // Logits

 8:  $$\log {p}_{\theta }({{\bf{y}}}_{k}| {\bf{X}})\leftarrow \,\mathrm{ComputeLogLikelihood}\,({{\bf{y}}}_{k},{{\bf{O}}}_{k})$$

 9: **end for**

10: // Compute reward-weighted cross-entropy loss

11: $${\bf{r}}\!\leftarrow\!\beta {\left[\log {p}_{\theta }({{\bf{y}}}_{1}| {\bf{X}})\!-\!\log {p}_{{\rm{ref}}}({{\bf{y}}}_{1}| {\bf{X}}),\!\ldots\! ,\log {p}_{\theta }({{\bf{y}}}_{K}| {\bf{X}})\!-\!\log {p}_{{\rm{ref}}}({{\bf{y}}}_{K}| {\bf{X}})\right]}^{\top }$$

12: ${{\mathcal{L}}}_{{\rm{DPO}}}\leftarrow -\mathop{\sum }\nolimits_{k = 1}^{K}{w}_{k}\log \frac{\exp ({r}_{k}/T)}{\mathop{\sum }\nolimits_{j = 1}^{K}\exp ({r}_{\!j}/T)}$ // Cross-entropy with temperature scaling

13: **return** $${{\mathcal{L}}}_{{\rm{DPO}}}$$

### Datasets

#### Megascale

The Megascale dataset (v2 4/20/2023) served as the experimentally measured stability dataset from which we aligned the pretrained ESM-IF1. After filtering out data marked as unreliable for machine learning by Megascale authors, the dataset contained approximately 660,000 variants for 405 protein domains. The variants consisted of insertions, deletions, single and double substitutions. Similar to prior methods^13^, insertions and deletions were not considered as DPO training was designed to compare stabilities across the same structure; however, due to the robust nature of the model we were able to train and model with the double-mutations in the dataset. We additionally removed duplicate sequences. FoldSeek^23^ clustering with the ‘easy-cluster algorithm was performed on all PDBs remaining in the Megascale dataset with an alignment threshold of 50%. From this, clusters were randomly assigned to respective train (90%), validation (5%) and test (5%) datasets to ensure generalizability of the model across different structures. Because the model is trained with sets of sequences for a common structure, either the Δ*G* (absolute) or ΔΔ*G* (relative) of sequences were used for training. While these should be identical in theory after accounting for native Δ*G*, due to noise in the data the two metrics are different. We found negligible difference in loss and validation accuracy training with either metric, so all models were trained on Δ*G* as more data points had Δ*G* measurements. For SFT, all variants with ΔΔ*G* < 0 were selected and the same splits were used. For using model likelihoods to predict Megascale thermostability, the whole-sequence log-likelihood of the variant (algorithm 1), normalized by subtracting the log-likelihood of the wild-type sequence, was used.

#### SKEMPIv2

SKEMPIv2 is database of binding free energy changes upon single point mutations within a variety of protein complex interfaces^28^. After removal of PDBs with non-canonical amino acids, the final dataset contained 6,487 variants. To score on this dataset, we followed instructions and code from the ESM-IF1 Github^5^, averaging the log-likelihood of simultaneous mutations given the entire complex structure and sequence as input. However we took the additional step of normalizing with the averaged wild-type likelihood of said mutated residues. Because certain complexes were only represented by very few variants, sometimes with a different PDB for each, we did not evaluate the average correlation within each complex but report an ‘all versus all’ comparison.

#### AB-Bind

AB-Bind is an antibody binding mutational database curated to train and evaluate computational affinity predictions from Sirin et al.^29^. The database includes 1,101 variants across 32 complexes curated from public data, each variant having experimentally determined changes in binding free energy (ΔΔ*G*). Duplicated variants, variants with missing mutated residues in the PDB, and those without an experimentally determined PDB were removed resulting in a final dataset size of 1,011 variants. We evaluated scoring on this dataset the same as with SKEMPIv2^28^; however, we reported the average correlations across each antibody–antigen complex as there were an ample number of variants per complex.

#### Antibody thermal unfolding

The antibody thermal stability dataset is derived from Widatalla et al.^32^, which compiled scores and IgFold^66^ predicted structures from the original Jain et al.^34^ and Shehata et al.^33^ datasets, consisting of thermal stability measurements for monoclonal antibodies (mAbs). These two studies collected several biophysical measurements for approximately 500 human B cell and LLPC derived mAbs^33^ and mAbs that have been in clinical trials^34^. Among these includes 483 measurements of the temperature of thermal unfolding (*T*_m_). While the entire fragment antigen binding (Fab) domain was measured in this experiment, only the structure and sequence of the variable domains (Fv) were taken as input to the models. We used no normalization for scoring on this dataset as we considered every sequence as a ‘native’ protein.

#### FireProt

The FireProt database is a curated dataset of changes in free energy (ΔΔ*G*) for 3,438 single mutations for 100 unique proteins^27^. To alleviate data-leakage from the Megascale training data, we used the Megascale ‘Homolog-free’ FireProt dataset from Diekhaus et al.^13^, which has all homologous sequences to the Megascale dataset removed, resulting in 2,578 variants. For this dataset, probably due to the presence of only single mutations, we found the best performance taking the log-likelihood of only the mutated residue, and normalizing by subtracting the wild-type residue likelihood.

#### S669

The S669 dataset contains experimentally measured ΔΔ*G* values of 669 single mutations of 94 proteins^26^. Due to the presence of non-canonical amino acids, four variants were removed from this dataset for evaluation of vanilla ESM-IF1 and ProteinDPO. Similar to FireProt^27^, we found the best performance taking the log likelihood of the mutated residue normalized by subtracting the wild-type residue likelihood. Results for all other methods on this dataset derived from Cuturello et al. and Diekhaus et al.^13,25^.

### Evaluation

#### Model sampling

The sampling evaluation conducted for (Fig. 3d) is described here. In this evaluation, 500 sequences were sampled with the Paired, Vanilla and SFT models using the backbones from PDBs 1STN, 1PHP as input. Sequences were generated from each model across five sampling temperatures [0.01, 0.05, 0.1, 0.5, 0.1] with standard autoregressive sampling and no additional filtering. Sampling was conducted using sampling code from the ESM-IF1 Github repository^5^, only differing the the use of model weights between experiments. The exact code used is made available at https://github.com/evo-design/protein-dpo/blob/main/sample.py

#### Sampling evaluation

Self-consistency template modeling (TM) was calculated between the ESMFold^1^ predicted structure of the designed sequence and the experimental PDB structure. pLDDT was computed as the average per-residue predicted error from the ESMFold prediction. Given the generation and original sequence have the same sequence length, sequence similarity is calculated as the number of the residue matches divided by the total number of positions.

#### Rosetta generation evaluation

The same exact sequences from the above sampling evaluation was then evaluated with the Rosetta cart_ddg protocol^38^. To run this protocol, code from the RosettaDDGPrediction repository was used www.github.com/ELELAB/RosettaDDGPrediction. Below is the command used to run the protocol for 1PHP generations:

rosetta_ddg_run

-p /path/to/relaxed/pdb

-cr /path/to/config_run/cartesian2020_ref2015.yaml

-cs /path/to/config_settings/nompi.yaml

-r /home/talal/rosetta.source.release-371/

-d /path/to/out/dir/

-l /path/to/mutations.txt

Where the mutations.txt file simply includes a comma-separated list of all differing positions from the native sequence, for example, A.N.2.G,A.K.3.Y,A.R.7.Y,A.V.9.I,A.R.12.S,A.F.17.L,A.C.18.L,A.R.19.F,A.F.22.L,

A.N.23.D,A.E.27.V,A.Q.28.D,A.A.30.R,A.T.32.L,A.D.34.P,A.T.35.S,A.R.38.L,

A.A.40.Q,A.K.53.R,A.L.56.I,A.A.57.I,A.H.59.Q,A.R.62.D,A.K.64.G,A.K.66.…

#### Rosetta benchmarking protocol

For benchmarking of REU in antibody thermal stability prediction in (Fig. 3c), all input structures were energy-minimized using the PyRosetta FastRelax protocol^67^. The protocol was run with both backbone and sidechain degrees of freedom enabled, while rigid-body movements were disabled. Minimization was performed using the LBFGS-Armijo nonmonotone minimizer with a maximum of 200 iterations. Energy evaluations were performed using the ref2015 scoring function. Total REU was used and was not normalized by length, as length-normalization decreased performance.

#### Scoring metrics

All correlation coefficients were calculated using the scipy python package, particularly the spearmanr and pearsonr functions. AUROC scores were calculated using the scikit-learn python package, particularly the roc_auc_score function. When a correlation is reported as, for example, average Spearman *ρ*, this means that the correlation is computed for all mutations within each protein domain, then averaged across all domains. Any scoring comparisons between variants of ESM IF1 (vanilla, paired, ranked, weighted and SFT) were made with the exact same code and configuration, with the only difference being model weights. The exact input into the correlation calculations varies per dataset due to the presence of single versus double and multimutations and is noted in the Datasets section. ProteinMPNN^9^ was used in the same manner as ESM-IF^5^ and ProteinDPO for scoring, also varying per dataset. To run ProteinMPNN, the score function in the ColabDesign implementation was used, accessible at https://github.com/sokrypton/ColabDesign.

### Model training

#### Input construction

Multiple methods for positive-negative pair selection from the scalar-labeled data were evaluated, including randomly selecting pairs then ordering them by scalar value, prioritizing pairs with small differences and prioritizing pairs with large differences. To have a tunable parameter that controls the distance between pairs for the two latter approaches, we implemented a ‘gap level’ heuristic, which is a parameter within [0,1]. A gap level of 1 corresponds to placing the largest gap possible in sequence ranking between pairs, whereas a gap level of 0 corresponds to the smallest possible gap. For any gap level choice, we ensure the gaps between sequences in terms of ranking is uniform across all pairs and that all sequences are used, except for a single excluded sequence in the case there is an odd number of sequences for a native protein. Although most choices of gap level had comparable performance, we found a gap level of 0.5 performed the best on validation data. Sets of sequences were randomly chosen for models trained with the ranked objective, and models trained with the weighted objective and set size (*K*) greater than two. For models trained with the weighted objective we experimented with various values temperature values (*T*) for scaling the scalar labels, finding 0.1 to be the most favorable.

#### DPO training details

Noise of 0.1 Å was applied to all PDB structures in line with the training of ESM-IF1^5^. For each set (ranked and weighted models) or pair (paired model) of sequence responses, the same native backbone was used as structural prompt, PDBs of which were derived from Tsuboyama et al.^18^. Hyperparameter tuning was performed with varying values for *β*, gap level (paired and weighted model), temperature *T* (weighted model), learning rate, *K* (weighted and ranked model) and batch size. For computational efficiency, reference model likelihoods were precomputed before training. Models were each trained on a single NVIDIA H100 GPU with 80 GB of memory. All three models (paired, ranked and weighted) share the same learning rate of 1 × 10^−7^, *β* of 0.1, maximum of 30 epochs and identical Adam optimizer settings (*β*_1_ = 0.9, *β*_2_ = 0.98, *ϵ* = 1 × 10^−8^ and decay of 0.1). The paired and weighted models used a batch size of 32 and *K* = 2, while the ranked model used a batch size of 8 and *K* = 3. A gap level of 0.5 was applied to both the paired and weighted models, and the weighted model additionally used a temperature parameter *T* = 0.1. All models were trained until validation loss convergence, up to the maximum number of epochs. Algorithms for training ESM-IF1 with all variants of DPO are reported above (algorithms 2–4).

#### SFT training details

SFT training largely mirrored DPO training with two distinctions. Fine-tuning used the same noise level, the same input PDBs, was initialized from the same weights and used the same optimizer. The major distinction is fine-tuning was only done on a subset of training and validation examples which had favorable stability, specifically variants with stability superior to the native protein (ΔΔ*G* < 0) were selected. The second major distinction is loss. For SFT negative log-likelihood (or cross-entropy loss) was minimized. The algorithm for SFT is provided below (algorithm 5). Training and validation loss and sequence recovery curves across a learning rate sweep are provided in Supplementary Information figure 11 as well as performance on a subset of fitness evaluations (Fig. 4). The checkpoint with the lowest validation loss was selected.

**Algorithm 5** SFT of ESM-IF model

 1: **Input:** dataset ${\mathcal{D}}$, pretrained model *θ*_0_

 2: **Output:** fine-tuned model parameters *θ*

 3: // Filter for favorable stability

 4: ${{{ \mathcal D }}}_{\mathrm{stable}}\leftarrow \{({\bf{X}},{\bf{y}},{\Delta }{\Delta }G)\in {{ \mathcal D }}:{\Delta }{\Delta }G < 0\}$ // Select variants more stable than native

 5: Initialize *θ* ← *θ*_0_

 6: **for all** $({\bf{X}},{\bf{y}},\Delta \Delta G)\in {{\mathcal{D}}}_{{\rm{stable}}}$ **do**

 7:  // Encode structure

 8:  **h** ← GVPEncoder(**X**)

 9:  // Get decoder outputs (logits) via teacher forcing

10:  **O** ← TransformerDecoder(**y**_[0: |**y**|−1]_, **h**)

11:  // Compute negative log-likelihood (see Algorithm 1)

12:  ${\mathcal{L}}\leftarrow -\,\mathrm{ComputeLogLikelihood}\,({\bf{y}},{\bf{O}})$ // Cross-entropy loss

13:  UpdateParameters$(\theta ,{\nabla }_{\theta }{\mathcal{L}})$ // One optimization step

14: **end for**

15: **return** *θ*

### In vitro experiments

#### Variant selection

To select variants for experimental testing, the sequence likelihood of every possible single-site substitution was evaluated using ProteinDPO using an experimental structure of H5N1 HA (PDB: 6CFG). This was done by individually substituting all 19 alternate amino acids across every position in the native sequence and scoring the whole-sequence likelihood of each variant using the algorithm defined above 1. Given HA is a homo-trimer, scores for identical chains were averaged. To alleviate structural bias due to experimental conditions during structural characterization of the input structure, this procedure was repeated across two other available structures of H5N1 hemagluttinin (PDBs: 2FK0, 6CF5), and the percentile ranking of mutants within each structure were averaged. The top-*n* variants in highest percentile rank averaged across structures were selected for characterization. We note that the first 20 single-site substitutions experimentally characterized (denoted as DPO-A1-20 in Extended Data Table 3) were selected using the monomeric form of HA, only utilizing PDB 6CFG, while the following 10 single-site substitutions (Extended Data Table 3, DPO-1,2,5,6,8,11,14,17,20,23), as well as all following multisite substitution variants were selected using the trimeric form of the PDB and all three experimental structures.

To select three-site, five-site and nine-site substitution variants in reasonable computational time, we utilized the top single-site substitutions predicted by the model given the trimeric structure as an initial pool to derive combinations from, removing six single-substitutions that were not significantly stabilizing (P160D, P160E, H26W, H26Y, G47W, H111F) in the first round of monomeric-structure-derived variants (Extended Data Table 3, DPO-A1-20). In addition, we excluded chain A of hemagluttinin (head region) as defined by PDB 6CFG from the pool of single-site substitution variants, given ProteinDPO heavily preferred variants in chain B (stem region). Finally, we trimmed down this pool to balance the benefits of coverage with the computational costs of exploring greater combinatorial space. This resulted in an initial pool of 16, 13 and 57 unique single-substitutions to be used as candidates for the three-site, five-site and nine-site substitution variants respectively. We then used the same procedure for scoring single-site substitutions, whole-sequence likelihood, for every possible combination of mutations from the pool for three-site, five-site and nine-site substitution variants. We once again repeated this across all three structures, calculated the ranking of each variant within each structure, and selected the best variants by average percentile rank across the structures for experimental testing. For combinations of three and five, we additionally included a variant consisting of a simple combination of the top-*n* stabilizing single-site substitution variants. This was not possible for combinations of nine, given some of these positions (for example, N95) had not been tested in the initial round of single variants (Extended Data Table 3, DPO-A1-20).

#### HA site-directed mutagenesis

The HA sequence was cloned into a pADD2 vector between the rBeta-globin intron and *β*-globin poly(A). HA constructs contain a Foldon trimerization domain, an AviTag and a C-terminal 6× His tag. We used H5N1 HA sequences from the A/Vietnam/1203/2004 for the initial experiments. We used the A/Texas/dairycattle/Texas/24009367011/2024 and A/British_Columbia/PHL-2032/2024 variants for the follow-up experiments, whose amino acid sequences were derived from GISAID^44^. Single amino acid mutations were introduced using mutagenic primers and Gibson assembly. The target plasmid was linearized by restriction digest outside the region of interest. PCR fragments were amplified from the wild-type plasmid using Q5 High-Fidelity DNA Polymerase (NEB) with mutagenic primers containing the desired mutations and Gibson-compatible overlaps. PCR products were gel-purified and assembled with the digested vector using Gibson Assembly Master Mix (NEB). Assembled products were transformed into *Escherichia coli* Stellar competent cells (Takara Bio, 636763) and plated on carbenicillin selection plates. Positive clones were verified by whole-plasmid sequencing before plasmid preparation.

#### Antibody cloning

Variable regions of antibody heavy and light chain sequences were ordered as eBlock gene fragments from Integrated DNA Technologies and cloned into a linearized CMV/R plasmid backbone by Gibson assembly, for expression under a constitutive CMV promoter as described^10^. Plasmid backbones possessed human IgG1 heavy or light chain constant domains; 13D4^68^ was cloned with a kappa light chain, whereas CR6261^69^ and AZD3152^70^ were cloned with lambda light chains. Primers used for destination plasmid linearization were also synthesized by Integrated DNA Technologies.

#### DNA preparation

Plasmids were transformed into Stellar competent cells, and transformed cells were plated and grown at 37 °C overnight. Colonies were midi-prepped per the manufacturer’s protocols (ZymoPure II Plasmid Midiprep Kit, Zymo Research). Plasmids were sterile filtered using a 0.22-μm syringe filter and stored at 4 °C.

#### Protein expression

All proteins were expressed in Expi293F cells (Thermo Fisher Scientific, A14527). For variants which where to be further tested for antibody binding, a biotinylation tag (AviTag) were also expressed in the presence of a BirA enzyme, resulting in spontaneous biotinylation during protein expression^10^. Expi293F cells were cultured in media containing 66% FreeStyle 33% Expi media (Thermo Fisher Scientific) and grown in polycarbonate shaking flasks at 37 °C in 8% carbon dioxide. Upon sufficient growth, cells were then diluted and transfected at a density of approximately 3–4 million cells per milliliter and volume of 45 ml. Transfection mixtures were made by adding the following components: 15 μg of midi-prepped DNA, 3 ml of culture media, 300 μl of glucose, 300 μl of valproic acid and FectoPRO (Polyplus) would be added to cells following manufacturer protocols. After mixing and a 10-min incubation, the transfection cocktail would be added to the 45 ml of cells. The cells were collected 3–5 days after transfection by spinning the cultures at 10,000*g* for 20 min. Supernatants were filtered using a 0.45 μm filter.

#### Protein purification

All variants of HA were His-tagged and purified using HisPur Cobalt resin (Thermo Fisher Scientific, 89964)^10^. A total of 1 ml of of resin was added to cell supernatants, and the samples were then incubated with rotation at 4 °C for at least 1 h. Resin/supernatant mixtures were added to chromatography columns for gravity flow purification. The resin in the column was washed with wash buffer (20 mM imidazole, phosphate buffered saline (PBS) pH 7.4, 150 mM NaCl). The proteins were then eluted with 250 mM imidazole, PBS 7.4, 150 mM NaCl. This was followed by size-exclusion chromatography on an ÄKTA pure system (Cytiva). ÄKTA pure FPLC with a Superdex 200 Increase (S200) gel filtration column was used for purification, for which 0.5 ml of sample was injected and run over the S200, which had been pre-equilibrated in degassed PBS and flash-frozen before storage at −80 °C.

#### Antibody expression

Antibody expression was similar to protein expression, except cells were transfected with a 1:2 mixture of heavy chain DNA (7.5 μg):light chain DNA (15 μg) in a total expression volume of 30 ml.

#### Antibody purification

We purified antibodies using a 5-ml HiTrap MabSelect PrismA column on the ÄKTA pure fast protein liquid chromatography (FPLC) instrument (Cytiva). The ÄKTA system was pre-equilibrated with PBS pH 7.4, before the filtered supernatant from Expi293 expression culture was applied to the column directly from a 50 ml Superloop at a flow rate of 2.5 ml min^−1^. The column was washed with at least five column volumes (CV) of PBS pH 7.4, after which bound IgG was eluted with up to 20 CV 0.1 M sodium citrate pH 3.0. Samples were eluted with into collection tubes containing 1 M Tris–HCl, pH 9.0 alkaline buffer for immediate neutralization of the antibody samples. Between runs, the column was equilibrated with 7 CV PBS pH 7.4, washed with 2 CV 2.5 M NaCl and re-equilibrated into PBS pH 7.4. Using 30-kDa molecular weight cutoff centrifugal concentrators, the eluted samples were concentrated and either used directly in experiments or flash-frozen and stored at −80 °C.

#### Biolayer interferometry binding experiments

All reactions were run on an Octet RED96 at 30 °C, and samples were run in 1× PBS with 0.1% BSA and 0.05% Tween 20 (Octet buffer)^10^. IgGs were assessed for binding to biotinylated antigens using streptavidin biosensors (Sartorius/ForteBio). Antigen was loaded at a concentration of 200 nM. Tips were then washed and baselined in wells containing only Octet buffer. Samples were then associated in wells containing IgG at 100 nM concentration. Tips without loaded antigen were also associated in wells containing 100 nM IgG and used as a baseline subtraction for data analysis. Association and dissociation binding curves were fit in Octet System Data Analysis Software version 13.0.3.52 using a 1:2 bivalent model for IgGs to determine apparent *K*_D_. The broadly neutralizing antibodies (bnAbs) targeting the head and stem region of HA were 13D4^68^ and CR6261^69^, respectively. A control antibody used to assess non-specific binding was the anti-SARS-CoV-2 RBD antibody AZD3152^70^.

#### Thermal melting experiments

Thermal melting profiles of HA variants were measured by differential scanning fluorimetry on a Prometheus NT.48 instrument (NanoTemper). A total of 30 μl of each protein was concentrated or dilluted to at least 0.2 mg ml^−1^ in PBS, and triplicate samples were loaded onto Monolith Premium Capillaries (NanoTemper) and then subjected to a temperature gradient from 20 °C to 95 °C at a heating rate of 1 °C min^−1^. Intrinsic fluorescence (350 nm and 330 nm) was recorded as a function of temperature. Thermal melting curves were plotted using the first derivative of the ratio (350 nm/330 nm). Melting temperatures were calculated automatically by the instrument (PR.ThermControl software, version 2.3.1) and represented positive or negative peaks in the thermal melting curves.

#### CD experiments

CD spectra were obtained for selected HA variants with a JASCO CD spectrophotometer in a 1-mm-pathlength cuvette (Hellma). Protein samples were diluted or concentrated to be within the range of 0.1 mg ml^−1^ to 0.3 mg ml^−1^ in PBS at 20 °C. Concentration was calculated using a Thermo Scientific NanoDrop OneC Microvolume Spectrophotometer measuring for absorbance at 280 nm. For obtaining a melting profile, absorption signal was monitored at 217 nm from 20 °C to 95 °C in 1 °C increments per minute, with 10 s of equilibration time and 1 s of digital integration time.

### Reporting summary

Further information on research design is available in the Nature Portfolio Reporting Summary linked to this article.

## Online content

Any methods, additional references, Nature Portfolio reporting summaries, source data, extended data, supplementary information, acknowledgements, peer review information; details of author contributions and competing interests; and statements of data and code availability are available at https://doi.org/10.1038/s41592-026-03137-3.

## Supplementary information

Reporting Summary

Peer Review File

Supplementary Code 1 Training code for ProteinDPO.

Supplementary Data 2 Primers for single-mutant HA variants.

Supplementary Data 3 DNA sequences of HA strains.

Supplementary Data 4 DNA sequences of antibodies.

Supplementary Data 5 Gene fragments of multimutant designs which were inserted into H5 Vietnam plasmid.

Supplementary Data 6 Primers for cloning antibodies and BC and Texas H5s.

## Source data

Source Data Fig. 4b Thermal melting values for all variants collected from software.

Source Data Fig. 4d Thermal melting curve (first derivative) for variant DPO-16 compared with wild-type value.

Source Data Fig. 4d Thermal melting curve (first derivative) for variant DPO-19 compared with wild-type value.

Source Data Fig. 4d Thermal melting curve (first derivative) for variant DPO-4 compared with wild-type value.

Source Data Fig. 5b Thermal melting curve (first derivative) for variant DPO-16 on BC strain compared with wild-type value.

Source Data Fig. 5b Thermal melting curve (first derivative) for variant DPO-16 on Texas strain compared with wild-type value.

Source Data Fig. 5c BLI-collected binding affinity data.

Source Data Fig. 5d CD spectra at for variant DPO-4.

Source Data Fig. 5d CD spectra at for variant DPO-16.

Source Data Fig. 5d CD spectra at for variant DPO-19.

Source Data Fig. 5d CD Sspectra for wild-type HA.

Source Data Fig. 5d CD melt at 217nm from 20° to 95° for wild-type HA.

Source Data Fig. 5d CD melt at 217nm from 20° to 95° for DPO-19.

Source Data Fig. 5d CD melt at 217nm from 20° to 95° for DPO-16.

Source Data Fig. 5d CD melt at 217nm from 20° to 95° for DPO-4.

## Figures and Tables

**Extended Data Fig. 1:**
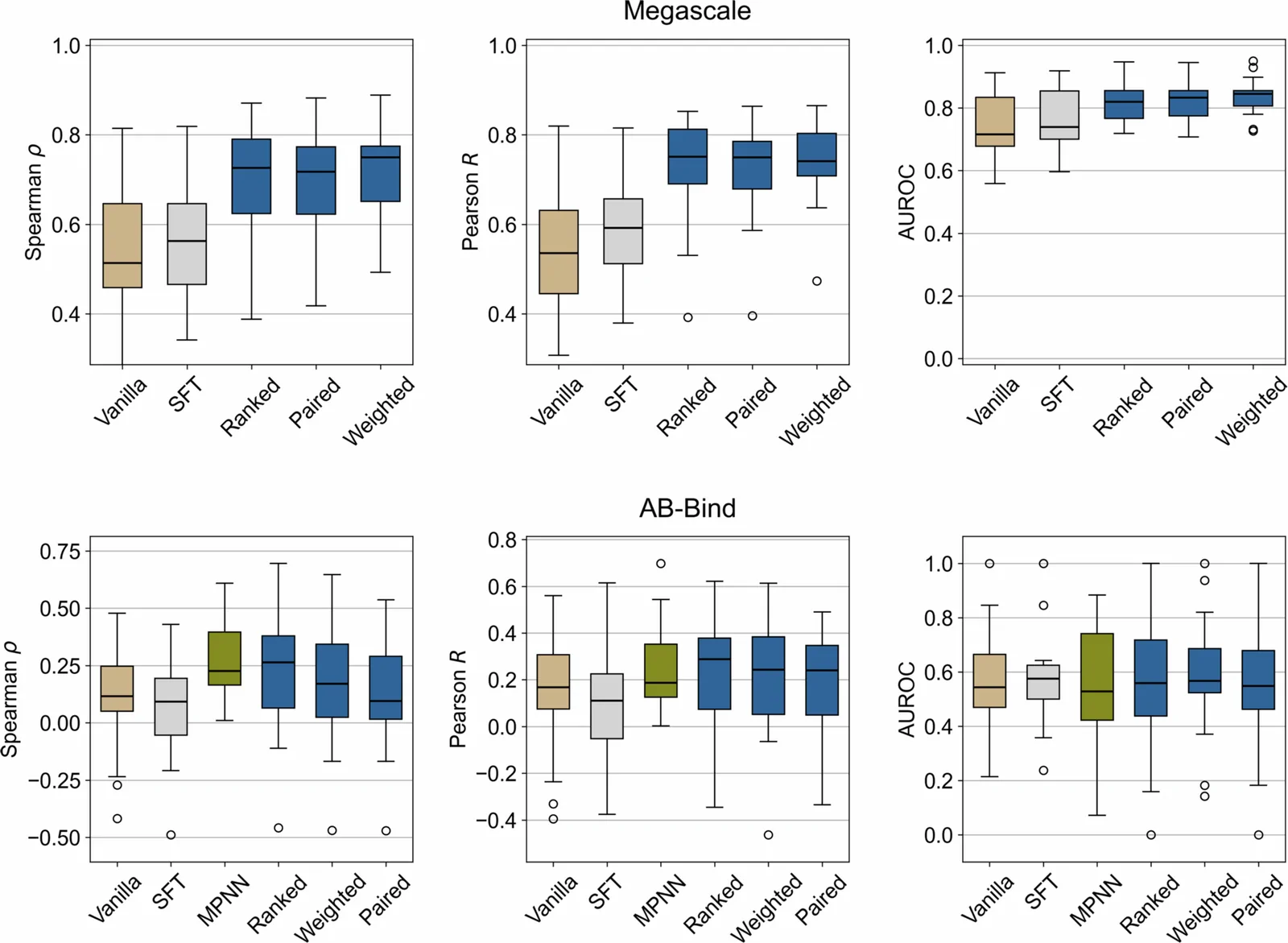
Performance distribution across protein domains for stability and binding prediction. Distribution of performance across varying protein domains (n=14) for predicting stability changes upon mutation ddG in curated Megascale holdout dataset, and predicting binding-affinity changes upon mutation across varying antibody-antigen complexes (n=27) in AB-Bind dataset.

**Extended Data Table 1:**
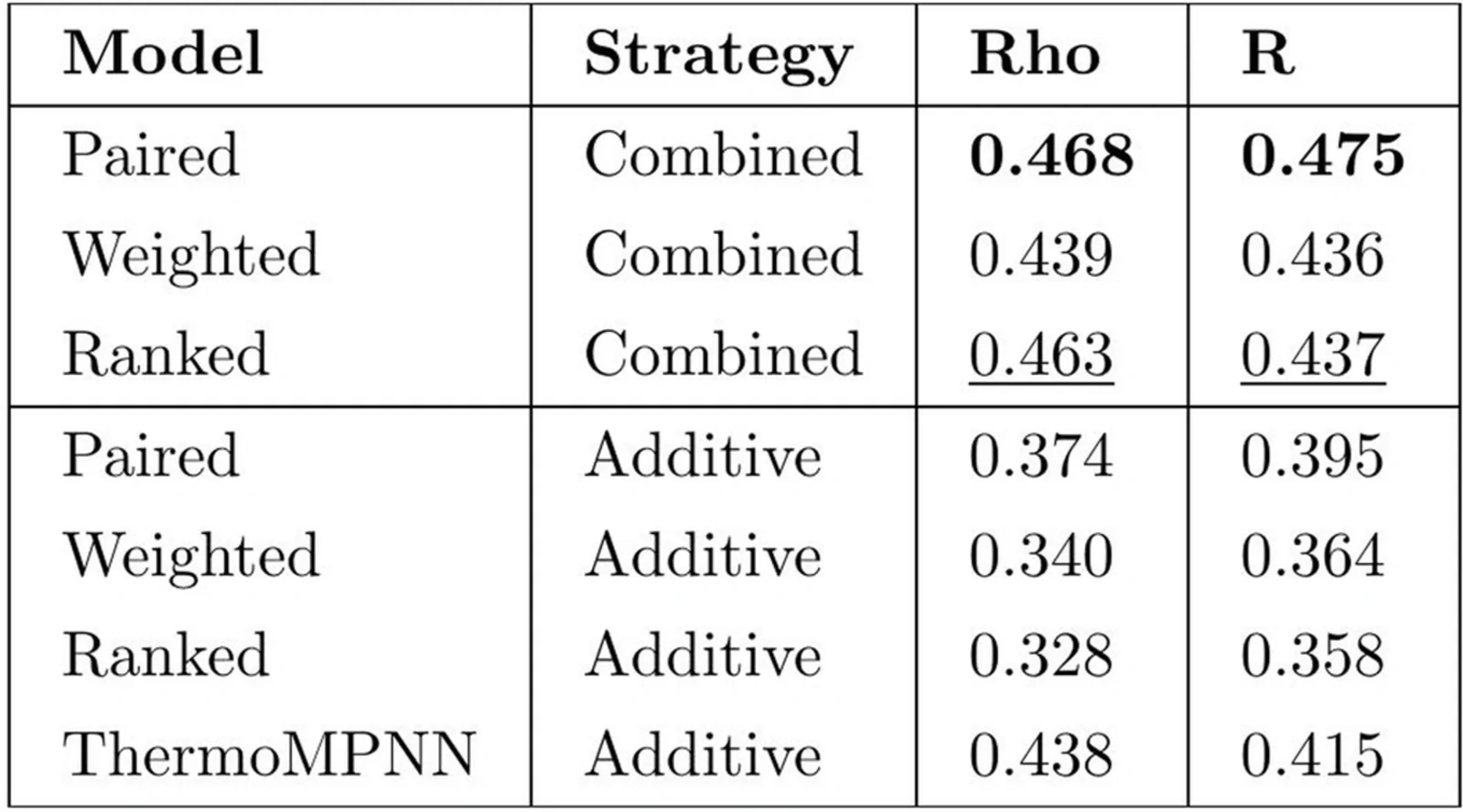
Additive versus combined scoring comparison for ProteinDPO models on double mutations

Additive and whole-sequence scoring on Megascale holdout double mutations (n=20,202) using ProteinDPO models and ThermoMPNN. Additive scoring refers to adding the respective log-likelihoods of each mutation evaluated individually, while combined scoring refers to adding the log-likelihood of both mutations while evaluating them simultaneously.

**Extended Data Table 2:**
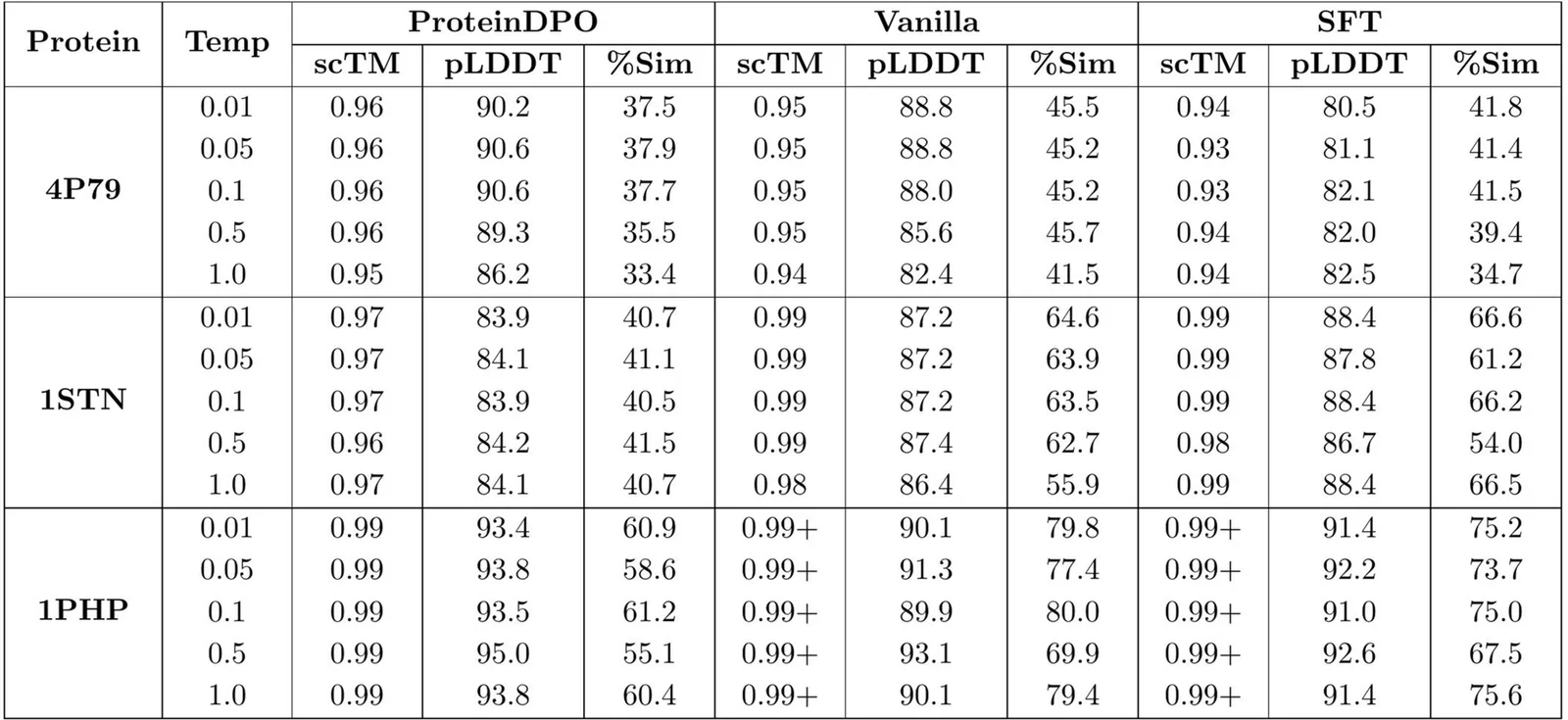
Self-consistency evaluation of protein structure generation at different temperatures

Self-consistency of generations for 4P79, 1STN, and 1PHP, evaluated with the TM score of the input backbone and ESMFold structure (scTM) and confidence of the predicted structure (pLDDT). For each temperature metrics were averaged across n=100 samples, for a total of 500 samples per model per protein.

**Extended Data Table 3:**
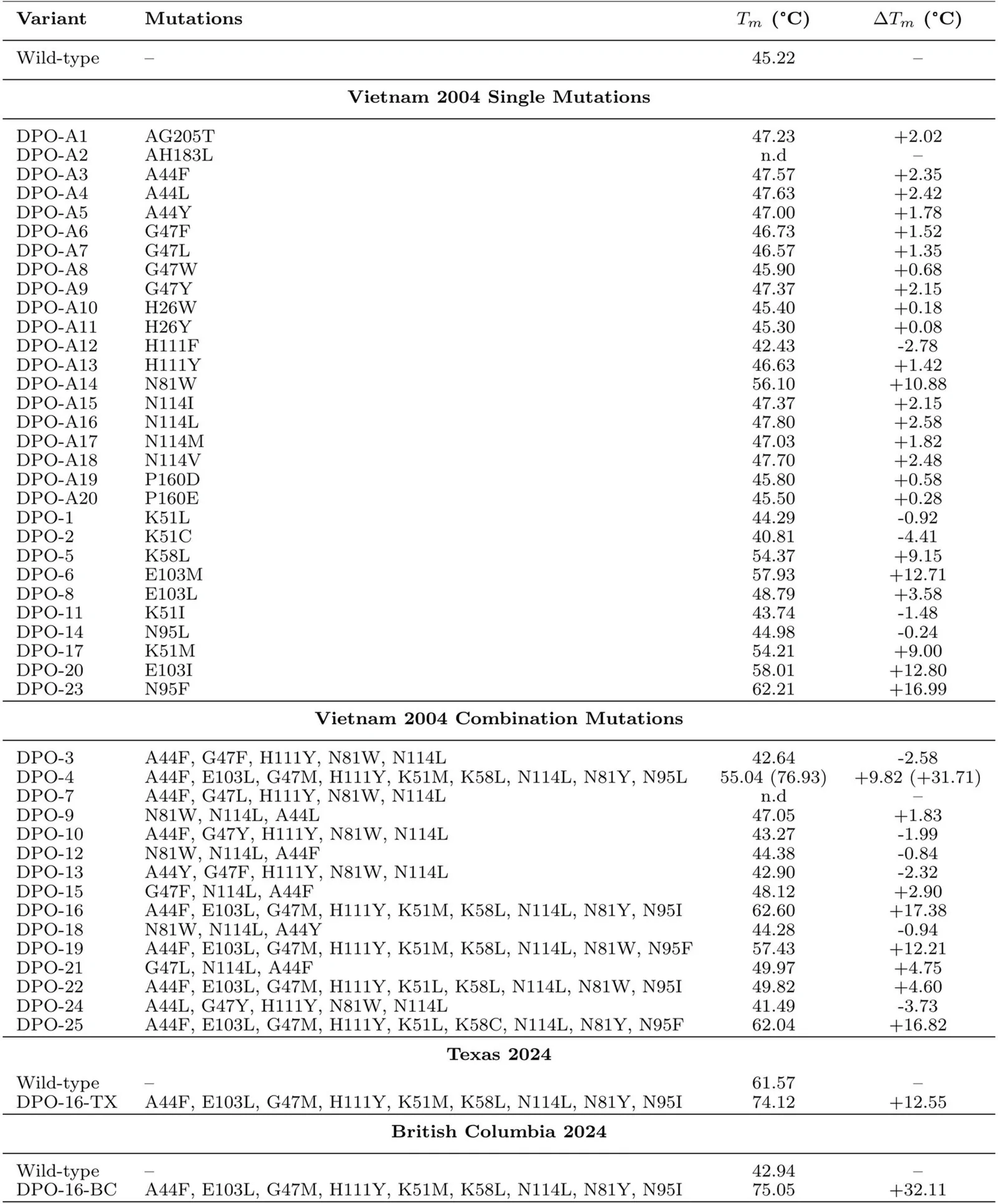
Experimental melting temperatures for ProteinDPO-identified mutations

Melting temperatures of protein variants as determined by NanoTemper Prometheus software and improvement over their respective wild-type sequence. At least three replicates were performed per measurement, and reported value is average over replicates. Unless otherwise denoted, all mutations are applied to chain B of the structure as demarcated in PDB 6CFG.

**Extended Data Table 4:**
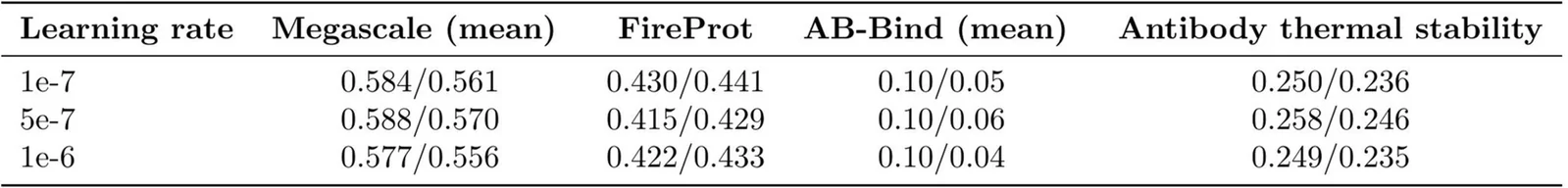
Learning rate comparison for supervised fine-tuning checkpoints

Comparison of different SFT checkpoints trained with varying learning rate on stability and binding prediction in terms of (mean) Pearson R / Spearman rho correlation coefficients.

## Data Availability

We used the Megascale^18^ dataset for training, either with our own training splits (derived from v2 4/20/2023), those from Diekhaus et al.^13^ or those from Cuturello et al.^25^, depending on subsequent evaluation. We used the following datasets for evaluation: Homolog-free FireProt^13,27^, S669^26^, antibody thermal stability^33,34^, SKEMPIv2^28^ and AB-Bind^29^. Additional details on these datasets are provided in the Methods. Source data are provided with this paper.

## Code availability

Code for ProteinDPO is available via GitHub at https://github.com/evo-design/protein-dpo. Weights for the model trained with the paired DPO objective are available via Zenodo at https://doi.org/10.5281/zenodo.11218181 (ref. ^71^).
