# Supplementary material for: Aligning protein-generative models to experimental fitness with ProteinDPO
Source: Nat Methods. 2026 Aug 14;23(9):1805–13. doi: 10.1038/s41592-026-03137-3 (PMC13541614; doi:10.1038/s41592-026-03137-3)
Supplement: Supplementary file 1 [file 41592_2026_3137_MOESM1_ESM.pdf]

nature portfolio

Corresponding author(s): Brian Lance Hie

Last updated by author(s): Mar 12, 2026

Reporting Summary

Nature Portfolio wishes to improve the reproducibility of the work that we publish. This form provides structure for consistency and transparency in reporting. For further information on Nature Portfolio policies, see our Editorial Policies and the Editorial Policy Checklist.

Statistics

For all statistical analyses, confirm that the following items are present in the figure legend, table legend, main text, or Methods section.

n/a	Confirmed	
☒	☐	The exact sample size (*n*) for each experimental group/condition, given as a discrete number and unit of measurement
☒	☐	A statement on whether measurements were taken from distinct samples or whether the same sample was measured repeatedly
☒	☐	The statistical test(s) used AND whether they are one- or two-sided<br>*Only common tests should be described solely by name; describe more complex techniques in the Methods section.*
☒	☐	A description of all covariates tested
☒	☐	A description of any assumptions or corrections, such as tests of normality and adjustment for multiple comparisons
☒	☐	A full description of the statistical parameters including central tendency (e.g. means) or other basic estimates (e.g. regression coefficient) AND variation (e.g. standard deviation) or associated estimates of uncertainty (e.g. confidence intervals)
☒	☐	For null hypothesis testing, the test statistic (e.g. *F*, *t*, *r*) with confidence intervals, effect sizes, degrees of freedom and *P* value noted<br>*Give P values as exact values whenever suitable.*
☒	☐	For Bayesian analysis, information on the choice of priors and Markov chain Monte Carlo settings
☒	☐	For hierarchical and complex designs, identification of the appropriate level for tests and full reporting of outcomes
☒	☐	Estimates of effect sizes (e.g. Cohen's *d*, Pearson's *r*), indicating how they were calculated

Our web collection on statistics for biologists contains articles on many of the points above.

Software and code

Policy information about availability of computer code

Data collection	Thermal stability - PR.ThermControl software, version 2.3.1; Biolayer Interferometry (BLI) - Octet System Data Analysis Software version 13.0.3.52
Data analysis	N/A

For manuscripts utilizing custom algorithms or software that are central to the research but not yet described in published literature, software must be made available to editors and reviewers. We strongly encourage code deposition in a community repository (e.g. GitHub). See the Nature Portfolio guidelines for submitting code & software for further information.

Data

Policy information about availability of data

All manuscripts must include a data availability statement. This statement should provide the following information, where applicable:

- Accession codes, unique identifiers, or web links for publicly available datasets
- A description of any restrictions on data availability
- For clinical datasets or third party data, please ensure that the statement adheres to our policy

Section 8 (Data availability) and section 7.5 (Datasets)

Research involving human participants, their data, or biological material

Policy information about studies with human participants or human data. See also policy information about sex, gender (identity/presentation), and sexual orientation and race, ethnicity and racism.

Reporting on sex and gender	N/A
Reporting on race, ethnicity, or other socially relevant groupings	N/A
Population characteristics	N/A
Recruitment	N/A
Ethics oversight	N/A

Note that full information on the approval of the study protocol must also be provided in the manuscript.

Field-specific reporting

Please select the one below that is the best fit for your research. If you are not sure, read the appropriate sections before making your selection.

☒ Life sciences ☐ Behavioural & social sciences ☐ Ecological, evolutionary & environmental sciences

For a reference copy of the document with all sections, see nature.com/documents/nr-reporting-summary-flat.pdf

Life sciences study design

All studies must disclose on these points even when the disclosure is negative.

Sample size	The same size of hemagglutinin (HA) variants tested was intended to be as large as possible while falling within a reasonable experimental workload.
Data exclusions	Thermal melting profiles which were clearly highly variable due to improper loading of the sample were excluded, and experiments were re-ran until a reliable measurement could be obtained.
Replication	Thermal melting experiments were performed in triplicate, and BLI experiments used at least two replicate experiments to determine affinity.
Randomization	The order and batch assignments for the production and testing of hemagglutinin (HA) variants was completely randomized.
Blinding	Blinding was not necessary due to the use of objective and unbiased external software for data collection.

Reporting for specific materials, systems and methods

We require information from authors about some types of materials, experimental systems and methods used in many studies. Here, indicate whether each material, system or method listed is relevant to your study. If you are not sure if a list item applies to your research, read the appropriate section before selecting a response.

Materials & experimental systems

n/a	Involved in the study	
☐	☒	Antibodies
☐	☒	Eukaryotic cell lines
☒	☐	Palaeontology and archaeology
☒	☐	Animals and other organisms
☒	☐	Clinical data
☒	☐	Dual use research of concern
☒	☐	Plants

Methods

n/a	Involved in the study	
☒	☐	ChIP-seq
☒	☐	Flow cytometry
☒	☐	MRI-based neuroimaging

Antibodies

Antibodies used	Anti-H5 IgG antibodies 13D4 (Q. Zheng et al., 2011) and CR6261 (D. C. Ekiert et al., 2009), and anti-SARS-CoV-2 RBD antibody AZD3152 (Y. Cai et al., 2024) were produced in-house.

Validation	In-house produced antibodies anti-H5 13D4 and CR6261 have been verified to bind to recombinant hemagglutinin (HA) extracellular domain from A/Vietnam/1203/04 (H5N1) by biolayer interferometry, while anti-SARS-CoV-2 RBD AZD3152 was verified to be non-HA binding. Crystal structures are available for the Fab domains of each antibody bound to their target: 13D4 (6A0Z); CR6261 (3GBN); AZD3152 (8SUO).

Eukaryotic cell lines

Policy information about cell lines and Sex and Gender in Research

Cell line source(s)	Expi293F cells were obtained from Thermo Fisher Scientific (A14527)
Authentication	No further authentication of the cell lines was carried after out after acquisition from their suppliers.
Mycoplasma contamination	Cell lines were not tested for mycoplasma contamination.
Commonly misidentified lines (See ICLAC register)	There were no commonly misidentified lines used in this study.

Plants

Seed stocks	N/A
Novel plant genotypes	N/A
Authentication	N/A
