# Supplementary material for: Aligning protein-generative models to experimental fitness with ProteinDPO
Source: Nat Methods. 2026 Aug 14;23(9):1805–13. doi: 10.1038/s41592-026-03137-3 (PMC13541614; doi:10.1038/s41592-026-03137-3)
Supplement: Supplementary file 2 [file 41592_2026_3137_MOESM2_ESM.pdf]

nature portfolio

# Peer Review File

# Aligning protein generative models to experimental fitness with ProteinDPO

Corresponding Author: Dr Brian Hie

**This file contains all editorial decision letters in order by version, followed by all author rebuttals in order by version.**

Version 0:

Decision Letter:

19th Sep 2025

Dear Brian,

Your Article, "Aligning protein generative models to experimental fitness with ProteinDPO", has now been seen by 3 reviewers. As you will see from their comments below, although the reviewers find your work of considerable potential interest, they have raised a number of concerns. We continue to be interested in potentially publishing your manuscript in Nature Methods, but would like to evaluate your response to these concerns before we reach a final decision on publication.

We therefore invite you to revise your manuscript to address these concerns. We recommend adding more comprehensive benchmarking against available methods in this field as well as addressing all the technical concerns. Regarding Reviewer #1's point 2 (vaccine relevance) - additional experiments to demonstrate that designed variants elicit protective immune responses against the target virus would be out of scope for us, so we recommend you tone down these claims.

We are committed to providing a fair and constructive peer-review process. Do not hesitate to contact us if there are specific requests from the reviewers that you believe are technically impossible or unlikely to yield a meaningful outcome.

When revising your paper:

* include a point-by-point response to the reviewers and to any editorial suggestions

* please underline/highlight any additions to the text or areas with other significant changes to facilitate review of the revised manuscript

* address the points listed described below to conform to our open science requirements

* ensure it complies with our general format requirements as set out in our guide to authors at www.nature.com/naturemethods

* resubmit all the necessary files by using the link below to access your home page

Link Redacted

**Note:** This URL links to your confidential account and associated information about manuscripts you may have submitted, or that you are reviewing for us. If you wish to forward this email to co-authors, please delete this link.

We hope to receive your revised paper within 6-8 weeks. If you are substantially delayed, please let us know. In this event, we will still be happy to reconsider your paper at a later date so long as nothing similar has been accepted for publication at Nature Methods or published elsewhere.

OPEN SCIENCE REQUIREMENTS

REPORTING SUMMARY
When revising your manuscript, please update your reporting summary.

Reporting summary: https://www.nature.com/documents/nr-reporting-summary.zip

If your paper includes custom software, we also ask you to complete a supplemental reporting summary.

Software supplement: https://www.nature.com/documents/nr-software-policy.pdf

Please submit these with your revised manuscript. They will be available to reviewers to aid in their evaluation if the paper is re-reviewed. If you have any questions about the checklist, please see http://www.nature.com/authors/policies/availability.html or contact me.

Please note that these forms are dynamic 'smart pdfs' and must therefore be downloaded and completed in Adobe Reader. We will then flatten them for ease of use by the reviewers. If you would like to reference the guidance text as you complete the template, please access these flattened versions at http://www.nature.com/authors/policies/availability.html.

For any revision that includes light microscopy data, we ask our authors to please include a completed light microscopy reporting table [https://www.nature.com/documents/Light_microscopy_reporting_table.xlsx] to ensure the methods are described thoroughly. The table will be available to reviewers and ultimately published should the manuscript be accepted at the journal.

IMAGE INTEGRITY
When submitting the revised version of your manuscript, please pay close attention to our <a href="https://www.nature.com/nature-research/editorial-policies/image-integrity">Digital Image Integrity Guidelines</a> and to the following points below:

-- that unprocessed scans are clearly labelled and match the gels and western blots presented in figures.
-- that control panels for gels and western blots are appropriately described as loading on sample processing controls
-- all images in the paper are checked for duplication of panels and for splicing of gel lanes.

Finally, please ensure that you retain unprocessed data and metadata files after publication, ideally archiving data in perpetuity, as these may be requested during the peer review and production process or after publication if any issues arise.

EXTENDED DATA FIGURES
When re-submitting your manuscript, please ensure that any supplementary figures and tables that are crucial to the manuscript's conclusions are converted into Extended Data figures and tables to increase visibility of these data. Extended Data figures and tables are online-only (present in the online PDF and full-text HTML versions of the paper), peer-reviewed display items that provide essential background to the article but are not included in the main article due to space constraints. A maximum of ten Extended Data display items (figures and tables) is permitted.

DATA AVAILABILITY
We strongly encourage you to deposit all new data associated with the paper in a persistent repository where they can be freely and enduringly accessed. We recommend submitting the data to discipline-specific and community-recognized repositories; a list of repositories is provided here: http://www.nature.com/sdata/policies/repositories

All novel DNA and RNA sequencing data, protein sequences, genetic polymorphisms, linked genotype and phenotype data, gene expression data, macromolecular structures, and proteomics data must be deposited in a publicly accessible database, and accession codes and associated hyperlinks must be provided in the "Data Availability" section.

For papers containing bioimaging data, we strongly recommend depositing the data to Bioimage Archive (https://www.ebi.ac.uk/bioimage-archive/). Associated accession codes and hyperlinks should be provided in the "Data Availability" section.

Refer to our data policies here: https://www.nature.com/nature-research/editorial-policies/reporting-standards#availability-of-data

To further increase transparency, we encourage you to provide, in tabular form, the data underlying the graphical representations used in your figures. This is in addition to our data-deposition policy for specific types of experiments and large datasets. For readers, the source data will be made accessible directly from the figure legend. Spreadsheets can be submitted in .xls, .xlsx or .csv formats. Only one (1) file per figure is permitted: thus if there is a multi-paneled figure the source data for each panel should be clearly labeled in the csv/Excel file; alternately the data for a figure can be included in multiple, clearly labeled sheets in an Excel file. File sizes of up to 30 MB are permitted. When submitting source data files with your manuscript please select the Source Data file type and use the Title field in the File Description tab to indicate which figure the source data

pertains to.

Please include a “Data availability” subsection in the Online Methods. This section should inform readers about the availability of the data used to support the conclusions of your study, including accession codes to public repositories, references to source data that may be published alongside the paper, unique identifiers such as URLs to data repository entries, or data set DOIs, and any other statement about data availability. At a minimum, you should include the following statement: “The data that support the findings of this study are available from the corresponding author upon request”, describing which data is available upon request and mentioning any restrictions on availability. If DOIs are provided, please include these in the Reference list (authors, title, publisher (repository name), identifier, year). For more guidance on how to write this section please see: http://www.nature.com/authors/policies/data/data-availability-statements-data-citations.pdf

CODE AVAILABILITY
Please include a “Code Availability” subsection in the Online Methods which details how your custom code is made available. Only in rare cases (where code is not central to the main conclusions of the paper) is the statement “available upon request” allowed (and reasons should be specified).

We request that you deposit code in a DOI-minting repository such as Zenodo, Gigantum or Code Ocean and cite the DOI in the Reference list. We also request that you use code versioning and provide a license.

For more information on our code sharing policy and requirements, please see: https://www.nature.com/nature-research/editorial-policies/reporting-standards#availability-of-computer-code

MATERIALS AVAILABILITY
As a condition of publication in Nature Methods, authors are required to make unique materials promptly available to others without undue qualifications.

Authors reporting new chemical compounds must provide chemical structure, synthesis and characterization details. Authors reporting mutant strains and cell lines are strongly encouraged to use established public repositories.

More details about our materials availability policy can be found at https://www.nature.com/nature-portfolio/editorial-policies/reporting-standards#availability-of-materials

SUPPLEMENTARY PROTOCOL
To help facilitate reproducibility and uptake of your method, we ask you to prepare a step-by-step Supplementary Protocol for the method described in this paper. We <a href="https://www.nature.com/nature-research/editorial-policies/reporting-standards#protocols" target="new">encourage authors to share their step-by-step experimental protocols</a> on a protocol sharing platform of their choice and report the protocol DOI in the reference list. Nature Portfolio's protocols.io is a free-to-use and open resource for protocols; protocols deposited onto protocols.io are citable and can be linked from the published article. More details can found at <a href="https://www.protocols.io/help/publish-articles" target="new">protocols.io</a>.

ORCID
Nature Methods is committed to improving transparency in authorship. As part of our efforts in this direction, we are now requesting that all authors identified as ‘corresponding author’ on published papers create and link their Open Researcher and Contributor Identifier (ORCID) with their account on the Manuscript Tracking System (MTS), prior to acceptance. This applies to primary research papers only. ORCID helps the scientific community achieve unambiguous attribution of all scholarly contributions. You can create and link your ORCID from the home page of the MTS by clicking on ‘Modify my Springer Nature account’. For more information please visit please visit <a href="http://www.springernature.com/orcid">www.springernature.com/orcid</a>.

Please do not hesitate to contact me if you have any questions or would like to discuss these revisions further. We look forward to seeing your revised manuscript and thank you for the opportunity to consider your work.

Best regards,
Arunima

Arunima Singh, Ph.D.
Senior Editor
Nature Methods

Reviewers' Comments:

Reviewer #1 (Remarks to the Author):

In this work, the authors introduce an approach called Direct Preference Optimization (DPO) to enhance desired properties of protein sequences generated by computational models. Specifically, they focus on protein stability using the inverse protein folding model ESM-IF1, and further optimize the model with protein mutational data. The authors report improvements in several benchmark tests compared with the original unsupervised ESM-IF1 and a fine-tuned supervised model, and in some cases, with another approach named ThermoMPNN. Finally, they apply the method to design hemagglutinin variants for influenza vaccines, reporting increased stability. The study presents an interesting strategy for optimizing deep learning models in protein design. However, I am not fully convinced that the proposed approach provides a significant advantage based on the current evidence. My major concerns are outlined below:

1. Lack of comparison in the HA application. While benchmark improvements are demonstrated, the application to HA design, arguably the most important example in this work, was not compared with other existing approaches. Since ESM-IF1 itself has shown some promising performance in similar tasks, it remains unclear how much Protein DPO actually improves upon it. A detailed comparison with alternative methods is necessary to convincingly establish the benefit of Protein DPO.

2. Vaccine relevance not fully established. For vaccine design, the key requirement is demonstrating that designed variants elicit protective immune responses against the target virus. The manuscript shows recognition of designed variants by existing antibodies, but this does not sufficiently demonstrate antigenic properties required for vaccine efficacy. Stronger experimental validation would be needed to support claims of relevance to vaccine design.

3. Potential overemphasis on stability. The importance of stability may be overstated. For example, Figure 3D shows that the model generates sequences with improved stability according to Rosetta energy functions, but it is unclear whether these sequences are experimentally synthesized. Extremely stable sequences often risk aggregation due to excess hydrophobic residues, which may hinder practical expression and use. Moreover, the sequence design procedure is insufficiently described in the Methods. Was it simply initiated from random seeds, as in ESM-IF1?

4. In Section 2.3, the claim that the method can rank “absolute stability of diverse antibodies” seems misleading, since only correlation coefficients are reported. These results reflect relative stability rankings, not absolute values. Furthermore, comparisons across antibodies likely rely on their shared fold and similar length. It is doubtful that the approach could be applied to rank melting temperatures across proteins of different folds.

5. The Methods section needs clarification and expansion, particularly regarding benchmark evaluation. A separate subsection should clearly describe the metrics used (e.g., correlation coefficients), how they are computed, and the specific model outputs used for evaluation. In addition, figures such as 2B and 3B would benefit from showing distributional statistics rather than only averages, as mean values can obscure important variation.

6. The main contribution of this work lies in the proposed algorithm for optimizing ESM-IF1, but the Methods provide insufficient implementation details. Moreover, the GitHub repository only contains scripts and weights for inference, without the training code or implementation necessary for reproduction. At minimum, the authors should provide pseudocode for their approach—particularly for the weighted DPO algorithm, which is a novel aspect of this study.

Reviewer #2 (Remarks to the Author):

This paper applies the Direct Preference Optimization (DPO) approach to "align" the structure-conditioned protein language model ESM-IF with the objective of designing high stability protein sequences. The authors point out that the "base" model ESM-IF is trained to recreate the probability distribution of the training data, which does not match the common objective in protein design to produce high stability proteins. The DPO method improves the alignment between the distribution sampled by the model and the author's (and community's) design objective of sampling high stability proteins. The resulting model (ProteinDPO) can be used for both scoring structures and for generating new sequences based on an input protein backbone. The authors examine several variants of ProteinDPO, computationally benchmark ProteinDPO on several design and ranking tasks against other leading models, and also apply ProteinDPO to experimentally design vaccine antigens with higher stability.

The main strengths of the paper are:

-The problem of improving generative AI models for protein design is timely and important and the approach of using DPO based on experimental folding stability data is relatively novel.

-The flexiblity of ProteinDPO enables the model to be applied to score multi-mutants as well as entire unrelated structures, which is rare in the field of stability prediction. The model achieves superior performance over a naive application of ThermoMPNN at predicting effects of multiple mutants (Fig. 2C). The model also shows a modest correlation with antibody binding and melting temperature (Fig 3B/C), which is superior to ESM-IF ("Vanilla") and cannot achieved using models like ThermoMPNN or MAESTRO that are designed to score single mutants.

-For predicting single mutants, several variants of ProteinDPO have strong performance at the S669 benchmark and come close to matching ThermoMPNN at the Fireprot benchmark.

-Using a computational benchmark, the authors show that ProteinDPO-based protein design improves on ESM-IF based

protein design according to the Rosetta energy functon(Fig. 3D).

-The experimental work demonstrates a successful application of ProteinDPO. However, this work is seemingly not intended to illustrate the superiority of ProteinDPO over other methods- the proposed mutants are not computationally evaluated using any method other than ProteinDPO.

The main weaknesses of the paper are:

-Although the authors make the case that ProteinDPO has unique applications beyond other stability models (such as Fig. 2C, Fig 2B, Fig 3C), the experimental demonstration doesn't really highlight these applications. Instead, it illustrates an application where other stability models might have performed equally well or better.

-The authors don't examine the performance of other stability models at their experimental demonstration. It would be very useful to the audience to know whether the output of ProteinDPO is unique or similar to predictions made by other stability models. At minimum, the authors should take the top 30 mutants designed by ProteinDPO (the ones that were experimentally tested), examine how these mutants score according to "vanilla" ESM-IF and ThermoMPNN, and report (1) whether the ProteinDPO-designed mutants are predicted to be favorable according to other methods, (2) whether they also rank near the top of all mutants evaluated by other methods, and (3) whether other methods show an ability rank mutants by Tm within the top 30 designed by ProteinDPO. It would also be good to note whether ProteinDPO itself can rank these 30 mutants, although this is a somewhat unfair task since they are all already highly favorable according to ProteinDPO.

-The benchmarking of ProteinDPO against other models for antibody melting temperature and protein-protein binding is somewhat minimal. Only ESM-IF is used as a benchmark. Are any other benchmarks available? At minimum, RosettaREU (total energy, energy per residue, or ddG) could be used, as in Fig 3D.

-The authors need to be careful about how they describe the overall accuracy of the ProteinDPO method. For example, in the 4th paragraph of the discussion, the authors write "ProteinDPO also generalizes to a greater variety of tasks than existing supervised models, being able to predict ΔΔG, binding affinity, and thermal melting across diverse wild-type proteins, multi-chain antibodies, and protein complexes." This ability to "predict binding affinity" is based on spearman rho values of 0.2 - 0.33 - poor overall. The ability predict thermal melting is based on spearman rho of ~0.35 - again poor. It would be accurate to say that the ProteinDPO model can predict these quantities ***more accurately than the vanilla ESM-IF model***, which is a worthwhile computational achievement. But the authors need to avoid unqualified statements like saying "ProteinDPO can predict X".

Short comments to the authors:

Fig 2 caption: It's not correct to say that FIreProt and S669 are made from deep mutational scanning datasets since these are generally piecemeal individual measurements, not multiplexed comprehensive measurements.

Fig 3B: should the the lower right panel be AUROC?

Fig 4B: It seems like the best single mutant is as good as the best combination of mutants? Is this correct? Does this mean that combining mutants was unsuccessful? Or is the light blue point at the top not a single mutant? A different coloring scheme would be beneficial. The authors should be more explicit in the text about the best single mutant.

Fig 5D: The CD wave scans look unusual- why is the signal so much weaker in DPO-19 and WT compared to DPO-4? If the proteins are folded and have the same structure, they should show the same spectrum. Is it possible the protein concentrations were measured incorrectly for all proteins except DPO-4? The methods should mention how protein concentrations were measured. A folded alpha-beta protein should have a signal with the intensity of the signal for DPO-4 (see a reference on this)- the other signals are strangely weak.

Methods: The authors should state which version of the Megascale dataset they used (v1 12/6/2022 or v2 4/20/2023).

Gabriel Rocklin
Northwestern University

Reviewer #3 (Remarks to the Author):

This work by Widatalla et al applies the DPO method to protein stability engineering problem. Protein stability engineering is clearly an important research subject, and the application of DPO technique to this problem is somewhat novel. There were several works in which protein language models were fine-tuned for the purpose, but to my knowledge, the application of DPO seems the first. I think validating their method through actual experiments is very encouraging and valuable; however, two major concerns listed below make it hard to fully appreciate the quality of the work.

1) The first is the basic philisophy of the work. From a fundamental point of view, is "tuning a pre-trained model" a relevant philosophy for the protein stability estimation? Regarding the complexity of protein folding and stability, I think this idea will only give a marginal improvement over existing methods sharing the same concept; i.e. another (slightly better) ThermoMPNN. A good work to compare is the recently published BioEmu, in which the authors simulated protein folding to approximate the equilibrium. I cannot see such conceptual novelty in this work that tackles the inherent challenge of the problem. Introducing

DPO technique may add little novelty in this regard.

2) The method is repeatedly compared against vanilla or STF, but I don't think this is a valuable comparison. This may show "how bad ESM-if" rather than "how good ProteinDPO" is for the problem. Also, it is hard to believe SFT in the manuscript was trained by their best. Therefore, it is worth testing just once for ablation, and the main comparison should be against other methods like ThermoMPNN. Also, the authors keep argue "method solely trained on small proteins generalizes to bigger protein". This is not an advantage but a prerequisite; methods not satisfying such property should be less appreciated.

1-1) Comparison to other methods is not very strong, but just based on the current figures, it is hard to identify clear advantages of the method. This marginal (or neglible) improvement should be related to the inherent limitation of the approach as pointed out in point 1.

Minor point.
1) This is a suggestion for the presentation of the experimental section. As I mentioned above, experimental validation is very encouraging. At the same time, readers may wonder what if other already-established method was tested in parallel. For example, ProteinMPNN already demonstrated its ability to improve stability in various previous works other methods (e.g. Rosetta) failed. It should have been much more impactful if ProteinDPO have proved experimetnal success in a similar way (although a relevant baseline now has largely shifted from Rosetta to ProteinMPNN since then). Otherwise readers may have impression that results presented were cherry-picked.

Reviewer #3 (Remarks on code availability):

I was not able to install the code with the error message below:

"Solving environment: failed
ResolvePackageNotFound:
- python[version='3.10.*,==3.9.18',build=h955ad1f_0]"

I do see this issue was posted in the github a bit ago, but was not fixed. Please fix this issue before your resubmission.

The inference code itself is very simple and straightforward.

Version 1:

Decision Letter:

15th Jan 2026

Dear Brian,

Thank you for your letter detailing how you would respond to the reviewer concerns regarding your Article, "Aligning protein generative models to experimental fitness with ProteinDPO". We have decided to invite you to revise your manuscript as you have outlined, before we reach a final decision on publication.

Please do not hesitate to contact me if you have any questions or would like to discuss these revisions further.

When revising your paper:

* include a point-by-point response to the reviewers and to any editorial suggestions

* please underline/highlight any additions to the text or areas with other significant changes to facilitate review of the revised manuscript

* address the points listed described below to conform to our open science requirements

* ensure it complies with our general format requirements as set out in our guide to authors at www.nature.com/naturemethods

* resubmit all the necessary files by using the link below to access your home page

Link Redacted

**Note:** This URL links to your confidential account and associated information about manuscripts you may have submitted, or that you are reviewing for us. If you wish to forward this email to co-authors, please delete this link.

We hope to receive your revised paper within 4-6 weeks. If you are substantially delayed, please let us know. In this event, we will still be happy to reconsider your paper at a later date so long as nothing similar has been accepted for publication at

Nature Methods or published elsewhere.

OPEN SCIENCE REQUIREMENTS

REPORTING SUMMARY
When revising your manuscript, please update your reporting summary.

Reporting summary: https://www.nature.com/documents/nr-reporting-summary.zip

If your paper includes custom software, we also ask you to complete a supplemental reporting summary.

Software supplement: https://www.nature.com/documents/nr-software-policy.pdf

Please submit these with your revised manuscript. They will be available to reviewers to aid in their evaluation if the paper is re-reviewed. If you have any questions about the checklist, please see http://www.nature.com/authors/policies/availability.html or contact me.

Please note that these forms are dynamic 'smart pdfs' and must therefore be downloaded and completed in Adobe Reader. We will then flatten them for ease of use by the reviewers. If you would like to reference the guidance text as you complete the template, please access these flattened versions at http://www.nature.com/authors/policies/availability.html.

For any revision that includes light microscopy data, we ask our authors to please include a completed light microscopy reporting table [https://www.nature.com/documents/Light_microscopy_reporting_table.xlsx] to ensure the methods are described thoroughly. The table will be available to reviewers and ultimately published should the manuscript be accepted at the journal.

EXTENDED DATA FIGURES
When re-submitting your manuscript, please ensure that any supplementary figures and tables that are crucial to the manuscript's conclusions are converted into Extended Data figures and tables to increase visibility of these data. Extended Data figures and tables are online-only (present in the online PDF and full-text HTML versions of the paper), peer-reviewed display items that provide essential background to the article but are not included in the main article due to space constraints. A maximum of ten Extended Data display items (figures and tables) is permitted.

DATA AVAILABILITY
Please include a "Data availability" subsection in the Online Methods. This section should inform readers about the availability of the data used to support the conclusions of your study, including accession codes to public repositories, references to source data that may be published alongside the paper, unique identifiers such as URLs to data repository entries, or data set DOIs, and any other statement about data availability. At a minimum, you should include the following statement: "The data that support the findings of this study are available from the corresponding author upon request", describing which data is available upon request and mentioning any restrictions on availability. If DOIs are provided, please include these in the Reference list (authors, title, publisher (repository name), identifier, year). For more guidance on how to write this section please see:
http://www.nature.com/authors/policies/data/data-availability-statements-data-citations.pdf

For papers containing bioimaging data, we strongly recommend depositing the data to Bioimage Archive (https://www.ebi.ac.uk/bioimage-archive/). Associated accession codes and hyperlinks should be provided in the "Data Availability" section.

CODE AVAILABILITY
Please include a "Code Availability" subsection in the Online Methods which details how your custom code is made available. Only in rare cases (where code is not central to the main conclusions of the paper) is the statement "available upon request" allowed (and reasons should be specified).

We request that you deposit code in a DOI-minting repository such as Zenodo, Gigantum or Code Ocean and cite the DOI in the Reference list. We also request that you use code versioning and provide a license.

For more information on our code sharing policy and requirements, please see:
https://www.nature.com/nature-research/editorial-policies/reporting-standards#availability-of-computer-code

MATERIALS AVAILABILITY
As a condition of publication in Nature Methods, authors are required to make unique materials promptly available to others without undue qualifications.

Authors reporting new chemical compounds must provide chemical structure, synthesis and characterization details. Authors

reporting mutant strains and cell lines are strongly encouraged to use established public repositories.

More details about our materials availability policy can be found at https://www.nature.com/nature-portfolio/editorial-policies/reporting-standards#availability-of-materials

SUPPLEMENTARY PROTOCOL
To help facilitate reproducibility and uptake of your method, we ask you to prepare a step-by-step Supplementary Protocol for the method described in this paper. We <a href="https://www.nature.com/nature-research/editorial-policies/reporting-standards#protocols" target="new">encourage authors to share their step-by-step experimental protocols</a> on a protocol sharing platform of their choice and report the protocol DOI in the reference list. Nature Portfolio's protocols.io is a free-to-use and open resource for protocols; protocols deposited onto protocols.io are citable and can be linked from the published article. More details can found at <a href="https://www.protocols.io/help/publish-articles" target="new">protocols.io</a>.

ORCID
Nature Methods is committed to improving transparency in authorship. As part of our efforts in this direction, we are now requesting that all authors identified as 'corresponding author' on published papers create and link their Open Researcher and Contributor Identifier (ORCID) with their account on the Manuscript Tracking System (MTS), prior to acceptance. This applies to primary research papers only. ORCID helps the scientific community achieve unambiguous attribution of all scholarly contributions. You can create and link your ORCID from the home page of the MTS by clicking on 'Modify my Springer Nature account'. For more information please visit please visit <a href="http://www.springernature.com/orcid">www.springernature.com/orcid</a>.

Please do not hesitate to contact me if you have any questions or would like to discuss these revisions further. We look forward to seeing the revised manuscript and thank you for the opportunity to consider your work.

Best regards,
Arunima

Arunima Singh, Ph.D.
Senior Editor
Nature Methods

Reviewers' Comments:

Reviewer #1 (Remarks to the Author):

The authors have satisfactorily addressed my concerns in this revision.

Reviewer #2 (Remarks to the Author):

The authors adequately addressed all of my comments except one small point: I would still like them to analyze and describe whether the stabilizing mutations identified by DPO are predicted to be stabilizing according to the other models. This is different from the top-N analysis performed by the authors. As the authors note, the top-N analysis is insufficient because the other stabilizing mutations predicted by the other method may also be stabilizing. For completeness you might as well just show the full distribution of mutation effects predicted by the other methods (which you have already computed), along with where the successful/unsuccessful mutations lie in the distribution.

as before: "At minimum, the authors should take the top 30 mutants designed by ProteinDPO (the ones that were experimentally tested), examine how these mutants score according to "vanilla" ESM-IF and ThermoMPNN, and report (1) whether the ProteinDPO-designed mutants are predicted to be favorable according to other methods" "

Gabriel Rocklin
Northwestern University

Reviewer #3 (Remarks to the Author):

I appreciate the authors' reasonable responses to points 2 and 3. However, I feel that point 1 has not yet been adequately addressed.

My original concern was not delivered clearly, so let me restate it. My comment was not intended to question the general

“pretrain-and-fine-tune” strategy itself, but rather to discuss the underlying physical motivation and conceptual foundation of the model. I referred to BioEmu because it is trained on protein dynamics and folding, which are directly relevant to stability estimation and thus provide a clear rationale for transfer learning in this context. In contrast, the pretrained model used for ProteinDPO (ESM-IF) is similar to that of ThermoMPNN (ProteinMPNN), which has not explicitly been trained with such objectives. For this reason, I would group ProteinDPO more closely with ThermoMPNN, while distinguishing it from BioEmu.

My concern is not about the relative performance of the methods — indeed, the differences appear to be marginal based on Figures 2C–E and 3B–C — but rather about the novelty of the approach and its potential for future extension. Adapting a sequence design model for stability prediction has already been explored by ThermoMPNN, albeit in a different formulation, and there may be a general consensus regarding its practical upper bound in performance. Moreover, simply characterizing a model as “generative” does not, by itself, address this limitation. For example, if ThermoMPNN were retrained to preserve the original ProteinMPNN "generation" functionality, it is unclear whether this would be a fundamentally novel approach.

I would therefore suggest to clearly discuss the methodological limitations of the proposed approach in the Discussion section, which may explain its marginal difference to ThermoMPNN.

Version 2:

Decision Letter:

Our ref: NMETH-A61939B

19th Feb 2026

Dear Brian,

Thank you for submitting your revised manuscript "Aligning protein generative models to experimental fitness with ProteinDPO" (NMETH-A61939B). It has now been seen by the original referees and their comments are below. The reviewers find that the paper has improved in revision, and therefore we'll be happy in principle to publish it in Nature Methods, pending minor revisions to satisfy the referees' final requests and to comply with our editorial and formatting guidelines.

We are now performing detailed checks on your paper and will send you a checklist detailing our editorial and formatting requirements within two weeks or so. Please do not upload the final materials and make any revisions until you receive this additional information from us.

TRANSPARENT PEER REVIEW
Nature Methods offers a transparent peer review option for new original research manuscripts submitted from 17th February 2021. We encourage increased transparency in peer review by publishing the reviewer comments, author rebuttal letters and editorial decision letters if the authors agree. Such peer review material is made available as a supplementary peer review file. **Please state in the cover letter ‘I wish to participate in transparent peer review’ if you want to opt in, or ‘I do not wish to participate in transparent peer review’ if you don’t.** Failure to state your preference will result in delays in accepting your manuscript for publication.

Please note: we allow redactions to authors’ rebuttal and reviewer comments in the interest of confidentiality. If you are concerned about the release of confidential data, please let us know specifically what information you would like to have removed. Please note that we cannot incorporate redactions for any other reasons. Reviewer names will be published in the peer review files if the reviewer signed the comments to authors, or if reviewers explicitly agree to release their name. For more information, please refer to our <a href="https://www.nature.com/documents/nr-transparent-peer-review.pdf" target="new">FAQ page</a>.

ORCID
IMPORTANT: Non-corresponding authors do not have to link their ORCIDs but are encouraged to do so. Please note that it will not be possible to add/modify ORCIDs at proof. Thus, please let your co-authors know that if they wish to have their ORCID added to the paper they must follow the procedure described in the following link prior to acceptance: https://www.springernature.com/gp/researchers/orcid/orcid-for-nature-research

**Author names using non-Roman characters**
Nature Portfolio journals can support presentation of author names using non-Roman characters in the HTML version of the article. If you wish to, please include author names in parentheses after the Roman-character spelling; <a href="https://www.nature.com/articles/s44222-024-00258-2">see example online here</a>. Currently supported scripts are: Arabic, Chinese, Cyrillic, Devanagari, Greek, Hebrew, Hangul, Japanese and Persian. You will be asked to verify the rendering is correct at proof stage.

Thank you again for your interest in Nature Methods. Please do not hesitate to contact me if you have any questions. We will be in touch again soon.

Sincerely,
Arunima

Arunima Singh, Ph.D.

Senior Editor
Nature Methods

Reviewer #2 (Remarks to the Author):

I approve of the final additions to the manuscript and find it suitable for publication as-is.

Reviewer #3 (Remarks to the Author):

The revised manuscript now contains much clearer explanation about the method's novelty. I recommend for the acceptance of this manuscript.

Reviewer #3 (Remarks on code availability):

The code is straightforward and works well. It would be helpful if the pip version requirement were updated to a more recent release (e.g., >25.x.x).

Version 3:

Decision Letter:

12th May 2026

Dear Brian,

I am pleased to inform you that your Article, "Aligning protein generative models to experimental fitness with ProteinDPO", has now been accepted for publication in Nature Methods. The received and accepted dates will be July 15, 2025 and May 12, 2026. This note is intended to let you know what to expect from us over the next month or so, and to let you know where to address any further questions.

Over the next few weeks, your paper will be copyedited to ensure that it conforms to Nature Methods style. Once your paper is typeset, you will receive an email with a link to choose the appropriate publishing options for your paper and our Author Services team will be in touch regarding any additional information that may be required. It is extremely important that you let us know now whether you will be difficult to contact over the next month. If this is the case, we ask that you send us the contact information (email, phone and fax) of someone who will be able to check the proofs and deal with any last-minute problems.

After the grant of rights is completed, you will receive a link to your electronic proof via email with a request to make any corrections within 48 hours. If, when you receive your proof, you cannot meet this deadline, please inform us at rjsproduction@springernature.com immediately.

**Authors may need to take specific actions to achieve compliance with funder and institutional open access mandates.**
If your research is supported by a funder that requires immediate open access (e.g. according to <a href="https://www.springernature.com/gp/open-science/plan-s-compliance"> Plan S principles</a> or the <a href="https://www.springernature.com/gp/open-science/us-federal-agency-compliance"> NIH public access policy</a>) then you should select the gold OA route, and we will direct you to the compliant route where possible. Because authors warrant under our subscription licensing terms that they haven't committed to licensing any version of their article under a licence inconsistent with the terms of our agreement – including the applicable embargo period – publication under the subscription model isn't suitable for authors whose funders require no embargo.

If you have any questions about our publishing options, costs, Open Access requirements, or our legal forms, please contact ASJournals@springernature.com

If you have posted a preprint on any preprint server, please ensure that the preprint details are updated with a publication reference, including the DOI and a URL to the published version of the article on the journal website.

You may wish to make your media relations office aware of your accepted publication, in case they consider it appropriate to organize some internal or external publicity. Once your paper has been scheduled you will receive an email confirming the publication details. This is normally 3-4 working days in advance of publication. If you need additional notice of the date and time of publication, please let the production team know when you receive the proof of your article to ensure there is sufficient time to coordinate. Further information on our embargo policies can be found here:
https://www.nature.com/authors/policies/embargo.html

To assist our authors in disseminating their research to the broader community, our SharedIt initiative provides you with a unique shareable link that will allow anyone (with or without a subscription) to read the published article. Recipients of the link with a subscription will also be able to download and print the PDF.

As soon as your article is published, you will receive an automated email with your shareable link.

If you are active on Twitter/X or Bluesky, please e-mail me your and your coauthors' handles so that we may tag you when the paper is published.

You can now use a single sign-on for all your accounts, view the status of all your manuscript submissions and reviews, access usage statistics for your published articles and download a record of your refereeing activity for the Nature journals.

Please note that you and any of your coauthors will be able to order reprints and single copies of the issue containing your article through Nature Portfolio's reprint website, which is located at http://www.nature.com/reprints/author-reprints.html. If there are any questions about reprints please send an email to author-reprints@nature.com and someone will assist you.

Please feel free to contact me if you have questions about any of these points.

Best regards,
Arunima

Arunima Singh, Ph.D.
Senior Editor
Nature Methods

** Visit the Springer Nature Editorial and Publishing website at <a href="http://editorial-jobs.springernature.com?utm_source=ejP_NMeth_email&utm_medium=ejP_NMeth_email&utm_campaign=ejp_Nmeth">www.springernature.com/editorial-and-publishing-jobs</a> for more information about our career opportunities. If you have any questions please click <a href="mailto:editorial.publishing.jobs@springernature.com">here</a>.**

**Open Access** This Peer Review File is licensed under a Creative Commons Attribution 4.0 International License, which permits use, sharing, adaptation, distribution and reproduction in any medium or format, as long as you give appropriate credit to the original author(s) and the source, provide a link to the Creative Commons license, and indicate if changes were made. In cases where reviewers are anonymous, credit should be given to 'Anonymous Referee' and the source.
The images or other third party material in this Peer Review File are included in the article's Creative Commons license, unless indicated otherwise in a credit line to the material. If material is not included in the article's Creative Commons license and your intended use is not permitted by statutory regulation or exceeds the permitted use, you will need to obtain permission directly from the copyright holder.
To view a copy of this license, visit https://creativecommons.org/licenses/by/4.0/

**General comments**

We thank all the reviewers for their thorough and constructive feedback on our manuscript. We appreciate the positive reception and are also grateful for their detailed comments which have led to substantial improvements to the clarity, rigor, and detail of our manuscript.

In this revision, we have:

1. *Added more comprehensive benchmarks*. Following reviewer requests, we have included comparisons to other models. Specifically, we now benchmark against ESM-IF1 (both zero-shot and after SFT), ThermoMPNN, and Rosetta for the HA stabilization task **(Fig. S5,6)**, demonstrating that ProteinDPO is superior in recovering literature mutations, while other models are not able to identify highly stabilizing variants from the literature or from our validated experimental pool. We have also added ProteinMPNN benchmarking for protein-protein binding **(Fig. 3B)** and RosettaREU evaluation for antibody thermal stability **(Fig. 3C)**, finding ProteinDPO has competitive or improved performance compared to benchmark models.

2. *Toned down claims*. We have carefully revised statements throughout the manuscript to ensure accurate representation of our results. This includes altering our claims with respect to vaccine design and clarifying that absolute stability predictions refer to relative improvements within a protein fold rather than absolute predictions across different folds. We have also added appropriate caveats regarding the limitations of computational metrics like Rosetta energy, and clarified that certain redesigned sequences in **Fig. 3** were not experimentally verified.

3. *Added more methodological details on our implementation and evaluations*. We have substantially expanded the **Methods** section to improve clarity. This includes new subsections for Model sampling, Sampling evaluation, Rosetta generation evaluation, and Scoring metrics **(Methods 6.6)**, detailed pseudocode for all training algorithms including Paired, Ranked, and Weighted DPO as well as SFT training **(Methods 6.7)**, and a new Mutation selection section describing the multi-mutation design protocol **(Methods 6.8)**. We have also added distributional plots for key results **(Fig. S3, S4)**, clarified dataset versions used, improved experimental methods descriptions, and fixed installation issues. In addition to pseudocode in the Methods, we attach the raw training code used for ProteinDPO as a supplementary zip file (**Supplementary Code**).

Other revisions are detailed individually in our point-by-point response below. These revisions address the reviewers' concerns while demonstrating the core contributions of our work: (1) that DPO can be used to align biological generative models with a desired experimental property and (2) our aligned model, ProteinDPO, improves upon its unsupervised predecessor to be useful as a stability predictor and stability-biased sequence generator, which we experimentally validate in the setting of influenza hemagglutinin.

## Point-by-point response

### Editorial comments

We recommend adding more comprehensive benchmarking against available methods in this field as well as addressing all the technical concerns. Regarding Reviewer #1's point 2 (vaccine relevance) - additional experiments to demonstrate that designed variants elicit protective immune responses against the target virus would be out of scope for us, so we recommend you tone down these claims.

We thank the editor for recognizing the interest in our work from the reviewers, and providing us the opportunity to improve our manuscript. To address the concern of comprehensive benchmarking, we have implemented the reviewers' suggestion of benchmarking against ProteinMPNN and Rosetta for binding and stability prediction (in addition to previous analyses benchmarking against both base and SFTed ESM-IF1), as well as benchmarking against a range of baseline models for the experimental application of hemagglutinin stabilization. We appreciate the guidance regarding vaccine relevance, and have toned down these claims accordingly. For example, in the **Abstract**:

*"Leveraging up to nine substitutions, we achieved a 17°C improvement in melting temperature on a HA from human H5N1 influenza A virus while preserving ~~antigenicity~~* ***recognition by existing antibodies****, and up to 32°C stability improvements across recently emerged mammalian viral H5 HAs."*

As well as in the **Results** section:

*"To determine whether ProteinDPO-identified mutations ~~compromise the antigenic properties required for vaccine efficacy~~* ***maintain key epitopes recognized by existing antibodies****, we measured the binding affinity of HA variants with nine substitutions to anti-HA broadly neutralizing antibodies (bnAbs) targeting either the head or the stem domains of HA via biolayer interferometry (BLI).*

And added an additional statement in the **Discussion** section:

*"Moreover, while pre-fusion stabilization and retention of binding to native antibodies are key components of vaccine design, further evidence would be needed to demonstrate antigenic properties required for vaccine efficacy"*

**Reviewer 1**

In this work, the authors introduce an approach called Direct Preference Optimization (DPO) to enhance desired properties of protein sequences generated by computational models. Specifically, they focus on protein stability using the inverse protein folding model ESM-IF1, and further optimize the model with protein mutational data. The authors report improvements in several benchmark tests compared with the original unsupervised ESM-IF1 and a fine-tuned supervised model, and in some cases, with another approach named ThermoMPNN. Finally, they apply the method to design hemagglutinin variants for influenza vaccines, reporting increased stability. The study presents an interesting strategy for optimizing deep learning models in protein design. However, I am not fully convinced that the proposed approach provides a significant advantage based on the current evidence. My major concerns are outlined below:

We thank the reviewer for recognizing the merits of our work, and include a point-by-point response below to address their major concerns of our method.

1. Lack of comparison in the HA application. While benchmark improvements are demonstrated, the application to HA design, arguably the most important example in this work, was not compared with other existing approaches. Since ESM-IF1 itself has shown some promising performance in similar tasks, it remains unclear how much Protein DPO actually improves upon it. A detailed comparison with alternative methods is necessary to convincingly establish the benefit of Protein DPO.

We thank the reviewer for raising this concern. On the task of HA stabilization, we now benchmark against ESM-IF1, SFT, ThermoMPNN, and ProteinMPNN in these models' ability to highly rank stabilizing mutations (1) from the literature and (2) out of the 30 single-mutations which were experimentally validated in our first revision, in part following the guidance of Reviewer 2. When comparing the ability of ESM-IF1, SFT, ThermoMPNN, ProteinMPNN and ProteinDPO to recover known HA-stabilizing mutations from the literature, specifically Milder et al [9] and Yassine et al [15] **(Fig. S5)**, we found that while some models were able to recover one mutation in their top-50, ProteinDPO has a higher recovery rate across all top-n levels.

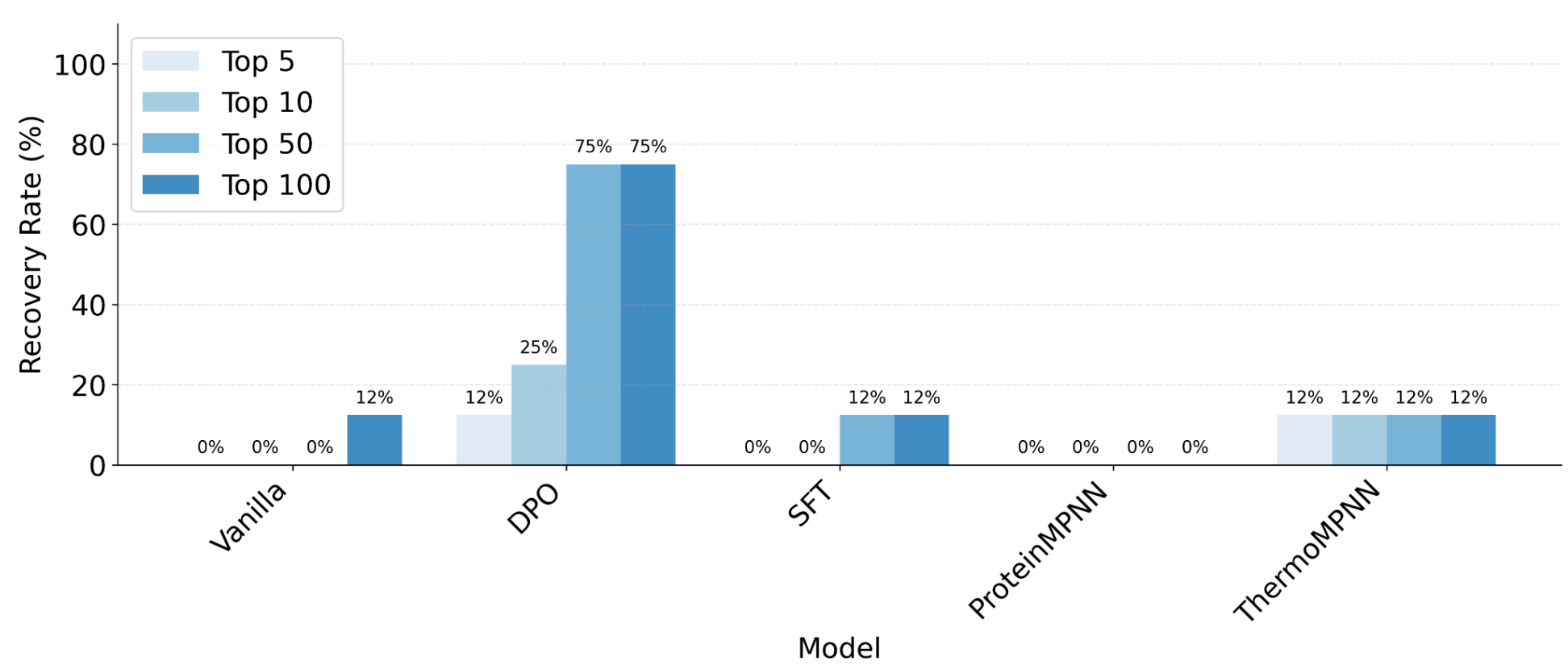

*Figure S5 | Recovery of literature-validated stabilizing mutations across protein design models. Bar plot showing the percentage of eight known stabilizing mutations (Milder et al. and Yassine et al.) recovered within the top-ranked predictions of each model. Models were evaluated using ensemble scoring across three PDB structures (6cf5, 6cfg, 2fk0). n=8 total literature mutations.*

Additionally, we compared the ability of ESM-IF1, SFT, ProteinMPNN and ThermoMPNN to rank the 6/30 highly-stabilizing (>= 9°C) mutations from the pool of 30 single-mutations we tested **(Fig. S5)**. Only the SFT model and ThermoMPNN were able to recover one of these mutations in its top-100.

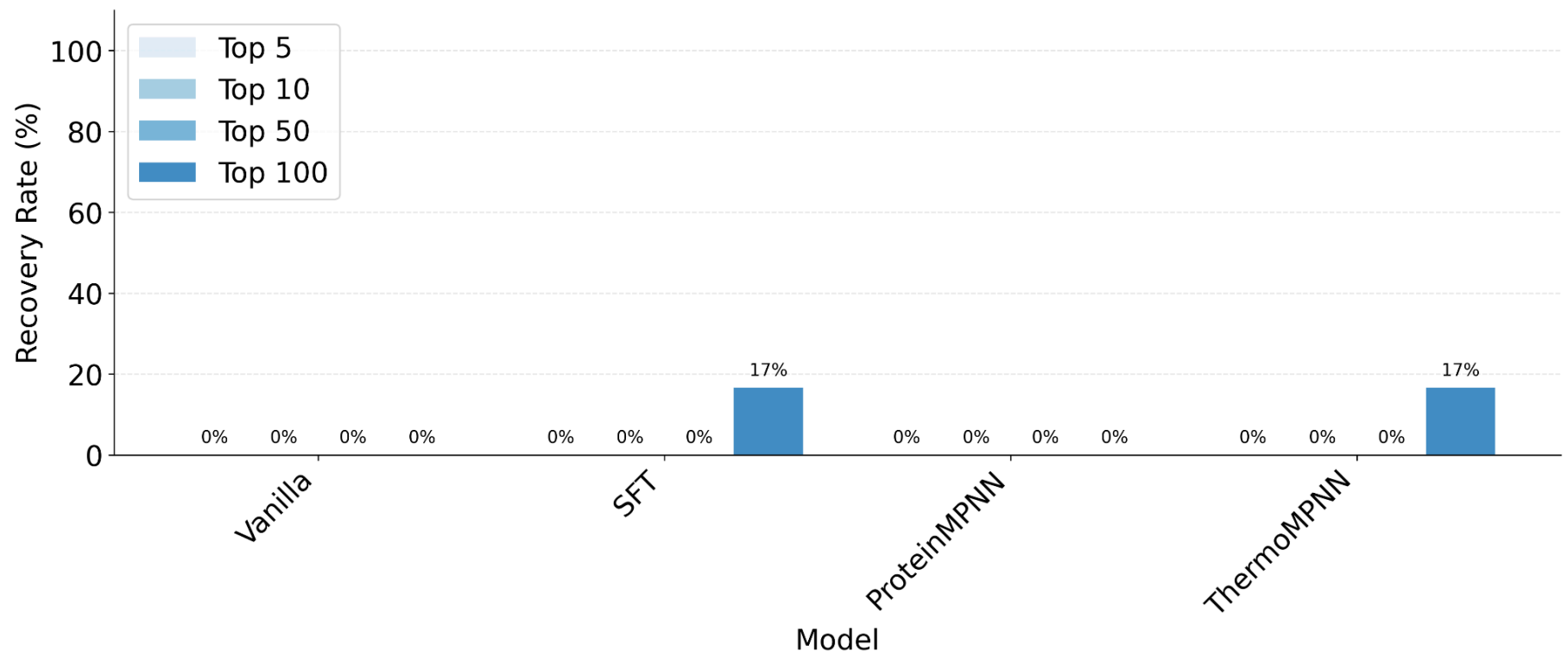


*Figure S6 | Recovery of experimentally tested DPO mutations by baseline models. Bar plot showing the percentage of six significantly stabilizing (>= 9◦C) DPO-identified mutations recovered within the top-ranked predictions of each model. Models were evaluated using ensemble scoring across three PDB structures (6cf5, 6cfg, 2fk0).*

Both analyses suggest that ProteinDPO is superior in highly ranking literature mutations, enabling low-n stabilization. Additionally the compared methods are not able to recover most of the best performing ProteinDPO mutations. We do note that a limitation of this test is that some of the highly ranked mutations by other methods may be stabilizing, which we also acknowledge in our text:

*'Only the SFT model and ThermoMPNN were able to recover one of these mutations in its top-100; however, it is possible top-ranked mutants from these baseline models are also stabilizing."*

2. Vaccine relevance not fully established. For vaccine design, the key requirement is demonstrating that designed variants elicit protective immune responses against the target virus. The manuscript shows recognition of designed variants by existing antibodies, but this does not sufficiently demonstrate antigenic properties required for vaccine efficacy. Stronger experimental validation would be needed to support claims of relevance to vaccine design.

We thank the reviewer for raising this concern. We agree that the results presented do not conclusively demonstrate vaccine efficacy. Considering the following guidance from the editor:

*"additional experiments to demonstrate that designed variants elicit protective immune responses against the target virus would be out of scope for us, so we recommend you tone down these claims"*

We toned down the implications of existing antibody recognition in the **Abstract**:

*"Leveraging up to nine substitutions, we achieved a 17°C improvement in melting temperature on a HA from human H5N1 influenza A virus while preserving ~~antigenicity~~* ***recognition by existing antibodies****, and up to 32°C stability improvements across recently emerged mammalian viral H5 HAs."*

as well as in the **Results** section:

*"To determine whether ProteinDPO-identified mutations ~~compromise the antigenic properties required for vaccine efficacy~~* ***maintain key epitopes recognized by existing antibodies****, we measured the binding affinity of HA variants with nine substitutions to anti-HA broadly neutralizing antibodies (bnAbs) targeting either the head or the stem domains of HA via biolayer interferometry (BLI).*

and added an additional statement in the **Discussion** section:

*"Moreover, while pre-fusion stabilization and retention of binding to native antibodies are key components of vaccine design, further evidence would be needed to demonstrate antigenic properties required for vaccine efficacy"*

3. Potential overemphasis on stability. The importance of stability may be overstated. For example, Figure 3D shows that the model generates sequences with improved stability according to Rosetta energy functions, but it is unclear whether these sequences are experimentally synthesized. Extremely stable sequences often risk aggregation due to excess hydrophobic residues, which may hinder practical expression and use. Moreover, the sequence design procedure is insufficiently described in the Methods. Was it simply initiated from random seeds, as in ESM-IF1?

We agree the Rosetta evaluation places an overemphasis on stability and needs clarification that these sequences were not experimentally tested. To address the former, we have added this clarification to the text:

*"Although Rosetta energy can be inaccurate and is not a substitute for experimental testing, it is a widely used independent evaluator of machine learning generations due to its reliance on physics-based parameters rather than shared training data with machine learning models [44, 5]"*

To address the latter, we added this clarification:

*"While these sequences were not experimentally verified to express, …."*

Additionally, we added a **Model sampling**, **Sampling evaluation**, and **Rosetta generation evaluation** sub-sections to the methods (**Methods 6.6**) to clearly describe the sampling procedure and evaluation for this experiment.

4. In Section 2.3, the claim that the method can rank "absolute stability of diverse antibodies" seems misleading, since only correlation coefficients are reported. These results reflect relative stability rankings, not absolute values. Furthermore, comparisons across antibodies likely rely on their shared fold and similar length. It is doubtful that the approach could be applied to rank melting temperatures across proteins of different folds.

We thank the reviewer for bringing up this concern. Being able to rank stability *within* a specific protein fold is what we intended to convey with this evaluation. We have made the following clarifications to the main text, only mentioning the term absolute when referring to ranking within a given fold:

*"we sought to evaluate if ProteinDPO can use whole-sequence reasoning to rank absolute stability of ~~diverse antibodies~~* ***sequences within a given fold, particularly antibodies"***

*"on the ability of vanilla ESM-IF1 in ranking the ~~absolute~~ differences in thermal melting points"*

*"ProteinDPO generalizes to scoring binding affinity of large complexes and ~~absolute antibody~~ thermal stability* ***of highly diverse and variable length antibodies"***

5. The Methods section needs clarification and expansion, particularly regarding benchmark evaluation. A separate subsection should clearly describe the metrics used (e.g., correlation coefficients), how they are computed, and the specific model outputs used for evaluation. In addition, figures such as 2B and 3B would benefit from showing distributional statistics rather than only averages, as mean values can obscure important variation.

We thank the reviewer for raising this concern regarding clarification in the methods section. To alleviate this we have added a sub-section, **Scoring metrics**, to the methods **(Methods 6.6)** to clearly describe the metrics used, including each of the coefficients. In this new text we point to the **Datasets** section **(Methods 6.5)** where one can find the specific model outputs used for evaluation.

Additionally, we added distributional plots for 2B and 3B **(Fig. S3, S4)** including for the newly benchmarked ProteinMPNN, and reference them in the main text:

*"Distributional plots of the individual values are included in the supplement* ***(Fig. S3)****."*

*"Distributional plots of the individual values for the AB-Bind dataset are included in the supplement* ***(Fig. S4)****."*

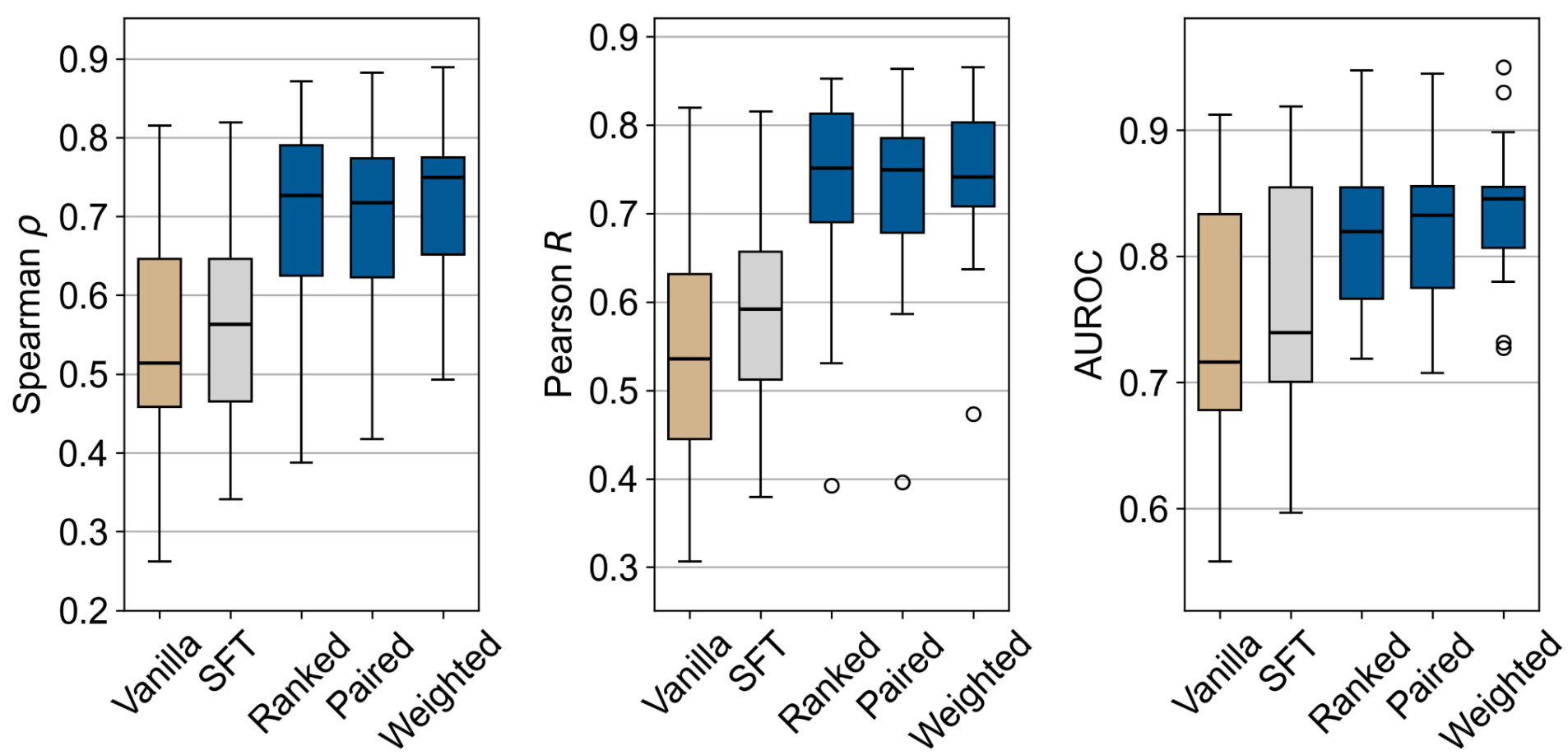


*Figure S3 | Distribution of performance across protein domains for predicting stability changes upon mutation (ΔΔG) in curated Megascale holdout dataset.*

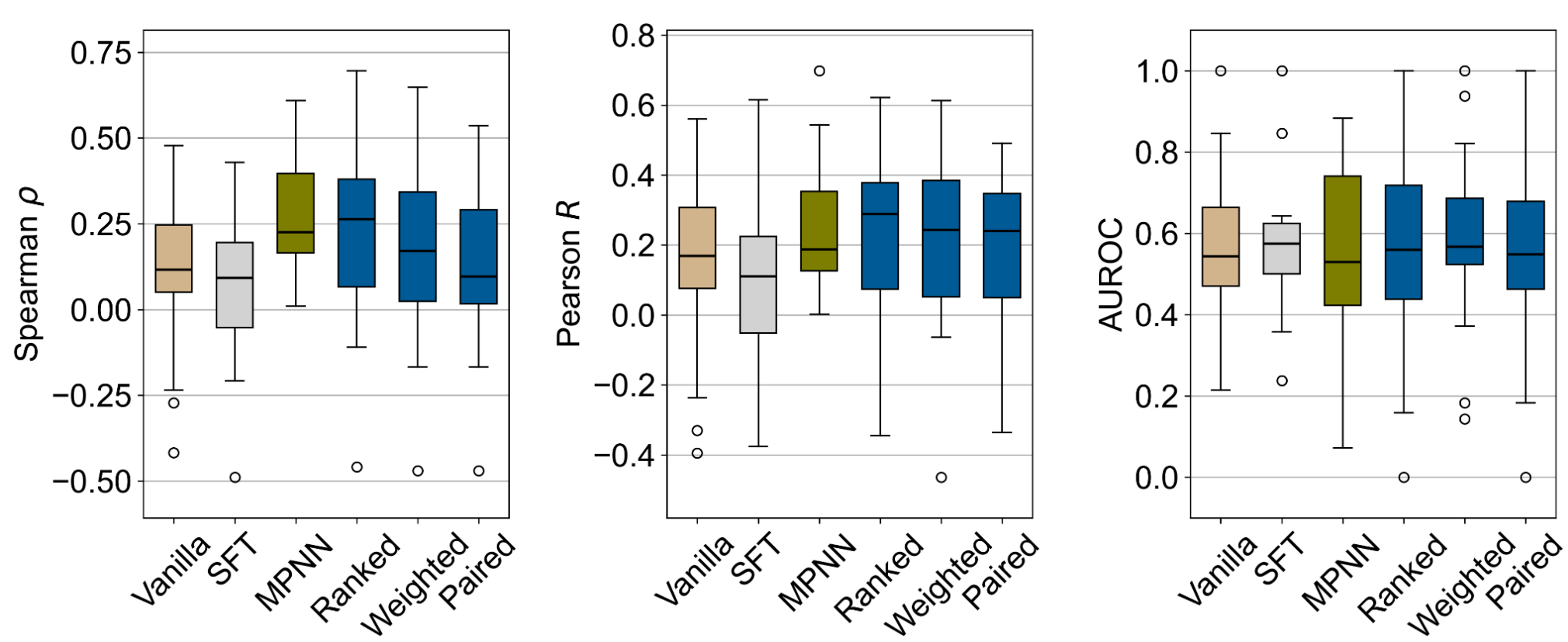

*Figure S4 | Distribution of performance across antibody-antigen complexes for predicting binding affinity changes upon mutation in AB-Bind dataset.*

6. The main contribution of this work lies in the proposed algorithm for optimizing ESM-IF1, but the Methods provide insufficient implementation details. Moreover, the GitHub repository only contains scripts and weights for inference, without the training code or implementation necessary for reproduction. At minimum, the authors should provide pseudocode for their approach—particularly for the weighted DPO algorithm, which is a novel aspect of this study.

We thank the reviewer for raising this concern. We have now included in-depth pseudocode for the approach. We have added algorithms for training the Paired, Ranked, and Weighted DPO models, as well as SFT training and log-likelihood scoring. All of these have been added to the **Model training** section in the Methods **(Methods 6.7)**. We have also added our training code for the model as **Supplementary Code**.

**Reviewer 2**

This paper applies the Direct Preference Optimization (DPO) approach to "align" the structure-conditioned protein language model ESM-IF with the objective of designing high stability protein sequences. The authors point out that the "base" model ESM-IF is trained to recreate the probability distribution of the training data, which does not match the common objective in protein design to produce high stability proteins. The DPO method improves the alignment between the distribution sampled by the model and the author's (and community's) design objective of sampling high stability proteins. The resulting model (ProteinDPO) can be used for both scoring structures and for generating new sequences based on an input protein backbone. The authors examine several variants of ProteinDPO, computationally benchmark ProteinDPO on several design and ranking tasks against other leading models, and also apply ProteinDPO to experimentally design vaccine antigens with higher stability.

The main strengths of the paper are:

-The problem of improving generative AI models for protein design is timely and important and the approach of using DPO based on experimental folding stability data is relatively novel.

-The flexiblity of ProteinDPO enables the model to be applied to score multi-mutation variants as well as entire unrelated structures, which is rare in the field of stability prediction. The model achieves superior performance over a naive application of ThermoMPNN at predicting effects of multiple mutants (Fig. 2C). The model also shows a modest correlation with antibody binding and melting temperature (Fig 3B/C), which is superior to ESM-IF ("Vanilla") and cannot achieved using models like ThermoMPNN or MAESTRO that are designed to score single mutants.

-For predicting single mutants, several variants of ProteinDPO have strong performance at the S669 benchmark and come close to matching ThermoMPNN at the Fireprot benchmark.

-Using a computational benchmark, the authors show that ProteinDPO-based protein design improves on ESM-IF based protein design according to the Rosetta energy functon(Fig. 3D).

-The experimental work demonstrates a successful application of ProteinDPO. However, this work is seemingly not intended to illustrate the superiority of ProteinDPO over other methods- the proposed mutants are not computationally evaluated using any method other than ProteinDPO.

We thank the reviewer for appreciating and highlighting the strengths of our work, particularly the need for improving protein generative models via alignment, the flexibility of the resulting model for stability prediction, and the successful experimental application.

The main weaknesses of the paper are:

1. Although the authors make the case that ProteinDPO has unique applications beyond other stability models (such as Fig. 2C, Fig 2B, Fig 3C), the experimental demonstration doesn't really highlight these applications. Instead, it illustrates an application where other stability models might have performed equally well or better.

We thank the reviewer for bringing up this concern. While we were interested in addressing these unique applications (binder design, backbone stabilization, antibody stabilization), in efforts of balancing model validation and finding a relevant practical application, we decided hemagglutinin stabilization was an especially interesting choice. We also note that ProteinDPO readily generalizes to multi-mutation scoring and note that this was tested in our experimental work **(Fig. 2C)**. While several substitutions in the multi-mutation variants were derived from single-mutants verified as stable from the initial round of 20 mutations (labeled DPO-A in **Table S6**), for many of the 5-substitution and 9-substitution variants, ProteinDPO included substitutions that were never previously evaluated as singletons, instead using its likelihood function to evaluate a combined score and demonstrating multi-mutation variant generation. We provide further detail on the multi-mutation selection protocol in a section titled **Mutation selection** in the Methods section **(Methods 6.8)**. Additionally, the HA structure is a homo-trimeric complex, and explicit scoring of multi-chain complexes is inaccessible to many stability models, including ThermoMPNN.

2. The authors don't examine the performance of other stability models at their experimental demonstration. It would be very useful to the audience to know whether the output of ProteinDPO is unique or similar to predictions made by other stability models. At minimum, the authors should take the top 30 mutants designed by ProteinDPO (the ones that were experimentally tested), examine how these mutants score according to "vanilla" ESM-IF and ThermoMPNN, and report (1) whether the ProteinDPO-designed mutants are predicted to be favorable according to other methods, (2) whether they also rank near the top of all mutants evaluated by other methods, and (3) whether other methods show an ability rank mutants by Tm within the top 30 designed by ProteinDPO. It would also be good to note whether ProteinDPO itself can rank these 30 mutants, although this is a somewhat unfair task since they are all already highly favorable according to ProteinDPO.

We are grateful for the set of recommended tasks, which we have performed and were also helpful in responding to a question from Reviewer 1. For HA stabilization, we benchmarked

against ESM-IF1, SFT, ThermoMPNN, and ProteinMPNN in these models' ability to highly rank stabilizing mutations (1) from the literature and (2) out of the 30 single-mutations which were experimentally validated in our first revision. For known HA-stabilizing mutations from the literature, specifically from Milder et al [9] and Yassine et al [15] **(Fig. S5)**, ProteinDPO has a much higher recovery rate across all top-n levels.

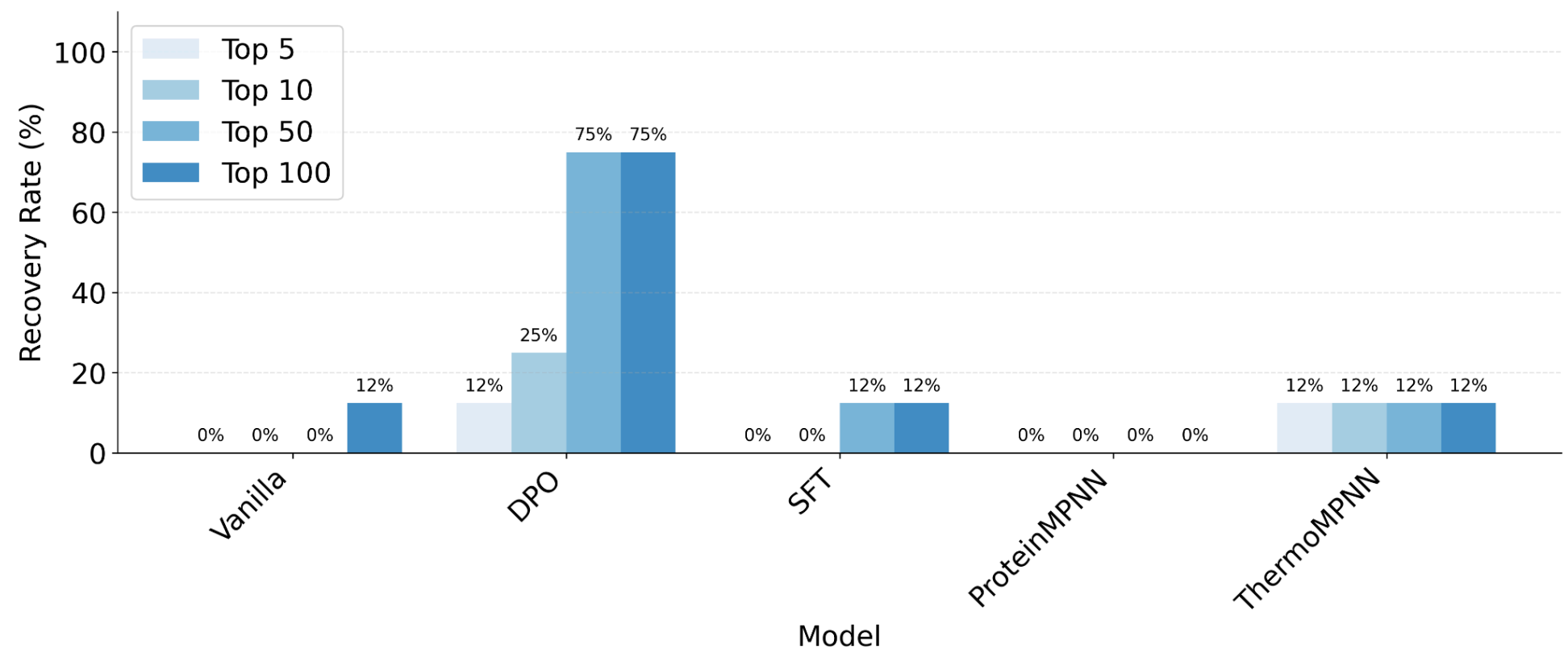


*Figure S5 | Recovery of literature-validated stabilizing mutations across protein design models. Bar plot showing the percentage of eight known stabilizing mutations (Milder et al. and Yassine et al.) recovered within the top-ranked predictions of each model. Models were evaluated using ensemble scoring across three PDB structures (6cf5, 6cfg, 2fk0). n=8 total literature mutations.*

Additionally, we compared the ability of ESM-IF1, SFT, ProteinMPNN and ThermoMPNN to rank the 6/30 highly-stabilizing (>= 9°C) mutations from the pool of 30 single-mutations we tested **(Fig. S5)**. Only the SFT model and ThermoMPNN were able to recover one of these mutations in its top-100.

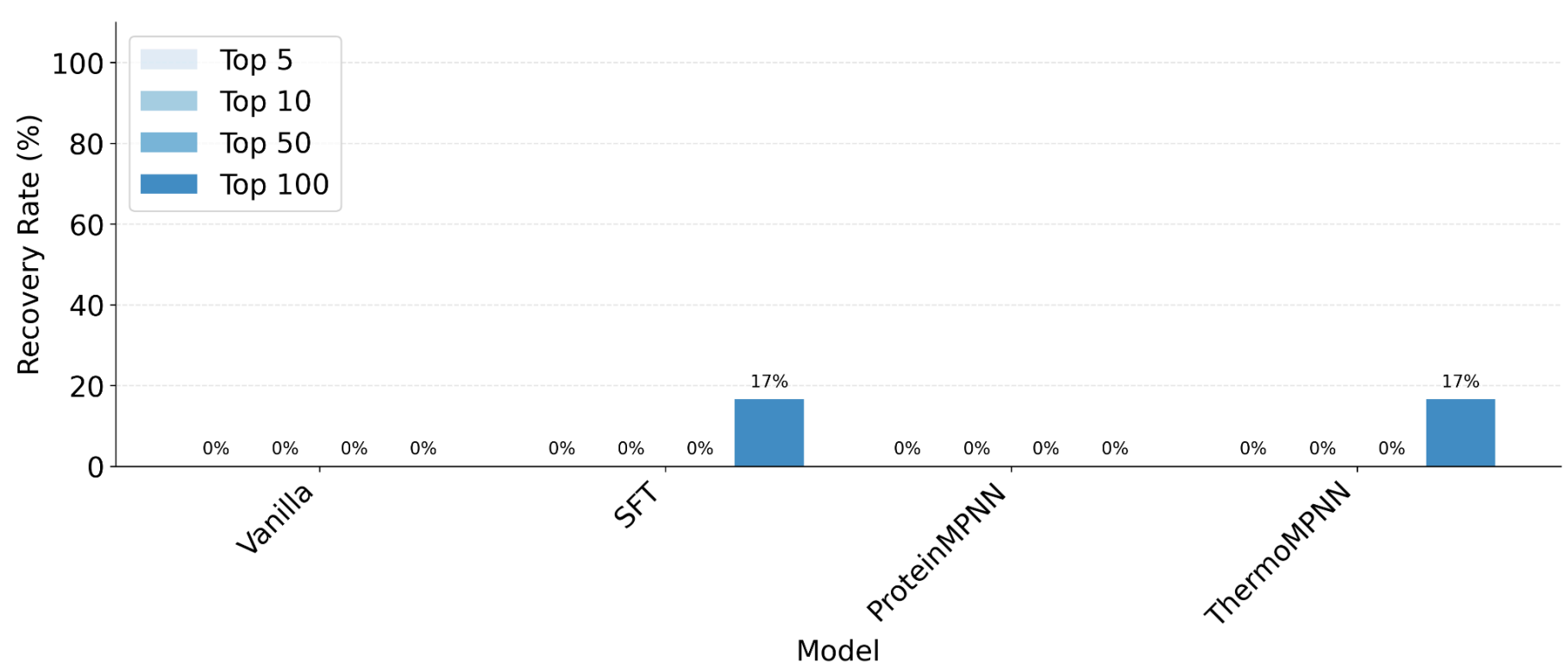


*Figure S6 | Recovery of experimentally tested DPO mutations by baseline models. Bar plot showing the percentage of six significantly stabilizing (>= 9◦C) DPO-identified mutations recovered within the top-ranked predictions of each model. Models were evaluated using ensemble scoring across three PDB structures (6cf5, 6cfg, 2fk0).*

Both analyses suggest that ProteinDPO is superior in highly ranking literature mutations within its top-n mutations, enabling low-n stabilization. We do note that a limitation of this test is that some of the highly ranked mutations by other methods may be stabilizing, which we also acknowledge in our text:

*'Only the SFT model and ThermoMPNN were able to recover one of these mutations in its top-100; however, it is possible top-ranked mutants from these baseline models are also stabilizing."*

We also conducted the third analysis that evaluates "whether other methods show an ability to rank mutants by Tm within the top 30 designed by ProteinDPO." As an important caveat, as expected, ProteinDPO cannot rank its own top-30 mutants, as these are all extremely high-likelihood mutations coming from the top ~0.3% of all possible mutations. The vanilla model, SFT and ProteinMPNN show modest ability to do so, with ThermoMPNN, being the only model trained on stability prediction, performing the best. We include the results of this analysis in **Fig. S7**:

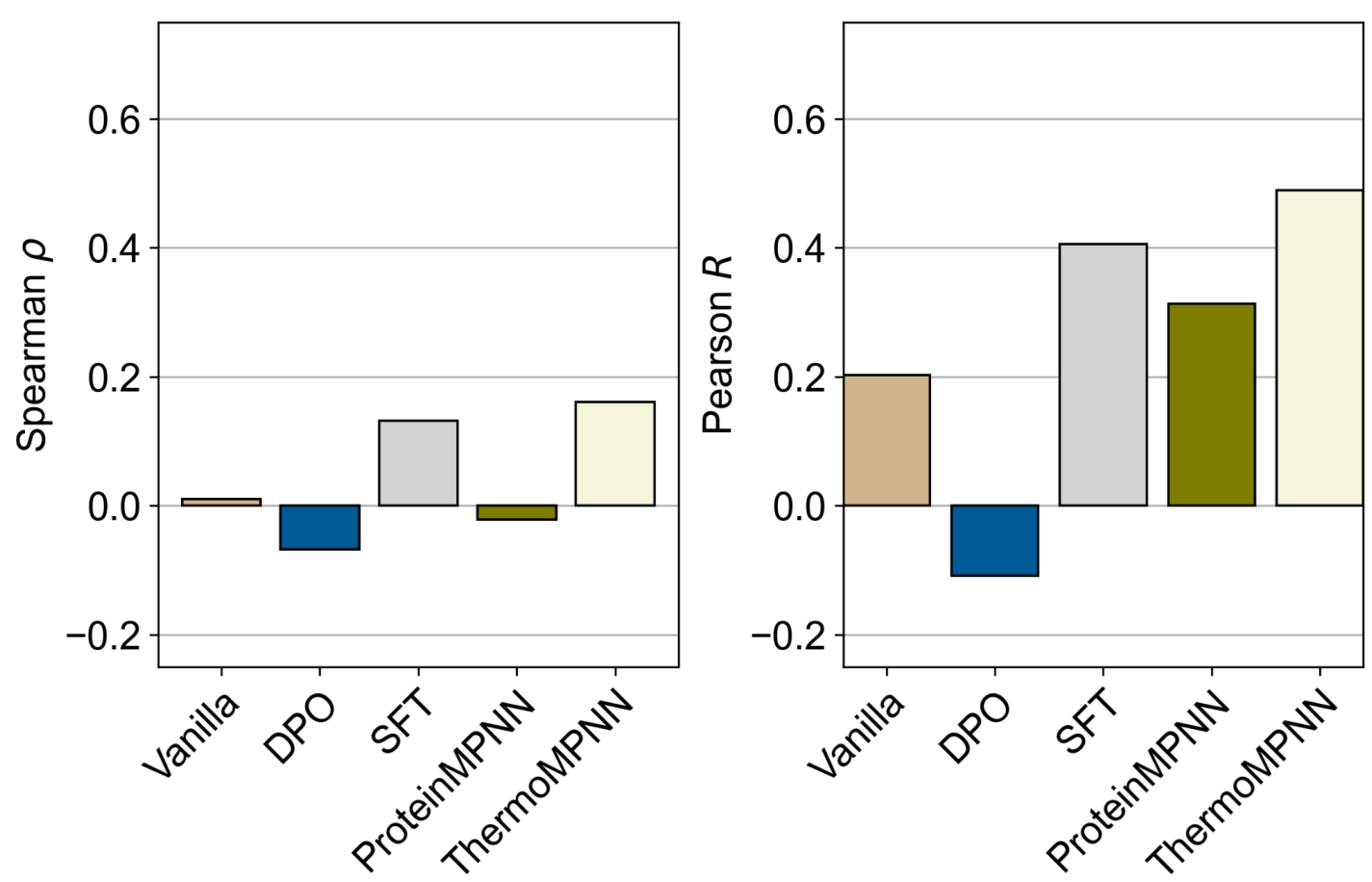


*Figure S7 | Comparison of methods in ability rank the 30 experimentally tested single-substitution variants identified by ProteinDPO, measured by Pearson R and Spearman ρ correlation coefficients between model scores and temperature of thermal unfolding (Tm).*

We also refer to these results in the main text:

*The SFT model, ProteinMPNN and ThermoMPNN do however show modest ability to rank the thermal stability (Tm) of these 30 variants **(Fig. S6)**.*

3. The benchmarking of ProteinDPO against other models for antibody melting temperature and protein-protein binding is somewhat minimal. Only ESM-IF is used as a benchmark. Are any

other benchmarks available? At minimum, RosettaREU (total energy, energy per residue, or ddG) could be used, as in Fig 3D.

We thank the reviewer for pointing out the need for additional benchmarking, and for recommending methods to utilize. To address this concern, we evaluated RosettaREU on the antibody thermal stability benchmark, finding that using total energy, rather than energy per residue is a superior metric. We have added this benchmark to the main text **(Fig. 3C).**

For the protein-protein binding benchmark, despite extensive effort, we found it very challenging and computationally intensive to use Rosetta for protein-protein binding evaluation. Naively relaxing the complexes with the mutations applied and extracting a ddG by difference in energy yielded very poor results (and resulted in several PDB and mutation processing errors); we then attempted to use the recommended flex_ddg protocol, which yielded similar errors and would also have taken an estimated 500,000 CPU hours for the ~8500 mutations in SKEMPIv2 and AB-Bind under recommended settings, which was not feasible for us to run in a reasonable timeline. Instead, we have added ProteinMPNN as another baseline method for the protein-protein binding benchmark (**Fig. 3B)** [4], given it is a widely used tool for binder design [1, 11] and the comments by Reviewer 3 asking us to use ProteinMPNN as a benchmark comparison. Our updated **Fig. 3** is provided below:

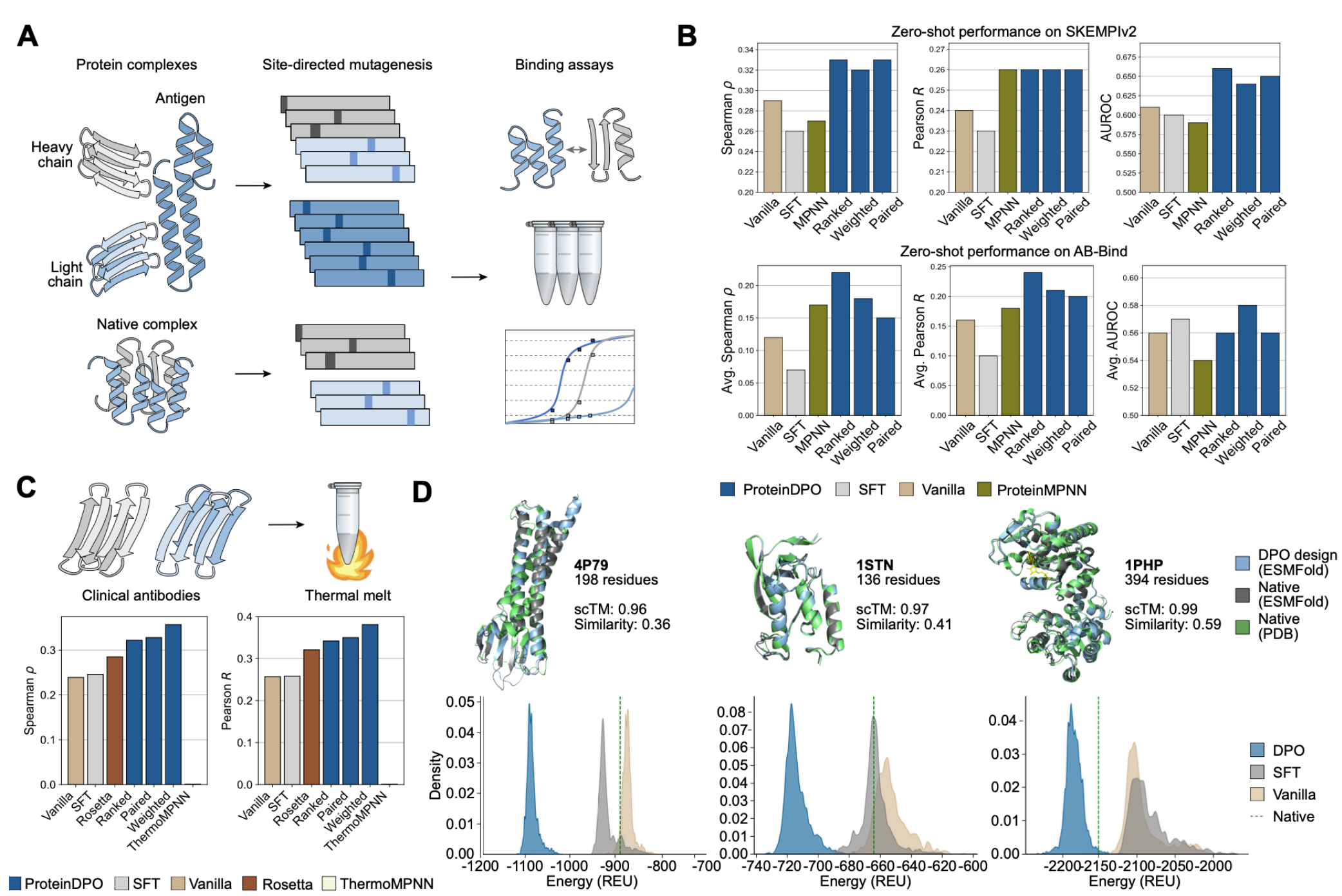


4. The authors need to be careful about how they describe the overall accuracy of the ProteinDPO method. For example, in the 4th paragraph of the discussion, the authors write

"ProteinDPO also generalizes to a greater variety of tasks than existing supervised models, being able to predict ΔΔG, binding affinity, and thermal melting across diverse wild-type proteins, multi-chain antibodies, and protein complexes." This ability to "predict binding affinity" is based on spearman rho values of 0.2 - 0.33 - poor overall. The ability to predict thermal melting is based on spearman rho of ~0.35 - again poor. It would be accurate to say that the ProteinDPO model can predict these quantities ***more accurately than the vanilla ESM-IF model***, which is a worthwhile computational achievement. But the authors need to avoid unqualified statements like saying "ProteinDPO can predict X".

We thank the reviewer for raising this concern and agree with these comments. For the noted cases, we have described these results as "modest":

*"ProteinDPO has modestly increased scoring of the binding affinity of large protein-protein complexes"*

And have also revised several statements in the text:

*"Notably, the aligned model also performs well in domains beyond its training data to enable stabilization and **improved** binding affinity prediction of large multi-chain proteins."*

*"and thermal melting across diverse wild-type proteins, multi-chain antibodies, and protein complexes **more accurately than the unaligned ESM-IF1 model."***

***"**ProteinDPO generalizes to **improved** ~~scoring~~ binding affinity **scoring** of large complexes and thermal stability of highly diverse and variable length antibodies**,"***

5a. It's not correct to say that FireProt and S669 are made from deep mutational scanning datasets since these are generally piecemeal individual measurements, not multiplexed comprehensive measurements.

Thank you for this comment. We have made this clarification in the text.

*"We benchmarked models using ~~deep mutational scanning (DMS)~~ **experimental protein stability** dataset FireProt and S669. **These datasets are compiled from small-scale** c~~ompiled from~~ mutagenesis experiments across diverse monomeric proteins, in which certain residues are mutated, variants are expressed, and their stability is measured experimentally."*

*"These datasets are compilations of experimental stability measurements ~~of mutations~~ derived from several **small-scale** mutagenesis experiments of native proteins"*

5b. Should lower right panel be AUROC?

Yes, it should, we have fixed this error. Thank you for pointing this out.

5c. Fig 4B: It seems like the best single mutant is as good as the best combination of mutants? Is this correct? Does this mean that combining mutants was unsuccessful? Or is the light blue point at the top not a single mutant? A different coloring scheme would be beneficial. The authors should be more explicit in the text about the best single mutant.

Yes, it is correct that the best single mutant is similar to the best combination of mutants, and have added a note of this to the main text. In the text, we do not make strong claims about the multi-mutation variants selected by ProteinDPO having higher stability than could be derived by additivity of the single-mutants. However, we do highlight that the five sets of ProteinDPO-identified nine-mutants are well tolerated by the structure and generally stabilizing.

*"While the best nine-substitution variant did not significantly surpass the best single-substitution variant, nine-substitution variants were 9.8 °C more stable on average than single-substitution variants."*

We give full details on the sequence and stability of all mutants in the Supplementary **(Supp. Table S6)**, to be more explicit we have added a note in the main text to refer to this table.

*"All variants and their associated melting temperatures are reported in the supplement (Table S6)"*

We have also adjusted the color scheme for a better distinction between the number of mutations.

5d. The CD wave scans look unusual- why is the signal so much weaker in DPO-19 and WT compared to DPO-4? If the proteins are folded and have the same structure, they should show the same spectrum. Is it possible the protein concentrations were measured incorrectly for all proteins except DPO-4? The methods should mention how protein concentrations were measured. A folded alpha-beta protein should have a signal with the intensity of the signal for DPO-4 (see a reference on this)- the other signals are strangely weak.

Yes, this was likely due to concentration differences. When collecting the CD wave scans, concentration was measured using a NanoDrop spectrophotometer and ensured to be in the range of 0.1-0.3 mg/ml as this was the guidance for using this particular CD spectrometer. It appears this window was too large between samples. We have updated the methods regarding how concentration was measured:

*"Protein samples* ***were*** *diluted or concentrated to ~~approximately 0.2 mg/ml~~* ***concentration within the range of 0.1mg/ml to 0.3mg/ml*** *in PBS at 20°C.* ***Concentration was calculated using a Thermo Scientific NanoDrop OneC Microvolume Spectrophotometer measuring for absorbance at 280nm."***

and added a disclaimer to the main text.

*"Differences in absorbance signal magnitude are due to concentration differences between samples* ***(Methods)"***

5e. Methods: The authors should state which version of the Megascale dataset they used (v1 12/6/2022 or v2 4/20/2023).

We have added a statement of the version of the dataset used.

*"We used the Megascale dataset for training, either with our own training splits* ***(derived from v2 4/20/2023)****, those from Diekhaus et al, or those from Cuturello et al depending on subsequent evaluation."*

*"The Megascale dataset* ***(v2 4/20/2023)*** *served as the experimentally measured stability dataset from which we aligned the pretrained ESM-IF1."*

**Reviewer 3**

This work by Widatalla et al applies the DPO method to protein stability engineering problem. Protein stability engineering is clearly an important research subject, and the application of DPO technique to this problem is somewhat novel. There were several works in which protein language models were fine-tuned for the purpose, but to my knowledge, the application of DPO seems the first. I think validating their method through actual experiments is very encouraging and valuable; however, two major concerns listed below make it hard to fully appreciate the quality of the work.

We thank the reviewer for recognizing the importance of protein stabilization, the novelty of our approach, and our experimental validation. We are also grateful for the questions and recommended analyses, which have improved the manuscript.

1. The first is the basic philisophy of the work. From a fundamental point of view, is "tuning a pre-trained model" a relevant philosophy for the protein stability estimation? Regarding the complexity of protein folding and stability, I think this idea will only give a marginal improvement over existing methods sharing the same concept; i.e. another (slightly better) ThermoMPNN. A good work to compare is the recently published BioEmu, in which the authors simulated protein folding to approximate the equilibrium. I cannot see such conceptual novelty in this work that tackles the inherent challenge of the problem. Introducing DPO technique may add little novelty in this regard.

We thank the reviewer for raising the concerns regarding "tuning a pre-trained model" and its relevance to stability prediction. While protein folding stability is a complex phenomenon, additional training beyond pre-training is an effective approach, as several state-of-the-art models (e.g., ThermoMPNN and MSAesm_ddG [5, 3]), are fine-tuned pretrained language models. Moreover, BioEmu [8] gains its stability prediction through additional fine-tuning; after its pretraining task of learning to generate protein conformations, similarly to ProteinDPO, it undergoes a stability fine-tuning stage on the Megascale dataset [8].

Regarding BioEmu, we note that ProteinDPO achieves a higher Spearman correlation (~0.7) than BioEMu (~0.6) in regards to stability prediction as reported in the section **Predicting protein stabilities** in our manuscript and Fig. 4 in the BioEmu manuscript [8] (though we note that ProteinDPO and BioEmu used slightly different Megascale train and test splits). Importantly, while BioEmu showed its simulations recapitulated folded versus disordered states from proteins in the CALVADOS and ProthermDB databases, Importantly, BioEmu did not show generalizability of stability (ddG) prediction outside of the Megascale dataset, while we show ProteinDPO generalizes in stability prediction to FireProt and S669 **(Fig. 2D, 2E)**.

Regarding the comparison to ThermoMPNN, ProteinDPO is inherently different to ThermoMPNN and other ddG prediction models. ProteinDPO remains a generative model; instead of predicting a scalar value given a mutation (usually limited to only a single-substitution for most models), ProteinDPO produces a probability distribution of amino acids (biased towards stable sequences via DPO) given an input backbone, from which one can sample to generate an entire sequence. While we use comparisons to stability predictors to measure the optimization performance, ProteinDPO is fundamentally different as it remains a generative model. Remaining a generative model allows ProteinDPO to naturally score multi-mutation variants in-silico, as well as generate them in the lab. Furthermore, the use of structure-conditioned language models to design large portions of protein sequences given a backbone has been successful in many design applications spanning design protein binders, enzymes and membrane proteins [1, 4, 6, 12, 14]. ProteinDPO now enables structure-conditioned sequence design that is biased towards even greater stability, a desired attribute in many design applications.

2. The method is repeatedly compared against vanilla or STF, but I don't think this is a valuable comparison. This may show "how bad ESM-if" rather than "how good ProteinDPO" is for the problem. Also, it is hard to believe SFT in the manuscript was trained by their best. Therefore, it is worth testing just once for ablation, and the main comparison should be against other methods like ThermoMPNN. Also, the authors keep argue "method solely trained on small proteins generalizes to bigger protein". This is not an advantage but a prerequisite; methods not satisfying such property should be less appreciated.

Comparison to other methods is not very strong, but just based on the current figures, it is hard to identify clear advantages of the method. This marginal (or neglible) improvement should be related to the inherent limitation of the approach as pointed out in point 1.

Thank you for these comments. We compared ProteinDPO to ESM-IF1 (and SFT) across all evaluations since this is the model we initialize the weights with, and allows for specific testing of the hypothesis of whether DPO training improves scoring and generation performance. Comparing ProteinDPO to the vanilla ESM-IF1 and an SFTed model ensures we can controllably isolate the effect of DPO training, as the pretraining data, initial weights, parameter count, and featurization are the same between the three models. With regards to concerns that the results primarily reflect “how bad ESM-IF is”, we note that ESM-IF1 is generally considered a strong base model, for example having been used experimentally for enriching binding affinity in Shanker et al. [13]. Thus we see improving upon it as a valuable contribution.

Additionally, supervised fine-tuning is a good baseline methodology as it is widely used in almost all state-of-the-art language models for natural text [11] and which has shown success experimentally for biological applications [6, 12, 10, 7].

In regards to concerns that SFT was not trained properly, we have added additional training details to the **Methods** section **(Supervised Finetuning)**, and included loss curves of the learning rate sweeps that were conducted to find an optimal checkpoint in the supplemental **(Fig. S2)**. Across different SFT training runs, we find that all checkpoints perform similarly:

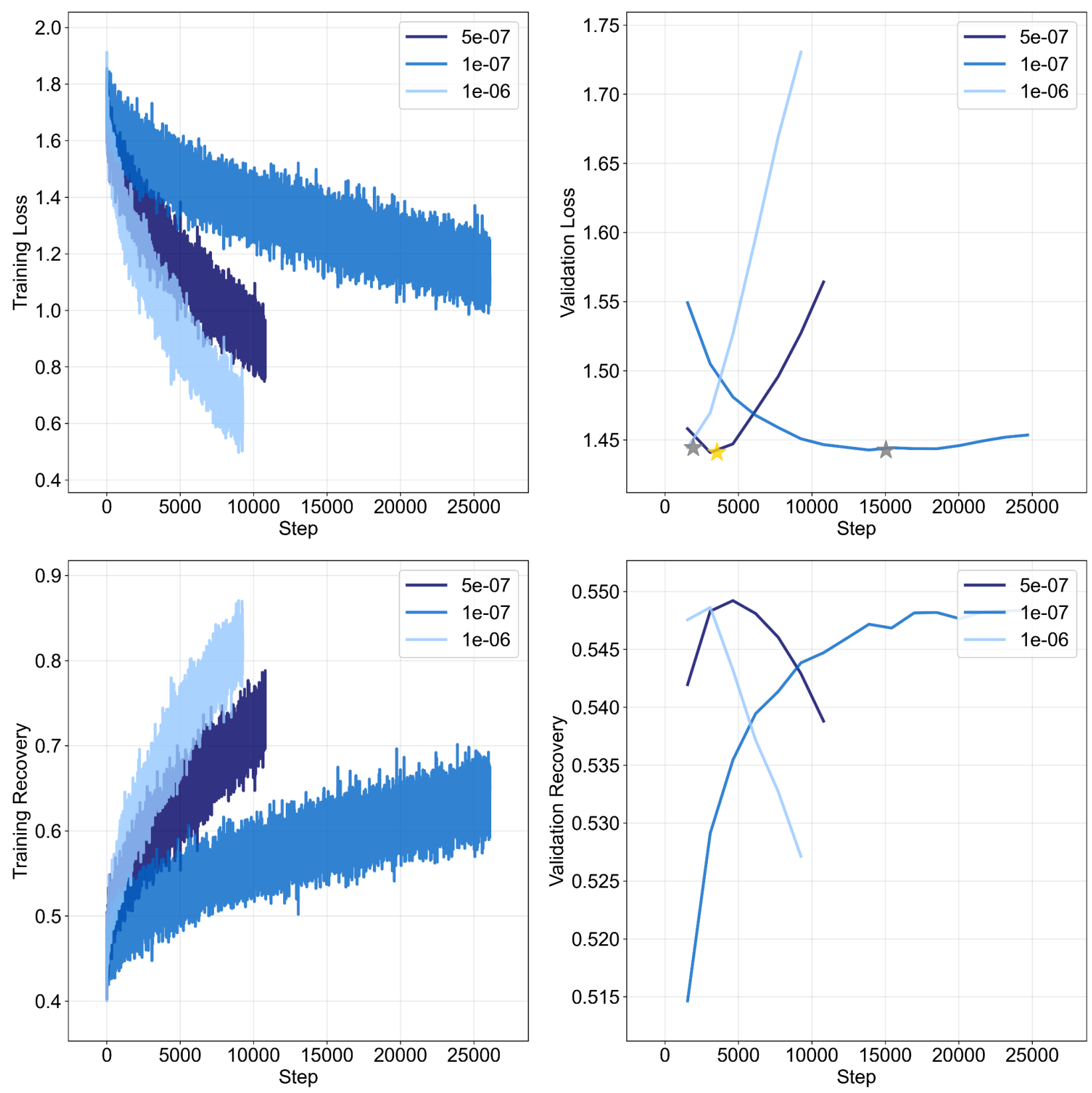


*Figure S2 | Training and validation loss and sequence recovery curves for training the supervised finetuned model. The selected checkpoint was the checkpoint which best minimized validation loss across all runs, and is denoted with a star. Checkpoints which minimized validation loss for other runs, are denoted with a gray star, and are compared against the selected checkpoint in* ***Table S7.***

| Learning rate | Megascale (mean) | FireProt | AB-Bind (mean) | Antibody thermal stability |
|---|---|---|---|---|
| 1e-7 | 0.584/0.561 | 0.430/0.441 | 0.10/0.05 | 0.250/0.236 |
| 5e-7 | 0.588/0.570 | 0.415/0.429 | 0.10/0.06 | 0.258/0.246 |
| 1e-6 | 0.577/0.556 | 0.422/0.433 | 0.10/0.04 | 0.249/0.235 |

*Table S7 | Comparison of different SFT checkpoints trained with varying learning rate on stability and binding prediction in terms of (mean) Pearson R / Spearman ρ correlation coefficients.*

Regarding ThermoMPNN, we benchmarked this model across several datasets (MegaScale single-mutants, Megascale double-mutants, S669, and Fireprot). However, we could not benchmark against ThermoMPNN for SKEMPI, AB-Bind or Antibody thermostability due to the inability of ThermoMPNN to score complexes or absolute differences in highly diverse sequences (e.g., those with different length). In lieu of ThermoMPNN, we have added benchmarking of ProteinMPNN and Rosetta, for protein-protein binding and antibody thermostability.

We also agree with the reviewer that generalization of a model to larger proteins is a "prerequisite, not advantage" for any model that ought to be used to predict protein stability. Notably, many previous machine learning models have had a tendency to overfit on their respective domain and to fail to generalize to larger proteins. A recent work featuring a Megascale-fine-tuned version of the ESM protein language model experienced this; despite performing well on prediction for a MegaScale test set, this model did not generalize to other DMS datasets, where the authors of that study cited a mismatch in the distribution of sequence lengths [2].

Lastly, we agree with the reviewer that for certain tasks, such as protein-protein binding, and antibody-antigen binding, the improvements from the base model are modest. However, for stability prediction, our performance improvements are indeed meaningful. Specifically, we see large improvements in stability prediction in the Megascale holdout set (Pearson $R$ and Spearman $\varrho$ improvements of 0.16 to 0.19), and the S669 and FireProt datasets (Pearson $R$ and Spearman $\varrho$ increases of 0.13 to 0.16). These improvements generalize to ranking absolute stability of diverse antibodies Pearson $R$ and Spearman $\varrho$ improvements from 0.08 to 0.12). Additionally, in **Figure 3D**, we see how these differences in scoring translate to a clear difference in the predicted stability of full sequence generations when evaluated by an orthogonal physics-based approach, Rosetta.

3. This is a suggestion for the presentation of the experimental section. As I mentioned above, experimental validation is very encouraging. At the same time, readers may wonder what if other already-established method was tested in parallel. For example, ProteinMPNN already demonstrated its ability to improve stability in various previous works other methods (e.g. Rosetta) failed. It should have been much more impactful if ProteinDPO have proved experimetnal success in a similar way (although a relevant baseline now has largely shifted from Rosetta to ProteinMPNN since then). Otherwise readers may have impression that results presented were cherry-picked.

We are grateful for this comment, which is similar to comments from the two other reviewers. On the task of HA stabilization, we now benchmark against ESM-IF1, SFT, ThermoMPNN, and ProteinMPNN in these models' ability to highly rank stabilizing mutations (1) from the literature and (2) out of the 30 single-mutations which were experimentally validated in our first revision, in part following the guidance of Reviewer 2. When comparing the ability of ESM-IF1, SFT, ThermoMPNN, ProteinMPNN and ProteinDPO to recover known HA-stabilizing mutations from the literature, specifically Milder et al [9] and Yassine et al [15] **(Fig. S5)**, we found that while some models were able to recover one mutation in their top-50, ProteinDPO has a higher recovery rate across all top-n levels.

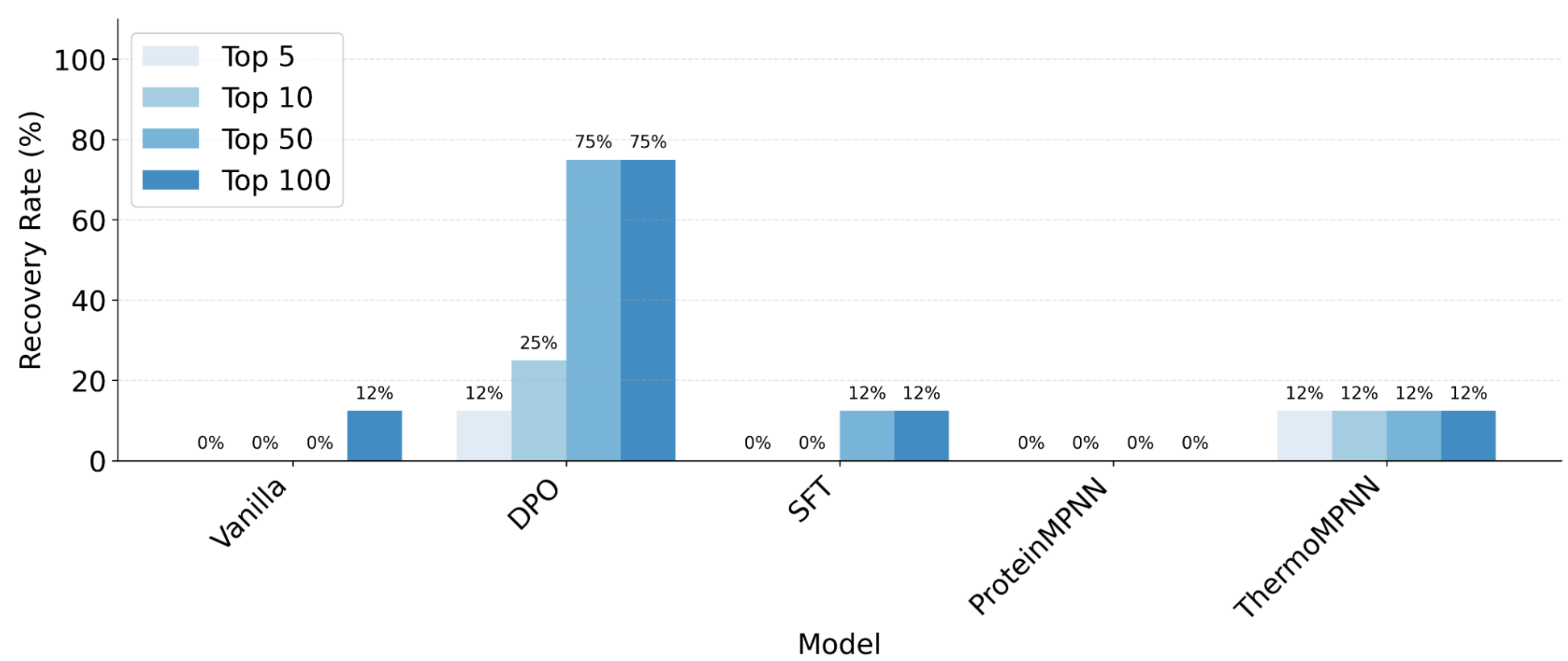


*Figure S5 | Recovery of literature-validated stabilizing mutations across protein design models. Bar plot showing the percentage of eight known stabilizing mutations (Milder et al. and Yassine et al.) recovered within the top-ranked predictions of each model. Models were evaluated using ensemble scoring across three PDB structures (6cf5, 6cfg, 2fk0). n=8 total literature mutations.*

Additionally, we compared the ability of ESM-IF1, SFT, ProteinMPNN and ThermoMPNN to rank the 6/30 highly-stabilizing (>= 9°C) mutations from the pool of 30 single-mutations we tested **(Fig. S6)**. Only the SFT model and ThermoMPNN were able to recover one of these mutations in its top-100.

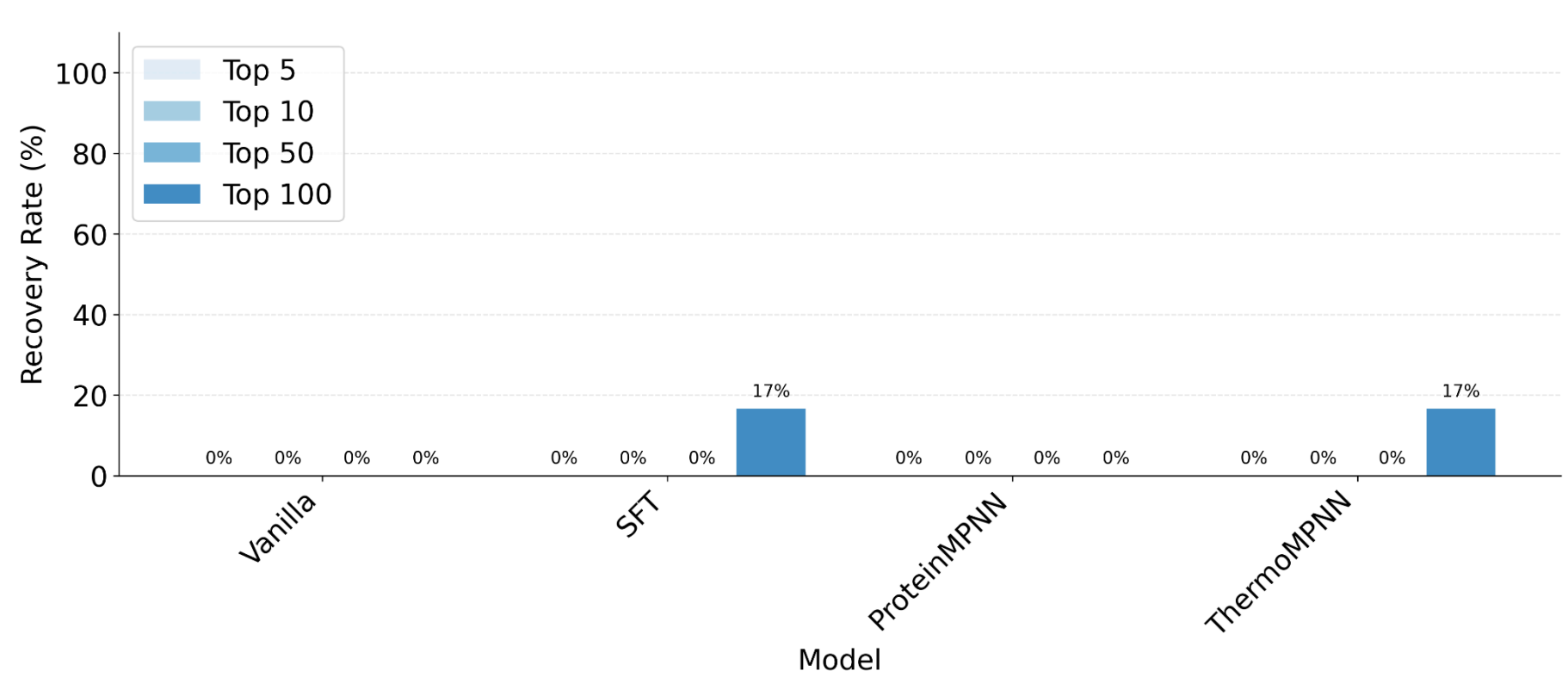

*Figure S6 | Recovery of experimentally tested DPO mutations by baseline models. Bar plot showing the percentage of six significantly stabilizing (>= 9◦C) DPO-identified mutations recovered within the top-ranked predictions of each model. Models were evaluated using ensemble scoring across three PDB structures (6cf5, 6cfg, 2fk0).*

Both analyses suggest that ProteinDPO is superior in highly ranking literature mutations, enabling low-n stabilization. Additionally the compared methods are not able to recover most of the best performing ProteinDPO mutations. We do note that a limitation of this test is that some of the highly ranked mutations by other methods may be stabilizing, which we also acknowledge in our text:

*'Only the SFT model and ThermoMPNN were able to recover one of these mutations in its top-100; however, it is possible top-ranked mutants from these baseline models are also stabilizing."*

4. I was not able to install the code with the error message below:

"Solving environment: failed

ResolvePackageNotFound:

- python[version='3.10.*,==3.9.18',build=h955ad1f_0]"

I do see this issue was posted in the github a bit ago, but was not fixed. Please fix this issue before your resubmission. The inference code itself is very simple and straightforward.

Thank you for bringing this to our attention, the installation issue has been fixed in commit #2ef299f.

## References


1. Bennett, N. R. *et al.* Atomically accurate de novo design of antibodies with RFdiffusion. *Nature* (2025). https://doi.org/10.1038/s41586-025-09721-5

2. Chu, S. K. S., Narang, K. & Siegel, J. B. Protein stability prediction by fine-tuning a protein language model on a mega-scale dataset. *PLOS Comput. Biol.* **20**, e1012248 (2024). https://doi.org/10.1371/journal.pcbi.1012248

3. Cuturello, F., Celoria, M., Ansuini, A. & Cazzaniga, A. Enhancing predictions of protein stability changes induced by single mutations using MSA-based Language Models. *bioRxiv* (2024). https://doi.org/10.1101/2024.04.11.589002

4. Dauparas, J. et al. Robust deep learning-based protein sequence design with ProteinMPNN. *Science* **378**, 49-56 (2022). https://doi.org/10.1126/science.add2187

5. Dieckhaus, H., Brocidiacono, M., Randolph, N. Z. & Kuhlman, B. Transfer learning to leverage larger datasets for improved prediction of protein stability changes. *Proc. Natl. Acad. Sci. U.S.A.* **121**, e2314853121 (2024). https://doi.org/10.1073/pnas.2314853121

6. Goverde, C. A. *et al.* Computational design of soluble and functional membrane protein analogues. *Nature* **631**, 449–458 (2024). https://doi.org/10.1038/s41586-024-07601-y

7. King, S. H. *et al.* Generative design of novel bacteriophages with genome language models. *bioRxiv* (2025). https://doi.org/10.1101/2025.09.12.675911

8. Lewis, S. *et al.* Scalable emulation of protein equilibrium ensembles with generative deep learning. *Science* **389**, eadv9817 (2025). https://doi.org/10.1126/science.adv9817

9. Milder, F. J. *et al.* Universal stabilization of the influenza hemagglutinin by structure-based redesign of the pH switch regions. *Proc. Natl. Acad. Sci. U.S.A.* **119**, e2115379119 (2022). https://doi.org/10.1073/pnas.2115379119

10. Nguyen, E. *et al.* Sequence modeling and design from molecular to genome scale with Evo. *Science* **386**, eado9336 (2024). https://doi.org/10.1126/science.ado9336

11. Ouyang, L. *et al.* Training language models to follow instructions with human feedback. *Adv. Neural Inf. Process. Syst.* **35**, 27730–27744 (2022).

12. Pacesa, M. *et al.* One-shot design of functional protein binders with BindCraft. *Nature* (2025). https://doi.org/10.1038/s41586-025-09429-6

13. Shanker, V. R., Bruun, T. U. J., Hie, B. L. & Kim, P. S. Unsupervised evolution of protein and antibody complexes with a structure-informed language model. *Science* **385**, 46–53 (2024). https://doi.org/10.1126/science.adk8946

14. Watson, J. L. *et al.* De novo design of protein structure and function with RFdiffusion. *Nature* **620**, 1089–1100 (2023). https://doi.org/10.1038/s41586-023-06415-8

15. Yassine, H. M. *et al.* Hemagglutinin-stem nanoparticles generate heterosubtypic influenza protection. *Nat. Med.* **21**, 1065–1070 (2015). https://doi.org/10.1038/nm.3927

**General comments**

We thank all the reviewers for their thoughtful comments and for providing an opportunity to increase the rigor and clarity of our manuscript.

In this revision, we have added:

1. *Stronger baseline analysis*. In response to the recommendation of Reviewer 2, we have added the requested analysis of how the experimentally tested ProteinDPO-identified mutations lie with the distribution of mutation effects among baseline methods. We have also identified and corrected errors in the previous analysis which inflated baseline performance, and updated the corresponding figures and text.

2. *Clarified novelty*. In response to Reviewer 3's feedback, we have provided the requested additional clarification on the methodological novelty of ProteinDPO. Specifically, we clarified differences between classes of machine learning models, helping frame an explanation of the methodological novelty of our approach, and how this brings about wider biological utility of ProteinDPO compared to baseline methods such as ThermoMPNN.

Other revisions are detailed individually in our point-by-point response below. These changes address the reviewers' concerns, and reaffirm the core demonstration of our work: namely, that DPO alignment of a biological generative model to a desired experimental property improved the model in stability prediction and stability-biased sequence generation, which was experimentally validated in the setting of influenza hemagglutinin.

## Point-by-point response

### Reviewer 1

The authors have satisfactorily addressed my concerns in this revision.

We are grateful that the reviewer finds our revisions satisfactory, and thank the reviewer for their initial comments which strengthened the rigor of our analysis and accuracy of our claims.

**Reviewer 2**

The authors adequately addressed all of my comments except one small point: I would still like them to analyze and describe whether the stabilizing mutations identified by DPO are predicted to be stabilizing according to the other models. This is different from the top-N analysis performed by the authors. As the authors note, the top-N analysis is insufficient because the other stabilizing mutations predicted by the other method may also be stabilizing. For completeness you might as well just show the full distribution of mutation effects predicted by the other methods (which you have already computed), along with where the successful/unsuccessful mutations lie in the distribution.

as before: "At minimum, the authors should take the top 30 mutants designed by ProteinDPO (the ones that were experimentally tested), examine how these mutants score according to "vanilla" ESM-IF and ThermoMPNN, and report (1) whether the ProteinDPO-designed mutants are predicted to be favorable according to other methods" "

We thank the reviewer for further clarifying their requested analysis. We addressed the remaining request, showing where experimentally-tested mutations designed by ProteinDPO lie within the full distribution of mutation effects of other models. In **Figure S8** (duplicated below) we also report classification metrics (accuracy and F1 score), which directly capture if these mutations are predicted to be stabilizing according to the other models.

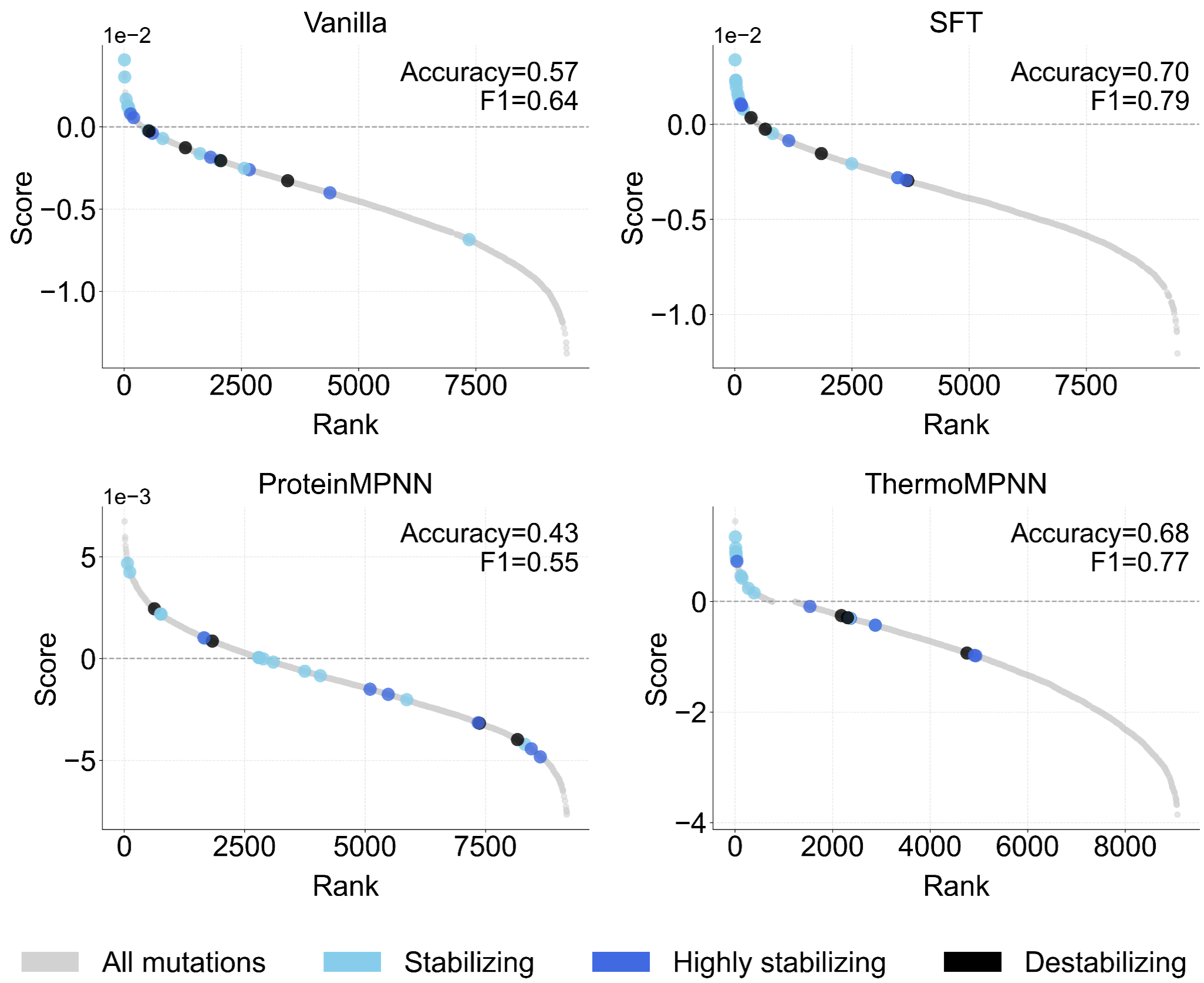


*Figure S8 | Method comparison in placement of stabilizing (light blue) and destabilizing (black) ProteinDPO-identified and experimentally-tested single-substitution variants within the score distributions all possible mutations (gray). Mutations are subset to those which had temperature of thermal unfolding (Tm) that was at least 1 degree higher or lower than the native sequence (N=23/30). Accuracy and F1 scores derived from the models' prediction of a given mutation being favorable or unfavorable is also shown. Additionally, the position of the six significantly stabilizing mutations (dark blue) analyzed in **Figure S6** are shown.*

Additionally, we have identified and corrected two errors in the previous analysis requested by Reviewer 2. First, in our mutation recovery analysis (**Figures S5 and S6**), all sequence positions were not scored across all baseline models. Second, in **Figure S7**, correlation values were incorrectly calculated. We have updated the figures (duplicated below) and amended the main text.

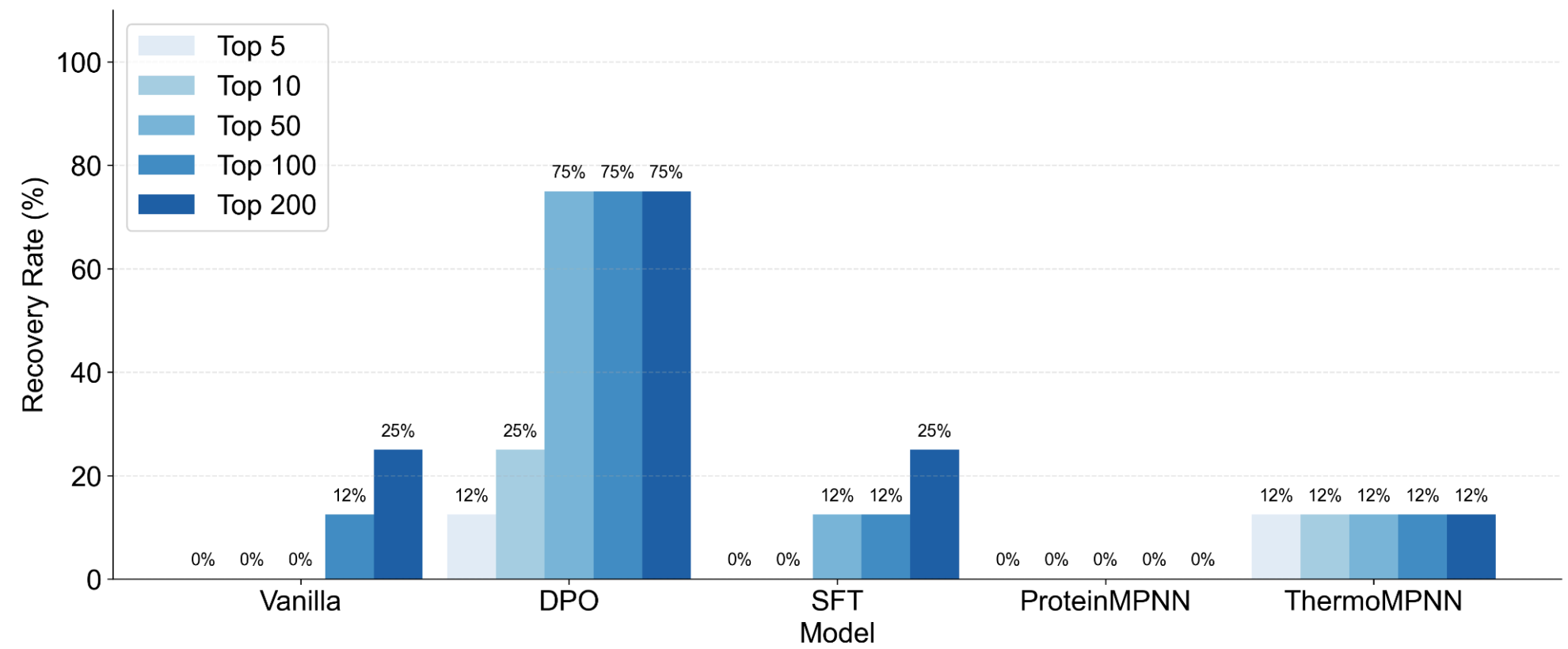


*Figure S5 | Recovery of literature-validated stabilizing mutations across protein design models. Bar plot showing the percentage of eight known stabilizing mutations (Milder et al. and Yassine et al.) recovered within the top-ranked predictions of each model. Models were evaluated using ensemble scoring across three PDB structures (6cf5, 6cfg, 2fk0).*

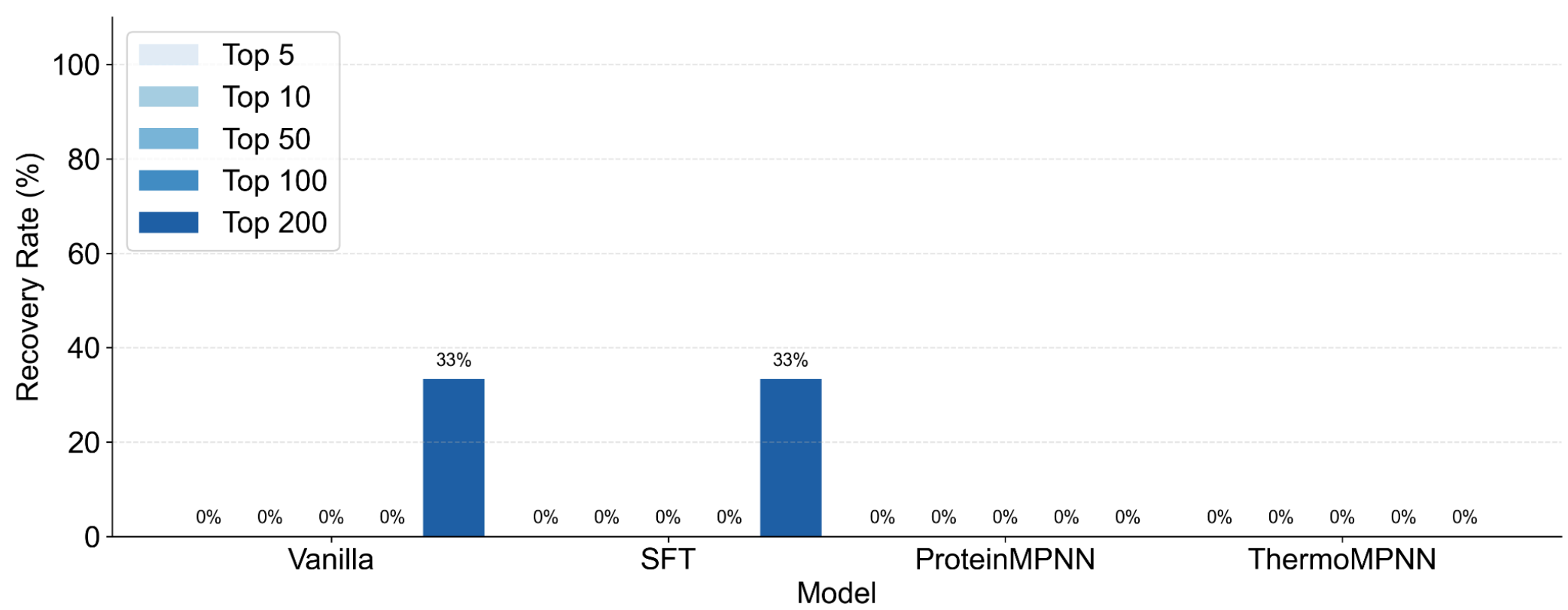


*Figure S6 | Recovery of highly-stabilizing ProteinDPO-identified single-substitutions across protein design models. Bar plot showing the percentage of six significantly stabilizing* (>= 9°C) *DPO-identified mutations recovered within the top-ranked predictions of each model. Models were evaluated using ensemble scoring across three PDB structures (6cf5, 6cfg, 2fk0).*

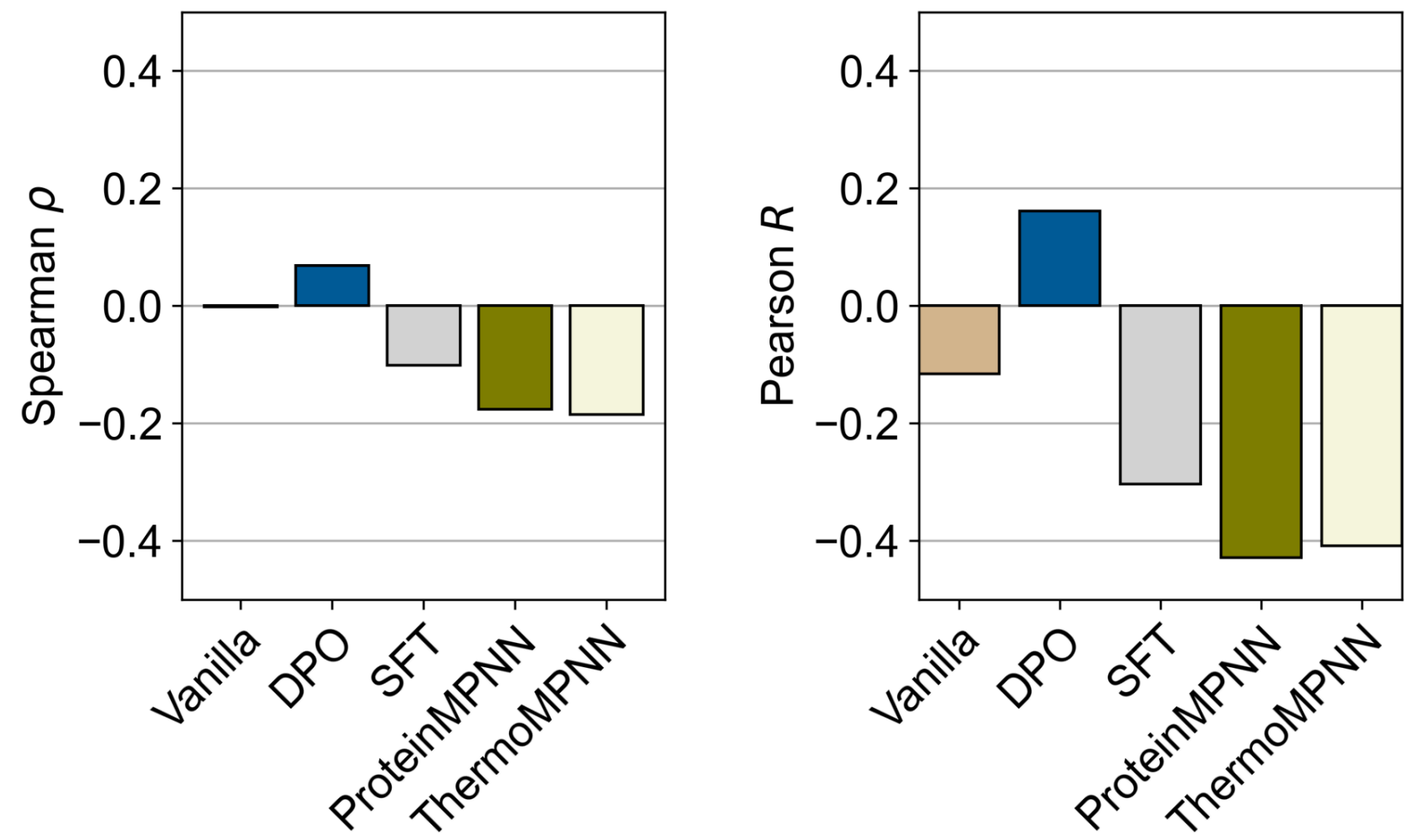


*Figure S7 | Comparison of methods in ability to rank the 30 experimentally tested single-substitution variants identified by ProteinDPO, measured by Pearson R and Spearman $\rho$ correlation coefficients between model scores and temperature of thermal unfolding (Tm).*

We then reference these result in the **Results** (bolded text indicates additions, strikethrough text indicates deletions):

> *We then conducted a similar analysis as done for literature mutation recovery, but instead comparing models' ability to identify six of the thirty ProteinDPO-identified single-substitution variants which were found to be significantly stabilizing (>= 9°C) (**Figure S6**). Only the SFT ~~model and ThermoMPNN~~ **and Vanilla models** were able to recover ~~one of these~~ mutations in their top-~~1~~200; although, it is possible other top-ranked mutants from these baseline models are also stabilizing. Additionally, **while all models are unable** ~~The SFT model, ProteinMPNN and ThermoMPNN nonetheless show modest ability~~ to rank the thermal stability (Tm) of these 30 variants **(Figure S7), the SFT model and ThermoMPNN show strong ability (F1 score > 0.75) to classify them as stabilizing or destabilizing (Figure S8), despite crucially failing to capture most highly-stabilizing mutations (Figures S6 and S8).***
>
> ***Beyond single-substitutions, and** ~~Looking~~ **aiming** to discover synergistic effects among beneficial mutations, we used ProteinDPO to score….*

**Reviewer 3**

I appreciate the authors' reasonable responses to points 2 and 3. However, I feel that point 1 has not yet been adequately addressed.

My original concern was not delivered clearly, so let me restate it. My comment was not intended to question the general "pretrain-and-fine-tune" strategy itself, but rather to discuss the underlying physical motivation and conceptual foundation of the model. I referred to BioEmu because it is trained on protein dynamics and folding, which are directly relevant to stability estimation and thus provide a clear rationale for transfer learning in this context. In contrast, the pretrained model used for ProteinDPO (ESM-IF) is similar to that of ThermoMPNN (ProteinMPNN), which has not explicitly been trained with such objectives. For this reason, I would group ProteinDPO more closely with ThermoMPNN, while distinguishing it from BioEmu.

My concern is not about the relative performance of the methods — indeed, the differences appear to be marginal based on Figures 2C–E and 3B–C — but rather about the novelty of the approach and its potential for future extension. Adapting a sequence design model for stability prediction has already been explored by ThermoMPNN, albeit in a different formulation, and there may be a general consensus regarding its practical upper bound in performance. Moreover, simply characterizing a model as "generative" does not, by itself, address this limitation. For example, if ThermoMPNN were retrained to preserve the original ProteinMPNN "generation" functionality, it is unclear whether this would be a fundamentally novel approach.

I would therefore suggest to clearly discuss the methodological limitations of the proposed approach in the Discussion section, which may explain its marginal difference to ThermoMPNN.

We appreciate your comments, which will be helpful for clarifying our result. As suggested, we have expanded our **Discussion** section to elucidate the methodological novelty of our approach over ThermoMPNN. These additions fall under addressing two points: (1) the difference between generative models and supervised regressors and (2) how this explains the methodological novelty and broader biological utility of ProteinDPO compared to ThermoMPNN.

1. The reviewer questions "if ThermoMPNN were retrained to preserve the original ProteinMPNN "generation" functionality". In fact, this "retraining to preserve generation" is non-trivial and precisely the contribution of our manuscript. To alleviate this concern, we further clarify the relationship between generative models and supervised regressors, highlighting the importance of retaining generative capabilities.

2. We also note that ProteinDPO has much greater conceptual novelty than ThermoMPNN. Instead of fine-tuning an inverse folding model as a regressor (which is commonly done

> in many computational biology workflows) to predict some aspects of stability, we use reinforcement learning-based alignment (a methodology far less explored) to align the probability distribution of the generative model to both *score and generate* sequences according to preferences determined by experimental data. We then show that the scoring capability of ThermoMPNN becomes a subset of ProteinDPO's capabilities (**Figure 2B,D,E**), though ProteinDPO is strictly more capable across a broader variety of tasks than is ThermoMPNN. Indeed, while there are limited settings where the improvement of ProteinDPO over ThermoMPNN is marginal (**Figure 2C,D**), there are several important and useful biological settings where ThermoMPNN cannot be used. These include absolute stability prediction with a fold, stability-aligned sequence design, multi-chain scoring, and binding prediction (**Figure 3B,C,D**), with ProteinDPO demonstrating a clear improvement that is precisely due to fundamental differences in methodology contributed by our manuscript.

We have updated the second-to-last paragraph in the **Discussion** section to emphasize these two points (bolded text indicates additions, strikethrough text indicates deletions):

> *While the model is competitive with supervised, task-specific ~~models~~* ***regression models*** *such as ThermoMPNN [15], it has not clearly surpassed all supervised models in scoring [12] (**Figure 2B,D,E**).* ***However, using reinforcement learning, unlike supervised regression, preserves essential generative capabilities. This enables ProteinDPO to both score sequences (like regression models) and generate entirely new sequences aligned with desirable functions, which are capabilities that supervised models cannot combine. This generative capability further extends to*** *~~However, ProteinDPO remains a generative model, which enables stability-informed sequence generation for natural or de novo protein folds with autoregressive sampling.~~ ~~ProteinDPO also generalizes to a greater variety of tasks than existing supervised models, being able to predict $\Delta\Delta G$,~~* ***the*** *prediction of binding affinity~~,~~ and thermal melting across diverse wild-type proteins, multi-chain antibodies, and protein complexes more accurately than the unaligned ESM-IF1 model* ***(Figures 3B,C,D), and the sampling of experimentally-validated nine-substitution variants (Figures 4 and 5).*** *~~This contrasts with models that are limited to scoring single-mutation $\Delta\Delta G$s of monomeric structures.~~* ***Furthermore,*** *we find that alignment based on one specific property (such as the stability of small monomers) improves performance ~~function prediction for other~~* ***on these*** *diverse tasks (such as binding affinity of large protein-protein complexes), most likely because both properties share common underlying physical principles.*

11/18/2025

**General comments**

We thank all the reviewers for their thorough and constructive feedback on our manuscript. We appreciate the positive reception and are also grateful for their detailed comments which have led to substantial improvements to the clarity, rigor, and detail of our manuscript.

In this revision, we have:

1. *Added more comprehensive benchmarks*. Following reviewer requests, we have included comparisons to other models. Specifically, we now benchmark against ESM-IF1 (both zero-shot and after SFT), ThermoMPNN, and Rosetta for the HA stabilization task **(Fig. S5,6)**, demonstrating that ProteinDPO is superior in recovering literature mutations, while other models are not able to identify highly stabilizing variants from the literature or from our validated experimental pool. We have also added ProteinMPNN benchmarking for protein-protein binding **(Fig. 3B)** and RosettaREU evaluation for antibody thermal stability **(Fig. 3C)**, finding ProteinDPO has competitive or improved performance compared to benchmark models.

2. *Toned down claims*. We have carefully revised statements throughout the manuscript to ensure accurate representation of our results. This includes altering our claims with respect to vaccine design and clarifying that absolute stability predictions refer to relative improvements within a protein fold rather than absolute predictions across different folds. We have also added appropriate caveats regarding the limitations of computational metrics like Rosetta energy, and clarified that certain redesigned sequences in **Fig. 3** were not experimentally verified.

3. *Added more methodological details on our implementation and evaluations*. We have substantially expanded the **Methods** section to improve clarity. This includes new subsections for Model sampling, Sampling evaluation, Rosetta generation evaluation, and Scoring metrics **(Methods 6.6)**, detailed pseudocode for all training algorithms including Paired, Ranked, and Weighted DPO as well as SFT training **(Methods 6.7)**, and a new Mutation selection section describing the multi-mutation design protocol **(Methods 6.8)**. We have also added distributional plots for key results **(Fig. S3, S4)**, clarified dataset versions used, improved experimental methods descriptions, and fixed installation issues. In addition to pseudocode in the Methods, we attach the raw training code used for ProteinDPO as a supplementary zip file (**Supplementary Code**).

Other revisions are detailed individually in our point-by-point response below. These revisions address the reviewers' concerns while demonstrating the core contributions of our work: (1) that DPO can be used to align biological generative models with a desired experimental property and (2) our aligned model, ProteinDPO, improves upon its unsupervised predecessor to be useful as a stability predictor and stability-biased sequence generator, which we experimentally validate in the setting of influenza hemagglutinin.

## Point-by-point response

### Editorial comments

We recommend adding more comprehensive benchmarking against available methods in this field as well as addressing all the technical concerns. Regarding Reviewer #1's point 2 (vaccine relevance) - additional experiments to demonstrate that designed variants elicit protective immune responses against the target virus would be out of scope for us, so we recommend you tone down these claims.

We thank the editor for recognizing the interest in our work from the reviewers, and providing us the opportunity to improve our manuscript. To address the concern of comprehensive benchmarking, we have implemented the reviewers' suggestion of benchmarking against ProteinMPNN and Rosetta for binding and stability prediction (in addition to previous analyses benchmarking against both base and SFTed ESM-IF1), as well as benchmarking against a range of baseline models for the experimental application of hemagglutinin stabilization. We appreciate the guidance regarding vaccine relevance, and have toned down these claims accordingly. For example, in the **Abstract**:

*"Leveraging up to nine substitutions, we achieved a 17°C improvement in melting temperature on a HA from human H5N1 influenza A virus while preserving ~~antigenicity~~* ***recognition by existing antibodies****, and up to 32°C stability improvements across recently emerged mammalian viral H5 HAs."*

As well as in the **Results** section:

*"To determine whether ProteinDPO-identified mutations ~~compromise the antigenic properties required for vaccine efficacy~~* ***maintain key epitopes recognized by existing antibodies****, we measured the binding affinity of HA variants with nine substitutions to anti-HA broadly neutralizing antibodies (bnAbs) targeting either the head or the stem domains of HA via biolayer interferometry (BLI).*

And added an additional statement in the **Discussion** section:

*"Moreover, while pre-fusion stabilization and retention of binding to native antibodies are key components of vaccine design, further evidence would be needed to demonstrate antigenic properties required for vaccine efficacy"*

**Reviewer 1**

In this work, the authors introduce an approach called Direct Preference Optimization (DPO) to enhance desired properties of protein sequences generated by computational models. Specifically, they focus on protein stability using the inverse protein folding model ESM-IF1, and further optimize the model with protein mutational data. The authors report improvements in several benchmark tests compared with the original unsupervised ESM-IF1 and a fine-tuned supervised model, and in some cases, with another approach named ThermoMPNN. Finally, they apply the method to design hemagglutinin variants for influenza vaccines, reporting increased stability. The study presents an interesting strategy for optimizing deep learning models in protein design. However, I am not fully convinced that the proposed approach provides a significant advantage based on the current evidence. My major concerns are outlined below:

We thank the reviewer for recognizing the merits of our work, and include a point-by-point response below to address their major concerns of our method.

1. Lack of comparison in the HA application. While benchmark improvements are demonstrated, the application to HA design, arguably the most important example in this work, was not compared with other existing approaches. Since ESM-IF1 itself has shown some promising performance in similar tasks, it remains unclear how much Protein DPO actually improves upon it. A detailed comparison with alternative methods is necessary to convincingly establish the benefit of Protein DPO.

We thank the reviewer for raising this concern. On the task of HA stabilization, we now benchmark against ESM-IF1, SFT, ThermoMPNN, and ProteinMPNN in these models' ability to highly rank stabilizing mutations (1) from the literature and (2) out of the 30 single-mutations which were experimentally validated in our first revision, in part following the guidance of Reviewer 2. When comparing the ability of ESM-IF1, SFT, ThermoMPNN, ProteinMPNN and ProteinDPO to recover known HA-stabilizing mutations from the literature, specifically Milder et al [9] and Yassine et al [15] **(Fig. S5)**, we found that while some models were able to recover one mutation in their top-50, ProteinDPO has a higher recovery rate across all top-n levels.

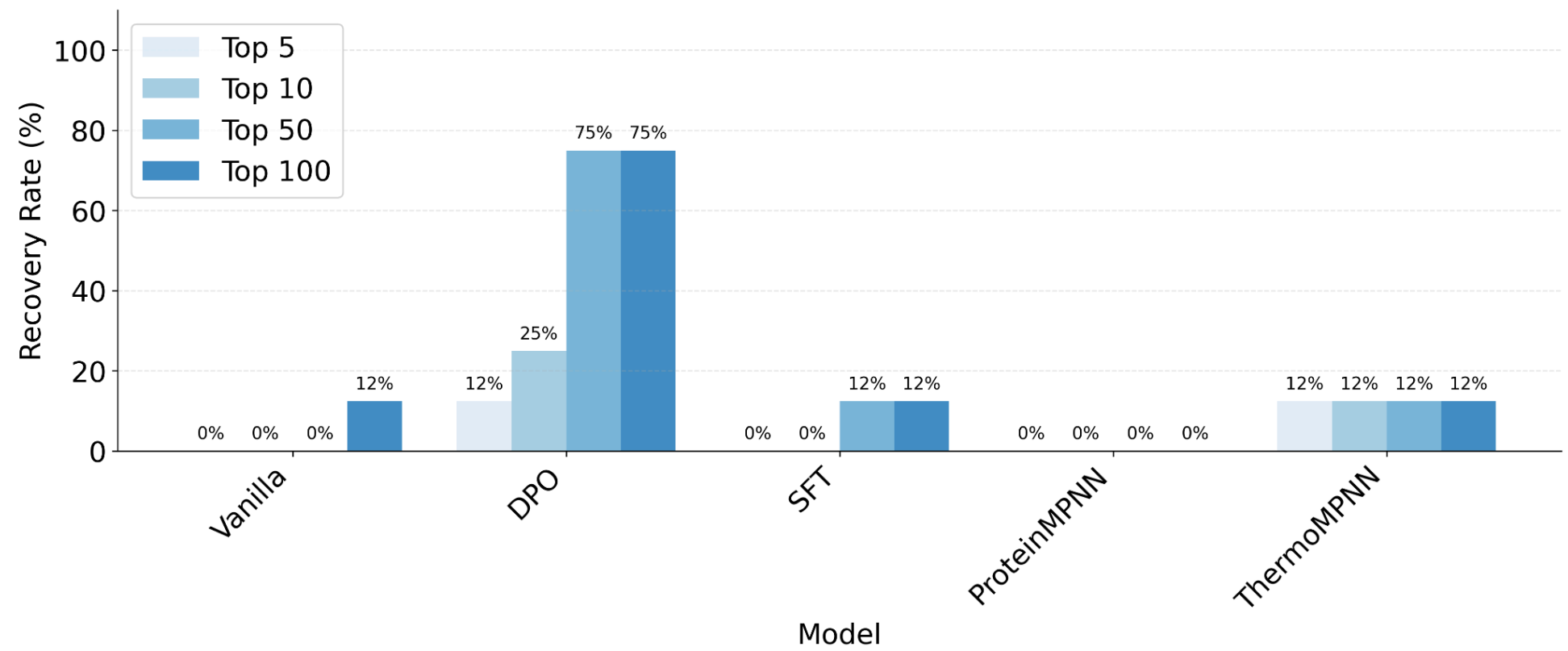


*Figure S5 | Recovery of literature-validated stabilizing mutations across protein design models. Bar plot showing the percentage of eight known stabilizing mutations (Milder et al. and Yassine et al.) recovered within the top-ranked predictions of each model. Models were evaluated using ensemble scoring across three PDB structures (6cf5, 6cfg, 2fk0). n=8 total literature mutations.*

Additionally, we compared the ability of ESM-IF1, SFT, ProteinMPNN and ThermoMPNN to rank the 6/30 highly-stabilizing (>= 9°C) mutations from the pool of 30 single-mutations we tested **(Fig. S5)**. Only the SFT model and ThermoMPNN were able to recover one of these mutations in its top-100.

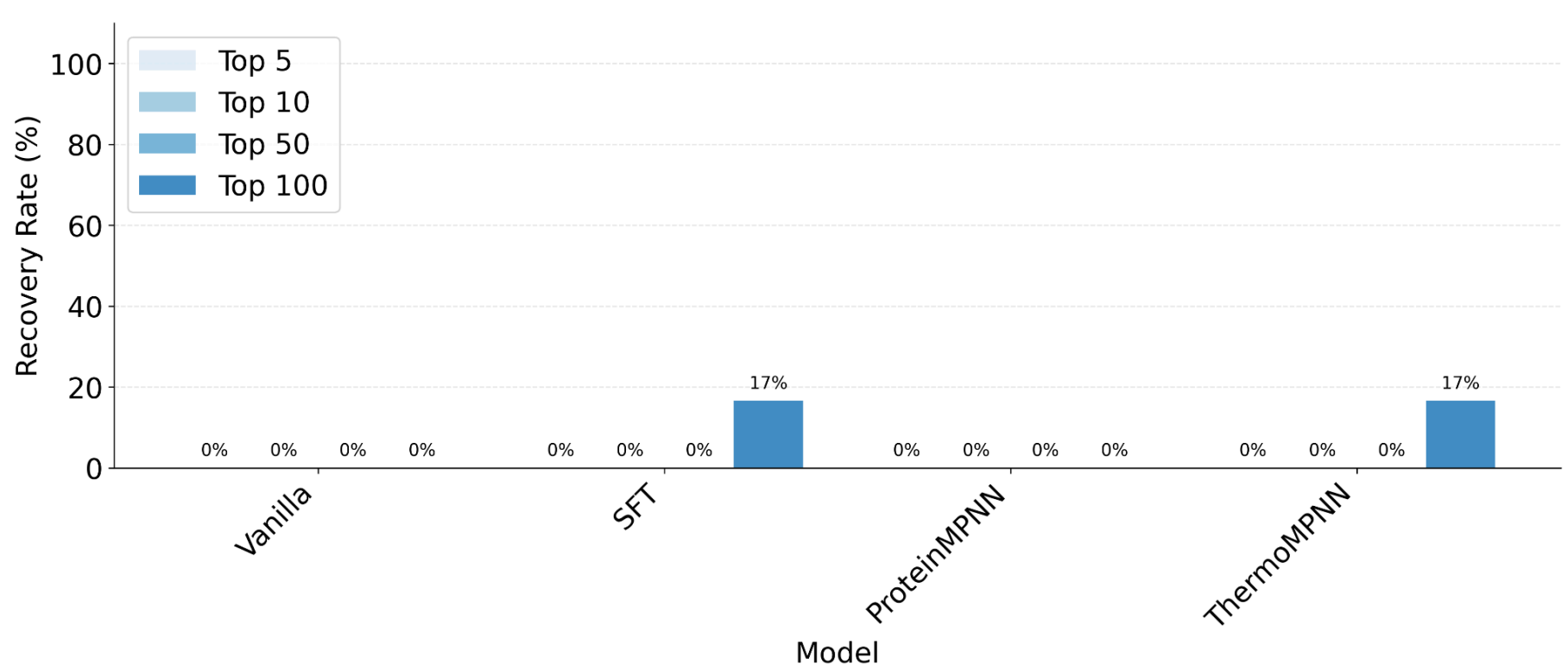


*Figure S6 | Recovery of experimentally tested DPO mutations by baseline models. Bar plot showing the percentage of six significantly stabilizing* (>= 9°C) *DPO-identified mutations recovered within the top-ranked predictions of each model. Models were evaluated using ensemble scoring across three PDB structures (6cf5, 6cfg, 2fk0).*

Both analyses suggest that ProteinDPO is superior in highly ranking literature mutations, enabling low-n stabilization. Additionally the compared methods are not able to recover most of the best performing ProteinDPO mutations. We do note that a limitation of this test is that some of the highly ranked mutations by other methods may be stabilizing, which we also acknowledge in our text:

*‘Only the SFT model and ThermoMPNN were able to recover one of these mutations in its top-100; however, it is possible top-ranked mutants from these baseline models are also stabilizing.”*

2. Vaccine relevance not fully established. For vaccine design, the key requirement is demonstrating that designed variants elicit protective immune responses against the target virus. The manuscript shows recognition of designed variants by existing antibodies, but this does not sufficiently demonstrate antigenic properties required for vaccine efficacy. Stronger experimental validation would be needed to support claims of relevance to vaccine design.

We thank the reviewer for raising this concern. We agree that the results presented do not conclusively demonstrate vaccine efficacy. Considering the following guidance from the editor:

*“additional experiments to demonstrate that designed variants elicit protective immune responses against the target virus would be out of scope for us, so we recommend you tone down these claims”*

We toned down the implications of existing antibody recognition in the **Abstract**:

*“Leveraging up to nine substitutions, we achieved a 17°C improvement in melting temperature on a HA from human H5N1 influenza A virus while preserving ~~antigenicity~~* ***recognition by existing antibodies****, and up to 32°C stability improvements across recently emerged mammalian viral H5 HAs.”*

as well as in the **Results** section:

*"To determine whether ProteinDPO-identified mutations ~~compromise the antigenic properties required for vaccine efficacy~~* ***maintain key epitopes recognized by existing antibodies****, we measured the binding affinity of HA variants with nine substitutions to anti-HA broadly neutralizing antibodies (bnAbs) targeting either the head or the stem domains of HA via biolayer interferometry (BLI).*

and added an additional statement in the **Discussion** section:

*“Moreover, while pre-fusion stabilization and retention of binding to native antibodies are key components of vaccine design, further evidence would be needed to demonstrate antigenic properties required for vaccine efficacy”*

3. Potential overemphasis on stability. The importance of stability may be overstated. For example, Figure 3D shows that the model generates sequences with improved stability according to Rosetta energy functions, but it is unclear whether these sequences are experimentally synthesized. Extremely stable sequences often risk aggregation due to excess hydrophobic residues, which may hinder practical expression and use. Moreover, the sequence design procedure is insufficiently described in the Methods. Was it simply initiated from random seeds, as in ESM-IF1?

We agree the Rosetta evaluation places an overemphasis on stability and needs clarification that these sequences were not experimentally tested. To address the former, we have added this clarification to the text:

*"Although Rosetta energy can be inaccurate and is not a substitute for experimental testing, it is a widely used independent evaluator of machine learning generations due to its reliance on physics-based parameters rather than shared training data with machine learning models [44, 5]"*

To address the latter, we added this clarification:

*"While these sequences were not experimentally verified to express, …."*

Additionally, we added a **Model sampling**, **Sampling evaluation**, and **Rosetta generation evaluation** sub-sections to the methods (**Methods 6.6**) to clearly describe the sampling procedure and evaluation for this experiment.

4. In Section 2.3, the claim that the method can rank "absolute stability of diverse antibodies" seems misleading, since only correlation coefficients are reported. These results reflect relative stability rankings, not absolute values. Furthermore, comparisons across antibodies likely rely on their shared fold and similar length. It is doubtful that the approach could be applied to rank melting temperatures across proteins of different folds.

We thank the reviewer for bringing up this concern. Being able to rank stability *within* a specific protein fold is what we intended to convey with this evaluation. We have made the following clarifications to the main text, only mentioning the term absolute when referring to ranking within a given fold:

*"we sought to evaluate if ProteinDPO can use whole-sequence reasoning to rank absolute stability of ~~diverse antibodies~~* ***sequences within a given fold, particularly antibodies"***

*"on the ability of vanilla ESM-IF1 in ranking the ~~absolute~~ differences in thermal melting points"*

*"ProteinDPO generalizes to scoring binding affinity of large complexes and ~~absolute antibody~~ thermal stability **of highly diverse and variable length antibodies"***

5. The Methods section needs clarification and expansion, particularly regarding benchmark evaluation. A separate subsection should clearly describe the metrics used (e.g., correlation coefficients), how they are computed, and the specific model outputs used for evaluation. In addition, figures such as 2B and 3B would benefit from showing distributional statistics rather than only averages, as mean values can obscure important variation.

We thank the reviewer for raising this concern regarding clarification in the methods section. To alleviate this we have added a sub-section, **Scoring metrics**, to the methods **(Methods 6.6)** to clearly describe the metrics used, including each of the coefficients. In this new text we point to the **Datasets** section **(Methods 6.5)** where one can find the specific model outputs used for evaluation.

Additionally, we added distributional plots for 2B and 3B **(Fig. S3, S4)** including for the newly benchmarked ProteinMPNN, and reference them in the main text:

*"Distributional plots of the individual values are included in the supplement **(Fig. S3)**."*

*"Distributional plots of the individual values for the AB-Bind dataset are included in the supplement **(Fig. S4)**."*

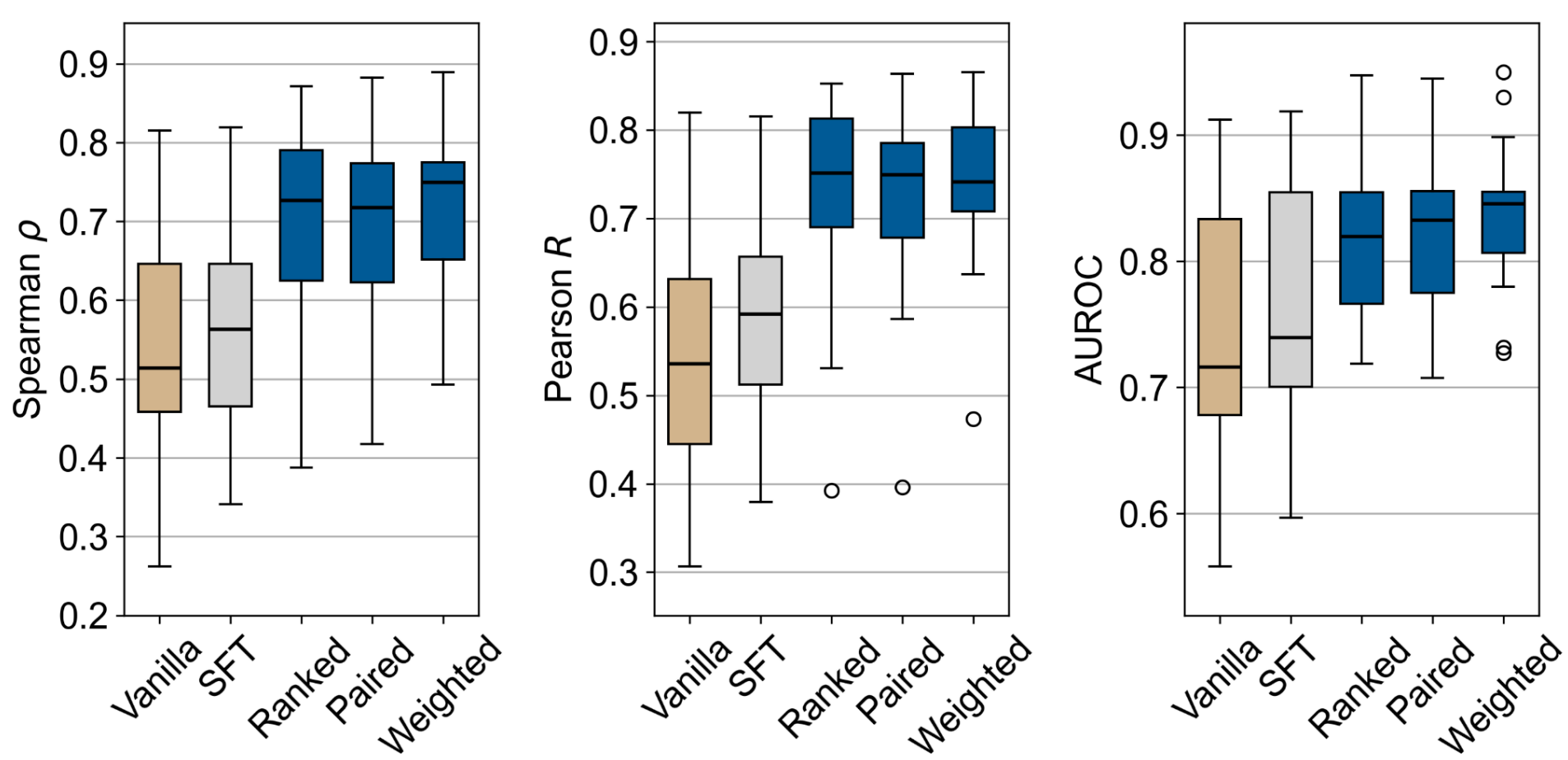


*Figure S3 | Distribution of performance across protein domains for predicting stability changes upon mutation (ΔΔG) in curated Megascale holdout dataset.*

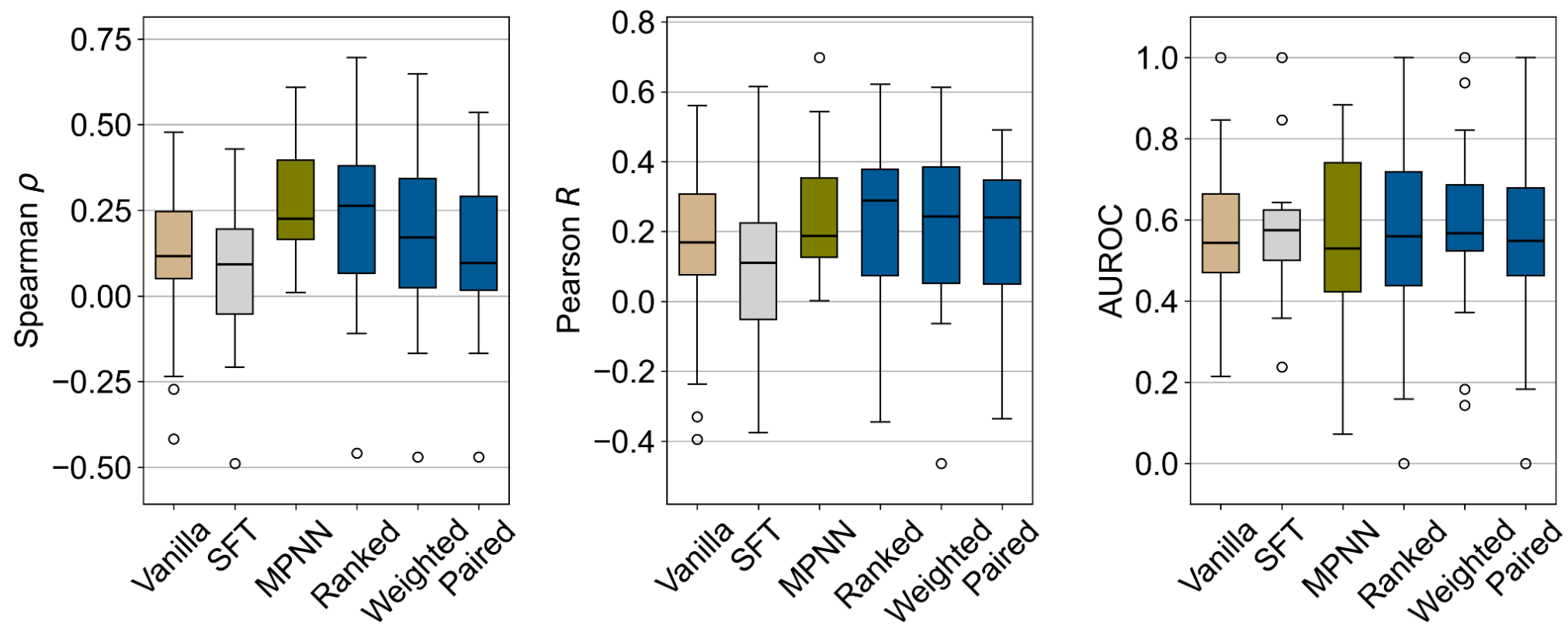


*Figure S4 | Distribution of performance across antibody-antigen complexes for predicting binding affinity changes upon mutation in AB-Bind dataset.*

6. The main contribution of this work lies in the proposed algorithm for optimizing ESM-IF1, but the Methods provide insufficient implementation details. Moreover, the GitHub repository only contains scripts and weights for inference, without the training code or implementation necessary for reproduction. At minimum, the authors should provide pseudocode for their approach—particularly for the weighted DPO algorithm, which is a novel aspect of this study.

We thank the reviewer for raising this concern. We have now included in-depth pseudocode for the approach. We have added algorithms for training the Paired, Ranked, and Weighted DPO models, as well as SFT training and log-likelihood scoring. All of these have been added to the **Model training** section in the Methods **(Methods 6.7)**. We have also added our training code for the model as **Supplementary Code**.

**Reviewer 2**

This paper applies the Direct Preference Optimization (DPO) approach to "align" the structure-conditioned protein language model ESM-IF with the objective of designing high stability protein sequences. The authors point out that the "base" model ESM-IF is trained to recreate the probability distribution of the training data, which does not match the common objective in protein design to produce high stability proteins. The DPO method improves the alignment between the distribution sampled by the model and the author's (and community's) design objective of sampling high stability proteins. The resulting model (ProteinDPO) can be used for both scoring structures and for generating new sequences based on an input protein backbone. The authors examine several variants of ProteinDPO, computationally benchmark ProteinDPO on several design and ranking tasks against other leading models, and also apply ProteinDPO to experimentally design vaccine antigens with higher stability.

The main strengths of the paper are:

-The problem of improving generative AI models for protein design is timely and important and the approach of using DPO based on experimental folding stability data is relatively novel.

-The flexiblity of ProteinDPO enables the model to be applied to score multi-mutation variants as well as entire unrelated structures, which is rare in the field of stability prediction. The model achieves superior performance over a naive application of ThermoMPNN at predicting effects of multiple mutants (Fig. 2C). The model also shows a modest correlation with antibody binding and melting temperature (Fig 3B/C), which is superior to ESM-IF ("Vanilla") and cannot achieved using models like ThermoMPNN or MAESTRO that are designed to score single mutants.

-For predicting single mutants, several variants of ProteinDPO have strong performance at the S669 benchmark and come close to matching ThermoMPNN at the Fireprot benchmark.

-Using a computational benchmark, the authors show that ProteinDPO-based protein design improves on ESM-IF based protein design according to the Rosetta energy functon(Fig. 3D).

-The experimental work demonstrates a successful application of ProteinDPO. However, this work is seemingly not intended to illustrate the superiority of ProteinDPO over other methods- the proposed mutants are not computationally evaluated using any method other than ProteinDPO.

We thank the reviewer for appreciating and highlighting the strengths of our work, particularly the need for improving protein generative models via alignment, the flexibility of the resulting model for stability prediction, and the successful experimental application.

The main weaknesses of the paper are:

1. Although the authors make the case that ProteinDPO has unique applications beyond other stability models (such as Fig. 2C, Fig 2B, Fig 3C), the experimental demonstration doesn't really highlight these applications. Instead, it illustrates an application where other stability models might have performed equally well or better.

We thank the reviewer for bringing up this concern. While we were interested in addressing these unique applications (binder design, backbone stabilization, antibody stabilization), in efforts of balancing model validation and finding a relevant practical application, we decided hemagglutinin stabilization was an especially interesting choice. We also note that ProteinDPO readily generalizes to multi-mutation scoring and note that this was tested in our experimental work **(Fig. 2C)**. While several substitutions in the multi-mutation variants were derived from single-mutants verified as stable from the initial round of 20 mutations (labeled DPO-A in **Table S6**), for many of the 5-substitution and 9-substitution variants, ProteinDPO included substitutions that were never previously evaluated as singletons, instead using its likelihood function to evaluate a combined score and demonstrating multi-mutation variant generation. We provide further detail on the multi-mutation selection protocol in a section titled **Mutation selection** in the Methods section **(Methods 6.8)**. Additionally, the HA structure is a homo-trimeric complex, and explicit scoring of multi-chain complexes is inaccessible to many stability models, including ThermoMPNN.

2. The authors don't examine the performance of other stability models at their experimental demonstration. It would be very useful to the audience to know whether the output of ProteinDPO is unique or similar to predictions made by other stability models. At minimum, the authors should take the top 30 mutants designed by ProteinDPO (the ones that were experimentally tested), examine how these mutants score according to "vanilla" ESM-IF and ThermoMPNN, and report (1) whether the ProteinDPO-designed mutants are predicted to be favorable according to other methods, (2) whether they also rank near the top of all mutants evaluated by other methods, and (3) whether other methods show an ability rank mutants by Tm within the top 30 designed by ProteinDPO. It would also be good to note whether ProteinDPO itself can rank these 30 mutants, although this is a somewhat unfair task since they are all already highly favorable according to ProteinDPO.

We are grateful for the set of recommended tasks, which we have performed and were also helpful in responding to a question from Reviewer 1. For HA stabilization, we benchmarked

against ESM-IF1, SFT, ThermoMPNN, and ProteinMPNN in these models' ability to highly rank stabilizing mutations (1) from the literature and (2) out of the 30 single-mutations which were experimentally validated in our first revision. For known HA-stabilizing mutations from the literature, specifically from Milder et al [9] and Yassine et al [15] **(Fig. S5)**, ProteinDPO has a much higher recovery rate across all top-n levels.

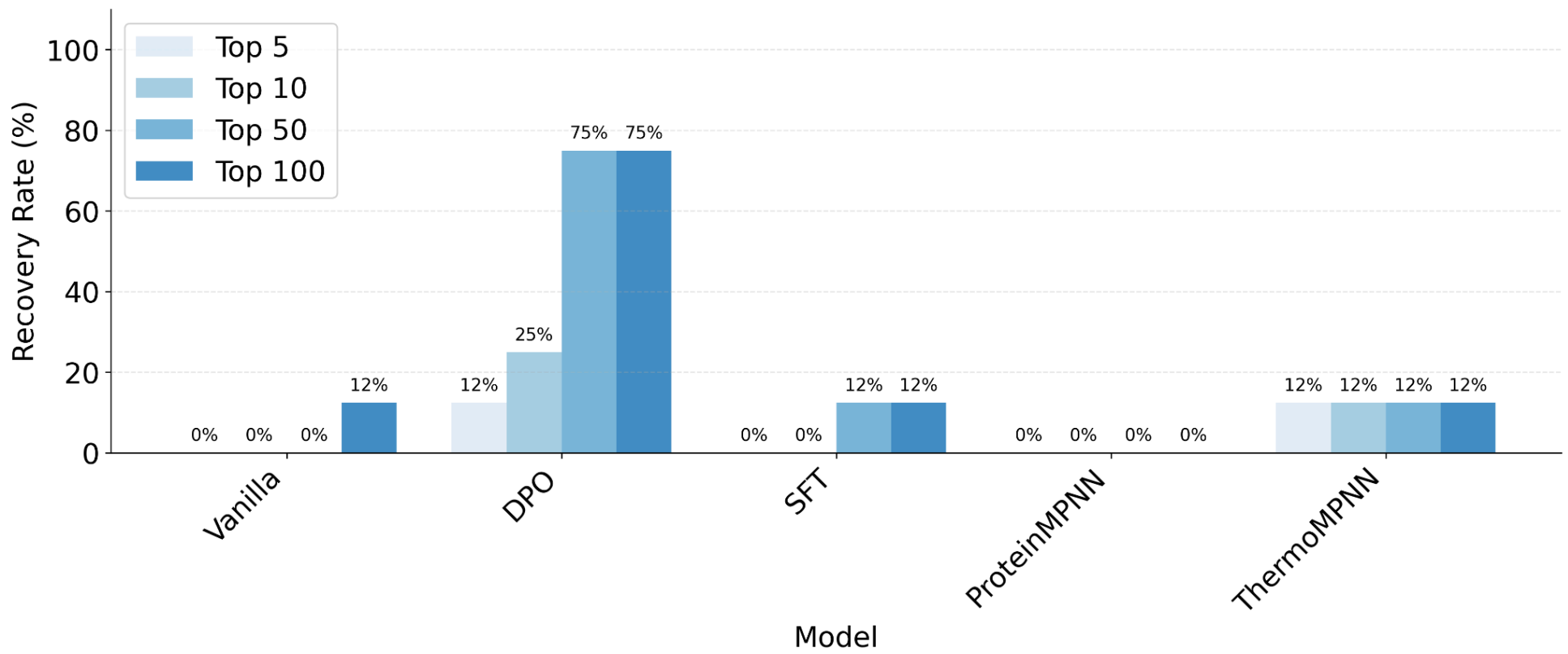


*Figure S5 | Recovery of literature-validated stabilizing mutations across protein design models. Bar plot showing the percentage of eight known stabilizing mutations (Milder et al. and Yassine et al.) recovered within the top-ranked predictions of each model. Models were evaluated using ensemble scoring across three PDB structures (6cf5, 6cfg, 2fk0). n=8 total literature mutations.*

Additionally, we compared the ability of ESM-IF1, SFT, ProteinMPNN and ThermoMPNN to rank the 6/30 highly-stabilizing (>= 9°C) mutations from the pool of 30 single-mutations we tested **(Fig. S5)**. Only the SFT model and ThermoMPNN were able to recover one of these mutations in its top-100.

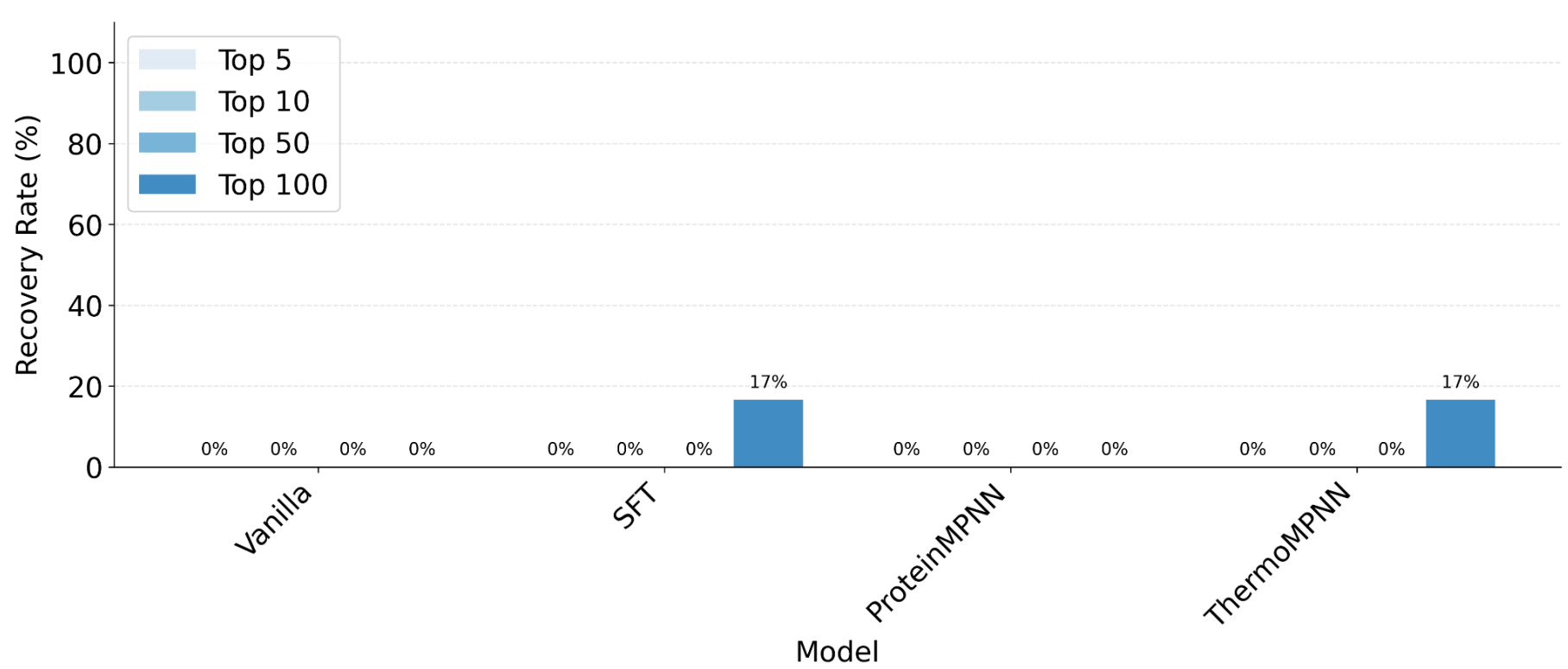


*Figure S6 | Recovery of experimentally tested DPO mutations by baseline models. Bar plot showing the percentage of six significantly stabilizing (>= 9◦C) DPO-identified mutations recovered within the top-ranked predictions of each model. Models were evaluated using ensemble scoring across three PDB structures (6cf5, 6cfg, 2fk0).*

Both analyses suggest that ProteinDPO is superior in highly ranking literature mutations within its top-n mutations, enabling low-n stabilization. We do note that a limitation of this test is that some of the highly ranked mutations by other methods may be stabilizing, which we also acknowledge in our text:

*'Only the SFT model and ThermoMPNN were able to recover one of these mutations in its top-100; however, it is possible top-ranked mutants from these baseline models are also stabilizing."*

We also conducted the third analysis that evaluates "whether other methods show an ability to rank mutants by Tm within the top 30 designed by ProteinDPO." As an important caveat, as expected, ProteinDPO cannot rank its own top-30 mutants, as these are all extremely high-likelihood mutations coming from the top ~0.3% of all possible mutations. The vanilla model, SFT and ProteinMPNN show modest ability to do so, with ThermoMPNN, being the only model trained on stability prediction, performing the best. We include the results of this analysis in **Fig. S7**:

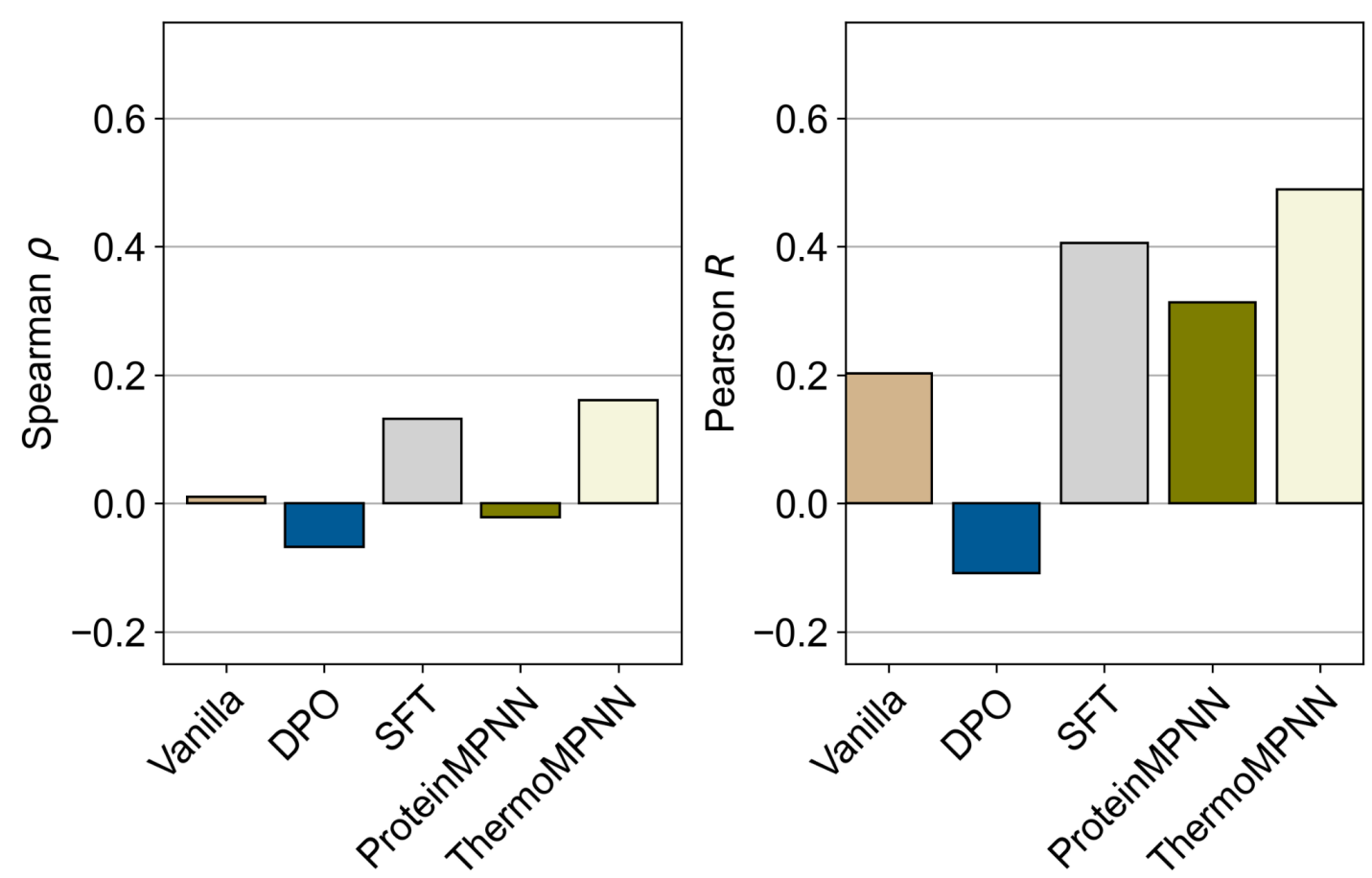


*Figure S7 | Comparison of methods in ability rank the 30 experimentally tested single-substitution variants identified by ProteinDPO, measured by Pearson R and Spearman ϱ correlation coefficients between model scores and temperature of thermal unfolding (Tm).*

We also refer to these results in the main text:

*The SFT model, ProteinMPNN and ThermoMPNN do however show modest ability to rank the thermal stability (Tm) of these 30 variants* ***(Fig. S6)****.*

3. The benchmarking of ProteinDPO against other models for antibody melting temperature and protein-protein binding is somewhat minimal. Only ESM-IF is used as a benchmark. Are any

other benchmarks available? At minimum, RosettaREU (total energy, energy per residue, or ddG) could be used, as in Fig 3D.

We thank the reviewer for pointing out the need for additional benchmarking, and for recommending methods to utilize. To address this concern, we evaluated RosettaREU on the antibody thermal stability benchmark, finding that using total energy, rather than energy per residue is a superior metric. We have added this benchmark to the main text **(Fig. 3C).**

For the protein-protein binding benchmark, despite extensive effort, we found it very challenging and computationally intensive to use Rosetta for protein-protein binding evaluation. Naively relaxing the complexes with the mutations applied and extracting a ddG by difference in energy yielded very poor results (and resulted in several PDB and mutation processing errors); we then attempted to use the recommended flex_ddg protocol, which yielded similar errors and would also have taken an estimated 500,000 CPU hours for the ~8500 mutations in SKEMPIv2 and AB-Bind under recommended settings, which was not feasible for us to run in a reasonable timeline. Instead, we have added ProteinMPNN as another baseline method for the protein-protein binding benchmark (**Fig. 3B)** [4], given it is a widely used tool for binder design [1, 11] and the comments by Reviewer 3 asking us to use ProteinMPNN as a benchmark comparison. Our updated **Fig. 3** is provided below:

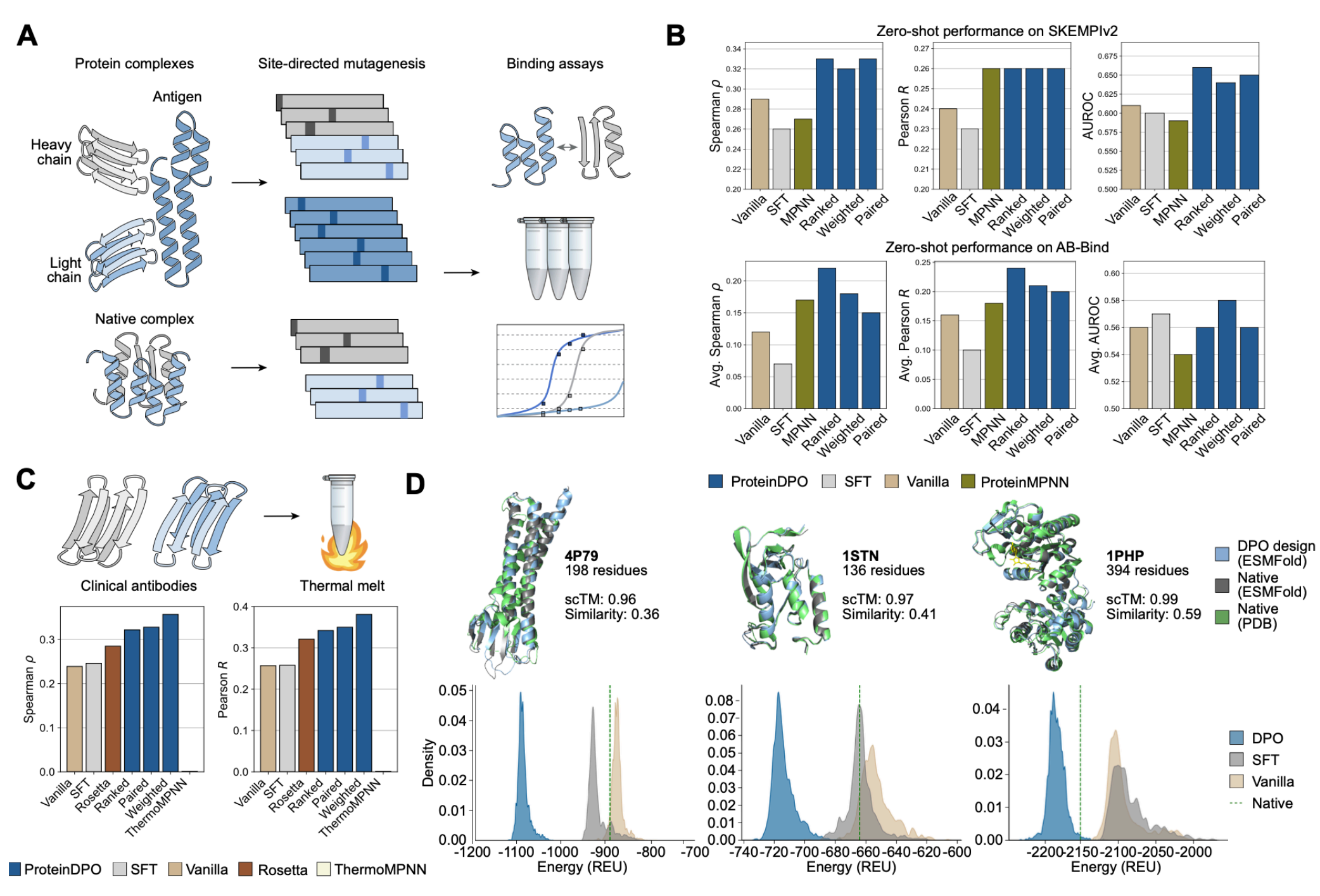


4. The authors need to be careful about how they describe the overall accuracy of the ProteinDPO method. For example, in the 4th paragraph of the discussion, the authors write

"ProteinDPO also generalizes to a greater variety of tasks than existing supervised models, being able to predict ΔΔG, binding affinity, and thermal melting across diverse wild-type proteins, multi-chain antibodies, and protein complexes." This ability to "predict binding affinity" is based on spearman rho values of 0.2 - 0.33 - poor overall. The ability to predict thermal melting is based on spearman rho of ~0.35 - again poor. It would be accurate to say that the ProteinDPO model can predict these quantities ***more accurately than the vanilla ESM-IF model***, which is a worthwhile computational achievement. But the authors need to avoid unqualified statements like saying "ProteinDPO can predict X".

We thank the reviewer for raising this concern and agree with these comments. For the noted cases, we have described these results as "modest":

*"ProteinDPO has modestly increased scoring of the binding affinity of large protein-protein complexes"*

And have also revised several statements in the text:

*"Notably, the aligned model also performs well in domains beyond its training data to enable stabilization and **improved** binding affinity prediction of large multi-chain proteins."*

*"and thermal melting across diverse wild-type proteins, multi-chain antibodies, and protein complexes **more accurately than the unaligned ESM-IF1 model."***

***"**ProteinDPO generalizes to **improved** ~~scoring~~ binding affinity **scoring** of large complexes and thermal stability of highly diverse and variable length antibodies**,"***

5a. It's not correct to say that FireProt and S669 are made from deep mutational scanning datasets since these are generally piecemeal individual measurements, not multiplexed comprehensive measurements.

Thank you for this comment. We have made this clarification in the text.

*"We benchmarked models using ~~deep mutational scanning (DMS)~~ **experimental protein stability** dataset FireProt and S669. **These datasets are compiled from small-scale** ~~compiled from~~ mutagenesis experiments across diverse monomeric proteins, in which certain residues are mutated, variants are expressed, and their stability is measured experimentally."*

*"These datasets are compilations of experimental stability measurements ~~of mutations~~ derived from several **small-scale** mutagenesis experiments of native proteins"*

5b.  Should lower right panel be AUROC?

Yes, it should, we have fixed this error. Thank you for pointing this out.

5c. Fig 4B: It seems like the best single mutant is as good as the best combination of mutants? Is this correct? Does this mean that combining mutants was unsuccessful? Or is the light blue point at the top not a single mutant? A different coloring scheme would be beneficial. The authors should be more explicit in the text about the best single mutant.

Yes, it is correct that the best single mutant is similar to the best combination of mutants, and have added a note of this to the main text. In the text, we do not make strong claims about the multi-mutation variants selected by ProteinDPO having higher stability than could be derived by additivity of the single-mutants. However, we do highlight that the five sets of ProteinDPO-identified nine-mutants are well tolerated by the structure and generally stabilizing.

*"While the best nine-substitution variant did not significantly surpass the best single-substitution variant, nine-substitution variants were 9.8 °C more stable on average than single-substitution variants."*

We give full details on the sequence and stability of all mutants in the Supplementary **(Supp. Table S6)**, to be more explicit we have added a note in the main text to refer to this table.

*"All variants and their associated melting temperatures are reported in the supplement (Table S6)"*

We have also adjusted the color scheme for a better distinction between the number of mutations.

5d. The CD wave scans look unusual- why is the signal so much weaker in DPO-19 and WT compared to DPO-4? If the proteins are folded and have the same structure, they should show the same spectrum. Is it possible the protein concentrations were measured incorrectly for all proteins except DPO-4? The methods should mention how protein concentrations were measured. A folded alpha-beta protein should have a signal with the intensity of the signal for DPO-4 (see a reference on this)- the other signals are strangely weak.

Yes, this was likely due to concentration differences. When collecting the CD wave scans, concentration was measured using a NanoDrop spectrophotometer and ensured to be in the range of 0.1-0.3 mg/ml as this was the guidance for using this particular CD spectrometer. It appears this window was too large between samples. We have updated the methods regarding how concentration was measured:

*"Protein samples* ***were*** *diluted or concentrated to* ~~*approximately 0.2 mg/ml*~~ ***concentration within the range of 0.1mg/ml to 0.3mg/ml*** *in PBS at 20°C.* ***Concentration was calculated using a Thermo Scientific NanoDrop OneC Microvolume Spectrophotometer measuring for absorbance at 280nm."***

and added a disclaimer to the main text.

*"Differences in absorbance signal magnitude are due to concentration differences between samples* ***(Methods)"***

5e. Methods: The authors should state which version of the Megascale dataset they used (v1 12/6/2022 or v2 4/20/2023).

We have added a statement of the version of the dataset used.

*"We used the Megascale dataset for training, either with our own training splits* ***(derived from v2 4/20/2023)****, those from Diekhaus et al, or those from Cuturello et al depending on subsequent evaluation."*

*"The Megascale dataset* ***(v2 4/20/2023)*** *served as the experimentally measured stability dataset from which we aligned the pretrained ESM-IF1."*

**Reviewer 3**

This work by Widatalla et al applies the DPO method to protein stability engineering problem. Protein stability engineering is clearly an important research subject, and the application of DPO technique to this problem is somewhat novel. There were several works in which protein language models were fine-tuned for the purpose, but to my knowledge, the application of DPO seems the first. I think validating their method through actual experiments is very encouraging and valuable; however, two major concerns listed below make it hard to fully appreciate the quality of the work.

We thank the reviewer for recognizing the importance of protein stabilization, the novelty of our approach, and our experimental validation. We are also grateful for the questions and recommended analyses, which have improved the manuscript.

1. The first is the basic philisophy of the work. From a fundamental point of view, is "tuning a pre-trained model" a relevant philosophy for the protein stability estimation? Regarding the complexity of protein folding and stability, I think this idea will only give a marginal improvement over existing methods sharing the same concept; i.e. another (slightly better) ThermoMPNN. A good work to compare is the recently published BioEmu, in which the authors simulated protein folding to approximate the equilibrium. I cannot see such conceptual novelty in this work that tackles the inherent challenge of the problem. Introducing DPO technique may add little novelty in this regard.

We thank the reviewer for raising the concerns regarding “tuning a pre-trained model” and its relevance to stability prediction. While protein folding stability is a complex phenomenon, additional training beyond pre-training is an effective approach, as several state-of-the-art models (e.g., ThermoMPNN and MSAesm_ddG [5, 3]), are fine-tuned pretrained language models. Moreover, BioEmu [8] gains its stability prediction through additional fine-tuning; after its pretraining task of learning to generate protein conformations, similarly to ProteinDPO, it undergoes a stability fine-tuning stage on the Megascale dataset [8].

Regarding BioEmu, we note that ProteinDPO achieves a higher Spearman correlation (~0.7) than BioEMu (~0.6) in regards to stability prediction as reported in the section **Predicting protein stabilities** in our manuscript and Fig. 4 in the BioEmu manuscript [8] (though we note that ProteinDPO and BioEmu used slightly different Megascale train and test splits). Importantly, while BioEmu showed its simulations recapitulated folded versus disordered states from proteins in the CALVADOS and ProthermDB databases, Importantly, BioEmu did not show generalizability of stability (ddG) prediction outside of the Megascale dataset, while we show ProteinDPO generalizes in stability prediction to FireProt and S669 **(Fig. 2D, 2E)**.

Regarding the comparison to ThermoMPNN, ProteinDPO is inherently different to ThermoMPNN and other ddG prediction models. ProteinDPO remains a generative model; instead of predicting a scalar value given a mutation (usually limited to only a single-substitution for most models), ProteinDPO produces a probability distribution of amino acids (biased towards stable sequences via DPO) given an input backbone, from which one can sample to generate an entire sequence. While we use comparisons to stability predictors to measure the optimization performance, ProteinDPO is fundamentally different as it remains a generative model. Remaining a generative model allows ProteinDPO to naturally score multi-mutation variants in-silico, as well as generate them in the lab. Furthermore, the use of structure-conditioned language models to design large portions of protein sequences given a backbone has been successful in many design applications spanning design protein binders, enzymes and membrane proteins [1, 4, 6, 12, 14]. ProteinDPO now enables structure-conditioned sequence design that is biased towards even greater stability, a desired attribute in many design applications.

2. The method is repeatedly compared against vanilla or STF, but I don't think this is a valuable comparison. This may show "how bad ESM-if" rather than "how good ProteinDPO" is for the problem. Also, it is hard to believe SFT in the manuscript was trained by their best. Therefore, it is worth testing just once for ablation, and the main comparison should be against other methods like ThermoMPNN. Also, the authors keep argue "method solely trained on small proteins generalizes to bigger protein". This is not an advantage but a prerequisite; methods not satisfying such property should be less appreciated.

Comparison to other methods is not very strong, but just based on the current figures, it is hard to identify clear advantages of the method. This marginal (or neglible) improvement should be related to the inherent limitation of the approach as pointed out in point 1.

Thank you for these comments. We compared ProteinDPO to ESM-IF1 (and SFT) across all evaluations since this is the model we initialize the weights with, and allows for specific testing of the hypothesis of whether DPO training improves scoring and generation performance. Comparing ProteinDPO to the vanilla ESM-IF1 and an SFTed model ensures we can controllably isolate the effect of DPO training, as the pretraining data, initial weights, parameter count, and featurization are the same between the three models. With regards to concerns that the results primarily reflect “how bad ESM-IF is”, we note that ESM-IF1 is generally considered a strong base model, for example having been used experimentally for enriching binding affinity in Shanker et al. [13]. Thus we see improving upon it as a valuable contribution.

Additionally, supervised fine-tuning is a good baseline methodology as it is widely used in almost all state-of-the-art language models for natural text [11] and which has shown success experimentally for biological applications [6, 12, 10, 7].

In regards to concerns that SFT was not trained properly, we have added additional training details to the **Methods** section **(Supervised Finetuning)**, and included loss curves of the learning rate sweeps that were conducted to find an optimal checkpoint in the supplemental **(Fig. S2)**. Across different SFT training runs, we find that all checkpoints perform similarly:

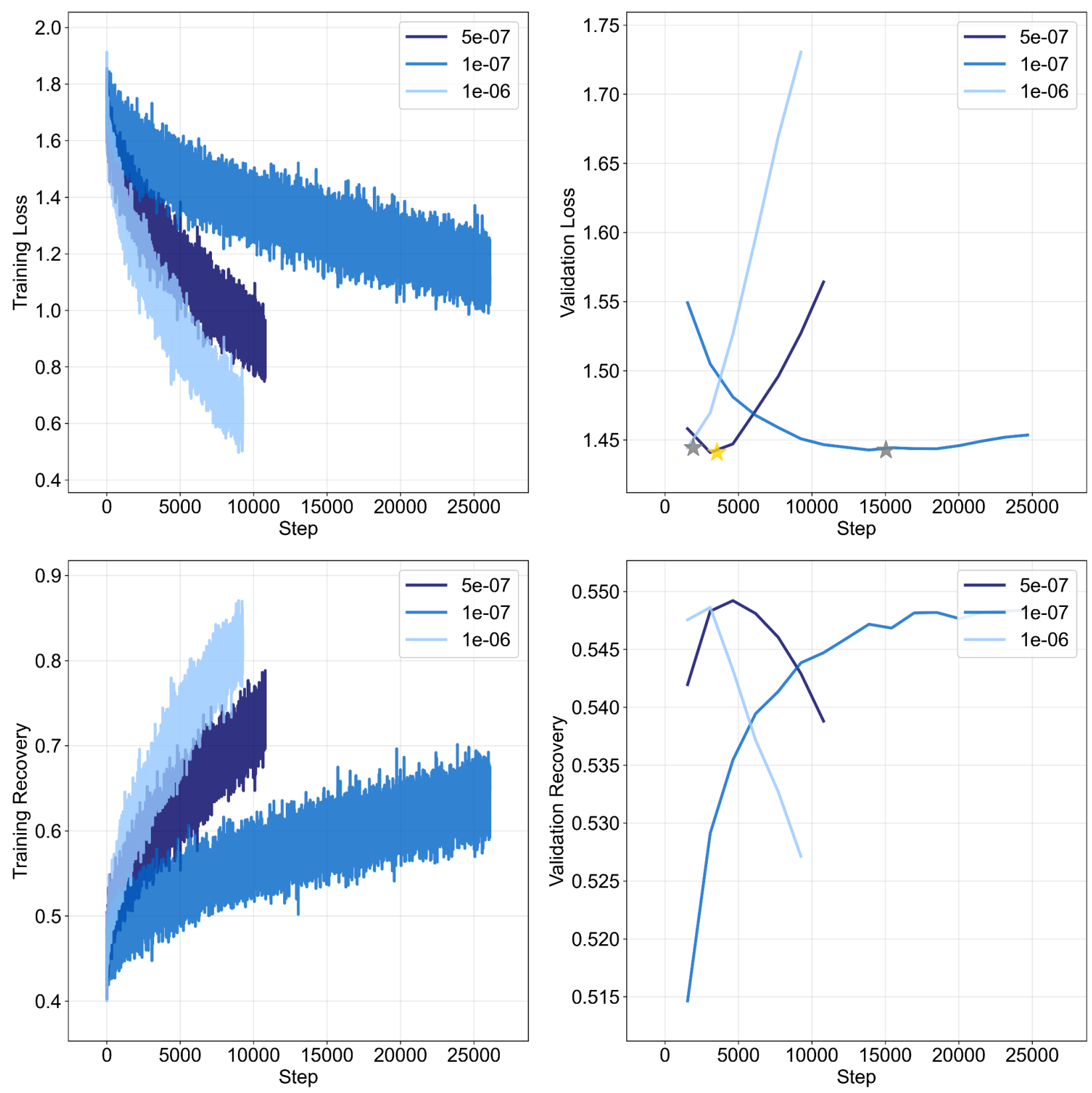


*Figure S2 | Training and validation loss and sequence recovery curves for training the supervised finetuned model. The selected checkpoint was the checkpoint which best minimized validation loss across all runs, and is denoted with a star. Checkpoints which minimized validation loss for other runs, are denoted with a gray star, and are compared against the selected checkpoint in* ***Table S7.***

| Learning rate | Megascale (mean) | FireProt | AB-Bind (mean) | Antibody thermal stability |
|---|---|---|---|---|
| 1e-7 | 0.584/0.561 | 0.430/0.441 | 0.10/0.05 | 0.250/0.236 |
| 5e-7 | 0.588/0.570 | 0.415/0.429 | 0.10/0.06 | 0.258/0.246 |
| 1e-6 | 0.577/0.556 | 0.422/0.433 | 0.10/0.04 | 0.249/0.235 |

*Table S7 | Comparison of different SFT checkpoints trained with varying learning rate on stability and binding prediction in terms of (mean) Pearson R / Spearman ρ correlation coefficients.*

Regarding ThermoMPNN, we benchmarked this model across several datasets (MegaScale single-mutants, Megascale double-mutants, S669, and Fireprot). However, we could not benchmark against ThermoMPNN for SKEMPI, AB-Bind or Antibody thermostability due to the inability of ThermoMPNN to score complexes or absolute differences in highly diverse sequences (e.g., those with different length). In lieu of ThermoMPNN, we have added benchmarking of ProteinMPNN and Rosetta, for protein-protein binding and antibody thermostability.

We also agree with the reviewer that generalization of a model to larger proteins is a "prerequisite, not advantage" for any model that ought to be used to predict protein stability. Notably, many previous machine learning models have had a tendency to overfit on their respective domain and to fail to generalize to larger proteins. A recent work featuring a Megascale-fine-tuned version of the ESM protein language model experienced this; despite performing well on prediction for a MegaScale test set, this model did not generalize to other DMS datasets, where the authors of that study cited a mismatch in the distribution of sequence lengths [2].

Lastly, we agree with the reviewer that for certain tasks, such as protein-protein binding, and antibody-antigen binding, the improvements from the base model are modest. However, for stability prediction, our performance improvements are indeed meaningful. Specifically, we see large improvements in stability prediction in the Megascale holdout set (Pearson $R$ and Spearman $\varrho$ improvements of 0.16 to 0.19), and the S669 and FireProt datasets (Pearson $R$ and Spearman $\varrho$ increases of 0.13 to 0.16). These improvements generalize to ranking absolute stability of diverse antibodies Pearson $R$ and Spearman $\varrho$ improvements from 0.08 to 0.12). Additionally, in **Figure 3D**, we see how these differences in scoring translate to a clear difference in the predicted stability of full sequence generations when evaluated by an orthogonal physics-based approach, Rosetta.

3. This is a suggestion for the presentation of the experimental section. As I mentioned above, experimental validation is very encouraging. At the same time, readers may wonder what if other already-established method was tested in parallel. For example, ProteinMPNN already demonstrated its ability to improve stability in various previous works other methods (e.g. Rosetta) failed. It should have been much more impactful if ProteinDPO have proved experimetnal success in a similar way (although a relevant baseline now has largely shifted from Rosetta to ProteinMPNN since then). Otherwise readers may have impression that results presented were cherry-picked.

We are grateful for this comment, which is similar to comments from the two other reviewers. On the task of HA stabilization, we now benchmark against ESM-IF1, SFT, ThermoMPNN, and ProteinMPNN in these models' ability to highly rank stabilizing mutations (1) from the literature and (2) out of the 30 single-mutations which were experimentally validated in our first revision, in part following the guidance of Reviewer 2. When comparing the ability of ESM-IF1, SFT, ThermoMPNN, ProteinMPNN and ProteinDPO to recover known HA-stabilizing mutations from the literature, specifically Milder et al [9] and Yassine et al [15] **(Fig. S5)**, we found that while some models were able to recover one mutation in their top-50, ProteinDPO has a higher recovery rate across all top-n levels.

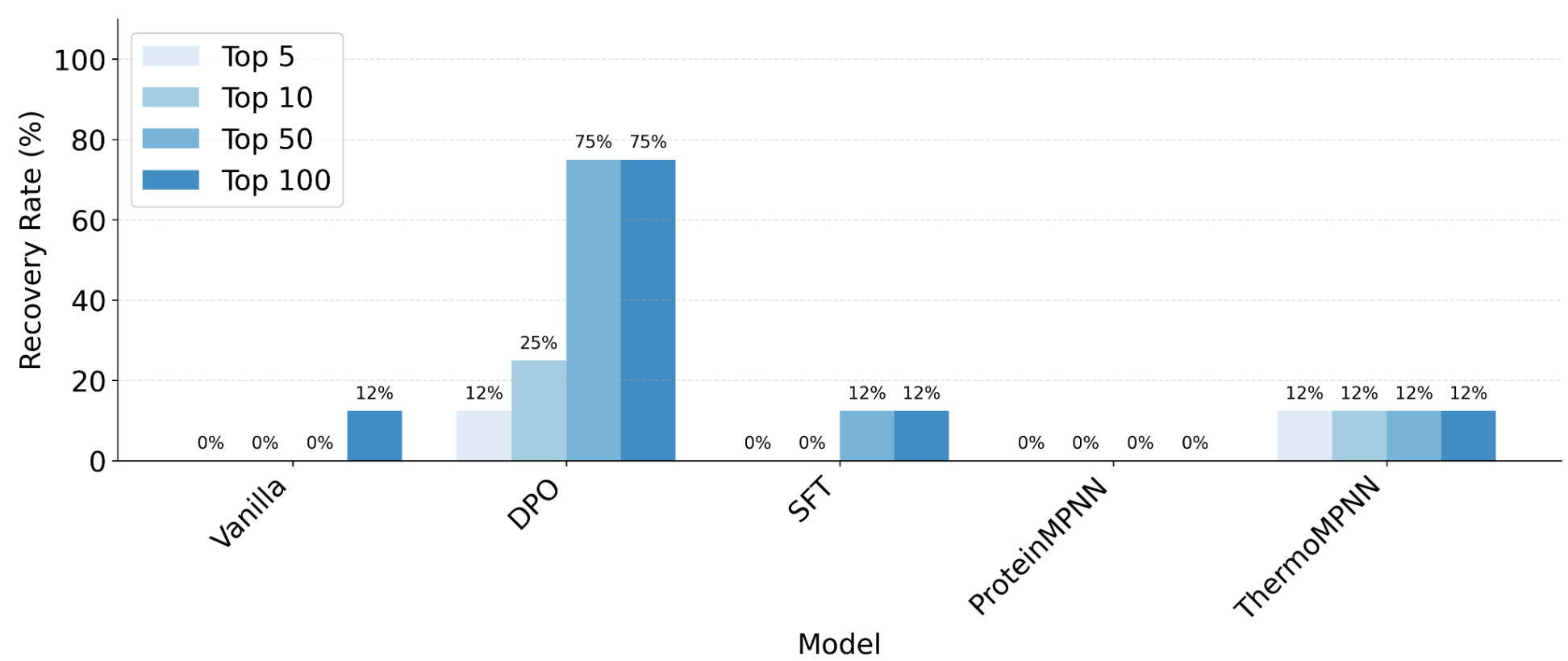


*Figure S5 | Recovery of literature-validated stabilizing mutations across protein design models. Bar plot showing the percentage of eight known stabilizing mutations (Milder et al. and Yassine et al.) recovered within the top-ranked predictions of each model. Models were evaluated using ensemble scoring across three PDB structures (6cf5, 6cfg, 2fk0). n=8 total literature mutations.*

Additionally, we compared the ability of ESM-IF1, SFT, ProteinMPNN and ThermoMPNN to rank the 6/30 highly-stabilizing (>= 9°C) mutations from the pool of 30 single-mutations we tested **(Fig. S6)**. Only the SFT model and ThermoMPNN were able to recover one of these mutations in its top-100.

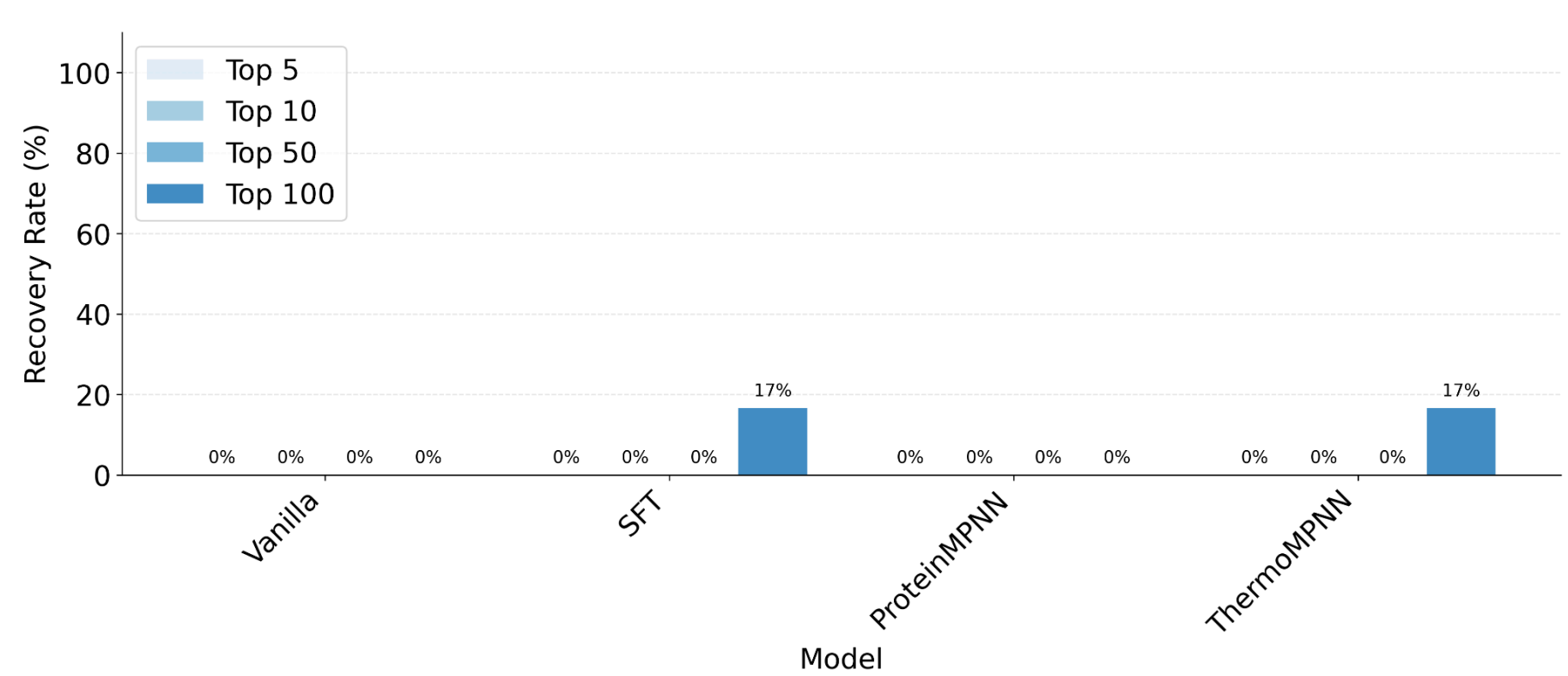

*Figure S6 | Recovery of experimentally tested DPO mutations by baseline models. Bar plot showing the percentage of six significantly stabilizing (>= 9◦C) DPO-identified mutations recovered within the top-ranked predictions of each model. Models were evaluated using ensemble scoring across three PDB structures (6cf5, 6cfg, 2fk0).*

Both analyses suggest that ProteinDPO is superior in highly ranking literature mutations, enabling low-n stabilization. Additionally the compared methods are not able to recover most of the best performing ProteinDPO mutations. We do note that a limitation of this test is that some of the highly ranked mutations by other methods may be stabilizing, which we also acknowledge in our text:

*'Only the SFT model and ThermoMPNN were able to recover one of these mutations in its top-100; however, it is possible top-ranked mutants from these baseline models are also stabilizing."*

4. I was not able to install the code with the error message below:

"Solving environment: failed

ResolvePackageNotFound:

- python[version='3.10.*,==3.9.18',build=h955ad1f_0]"

I do see this issue was posted in the github a bit ago, but was not fixed. Please fix this issue before your resubmission. The inference code itself is very simple and straightforward.

Thank you for bringing this to our attention, the installation issue has been fixed in commit #2ef299f.

## References


1. Bennett, N. R. *et al.* Atomically accurate de novo design of antibodies with RFdiffusion. *Nature* (2025). https://doi.org/10.1038/s41586-025-09721-5
2. Chu, S. K. S., Narang, K. & Siegel, J. B. Protein stability prediction by fine-tuning a protein language model on a mega-scale dataset. *PLOS Comput. Biol.* **20**, e1012248 (2024). https://doi.org/10.1371/journal.pcbi.1012248
3. Cuturello, F., Celoria, M., Ansuini, A. & Cazzaniga, A. Enhancing predictions of protein stability changes induced by single mutations using MSA-based Language Models. *bioRxiv* (2024). https://doi.org/10.1101/2024.04.11.589002
4. Dauparas, J. et al. Robust deep learning-based protein sequence design with ProteinMPNN. *Science* **378**, 49-56 (2022). https://doi.org/10.1126/science.add2187
5. Dieckhaus, H., Brocidiacono, M., Randolph, N. Z. & Kuhlman, B. Transfer learning to leverage larger datasets for improved prediction of protein stability changes. *Proc. Natl. Acad. Sci. U.S.A.* **121**, e2314853121 (2024). https://doi.org/10.1073/pnas.2314853121
6. Goverde, C. A. *et al.* Computational design of soluble and functional membrane protein analogues. *Nature* **631**, 449–458 (2024). https://doi.org/10.1038/s41586-024-07601-y
7. King, S. H. *et al.* Generative design of novel bacteriophages with genome language models. *bioRxiv* (2025). https://doi.org/10.1101/2025.09.12.675911
8. Lewis, S. *et al.* Scalable emulation of protein equilibrium ensembles with generative deep learning. *Science* **389**, eadv9817 (2025). https://doi.org/10.1126/science.adv9817
9. Milder, F. J. *et al.* Universal stabilization of the influenza hemagglutinin by structure-based redesign of the pH switch regions. *Proc. Natl. Acad. Sci. U.S.A.* **119**, e2115379119 (2022). https://doi.org/10.1073/pnas.2115379119
10. Nguyen, E. *et al.* Sequence modeling and design from molecular to genome scale with Evo. *Science* **386**, eado9336 (2024). https://doi.org/10.1126/science.ado9336
11. Ouyang, L. *et al.* Training language models to follow instructions with human feedback. *Adv. Neural Inf. Process. Syst.* **35**, 27730–27744 (2022).
12. Pacesa, M. *et al.* One-shot design of functional protein binders with BindCraft. *Nature* (2025). https://doi.org/10.1038/s41586-025-09429-6
13. Shanker, V. R., Bruun, T. U. J., Hie, B. L. & Kim, P. S. Unsupervised evolution of protein and antibody complexes with a structure-informed language model. *Science* **385**, 46–53 (2024). https://doi.org/10.1126/science.adk8946

14. Watson, J. L. *et al.* De novo design of protein structure and function with RFdiffusion. *Nature* **620**, 1089–1100 (2023). https://doi.org/10.1038/s41586-023-06415-8

15. Yassine, H. M. *et al.* Hemagglutinin-stem nanoparticles generate heterosubtypic influenza protection. *Nat. Med.* **21**, 1065–1070 (2015). https://doi.org/10.1038/nm.3927

## Point-by-point response

**Reviewer 2**

I approve of the final additions to the manuscript and find it suitable for publication as-is.

We are grateful that the reviewer finds the final additions satisfactory and the manuscript suitable for publication, and thank the reviewer for comments which strengthened the rigor of our work.

**Reviewer 3**

Remarks to the Author:

The revised manuscript now contains much clearer explanation about the method's novelty. I recommend for the acceptance of this manuscript.

Remarks on code availability:

The code is straightforward and works well. It would be helpful if the pip version requirement were updated to a more recent release (e.g., >25.x.x).

We are grateful that the reviewer now finds our manuscript satisfactory and appreciate their assistance in helping us craft a clearer explanation of the novelty. We have addressed their concern on code availability by updating the environment to pip version >25.x.x on our public Github repository.

**General comments**

We thank all the reviewers for their thoughtful comments and for providing an opportunity to increase the rigor and clarity of our manuscript.

In this revision, we have added:

1. *Stronger baseline analysis*. In response to the recommendation of Reviewer 2, we have added the requested analysis of how the experimentally tested ProteinDPO-identified mutations lie with the distribution of mutation effects among baseline methods. We have also identified and corrected errors in the previous analysis which inflated baseline performance, and updated the corresponding figures and text.

2. *Clarified novelty*. In response to Reviewer 3's feedback, we have provided the requested additional clarification on the methodological novelty of ProteinDPO. Specifically, we clarified differences between classes of machine learning models, helping frame an explanation of the methodological novelty of our approach, and how this brings about wider biological utility of ProteinDPO compared to baseline methods such as ThermoMPNN.

Other revisions are detailed individually in our point-by-point response below. These changes address the reviewers' concerns, and reaffirm the core demonstration of our work: namely, that DPO alignment of a biological generative model to a desired experimental property improved the model in stability prediction and stability-biased sequence generation, which was experimentally validated in the setting of influenza hemagglutinin.

## Point-by-point response

### Reviewer 1

The authors have satisfactorily addressed my concerns in this revision.

We are grateful that the reviewer finds our revisions satisfactory, and thank the reviewer for their initial comments which strengthened the rigor of our analysis and accuracy of our claims.

**Reviewer 2**

The authors adequately addressed all of my comments except one small point: I would still like them to analyze and describe whether the stabilizing mutations identified by DPO are predicted to be stabilizing according to the other models. This is different from the top-N analysis performed by the authors. As the authors note, the top-N analysis is insufficient because the other stabilizing mutations predicted by the other method may also be stabilizing. For completeness you might as well just show the full distribution of mutation effects predicted by the other methods (which you have already computed), along with where the successful/unsuccessful mutations lie in the distribution.

as before: "At minimum, the authors should take the top 30 mutants designed by ProteinDPO (the ones that were experimentally tested), examine how these mutants score according to "vanilla" ESM-IF and ThermoMPNN, and report (1) whether the ProteinDPO-designed mutants are predicted to be favorable according to other methods" "

We thank the reviewer for further clarifying their requested analysis. We addressed the remaining request, showing where experimentally-tested mutations designed by ProteinDPO lie within the full distribution of mutation effects of other models. In **Figure S8** (duplicated below) we also report classification metrics (accuracy and F1 score), which directly capture if these mutations are predicted to be stabilizing according to the other models.

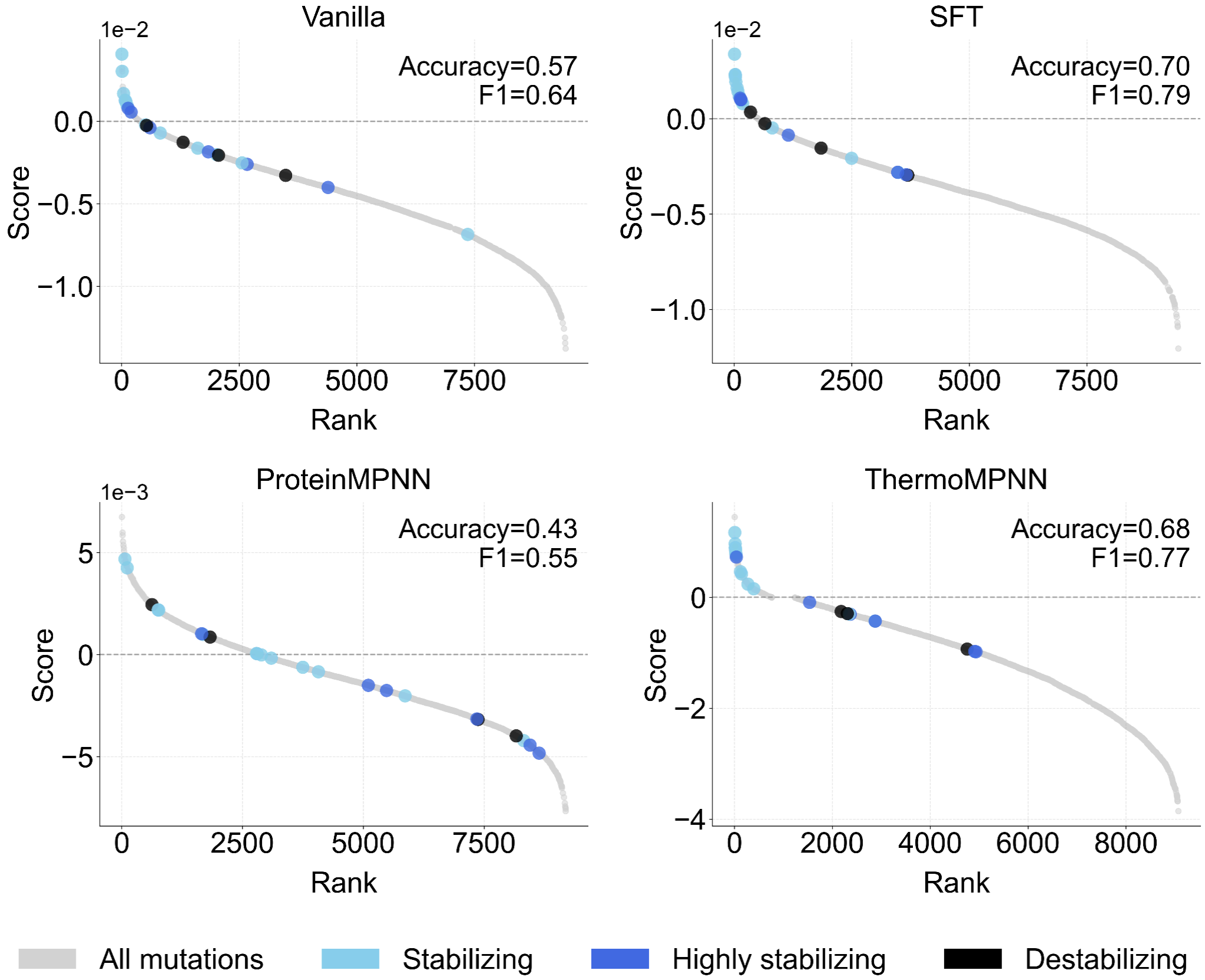


*Figure S8 | Method comparison in placement of stabilizing (light blue) and destabilizing (black) ProteinDPO-identified and experimentally-tested single-substitution variants within the score distributions all possible mutations (gray). Mutations are subset to those which had temperature of thermal unfolding (Tm) that was at least 1 degree higher or lower than the native sequence (N=23/30). Accuracy and F1 scores derived from the models' prediction of a given mutation being favorable or unfavorable is also shown. Additionally, the position of the six significantly stabilizing mutations (dark blue) analyzed in* ***Figure S6*** *are shown.*

Additionally, we have identified and corrected two errors in the previous analysis requested by Reviewer 2. First, in our mutation recovery analysis (**Figures S5 and S6**), all sequence positions were not scored across all baseline models. Second, in **Figure S7**, correlation values were incorrectly calculated. We have updated the figures (duplicated below) and amended the main text.

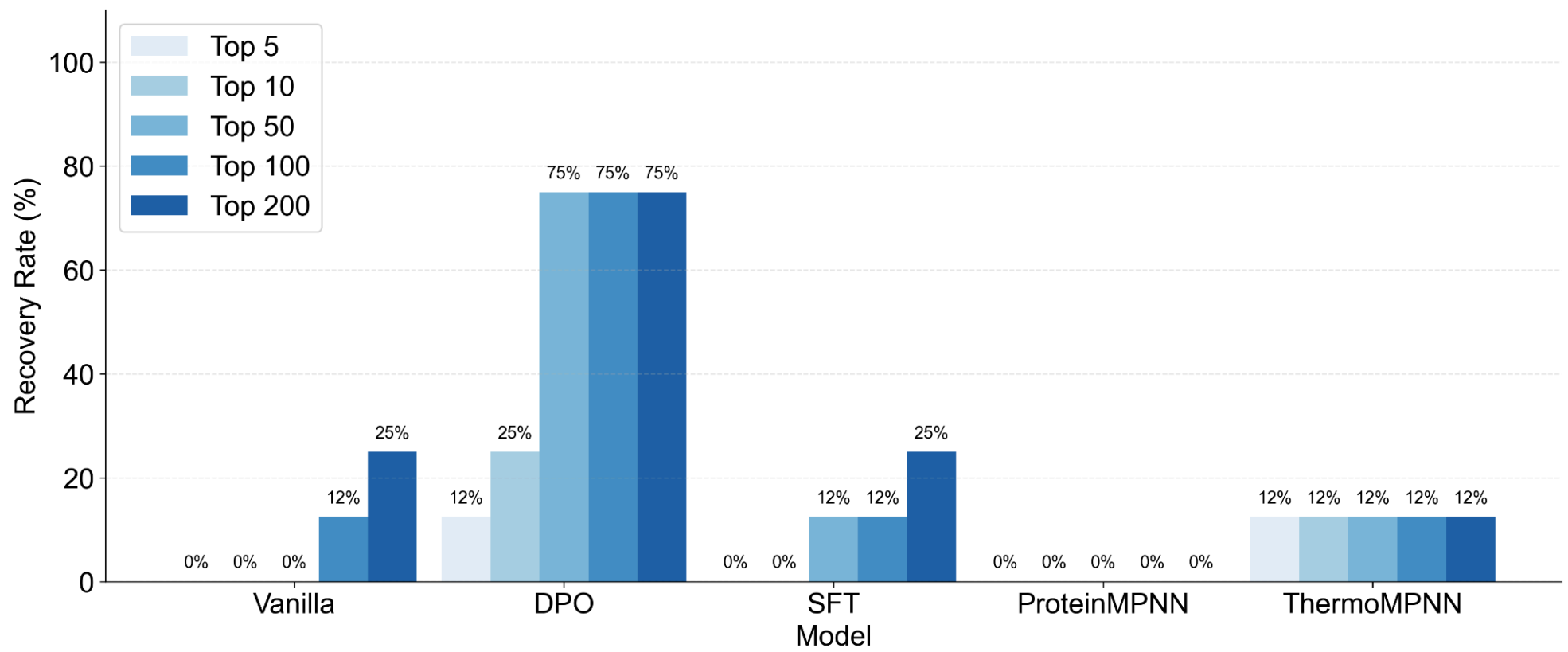


*Figure S5 | Recovery of literature-validated stabilizing mutations across protein design models. Bar plot showing the percentage of eight known stabilizing mutations (Milder et al. and Yassine et al.) recovered within the top-ranked predictions of each model. Models were evaluated using ensemble scoring across three PDB structures (6cf5, 6cfg, 2fk0).*

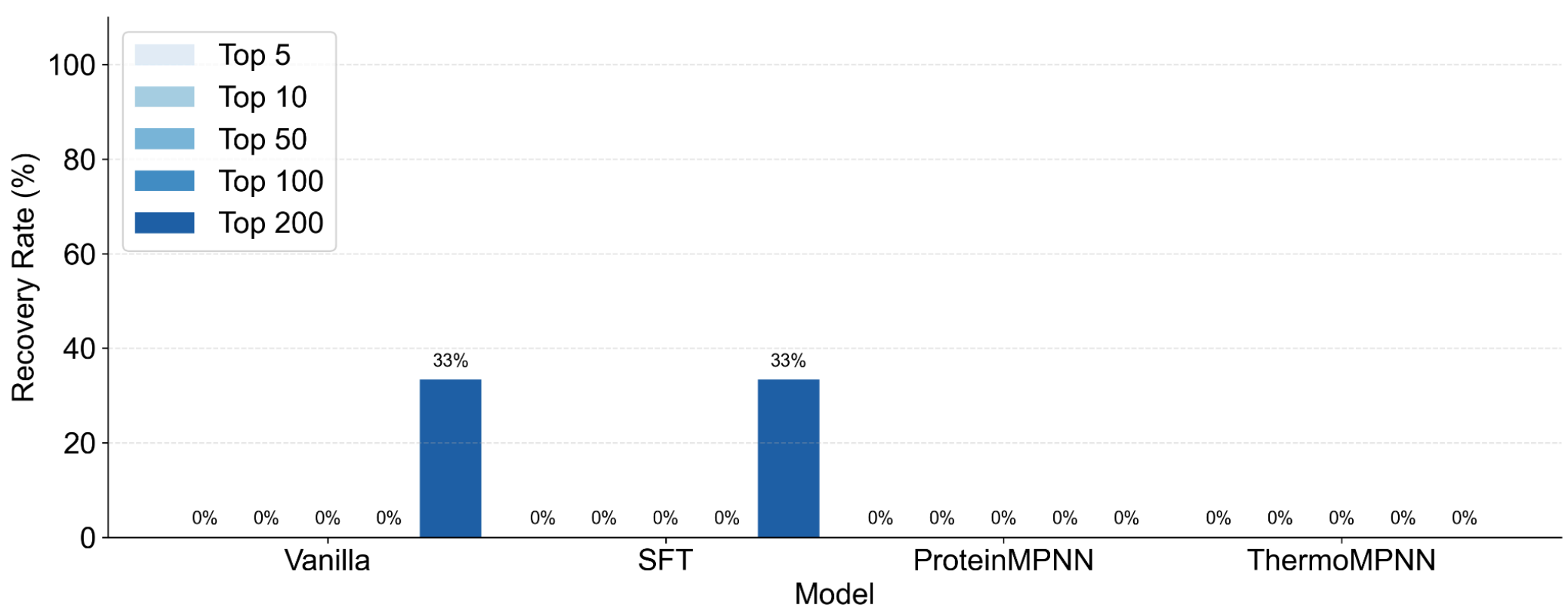


*Figure S6 | Recovery of highly-stabilizing ProteinDPO-identified single-substitutions across protein design models. Bar plot showing the percentage of six significantly stabilizing* (>= 9°C) *DPO-identified mutations recovered within the top-ranked predictions of each model. Models were evaluated using ensemble scoring across three PDB structures (6cf5, 6cfg, 2fk0).*

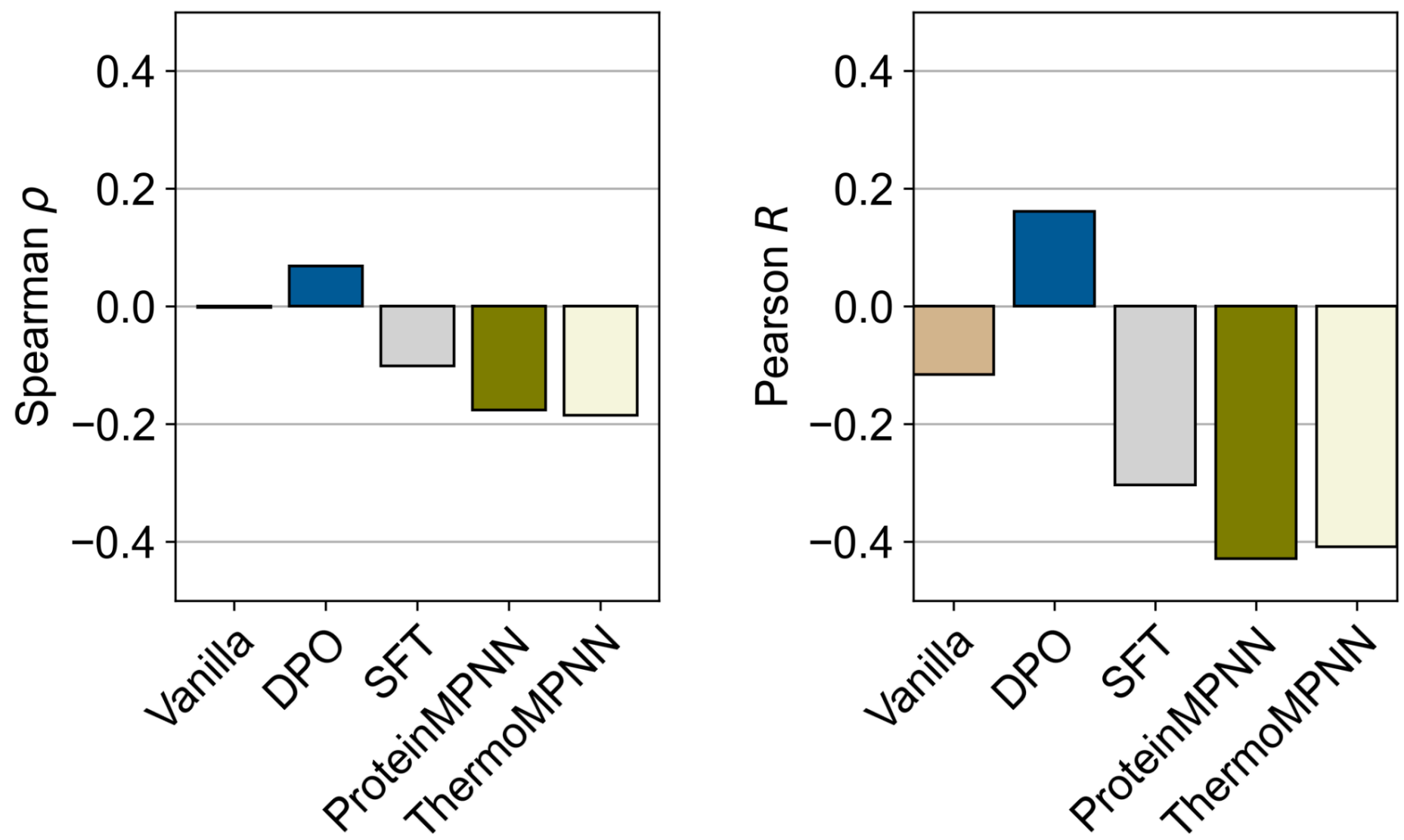


*Figure S7 | Comparison of methods in ability to rank the 30 experimentally tested single-substitution variants identified by ProteinDPO, measured by Pearson R and Spearman ϱ correlation coefficients between model scores and temperature of thermal unfolding (Tm).*

We then reference these result in the **Results** (bolded text indicates additions, strikethrough text indicates deletions):

> *We then conducted a similar analysis as done for literature mutation recovery, but instead comparing models' ability to identify six of the thirty ProteinDPO-identified single-substitution variants which were found to be significantly stabilizing (>= 9°C) (**Figure S6**). Only the SFT ~~model and ThermoMPNN~~ **and Vanilla models** were able to recover ~~one of these~~ mutations in their top-~~1~~200; although, it is possible other top-ranked mutants from these baseline models are also stabilizing. Additionally, **while all models are unable** ~~The SFT model, ProteinMPNN and ThermoMPNN nonetheless show modest ability~~ to rank the thermal stability (Tm) of these 30 variants **(Figure S7), the SFT model and ThermoMPNN show strong ability (F1 score > 0.75) to classify them as stabilizing or destabilizing (Figure S8), despite crucially failing to capture most highly-stabilizing mutations (Figures S6 and S8).***
>
> ***Beyond single-substitutions, and*** ~~*Looking*~~ ***aiming*** *to discover synergistic effects among beneficial mutations, we used ProteinDPO to score….*

**Reviewer 3**

I appreciate the authors' reasonable responses to points 2 and 3. However, I feel that point 1 has not yet been adequately addressed.

My original concern was not delivered clearly, so let me restate it. My comment was not intended to question the general "pretrain-and-fine-tune" strategy itself, but rather to discuss the underlying physical motivation and conceptual foundation of the model. I referred to BioEmu because it is trained on protein dynamics and folding, which are directly relevant to stability estimation and thus provide a clear rationale for transfer learning in this context. In contrast, the pretrained model used for ProteinDPO (ESM-IF) is similar to that of ThermoMPNN (ProteinMPNN), which has not explicitly been trained with such objectives. For this reason, I would group ProteinDPO more closely with ThermoMPNN, while distinguishing it from BioEmu.

My concern is not about the relative performance of the methods — indeed, the differences appear to be marginal based on Figures 2C–E and 3B–C — but rather about the novelty of the approach and its potential for future extension. Adapting a sequence design model for stability prediction has already been explored by ThermoMPNN, albeit in a different formulation, and there may be a general consensus regarding its practical upper bound in performance. Moreover, simply characterizing a model as "generative" does not, by itself, address this limitation. For example, if ThermoMPNN were retrained to preserve the original ProteinMPNN "generation" functionality, it is unclear whether this would be a fundamentally novel approach.

I would therefore suggest to clearly discuss the methodological limitations of the proposed approach in the Discussion section, which may explain its marginal difference to ThermoMPNN.

We appreciate your comments, which will be helpful for clarifying our result. As suggested, we have expanded our **Discussion** section to elucidate the methodological novelty of our approach over ThermoMPNN. These additions fall under addressing two points: (1) the difference between generative models and supervised regressors and (2) how this explains the methodological novelty and broader biological utility of ProteinDPO compared to ThermoMPNN.

1. The reviewer questions "if ThermoMPNN were retrained to preserve the original ProteinMPNN "generation" functionality". In fact, this "retraining to preserve generation" is non-trivial and precisely the contribution of our manuscript. To alleviate this concern, we further clarify the relationship between generative models and supervised regressors, highlighting the importance of retaining generative capabilities.

2. We also note that ProteinDPO has much greater conceptual novelty than ThermoMPNN. Instead of fine-tuning an inverse folding model as a regressor (which is commonly done

> in many computational biology workflows) to predict some aspects of stability, we use reinforcement learning-based alignment (a methodology far less explored) to align the probability distribution of the generative model to both *score and generate* sequences according to preferences determined by experimental data. We then show that the scoring capability of ThermoMPNN becomes a subset of ProteinDPO's capabilities (**Figure 2B,D,E**), though ProteinDPO is strictly more capable across a broader variety of tasks than is ThermoMPNN. Indeed, while there are limited settings where the improvement of ProteinDPO over ThermoMPNN is marginal (**Figure 2C,D**), there are several important and useful biological settings where ThermoMPNN cannot be used. These include absolute stability prediction with a fold, stability-aligned sequence design, multi-chain scoring, and binding prediction (**Figure 3B,C,D**), with ProteinDPO demonstrating a clear improvement that is precisely due to fundamental differences in methodology contributed by our manuscript.

We have updated the second-to-last paragraph in the **Discussion** section to emphasize these two points (bolded text indicates additions, strikethrough text indicates deletions):

> *While the model is competitive with supervised, task-specific ~~models~~* ***regression models*** *such as ThermoMPNN [15], it has not clearly surpassed all supervised models in scoring [12] (**Figure 2B,D,E**).* ***However, using reinforcement learning, unlike supervised regression, preserves essential generative capabilities. This enables ProteinDPO to both score sequences (like regression models) and generate entirely new sequences aligned with desirable functions, which are capabilities that supervised models cannot combine. This generative capability further extends to*** *~~However, ProteinDPO remains a generative model, which enables stability-informed sequence generation for natural or de novo protein folds with autoregressive sampling.~~ ~~ProteinDPO also generalizes to a greater variety of tasks than existing supervised models, being able to predict ΔΔG,~~* ***the*** *prediction of binding affinity~~,~~ and thermal melting across diverse wild-type proteins, multi-chain antibodies, and protein complexes more accurately than the unaligned ESM-IF1 model* ***(Figures 3B,C,D), and the sampling of experimentally-validated nine-substitution variants (Figures 4 and 5).*** *~~This contrasts with models that are limited to scoring single-mutation ΔΔGs of monomeric structures.~~* ***Furthermore,*** *we find that alignment based on one specific property (such as the stability of small monomers) improves performance ~~function prediction for other~~* ***on these*** *diverse tasks (such as binding affinity of large protein-protein complexes), most likely because both properties share common underlying physical principles.*

11/18/2025

**General comments**

We thank all the reviewers for their thorough and constructive feedback on our manuscript. We appreciate the positive reception and are also grateful for their detailed comments which have led to substantial improvements to the clarity, rigor, and detail of our manuscript.

In this revision, we have:

1. *Added more comprehensive benchmarks*. Following reviewer requests, we have included comparisons to other models. Specifically, we now benchmark against ESM-IF1 (both zero-shot and after SFT), ThermoMPNN, and Rosetta for the HA stabilization task **(Fig. S5,6)**, demonstrating that ProteinDPO is superior in recovering literature mutations, while other models are not able to identify highly stabilizing variants from the literature or from our validated experimental pool. We have also added ProteinMPNN benchmarking for protein-protein binding **(Fig. 3B)** and RosettaREU evaluation for antibody thermal stability **(Fig. 3C)**, finding ProteinDPO has competitive or improved performance compared to benchmark models.

2. *Toned down claims*. We have carefully revised statements throughout the manuscript to ensure accurate representation of our results. This includes altering our claims with respect to vaccine design and clarifying that absolute stability predictions refer to relative improvements within a protein fold rather than absolute predictions across different folds. We have also added appropriate caveats regarding the limitations of computational metrics like Rosetta energy, and clarified that certain redesigned sequences in **Fig. 3** were not experimentally verified.

3. *Added more methodological details on our implementation and evaluations*. We have substantially expanded the **Methods** section to improve clarity. This includes new subsections for Model sampling, Sampling evaluation, Rosetta generation evaluation, and Scoring metrics **(Methods 6.6)**, detailed pseudocode for all training algorithms including Paired, Ranked, and Weighted DPO as well as SFT training **(Methods 6.7)**, and a new Mutation selection section describing the multi-mutation design protocol **(Methods 6.8)**. We have also added distributional plots for key results **(Fig. S3, S4)**, clarified dataset versions used, improved experimental methods descriptions, and fixed installation issues. In addition to pseudocode in the Methods, we attach the raw training code used for ProteinDPO as a supplementary zip file (**Supplementary Code**).

Other revisions are detailed individually in our point-by-point response below. These revisions address the reviewers' concerns while demonstrating the core contributions of our work: (1) that DPO can be used to align biological generative models with a desired experimental property and (2) our aligned model, ProteinDPO, improves upon its unsupervised predecessor to be useful as a stability predictor and stability-biased sequence generator, which we experimentally validate in the setting of influenza hemagglutinin.

## Point-by-point response

### Editorial comments

We recommend adding more comprehensive benchmarking against available methods in this field as well as addressing all the technical concerns. Regarding Reviewer #1's point 2 (vaccine relevance) - additional experiments to demonstrate that designed variants elicit protective immune responses against the target virus would be out of scope for us, so we recommend you tone down these claims.

We thank the editor for recognizing the interest in our work from the reviewers, and providing us the opportunity to improve our manuscript. To address the concern of comprehensive benchmarking, we have implemented the reviewers' suggestion of benchmarking against ProteinMPNN and Rosetta for binding and stability prediction (in addition to previous analyses benchmarking against both base and SFTed ESM-IF1), as well as benchmarking against a range of baseline models for the experimental application of hemagglutinin stabilization. We appreciate the guidance regarding vaccine relevance, and have toned down these claims accordingly. For example, in the **Abstract**:

*"Leveraging up to nine substitutions, we achieved a 17°C improvement in melting temperature on a HA from human H5N1 influenza A virus while preserving ~~antigenicity~~ **recognition by existing antibodies**, and up to 32°C stability improvements across recently emerged mammalian viral H5 HAs."*

As well as in the **Results** section:

*"To determine whether ProteinDPO-identified mutations ~~compromise the antigenic properties required for vaccine efficacy~~ **maintain key epitopes recognized by existing antibodies**, we measured the binding affinity of HA variants with nine substitutions to anti-HA broadly neutralizing antibodies (bnAbs) targeting either the head or the stem domains of HA via biolayer interferometry (BLI).*

And added an additional statement in the **Discussion** section:

*"Moreover, while pre-fusion stabilization and retention of binding to native antibodies are key components of vaccine design, further evidence would be needed to demonstrate antigenic properties required for vaccine efficacy"*

**Reviewer 1**

In this work, the authors introduce an approach called Direct Preference Optimization (DPO) to enhance desired properties of protein sequences generated by computational models. Specifically, they focus on protein stability using the inverse protein folding model ESM-IF1, and further optimize the model with protein mutational data. The authors report improvements in several benchmark tests compared with the original unsupervised ESM-IF1 and a fine-tuned supervised model, and in some cases, with another approach named ThermoMPNN. Finally, they apply the method to design hemagglutinin variants for influenza vaccines, reporting increased stability. The study presents an interesting strategy for optimizing deep learning models in protein design. However, I am not fully convinced that the proposed approach provides a significant advantage based on the current evidence. My major concerns are outlined below:

We thank the reviewer for recognizing the merits of our work, and include a point-by-point response below to address their major concerns of our method.

1. Lack of comparison in the HA application. While benchmark improvements are demonstrated, the application to HA design, arguably the most important example in this work, was not compared with other existing approaches. Since ESM-IF1 itself has shown some promising performance in similar tasks, it remains unclear how much Protein DPO actually improves upon it. A detailed comparison with alternative methods is necessary to convincingly establish the benefit of Protein DPO.

We thank the reviewer for raising this concern. On the task of HA stabilization, we now benchmark against ESM-IF1, SFT, ThermoMPNN, and ProteinMPNN in these models' ability to highly rank stabilizing mutations (1) from the literature and (2) out of the 30 single-mutations which were experimentally validated in our first revision, in part following the guidance of Reviewer 2. When comparing the ability of ESM-IF1, SFT, ThermoMPNN, ProteinMPNN and ProteinDPO to recover known HA-stabilizing mutations from the literature, specifically Milder et al [9] and Yassine et al [15] **(Fig. S5)**, we found that while some models were able to recover one mutation in their top-50, ProteinDPO has a higher recovery rate across all top-n levels.

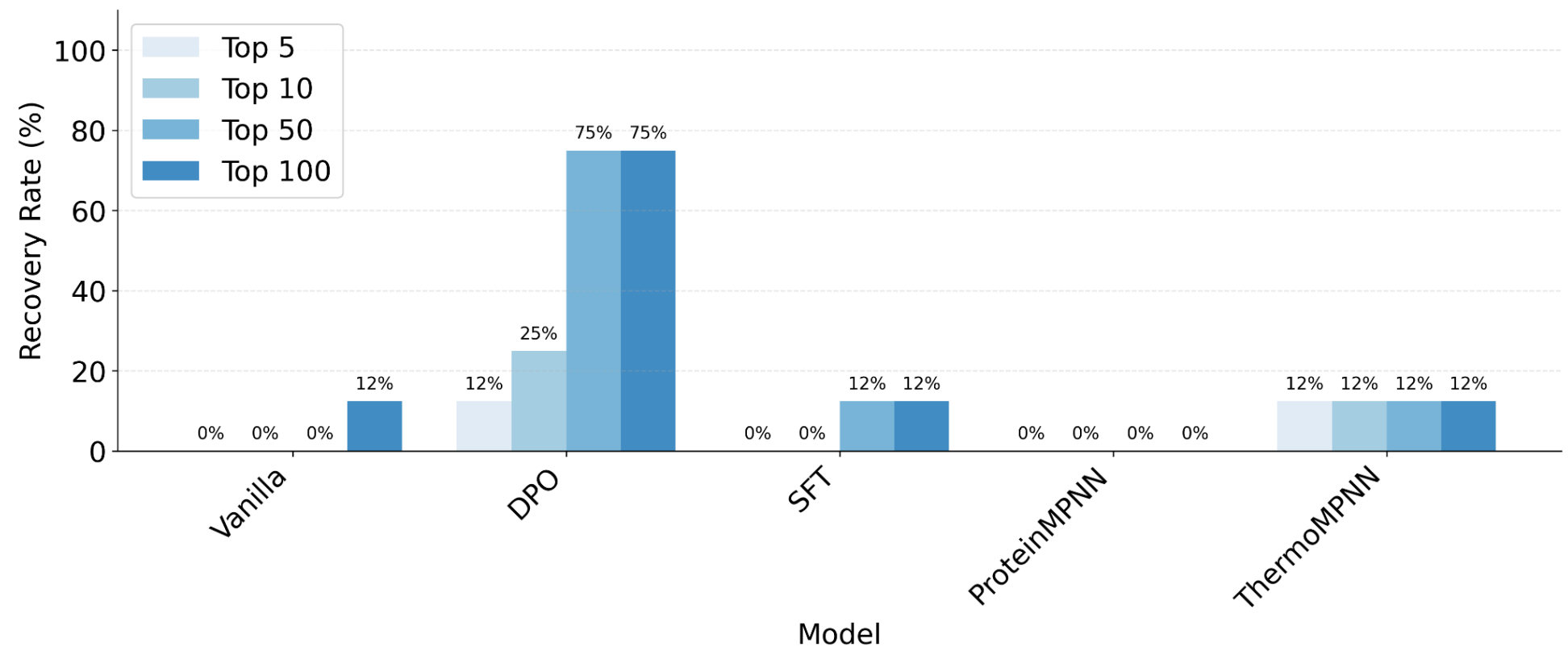


*Figure S5 | Recovery of literature-validated stabilizing mutations across protein design models. Bar plot showing the percentage of eight known stabilizing mutations (Milder et al. and Yassine et al.) recovered within the top-ranked predictions of each model. Models were evaluated using ensemble scoring across three PDB structures (6cf5, 6cfg, 2fk0). n=8 total literature mutations.*

Additionally, we compared the ability of ESM-IF1, SFT, ProteinMPNN and ThermoMPNN to rank the 6/30 highly-stabilizing (>= 9°C) mutations from the pool of 30 single-mutations we tested **(Fig. S5)**. Only the SFT model and ThermoMPNN were able to recover one of these mutations in its top-100.

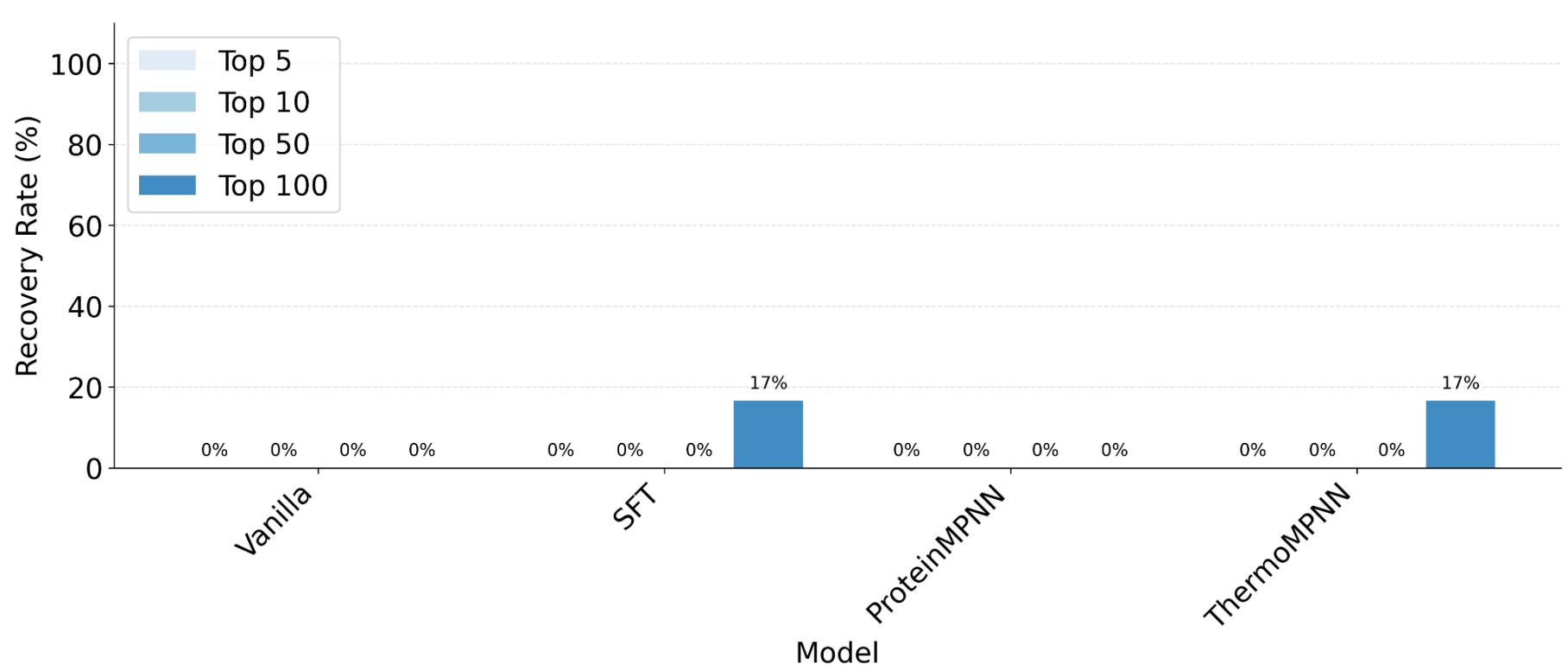


*Figure S6 | Recovery of experimentally tested DPO mutations by baseline models. Bar plot showing the percentage of six significantly stabilizing (>= 9°C) DPO-identified mutations recovered within the top-ranked predictions of each model. Models were evaluated using ensemble scoring across three PDB structures (6cf5, 6cfg, 2fk0).*

Both analyses suggest that ProteinDPO is superior in highly ranking literature mutations, enabling low-n stabilization. Additionally the compared methods are not able to recover most of the best performing ProteinDPO mutations. We do note that a limitation of this test is that some of the highly ranked mutations by other methods may be stabilizing, which we also acknowledge in our text:

*'Only the SFT model and ThermoMPNN were able to recover one of these mutations in its top-100; however, it is possible top-ranked mutants from these baseline models are also stabilizing."*

2. Vaccine relevance not fully established. For vaccine design, the key requirement is demonstrating that designed variants elicit protective immune responses against the target virus. The manuscript shows recognition of designed variants by existing antibodies, but this does not sufficiently demonstrate antigenic properties required for vaccine efficacy. Stronger experimental validation would be needed to support claims of relevance to vaccine design.

We thank the reviewer for raising this concern. We agree that the results presented do not conclusively demonstrate vaccine efficacy. Considering the following guidance from the editor:

*"additional experiments to demonstrate that designed variants elicit protective immune responses against the target virus would be out of scope for us, so we recommend you tone down these claims"*

We toned down the implications of existing antibody recognition in the **Abstract**:

*"Leveraging up to nine substitutions, we achieved a 17°C improvement in melting temperature on a HA from human H5N1 influenza A virus while preserving ~~antigenicity~~ **recognition by existing antibodies**, and up to 32°C stability improvements across recently emerged mammalian viral H5 HAs."*

as well as in the **Results** section:

*"To determine whether ProteinDPO-identified mutations ~~compromise the antigenic properties required for vaccine efficacy~~ **maintain key epitopes recognized by existing antibodies**, we measured the binding affinity of HA variants with nine substitutions to anti-HA broadly neutralizing antibodies (bnAbs) targeting either the head or the stem domains of HA via biolayer interferometry (BLI).*

and added an additional statement in the **Discussion** section:

*"Moreover, while pre-fusion stabilization and retention of binding to native antibodies are key components of vaccine design, further evidence would be needed to demonstrate antigenic properties required for vaccine efficacy"*

3. Potential overemphasis on stability. The importance of stability may be overstated. For example, Figure 3D shows that the model generates sequences with improved stability according to Rosetta energy functions, but it is unclear whether these sequences are experimentally synthesized. Extremely stable sequences often risk aggregation due to excess hydrophobic residues, which may hinder practical expression and use. Moreover, the sequence design procedure is insufficiently described in the Methods. Was it simply initiated from random seeds, as in ESM-IF1?

We agree the Rosetta evaluation places an overemphasis on stability and needs clarification that these sequences were not experimentally tested. To address the former, we have added this clarification to the text:

*"Although Rosetta energy can be inaccurate and is not a substitute for experimental testing, it is a widely used independent evaluator of machine learning generations due to its reliance on physics-based parameters rather than shared training data with machine learning models [44, 5]"*

To address the latter, we added this clarification:

*"While these sequences were not experimentally verified to express, …."*

Additionally, we added a **Model sampling**, **Sampling evaluation**, and **Rosetta generation evaluation** sub-sections to the methods (**Methods 6.6**) to clearly describe the sampling procedure and evaluation for this experiment.

4. In Section 2.3, the claim that the method can rank "absolute stability of diverse antibodies" seems misleading, since only correlation coefficients are reported. These results reflect relative stability rankings, not absolute values. Furthermore, comparisons across antibodies likely rely on their shared fold and similar length. It is doubtful that the approach could be applied to rank melting temperatures across proteins of different folds.

We thank the reviewer for bringing up this concern. Being able to rank stability *within* a specific protein fold is what we intended to convey with this evaluation. We have made the following clarifications to the main text, only mentioning the term absolute when referring to ranking within a given fold:

*"we sought to evaluate if ProteinDPO can use whole-sequence reasoning to rank absolute stability of ~~diverse antibodies~~* ***sequences within a given fold, particularly antibodies"***

*"on the ability of vanilla ESM-IF1 in ranking the ~~absolute~~ differences in thermal melting points"*

*"ProteinDPO generalizes to scoring binding affinity of large complexes and ~~absolute antibody~~ thermal stability* ***of highly diverse and variable length antibodies"***

5. The Methods section needs clarification and expansion, particularly regarding benchmark evaluation. A separate subsection should clearly describe the metrics used (e.g., correlation coefficients), how they are computed, and the specific model outputs used for evaluation. In addition, figures such as 2B and 3B would benefit from showing distributional statistics rather than only averages, as mean values can obscure important variation.

We thank the reviewer for raising this concern regarding clarification in the methods section. To alleviate this we have added a sub-section, **Scoring metrics**, to the methods **(Methods 6.6)** to clearly describe the metrics used, including each of the coefficients. In this new text we point to the **Datasets** section **(Methods 6.5)** where one can find the specific model outputs used for evaluation.

Additionally, we added distributional plots for 2B and 3B **(Fig. S3, S4)** including for the newly benchmarked ProteinMPNN, and reference them in the main text:

*"Distributional plots of the individual values are included in the supplement* ***(Fig. S3)****."*

*"Distributional plots of the individual values for the AB-Bind dataset are included in the supplement* ***(Fig. S4)****."*

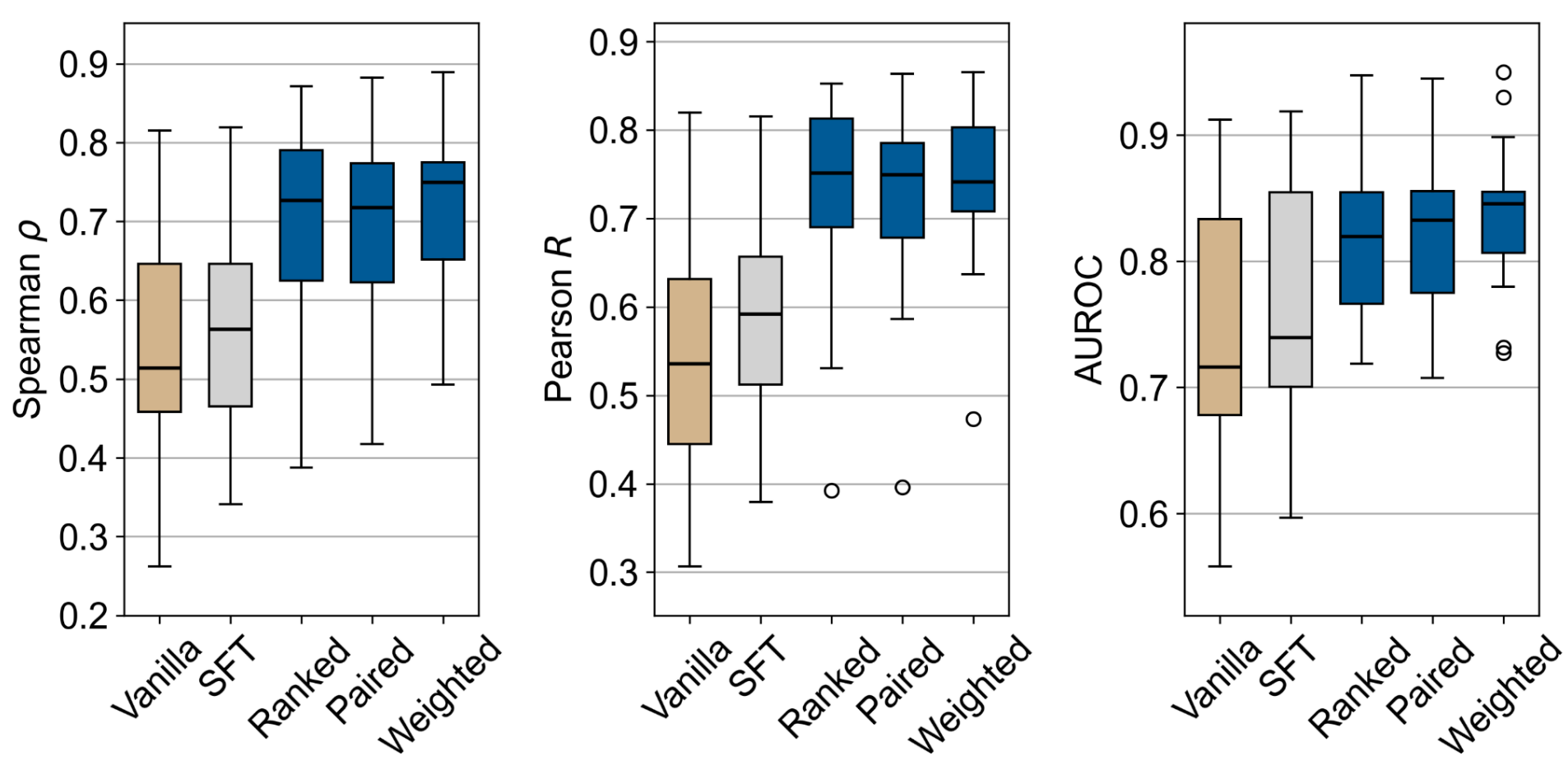


*Figure S3 | Distribution of performance across protein domains for predicting stability changes upon mutation (ΔΔG) in curated Megascale holdout dataset.*

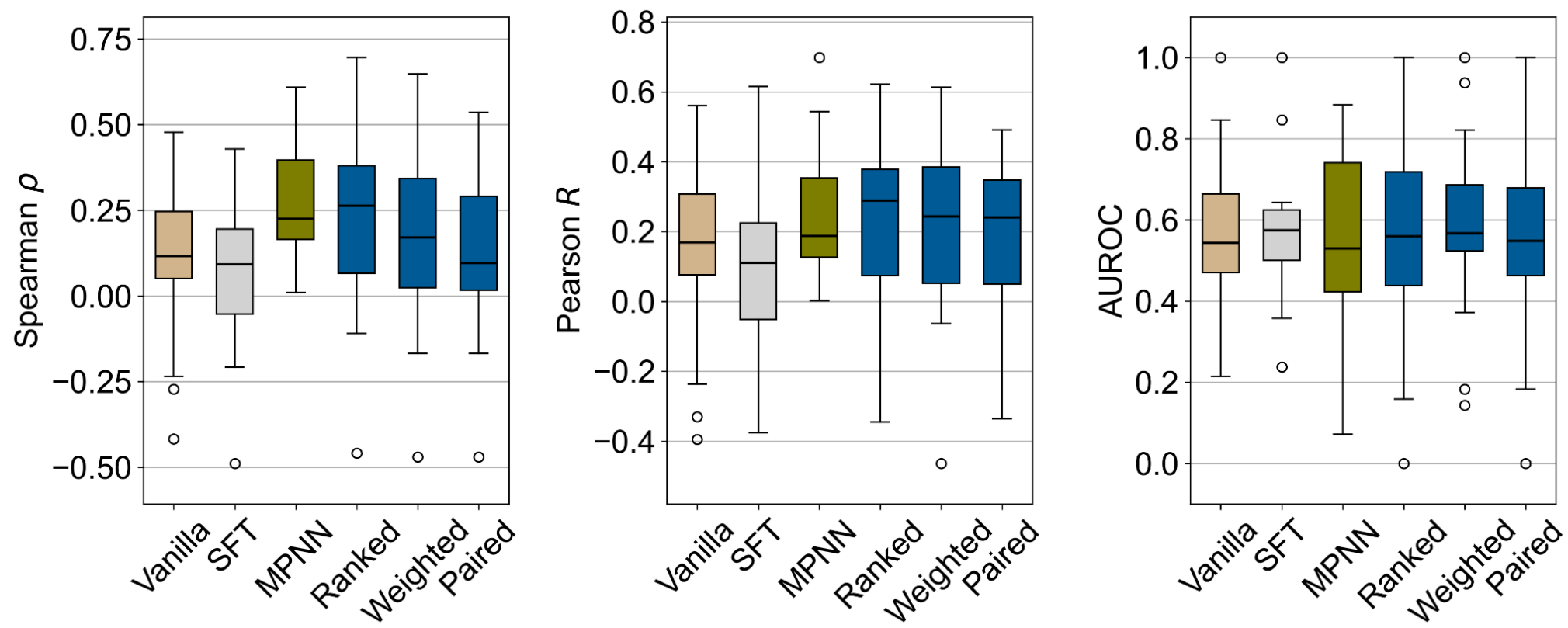


*Figure S4 | Distribution of performance across antibody-antigen complexes for predicting binding affinity changes upon mutation in AB-Bind dataset.*

6. The main contribution of this work lies in the proposed algorithm for optimizing ESM-IF1, but the Methods provide insufficient implementation details. Moreover, the GitHub repository only contains scripts and weights for inference, without the training code or implementation necessary for reproduction. At minimum, the authors should provide pseudocode for their approach—particularly for the weighted DPO algorithm, which is a novel aspect of this study.

We thank the reviewer for raising this concern. We have now included in-depth pseudocode for the approach. We have added algorithms for training the Paired, Ranked, and Weighted DPO models, as well as SFT training and log-likelihood scoring. All of these have been added to the **Model training** section in the Methods **(Methods 6.7)**. We have also added our training code for the model as **Supplementary Code**.

**Reviewer 2**

This paper applies the Direct Preference Optimization (DPO) approach to "align" the structure-conditioned protein language model ESM-IF with the objective of designing high stability protein sequences. The authors point out that the "base" model ESM-IF is trained to recreate the probability distribution of the training data, which does not match the common objective in protein design to produce high stability proteins. The DPO method improves the alignment between the distribution sampled by the model and the author's (and community's) design objective of sampling high stability proteins. The resulting model (ProteinDPO) can be used for both scoring structures and for generating new sequences based on an input protein backbone. The authors examine several variants of ProteinDPO, computationally benchmark ProteinDPO on several design and ranking tasks against other leading models, and also apply ProteinDPO to experimentally design vaccine antigens with higher stability.

The main strengths of the paper are:

-The problem of improving generative AI models for protein design is timely and important and the approach of using DPO based on experimental folding stability data is relatively novel.

-The flexiblity of ProteinDPO enables the model to be applied to score multi-mutation variants as well as entire unrelated structures, which is rare in the field of stability prediction. The model achieves superior performance over a naive application of ThermoMPNN at predicting effects of multiple mutants (Fig. 2C). The model also shows a modest correlation with antibody binding and melting temperature (Fig 3B/C), which is superior to ESM-IF ("Vanilla") and cannot achieved using models like ThermoMPNN or MAESTRO that are designed to score single mutants.

-For predicting single mutants, several variants of ProteinDPO have strong performance at the S669 benchmark and come close to matching ThermoMPNN at the Fireprot benchmark.

-Using a computational benchmark, the authors show that ProteinDPO-based protein design improves on ESM-IF based protein design according to the Rosetta energy functon(Fig. 3D).

-The experimental work demonstrates a successful application of ProteinDPO. However, this work is seemingly not intended to illustrate the superiority of ProteinDPO over other methods- the proposed mutants are not computationally evaluated using any method other than ProteinDPO.

We thank the reviewer for appreciating and highlighting the strengths of our work, particularly the need for improving protein generative models via alignment, the flexibility of the resulting model for stability prediction, and the successful experimental application.

The main weaknesses of the paper are:

1. Although the authors make the case that ProteinDPO has unique applications beyond other stability models (such as Fig. 2C, Fig 2B, Fig 3C), the experimental demonstration doesn't really highlight these applications. Instead, it illustrates an application where other stability models might have performed equally well or better.

We thank the reviewer for bringing up this concern. While we were interested in addressing these unique applications (binder design, backbone stabilization, antibody stabilization), in efforts of balancing model validation and finding a relevant practical application, we decided hemagglutinin stabilization was an especially interesting choice. We also note that ProteinDPO readily generalizes to multi-mutation scoring and note that this was tested in our experimental work **(Fig. 2C)**. While several substitutions in the multi-mutation variants were derived from single-mutants verified as stable from the initial round of 20 mutations (labeled DPO-A in **Table S6**), for many of the 5-substitution and 9-substitution variants, ProteinDPO included substitutions that were never previously evaluated as singletons, instead using its likelihood function to evaluate a combined score and demonstrating multi-mutation variant generation. We provide further detail on the multi-mutation selection protocol in a section titled **Mutation selection** in the Methods section **(Methods 6.8)**. Additionally, the HA structure is a homo-trimeric complex, and explicit scoring of multi-chain complexes is inaccessible to many stability models, including ThermoMPNN.

2. The authors don't examine the performance of other stability models at their experimental demonstration. It would be very useful to the audience to know whether the output of ProteinDPO is unique or similar to predictions made by other stability models. At minimum, the authors should take the top 30 mutants designed by ProteinDPO (the ones that were experimentally tested), examine how these mutants score according to "vanilla" ESM-IF and ThermoMPNN, and report (1) whether the ProteinDPO-designed mutants are predicted to be favorable according to other methods, (2) whether they also rank near the top of all mutants evaluated by other methods, and (3) whether other methods show an ability rank mutants by Tm within the top 30 designed by ProteinDPO. It would also be good to note whether ProteinDPO itself can rank these 30 mutants, although this is a somewhat unfair task since they are all already highly favorable according to ProteinDPO.

We are grateful for the set of recommended tasks, which we have performed and were also helpful in responding to a question from Reviewer 1. For HA stabilization, we benchmarked

against ESM-IF1, SFT, ThermoMPNN, and ProteinMPNN in these models' ability to highly rank stabilizing mutations (1) from the literature and (2) out of the 30 single-mutations which were experimentally validated in our first revision. For known HA-stabilizing mutations from the literature, specifically from Milder et al [9] and Yassine et al [15] **(Fig. S5)**, ProteinDPO has a much higher recovery rate across all top-n levels.

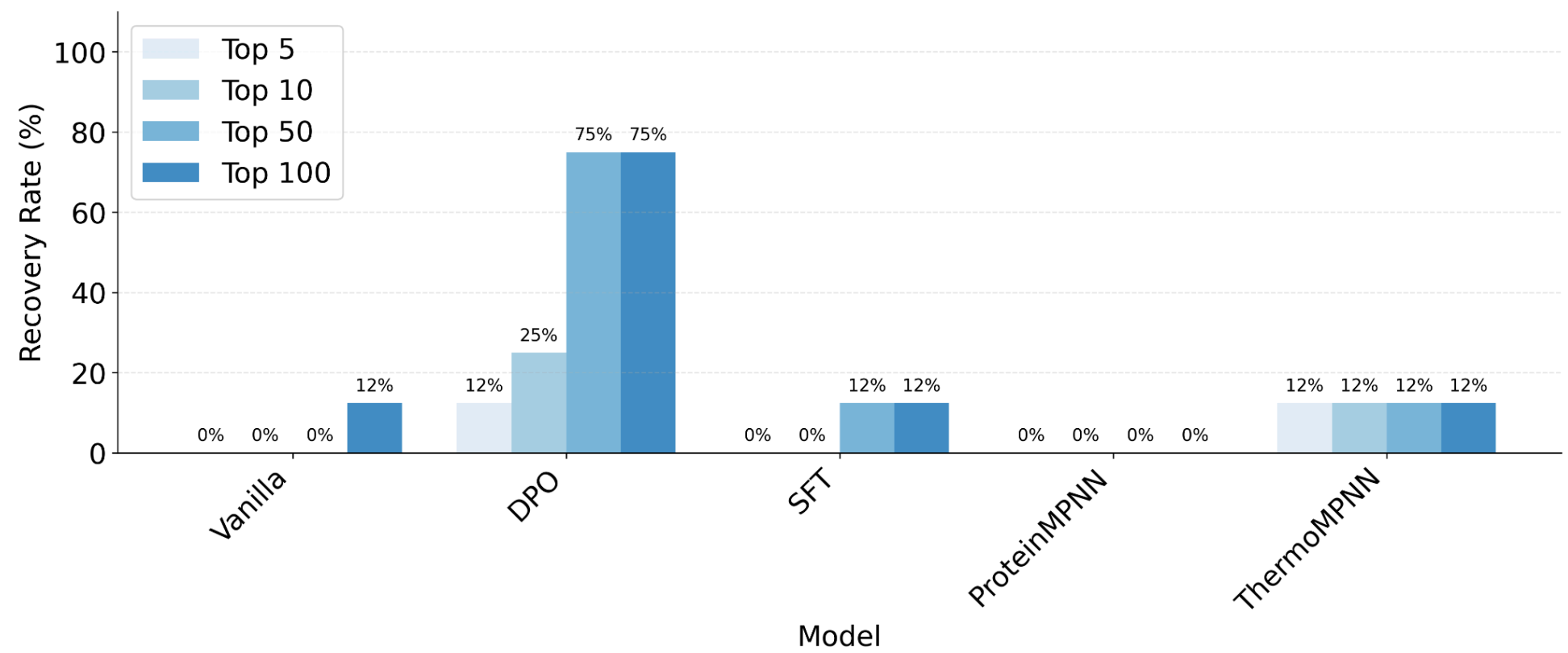


*Figure S5 | Recovery of literature-validated stabilizing mutations across protein design models. Bar plot showing the percentage of eight known stabilizing mutations (Milder et al. and Yassine et al.) recovered within the top-ranked predictions of each model. Models were evaluated using ensemble scoring across three PDB structures (6cf5, 6cfg, 2fk0). n=8 total literature mutations.*

Additionally, we compared the ability of ESM-IF1, SFT, ProteinMPNN and ThermoMPNN to rank the 6/30 highly-stabilizing (>= 9°C) mutations from the pool of 30 single-mutations we tested **(Fig. S5)**. Only the SFT model and ThermoMPNN were able to recover one of these mutations in its top-100.

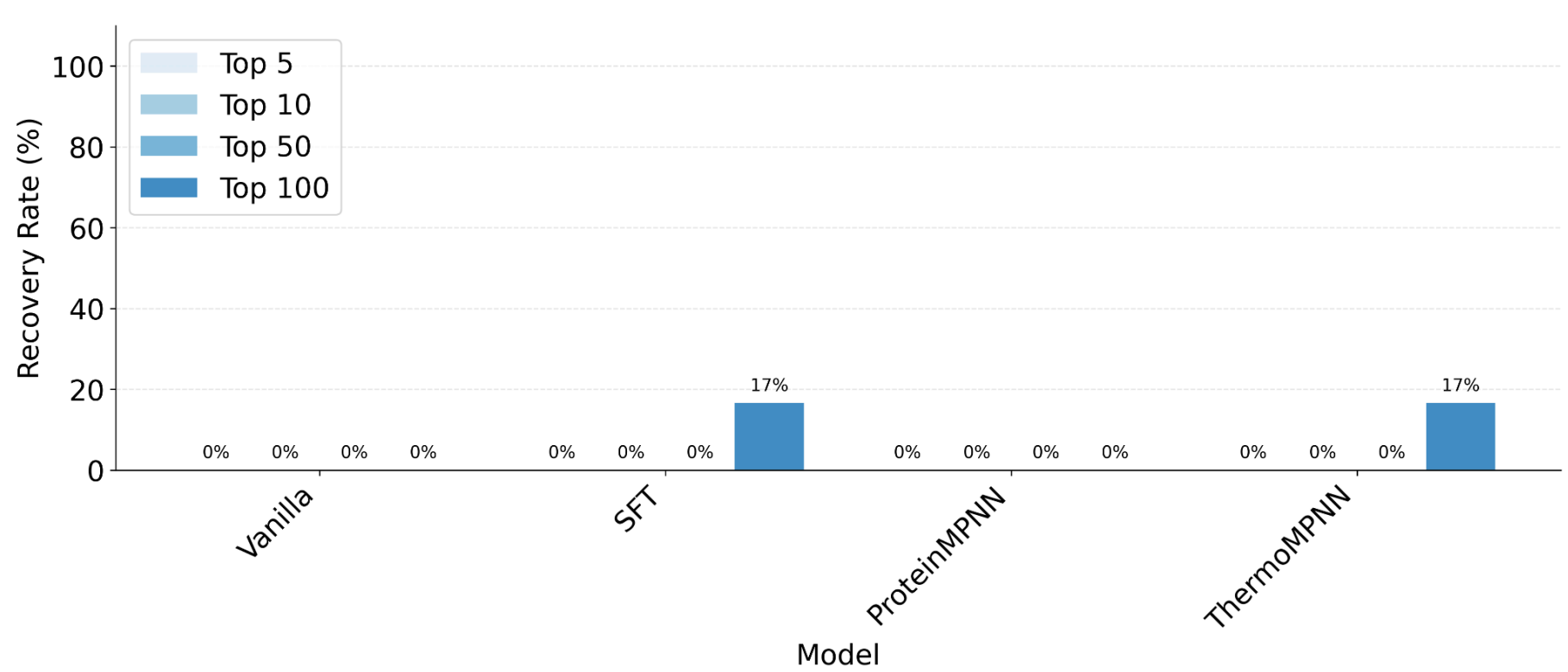


*Figure S6 | Recovery of experimentally tested DPO mutations by baseline models. Bar plot showing the percentage of six significantly stabilizing (>= 9◦C) DPO-identified mutations recovered within the top-ranked predictions of each model. Models were evaluated using ensemble scoring across three PDB structures (6cf5, 6cfg, 2fk0).*

Both analyses suggest that ProteinDPO is superior in highly ranking literature mutations within its top-n mutations, enabling low-n stabilization. We do note that a limitation of this test is that some of the highly ranked mutations by other methods may be stabilizing, which we also acknowledge in our text:

*'Only the SFT model and ThermoMPNN were able to recover one of these mutations in its top-100; however, it is possible top-ranked mutants from these baseline models are also stabilizing."*

We also conducted the third analysis that evaluates "whether other methods show an ability to rank mutants by Tm within the top 30 designed by ProteinDPO." As an important caveat, as expected, ProteinDPO cannot rank its own top-30 mutants, as these are all extremely high-likelihood mutations coming from the top ~0.3% of all possible mutations. The vanilla model, SFT and ProteinMPNN show modest ability to do so, with ThermoMPNN, being the only model trained on stability prediction, performing the best. We include the results of this analysis in **Fig. S7**:

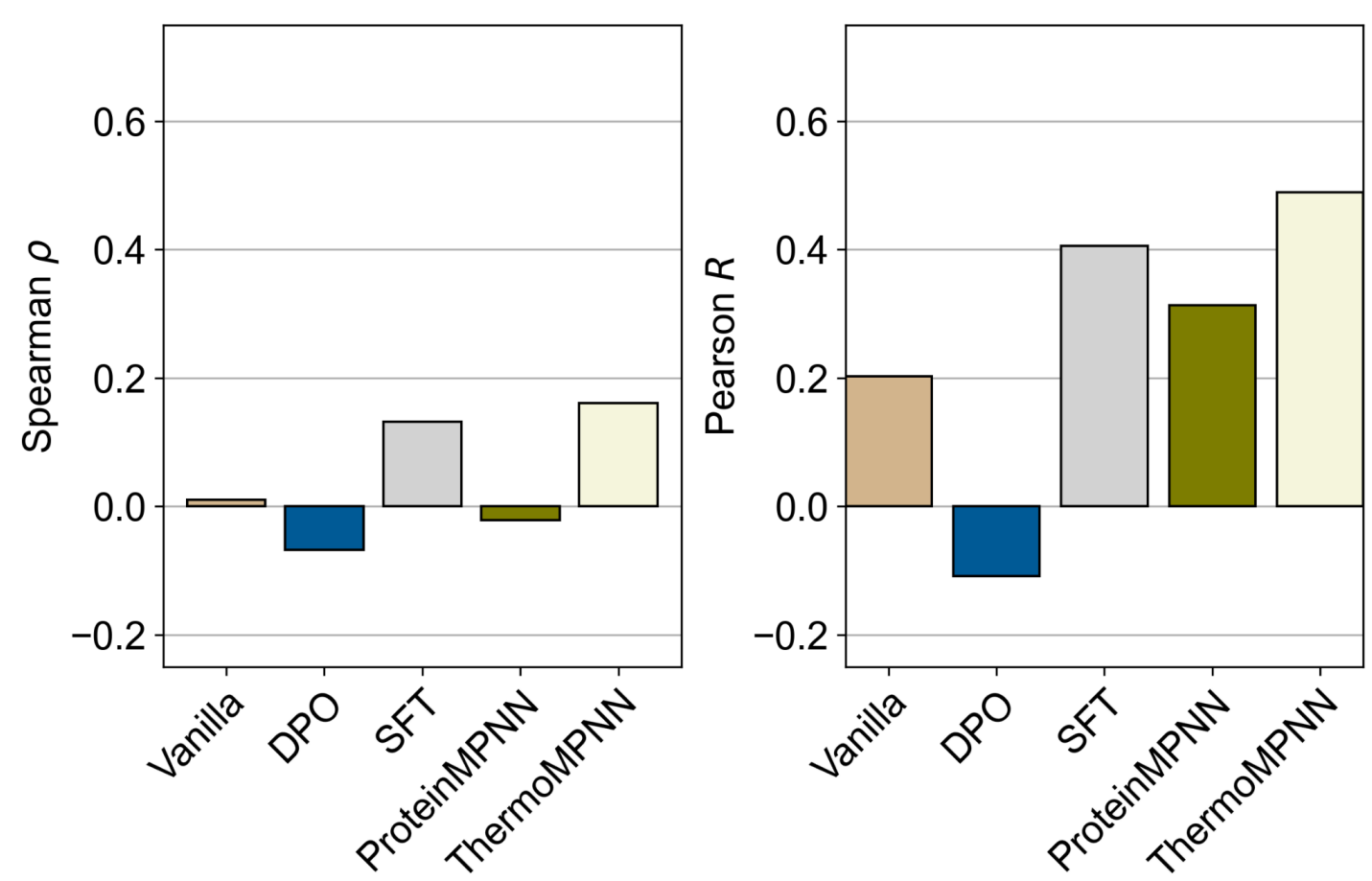


*Figure S7 | Comparison of methods in ability rank the 30 experimentally tested single-substitution variants identified by ProteinDPO, measured by Pearson R and Spearman ρ correlation coefficients between model scores and temperature of thermal unfolding (Tm).*

We also refer to these results in the main text:

*The SFT model, ProteinMPNN and ThermoMPNN do however show modest ability to rank the thermal stability (Tm) of these 30 variants* ***(Fig. S6)****.*

3. The benchmarking of ProteinDPO against other models for antibody melting temperature and protein-protein binding is somewhat minimal. Only ESM-IF is used as a benchmark. Are any

other benchmarks available? At minimum, RosettaREU (total energy, energy per residue, or ddG) could be used, as in Fig 3D.

We thank the reviewer for pointing out the need for additional benchmarking, and for recommending methods to utilize. To address this concern, we evaluated RosettaREU on the antibody thermal stability benchmark, finding that using total energy, rather than energy per residue is a superior metric. We have added this benchmark to the main text **(Fig. 3C).**

For the protein-protein binding benchmark, despite extensive effort, we found it very challenging and computationally intensive to use Rosetta for protein-protein binding evaluation. Naively relaxing the complexes with the mutations applied and extracting a ddG by difference in energy yielded very poor results (and resulted in several PDB and mutation processing errors); we then attempted to use the recommended flex_ddg protocol, which yielded similar errors and would also have taken an estimated 500,000 CPU hours for the ~8500 mutations in SKEMPIv2 and AB-Bind under recommended settings, which was not feasible for us to run in a reasonable timeline. Instead, we have added ProteinMPNN as another baseline method for the protein-protein binding benchmark (**Fig. 3B)** [4], given it is a widely used tool for binder design [1, 11] and the comments by Reviewer 3 asking us to use ProteinMPNN as a benchmark comparison. Our updated **Fig. 3** is provided below:

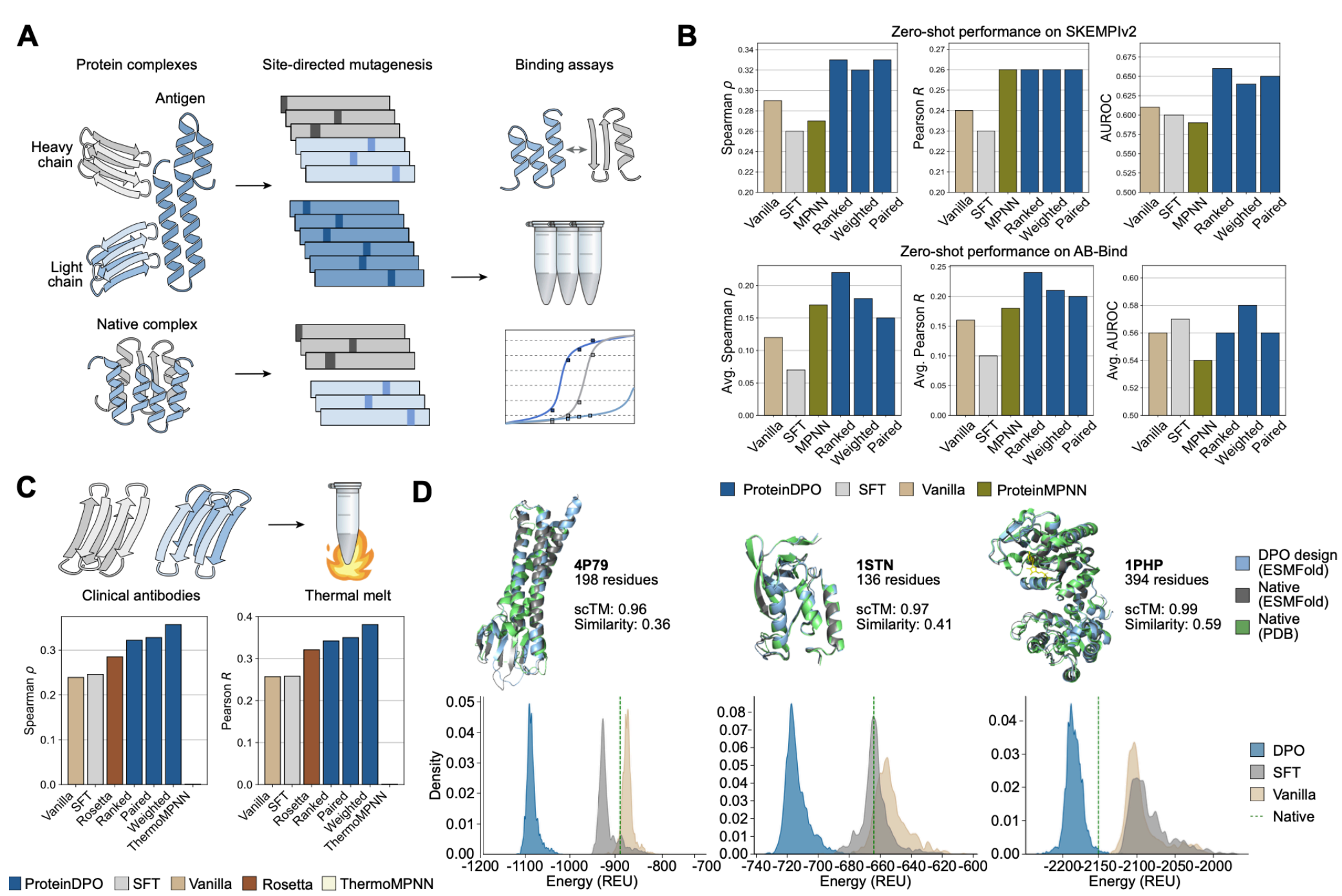


4. The authors need to be careful about how they describe the overall accuracy of the ProteinDPO method. For example, in the 4th paragraph of the discussion, the authors write

"ProteinDPO also generalizes to a greater variety of tasks than existing supervised models, being able to predict ΔΔG, binding affinity, and thermal melting across diverse wild-type proteins, multi-chain antibodies, and protein complexes." This ability to "predict binding affinity" is based on spearman rho values of 0.2 - 0.33 - poor overall. The ability to predict thermal melting is based on spearman rho of ~0.35 - again poor. It would be accurate to say that the ProteinDPO model can predict these quantities ***more accurately than the vanilla ESM-IF model***, which is a worthwhile computational achievement. But the authors need to avoid unqualified statements like saying "ProteinDPO can predict X".

We thank the reviewer for raising this concern and agree with these comments. For the noted cases, we have described these results as “modest”:

*“ProteinDPO has modestly increased scoring of the binding affinity of large protein-protein complexes”*

And have also revised several statements in the text:

*“Notably, the aligned model also performs well in domains beyond its training data to enable stabilization and **improved** binding affinity prediction of large multi-chain proteins.”*

*“and thermal melting across diverse wild-type proteins, multi-chain antibodies, and protein complexes **more accurately than the unaligned ESM-IF1 model.”***

***“**ProteinDPO generalizes to **improved** ~~scoring~~ binding affinity **scoring** of large complexes and thermal stability of highly diverse and variable length antibodies**,”***

5a. It's not correct to say that FireProt and S669 are made from deep mutational scanning datasets since these are generally piecemeal individual measurements, not multiplexed comprehensive measurements.

Thank you for this comment. We have made this clarification in the text.

*“We benchmarked models using ~~deep mutational scanning (DMS)~~ **experimental protein stability** dataset FireProt and S669. **These datasets are compiled from small-scale** c~~ompiled from~~ mutagenesis experiments across diverse monomeric proteins, in which certain residues are mutated, variants are expressed, and their stability is measured experimentally.”*

*“These datasets are compilations of experimental stability measurements ~~of mutations~~ derived from several **small-scale** mutagenesis experiments of native proteins”*

5b.  Should lower right panel be AUROC?

Yes, it should, we have fixed this error. Thank you for pointing this out.

5c. Fig 4B: It seems like the best single mutant is as good as the best combination of mutants? Is this correct? Does this mean that combining mutants was unsuccessful? Or is the light blue point at the top not a single mutant? A different coloring scheme would be beneficial. The authors should be more explicit in the text about the best single mutant.

Yes, it is correct that the best single mutant is similar to the best combination of mutants, and have added a note of this to the main text. In the text, we do not make strong claims about the multi-mutation variants selected by ProteinDPO having higher stability than could be derived by additivity of the single-mutants. However, we do highlight that the five sets of ProteinDPO-identified nine-mutants are well tolerated by the structure and generally stabilizing.

*"While the best nine-substitution variant did not significantly surpass the best single-substitution variant, nine-substitution variants were 9.8 °C more stable on average than single-substitution variants."*

We give full details on the sequence and stability of all mutants in the Supplementary **(Supp. Table S6)**, to be more explicit we have added a note in the main text to refer to this table.

*"All variants and their associated melting temperatures are reported in the supplement (Table S6)"*

We have also adjusted the color scheme for a better distinction between the number of mutations.

5d. The CD wave scans look unusual- why is the signal so much weaker in DPO-19 and WT compared to DPO-4? If the proteins are folded and have the same structure, they should show the same spectrum. Is it possible the protein concentrations were measured incorrectly for all proteins except DPO-4? The methods should mention how protein concentrations were measured. A folded alpha-beta protein should have a signal with the intensity of the signal for DPO-4 (see a reference on this)- the other signals are strangely weak.

Yes, this was likely due to concentration differences. When collecting the CD wave scans, concentration was measured using a NanoDrop spectrophotometer and ensured to be in the range of 0.1-0.3 mg/ml as this was the guidance for using this particular CD spectrometer. It appears this window was too large between samples. We have updated the methods regarding how concentration was measured:

*"Protein samples **were** diluted or concentrated to ~~approximately 0.2 mg/ml~~ **concentration within the range of 0.1mg/ml to 0.3mg/ml** in PBS at 20°C. **Concentration was calculated using a Thermo Scientific NanoDrop OneC Microvolume Spectrophotometer measuring for absorbance at 280nm."***

and added a disclaimer to the main text.

*"Differences in absorbance signal magnitude are due to concentration differences between samples **(Methods)"***

5e. Methods: The authors should state which version of the Megascale dataset they used (v1 12/6/2022 or v2 4/20/2023).

We have added a statement of the version of the dataset used.

*"We used the Megascale dataset for training, either with our own training splits **(derived from v2 4/20/2023)**, those from Diekhaus et al, or those from Cuturello et al depending on subsequent evaluation."*

*"The Megascale dataset **(v2 4/20/2023)** served as the experimentally measured stability dataset from which we aligned the pretrained ESM-IF1."*

**Reviewer 3**

This work by Widatalla et al applies the DPO method to protein stability engineering problem. Protein stability engineering is clearly an important research subject, and the application of DPO technique to this problem is somewhat novel. There were several works in which protein language models were fine-tuned for the purpose, but to my knowledge, the application of DPO seems the first. I think validating their method through actual experiments is very encouraging and valuable; however, two major concerns listed below make it hard to fully appreciate the quality of the work.

We thank the reviewer for recognizing the importance of protein stabilization, the novelty of our approach, and our experimental validation. We are also grateful for the questions and recommended analyses, which have improved the manuscript.

1. The first is the basic philisophy of the work. From a fundamental point of view, is "tuning a pre-trained model" a relevant philosophy for the protein stability estimation? Regarding the complexity of protein folding and stability, I think this idea will only give a marginal improvement over existing methods sharing the same concept; i.e. another (slightly better) ThermoMPNN. A good work to compare is the recently published BioEmu, in which the authors simulated protein folding to approximate the equilibrium. I cannot see such conceptual novelty in this work that tackles the inherent challenge of the problem. Introducing DPO technique may add little novelty in this regard.

We thank the reviewer for raising the concerns regarding “tuning a pre-trained model” and its relevance to stability prediction. While protein folding stability is a complex phenomenon, additional training beyond pre-training is an effective approach, as several state-of-the-art models (e.g., ThermoMPNN and MSAesm_ddG [5, 3]), are fine-tuned pretrained language models. Moreover, BioEmu [8] gains its stability prediction through additional fine-tuning; after its pretraining task of learning to generate protein conformations, similarly to ProteinDPO, it undergoes a stability fine-tuning stage on the Megascale dataset [8].

Regarding BioEmu, we note that ProteinDPO achieves a higher Spearman correlation (~0.7) than BioEMu (~0.6) in regards to stability prediction as reported in the section **Predicting protein stabilities** in our manuscript and Fig. 4 in the BioEmu manuscript [8] (though we note that ProteinDPO and BioEmu used slightly different Megascale train and test splits). Importantly, while BioEmu showed its simulations recapitulated folded versus disordered states from proteins in the CALVADOS and ProthermDB databases, Importantly, BioEmu did not show generalizability of stability (ddG) prediction outside of the Megascale dataset, while we show ProteinDPO generalizes in stability prediction to FireProt and S669 **(Fig. 2D, 2E)**.

Regarding the comparison to ThermoMPNN, ProteinDPO is inherently different to ThermoMPNN and other ddG prediction models. ProteinDPO remains a generative model; instead of predicting a scalar value given a mutation (usually limited to only a single-substitution for most models), ProteinDPO produces a probability distribution of amino acids (biased towards stable sequences via DPO) given an input backbone, from which one can sample to generate an entire sequence. While we use comparisons to stability predictors to measure the optimization performance, ProteinDPO is fundamentally different as it remains a generative model. Remaining a generative model allows ProteinDPO to naturally score multi-mutation variants in-silico, as well as generate them in the lab. Furthermore, the use of structure-conditioned language models to design large portions of protein sequences given a backbone has been successful in many design applications spanning design protein binders, enzymes and membrane proteins [1, 4, 6, 12, 14]. ProteinDPO now enables structure-conditioned sequence design that is biased towards even greater stability, a desired attribute in many design applications.

2. The method is repeatedly compared against vanilla or STF, but I don't think this is a valuable comparison. This may show "how bad ESM-if" rather than "how good ProteinDPO" is for the problem. Also, it is hard to believe SFT in the manuscript was trained by their best. Therefore, it is worth testing just once for ablation, and the main comparison should be against other methods like ThermoMPNN. Also, the authors keep argue "method solely trained on small proteins generalizes to bigger protein". This is not an advantage but a prerequisite; methods not satisfying such property should be less appreciated.

Comparison to other methods is not very strong, but just based on the current figures, it is hard to identify clear advantages of the method. This marginal (or neglible) improvement should be related to the inherent limitation of the approach as pointed out in point 1.

Thank you for these comments. We compared ProteinDPO to ESM-IF1 (and SFT) across all evaluations since this is the model we initialize the weights with, and allows for specific testing of the hypothesis of whether DPO training improves scoring and generation performance. Comparing ProteinDPO to the vanilla ESM-IF1 and an SFTed model ensures we can controllably isolate the effect of DPO training, as the pretraining data, initial weights, parameter count, and featurization are the same between the three models. With regards to concerns that the results primarily reflect “how bad ESM-IF is”, we note that ESM-IF1 is generally considered a strong base model, for example having been used experimentally for enriching binding affinity in Shanker et al. [13]. Thus we see improving upon it as a valuable contribution.

Additionally, supervised fine-tuning is a good baseline methodology as it is widely used in almost all state-of-the-art language models for natural text [11] and which has shown success experimentally for biological applications [6, 12, 10, 7].

In regards to concerns that SFT was not trained properly, we have added additional training details to the **Methods** section **(Supervised Finetuning)**, and included loss curves of the learning rate sweeps that were conducted to find an optimal checkpoint in the supplemental **(Fig. S2)**. Across different SFT training runs, we find that all checkpoints perform similarly:

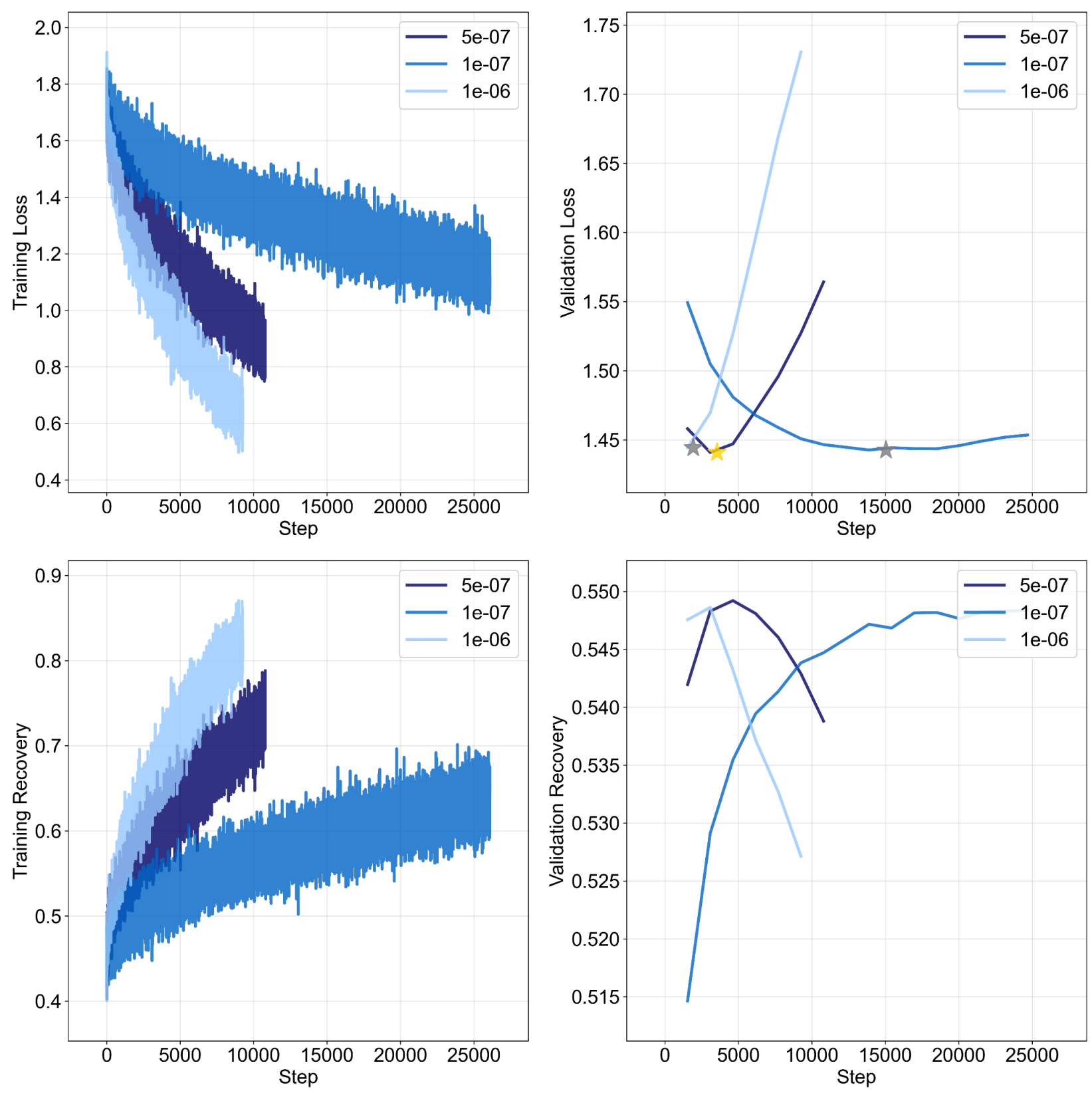


*Figure S2 | Training and validation loss and sequence recovery curves for training the supervised finetuned model. The selected checkpoint was the checkpoint which best minimized validation loss across all runs, and is denoted with a star. Checkpoints which minimized validation loss for other runs, are denoted with a gray star, and are compared against the selected checkpoint in* ***Table S7.***

| Learning rate | Megascale (mean) | FireProt | AB-Bind (mean) | Antibody thermal stability |
|---|---|---|---|---|
| 1e-7 | 0.584/0.561 | 0.430/0.441 | 0.10/0.05 | 0.250/0.236 |
| 5e-7 | 0.588/0.570 | 0.415/0.429 | 0.10/0.06 | 0.258/0.246 |
| 1e-6 | 0.577/0.556 | 0.422/0.433 | 0.10/0.04 | 0.249/0.235 |

*Table S7 | Comparison of different SFT checkpoints trained with varying learning rate on stability and binding prediction in terms of (mean) Pearson R / Spearman ρ correlation coefficients.*

Regarding ThermoMPNN, we benchmarked this model across several datasets (MegaScale single-mutants, Megascale double-mutants, S669, and Fireprot). However, we could not benchmark against ThermoMPNN for SKEMPI, AB-Bind or Antibody thermostability due to the inability of ThermoMPNN to score complexes or absolute differences in highly diverse sequences (e.g., those with different length). In lieu of ThermoMPNN, we have added benchmarking of ProteinMPNN and Rosetta, for protein-protein binding and antibody thermostability.

We also agree with the reviewer that generalization of a model to larger proteins is a "prerequisite, not advantage" for any model that ought to be used to predict protein stability. Notably, many previous machine learning models have had a tendency to overfit on their respective domain and to fail to generalize to larger proteins. A recent work featuring a Megascale-fine-tuned version of the ESM protein language model experienced this; despite performing well on prediction for a MegaScale test set, this model did not generalize to other DMS datasets, where the authors of that study cited a mismatch in the distribution of sequence lengths [2].

Lastly, we agree with the reviewer that for certain tasks, such as protein-protein binding, and antibody-antigen binding, the improvements from the base model are modest. However, for stability prediction, our performance improvements are indeed meaningful. Specifically, we see large improvements in stability prediction in the Megascale holdout set (Pearson $R$ and Spearman $\varrho$ improvements of 0.16 to 0.19), and the S669 and FireProt datasets (Pearson $R$ and Spearman $\varrho$ increases of 0.13 to 0.16). These improvements generalize to ranking absolute stability of diverse antibodies Pearson $R$ and Spearman $\varrho$ improvements from 0.08 to 0.12). Additionally, in **Figure 3D**, we see how these differences in scoring translate to a clear difference in the predicted stability of full sequence generations when evaluated by an orthogonal physics-based approach, Rosetta.

3. This is a suggestion for the presentation of the experimental section. As I mentioned above, experimental validation is very encouraging. At the same time, readers may wonder what if other already-established method was tested in parallel. For example, ProteinMPNN already demonstrated its ability to improve stability in various previous works other methods (e.g. Rosetta) failed. It should have been much more impactful if ProteinDPO have proved experimetnal success in a similar way (although a relevant baseline now has largely shifted from Rosetta to ProteinMPNN since then). Otherwise readers may have impression that results presented were cherry-picked.

We are grateful for this comment, which is similar to comments from the two other reviewers. On the task of HA stabilization, we now benchmark against ESM-IF1, SFT, ThermoMPNN, and ProteinMPNN in these models' ability to highly rank stabilizing mutations (1) from the literature and (2) out of the 30 single-mutations which were experimentally validated in our first revision, in part following the guidance of Reviewer 2. When comparing the ability of ESM-IF1, SFT, ThermoMPNN, ProteinMPNN and ProteinDPO to recover known HA-stabilizing mutations from the literature, specifically Milder et al [9] and Yassine et al [15] **(Fig. S5)**, we found that while some models were able to recover one mutation in their top-50, ProteinDPO has a higher recovery rate across all top-n levels.

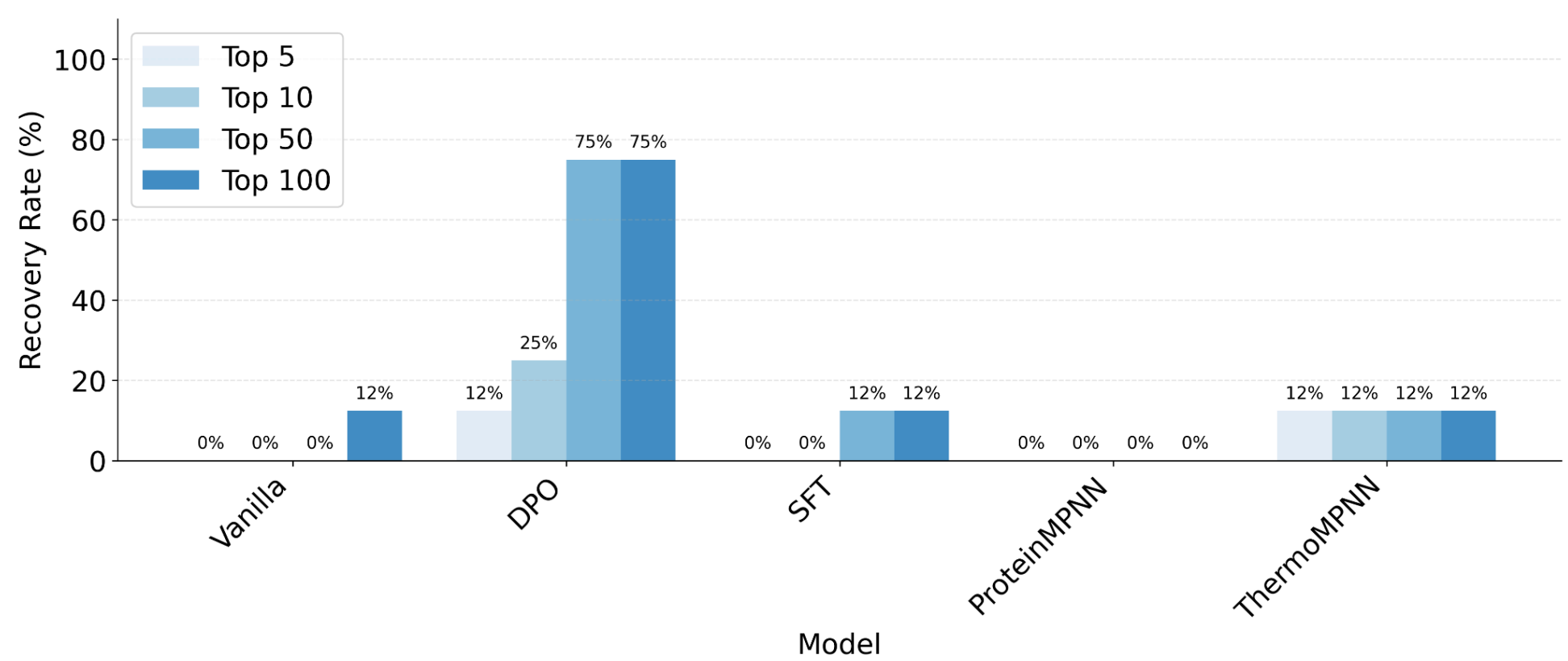


*Figure S5 | Recovery of literature-validated stabilizing mutations across protein design models. Bar plot showing the percentage of eight known stabilizing mutations (Milder et al. and Yassine et al.) recovered within the top-ranked predictions of each model. Models were evaluated using ensemble scoring across three PDB structures (6cf5, 6cfg, 2fk0). n=8 total literature mutations.*

Additionally, we compared the ability of ESM-IF1, SFT, ProteinMPNN and ThermoMPNN to rank the 6/30 highly-stabilizing (>= 9°C) mutations from the pool of 30 single-mutations we tested **(Fig. S6)**. Only the SFT model and ThermoMPNN were able to recover one of these mutations in its top-100.

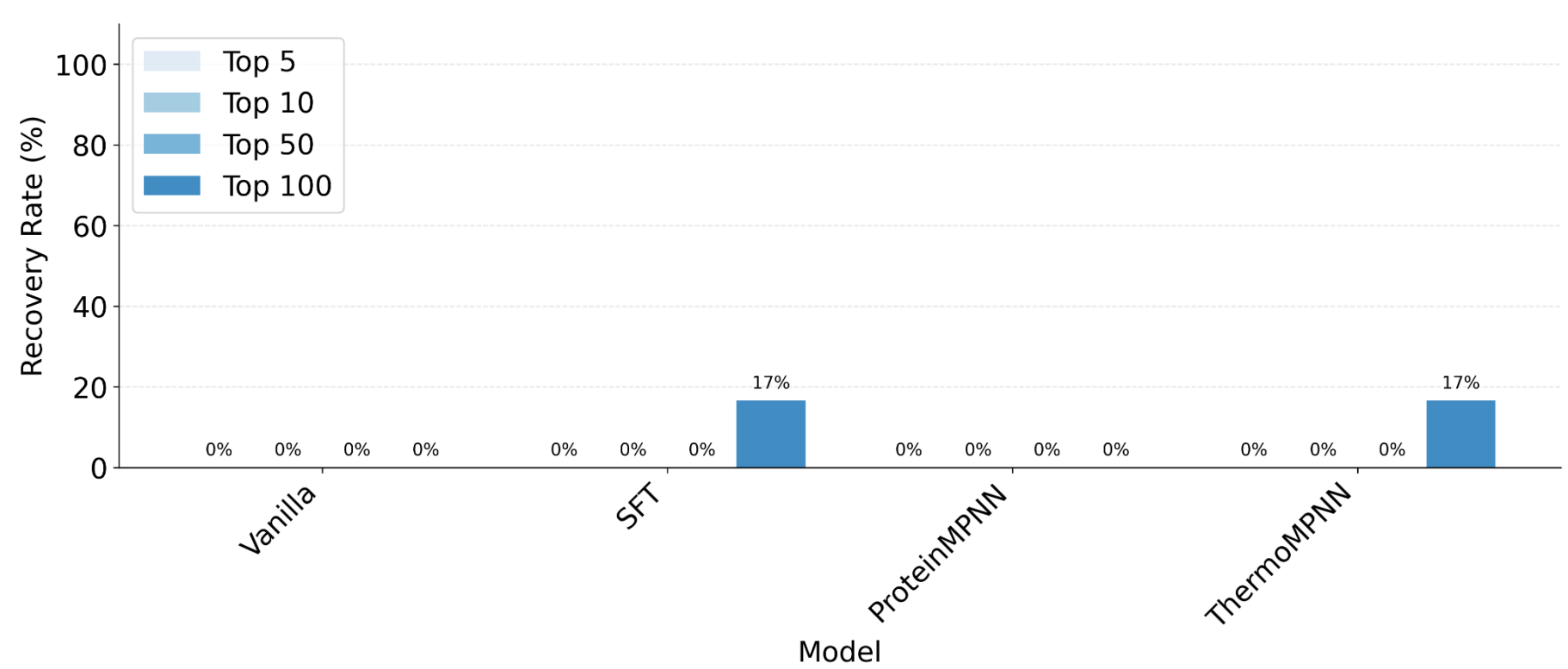

*Figure S6 | Recovery of experimentally tested DPO mutations by baseline models. Bar plot showing the percentage of six significantly stabilizing (>= 9◦C) DPO-identified mutations recovered within the top-ranked predictions of each model. Models were evaluated using ensemble scoring across three PDB structures (6cf5, 6cfg, 2fk0).*

Both analyses suggest that ProteinDPO is superior in highly ranking literature mutations, enabling low-n stabilization. Additionally the compared methods are not able to recover most of the best performing ProteinDPO mutations. We do note that a limitation of this test is that some of the highly ranked mutations by other methods may be stabilizing, which we also acknowledge in our text:

*'Only the SFT model and ThermoMPNN were able to recover one of these mutations in its top-100; however, it is possible top-ranked mutants from these baseline models are also stabilizing."*

4. I was not able to install the code with the error message below:

"Solving environment: failed

ResolvePackageNotFound:

- python[version='3.10.*,==3.9.18',build=h955ad1f_0]"

I do see this issue was posted in the github a bit ago, but was not fixed. Please fix this issue before your resubmission. The inference code itself is very simple and straightforward.

Thank you for bringing this to our attention, the installation issue has been fixed in commit #2ef299f.

## References


1. Bennett, N. R. *et al.* Atomically accurate de novo design of antibodies with RFdiffusion. *Nature* (2025). https://doi.org/10.1038/s41586-025-09721-5
2. Chu, S. K. S., Narang, K. & Siegel, J. B. Protein stability prediction by fine-tuning a protein language model on a mega-scale dataset. *PLOS Comput. Biol.* **20**, e1012248 (2024). https://doi.org/10.1371/journal.pcbi.1012248
3. Cuturello, F., Celoria, M., Ansuini, A. & Cazzaniga, A. Enhancing predictions of protein stability changes induced by single mutations using MSA-based Language Models. *bioRxiv* (2024). https://doi.org/10.1101/2024.04.11.589002
4. Dauparas, J. et al. Robust deep learning-based protein sequence design with ProteinMPNN. *Science* **378**, 49-56 (2022). https://doi.org/10.1126/science.add2187
5. Dieckhaus, H., Brocidiacono, M., Randolph, N. Z. & Kuhlman, B. Transfer learning to leverage larger datasets for improved prediction of protein stability changes. *Proc. Natl. Acad. Sci. U.S.A.* **121**, e2314853121 (2024). https://doi.org/10.1073/pnas.2314853121
6. Goverde, C. A. *et al.* Computational design of soluble and functional membrane protein analogues. *Nature* **631**, 449–458 (2024). https://doi.org/10.1038/s41586-024-07601-y
7. King, S. H. *et al.* Generative design of novel bacteriophages with genome language models. *bioRxiv* (2025). https://doi.org/10.1101/2025.09.12.675911
8. Lewis, S. *et al.* Scalable emulation of protein equilibrium ensembles with generative deep learning. *Science* **389**, eadv9817 (2025). https://doi.org/10.1126/science.adv9817
9. Milder, F. J. *et al.* Universal stabilization of the influenza hemagglutinin by structure-based redesign of the pH switch regions. *Proc. Natl. Acad. Sci. U.S.A.* **119**, e2115379119 (2022). https://doi.org/10.1073/pnas.2115379119
10. Nguyen, E. *et al.* Sequence modeling and design from molecular to genome scale with Evo. *Science* **386**, eado9336 (2024). https://doi.org/10.1126/science.ado9336
11. Ouyang, L. *et al.* Training language models to follow instructions with human feedback. *Adv. Neural Inf. Process. Syst.* **35**, 27730–27744 (2022).
12. Pacesa, M. *et al.* One-shot design of functional protein binders with BindCraft. *Nature* (2025). https://doi.org/10.1038/s41586-025-09429-6
13. Shanker, V. R., Bruun, T. U. J., Hie, B. L. & Kim, P. S. Unsupervised evolution of protein and antibody complexes with a structure-informed language model. *Science* **385**, 46–53 (2024). https://doi.org/10.1126/science.adk8946

14. Watson, J. L. *et al.* De novo design of protein structure and function with RFdiffusion. *Nature* **620**, 1089–1100 (2023). https://doi.org/10.1038/s41586-023-06415-8

15. Yassine, H. M. *et al.* Hemagglutinin-stem nanoparticles generate heterosubtypic influenza protection. *Nat. Med.* **21**, 1065–1070 (2015). https://doi.org/10.1038/nm.3927
